# Supplementary material for: Association between antidepressant use during pregnancy and miscarriage: a systematic review and meta-analysis
Source: BMJ Open. 2024 Jan 25;14(1):e074600. doi: 10.1136/bmjopen-2023-074600 (PMC10824002; doi:10.1136/bmjopen-2023-074600)
Supplement: Supplementary data [file bmjopen-2023-074600supp002.pdf]

EMBASE

Number	Search term	Outputs
1	exp antidepressant agent/	544895
2	Serotonin uptake inhibitor/	52823
3	Monoamine oxidase inhibitor/	15008
4	(anti*depress or antidepress* or tricyclic* or TCA* or heterocyclic* or serotonin uptake or SSRI* or SNRI* or monoamine oxidase inhibitor* or MAOI*).tw.	176810
5	(desipramine or imipramine or clomipramine or opipramol or trimipramine or lofepramine or dibenzepin or amitriptyline or nortriptyline or protriptyline or doxepin or iprindole or melitracen or butriptyline or dosulepin or amoxapine or dimetacrine or amineptine or maprotiline or quinupramine or zimeldine or fluoxetine or citalopram or paroxetine or sertraline or alaproclate or fluvoxamine or etoperidone or escitalopram or isocarboxazid or nialamide or phenelzine or tranylcypromine or iproniazide or iproclozide or moclobemide or toloxatone or oxitriptan or tryptophan or mianserin or nomifensine or trazodone or nefazodone or minaprine or bifemelane or viloxazine or oxaflozane or mirtazapine or bupropion or medifoxamine or tianeptine or pivagabine or venlafaxine or milnacipran or reboxetine or gepirone or duloxetine or agomelatine or desvenlafaxine or vilazodone).tw.	144967
6	1 or 2 or 3 or 4 or 5	673317
7	exp stillbirth/	22575
8	exp perinatal mortality/	27721
9	exp perinatal death/	4865
10	exp fetus death/	43412
11	(stillbirth or stillborn or perinatal death or peri-natal death or neonatal death or intrauterine death or intra-uterine death or newborn mortality).tw.	30264
12	exp spontaneous abortion/	49635
13	recurrent abortion/	7454
14	(Spontaneous abortion or Miscarriage$).tw.	39878
15	7 or 8 or 9 or 10 or 11	80549
16	12 or 13 or 14	62656

17	6 and 15	1176
18	6 and 16	1424
19	17 or 18	2340

MEDLINE

Number	Search term	Output
	exp Antidepressive Agents/	160660
	exp Serotonin Uptake Inhibitors/	46158
	exp Monoamine Oxidase Inhibitors/	22716
	(anti*depress or antidepress* or tricyclic* or TCA* or heterocyclic* or serotonin uptake or SSRI* or SNRI* or monoamine oxidase inhibitor* or MAOI*).tw.	132787
	(desipramine or imipramine or clomipramine or opipramol or trimipramine or lofepramine or dibenzepin or amitriptyline or nortriptyline or protriptyline or doxepin or iprindole or melitracen or butriptyline or dosulepin or amoxapine or dimetacrine or amineptine or maprotiline or quinupramine or zimeldine or fluoxetine or citalopram or paroxetine or sertraline or alaproclate or fluvoxamine or etoperidone or escitalopram or isocarboxazid or nialamide or phenelzine or tranylcypromine or iproniazide or iproclozide or moclobemide or toloxatone or oxitriptan or tryptophan or mianserin or nomifensine or trazodone or nefazodone or minaprine or bifemelane or viloxazine or oxaflozane or mirtazapine or bupropion or medifoxamine or tianeptine or pivagabine or venlafaxine or milnacipran or reboxetine or gepirone or duloxetine or agomelatine or desvenlafaxine or vilazodone).tw.	122965
	1 or 2 or 3 or 4 or 5	304323
	Stillbirth/	6346
	Perinatal Mortality/	2478
	exp Fetal Death/	31204
	PERINATAL DEATH/	2104
	(stillbirth or stillborn or perinatal death or peri-natal death or neonatal death or intrauterine death or intra-uterine death or newborn mortality).tw.	22241
	7 or 8 or 9 or 10 or 11	47887

	exp Abortion, Spontaneous/	38966
	(Spontaneous abortion or Miscarriage$).tw.	24795
	13 or 14	52292
	6 and 12	98
	6 and 15	202
	16 or 17	273

PSYCINFO

	Search terms	Outputs
	exp Antidepressant Drugs/	41562
	exp Serotonin Reuptake Inhibitors/	14269
	exp Monoamine Oxidase Inhibitors/	2318
	(anti*depress or antidepress* or tricyclic* or TCA* or heterocyclic* or serotonin uptake or SSRI* or SNRI* or monoamine oxidase inhibitor* or MAOI*).tw.	50486
	(desipramine or imipramine or clomipramine or opipramol or trimipramine or lofepramine or dibenzepin or amitriptyline or nortriptyline or protriptyline or doxepin or iprindole or melitracen or butriptyline or dosulepin or amoxapine or dimetacrine or amineptine or maprotiline or quinupramine or zimeldine or fluoxetine or citalopram or paroxetine or sertraline or alaproclate or fluvoxamine or etoperidone or escitalopram or isocarboxazid or nialamide or phenelzine or tranylcypromine or iproniazide or iproclozide or moclobemide or toloxatone or oxitriptan or tryptophan or mianserin or nomifensine or trazodone or nefazodone or minaprine or bifemelane or viloxazine or oxaflozane or mirtazapine or bupropion or medifoxamine or tianeptine or pivagabine or venlafaxine or milnacipran or reboxetine or gepirone or duloxetine or agomelatine or desvenlafaxine or vilazodone).tw.	35997
	(stillbirth or stillborn or perinatal death or peri-natal death or neonatal death or intrauterine death or intra-uterine death or newborn mortality).tw.	1195
	exp Spontaneous Abortion/	980
	(Spontaneous abortion or Miscarriage$).tw.	1706
	1 or 2 or 3 or 4 or 5	72741

	7 or 8	1981
	9 and 6	15
	9 and 10	46
	11 or 12	56
