# Supplementary material for: Association between antidepressant use during pregnancy and miscarriage: a systematic review and meta-analysis
Source: BMJ Open. 2024 Jan 25;14(1):e074600. doi: 10.1136/bmjopen-2023-074600 (PMC10824002; doi:10.1136/bmjopen-2023-074600)
Supplement: Supplementary data [file bmjopen-2023-074600supp001.pdf]

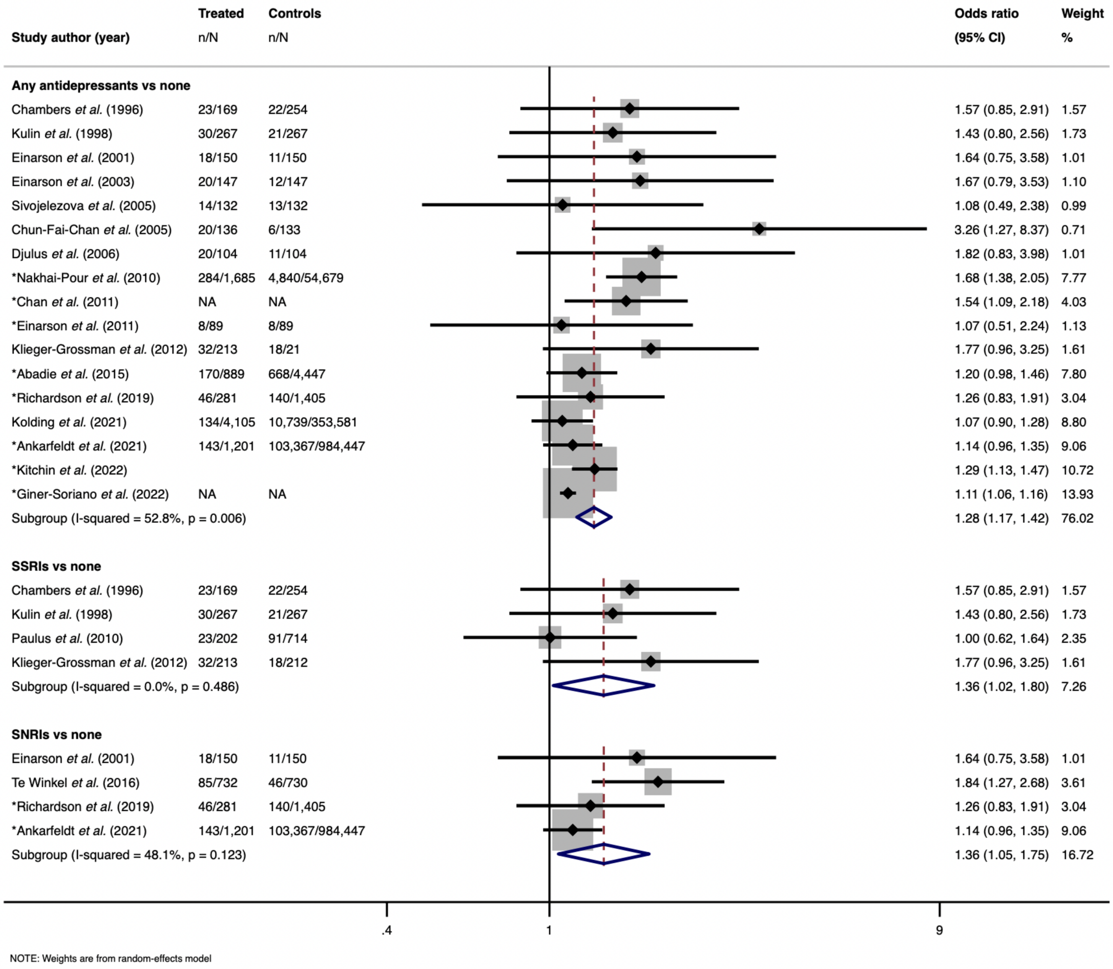

S1 Figure. Sensitivity analysis utilising only papers reporting odds ratios.

General population sensitivity analysis including only papers which included odds ratio, and excluding papers reporting hazards and risk ratio

*These studies had an adjusted estimate from multivariable model which was pooled in the meta-analysis rather than raw data, therefore the treated and controls (n/N) data does not correspond to the odds ratio shown.
