# Supplementary material for: Association between antidepressant use during pregnancy and miscarriage: a systematic review and meta-analysis
Source: BMJ Open. 2024 Jan 25;14(1):e074600. doi: 10.1136/bmjopen-2023-074600 (PMC10824002; doi:10.1136/bmjopen-2023-074600)
Supplement: Supplementary data [file bmjopen-2023-074600supp004.pdf]

S2 Table. Excluded studies

**Study excluded at full-text screening**	**Reason**
Lennestal et al. (2007)	Stillbirth outcome only
Sjaarda et al. (2020)	Combined outcome of both stillbirth and miscarriage
Stephansson et al. (2013)	Stillbirth outcome only
Stephens et al. (2011)	Wrong study design
Wen et al. (2006)	Stillbirth outcome only
Winterfeld et al. (2015)	Combined outcome of both stillbirth and miscarriage
Pastuszak et al. (1993)	No outcome data
Ramio et al. (2007)	Wrong study design
Robinson-Wolrath et al. (2016)	No outcome data
Sjaarda et al. (2019)	Duplicate
Andersen et al. (2013)	Duplicate
Burian et al. (2016)	No outcome data
Einarson et al. (2011)	No comparator
Einarson et al. (2009)	Duplicate
Jordan et al. (2016)	Wrong outcome
Einarson et al. (2011)	No comparator
Evans-Hoeker et al. (2017)	Duplicate
Klieger-Grossman et al. (2010)	Duplicate
Goldstein et al. (1997)	No comparator
Hoog et al. (2013)	No comparator
Einarson et al. (2012)	No outcome data
Innocenti et al. (2019)	No outcome data
Jimenez-Solem et al. (2014)	Stillbirth outcome only
Jimenez-Solem et al. (2013)	Stillbirth outcome only
Ericson et al. (1999)	Stillbirth outcome only
