# Supplementary material for: Association between antidepressant use during pregnancy and miscarriage: a systematic review and meta-analysis
Source: BMJ Open. 2024 Jan 25;14(1):e074600. doi: 10.1136/bmjopen-2023-074600 (PMC10824002; doi:10.1136/bmjopen-2023-074600)
Supplement: Supplementary data [file bmjopen-2023-074600supp005.pdf]

Table S3. Characteristics of included studies

Study	Study design	Country of study & study period	Data sampling methods & mean age of individuals	Type of AD exposure	Timing of AD exposure	Definition of exposure and comparator groups	Identification of exposure	Identification of outcome	Sample size	Confounding adjustments	Raw data (no. of SA/ total)	Adjusted estimates
Chambers et al. (1996) [1]	Prospective cohort	California 1989-1995	TIS data collection<br><br>**Exposed early:** 32+-5<br>**Exposed late:** 32+-6<br>**Control:** 30+-5	Fluoxetine (SSRI)	Anytime	**Exposed:** Fluoxetine use<br>**Comparator group 1:** women enquiring about non-teratogens	Telephone interviews	Telephone interview and confirmed by physician	482	Maternal age, average dose of fluoxetine	**Exposed early =** 23/169<br>**Control group** = 22/254	NA
Kulin et al. (1998) [2]	Prospective cohort	US&Canada	TIS data<br><br>**Exposed :** mean = 31.3<br>**Unexposed:** mean = 30.8	SSRI	First trimester	**Exposed:** SSRI use during 1st trimester<br>**Comparator group 1:** Women enquiring about non-teratogens	Telephone interview	After expected date of delivery women were interviewed	534	None	**Exposed** = 30/267<br>**Control** = 21/267	NA
Einarson et al. (2001) [3]	Cohort	European and non-european countries	Women contacting motherisk program around exposure advice<br><br>NA	Venlafaxine (SNRI)	Anytime	**Exposed:** Venlafaxine use during 4th-14th week of pregnancy<br>**Comparator group 1:** SSRI users<br>**Comparator group 2:** women making non-teratogenic enquiries	Once contacted the service women were interviewed	Follow-up questionnaire after delivery and information verified by physician	450	None	**Venlafaxine =** 18/150<br>**SSRI comparator** = 16/150<br>**Nonteratogenic comparator =** 11/150	NA
Einarson et al. (2003)[4]	Prospective cohort	European and non-european countries	Women contacting motherisk program<br><br>NA	Trazodone and Nefazodone (NaSSA)	First trimester	**Exposed:** Use of NaSSAs during 4th-14th week<br>**Comparator group 1:** Women taking other ADs<br>**Comparator group 2**: non-teratogen drug exposures	Questionnaire	Follow-up questionnaire after delivery and verified by physician	441	Matched on gestational age	**Trazodone/Nefazodone** = 20/147<br>**Group 1 (other Ads)** = 17/147<br>**Group 2 – nonteratogens** = 12/147	NA
Sivojelezova et al. (2005) [5]	Prospective cohort	Toronto 1999-2002	Women contacting motherisk program<br><br>Range of 18-42 and mean of 31.9	Citalopram (SSRI)	Anytime	**Exposed:** Use of citalopram<br>**Comparator group 1:** pregnant women taking other SSRI Ads<br>**Comparator group 2:** non-teratogenic drug use	Telephone interview to individuals contacting the service	Follow-up questionnaire after delivery and verified by physician	396	None	**Exposed** = 14/132<br>**Comparison 1 =** 13/132<br>**Comparison 2 =** 13/132	NA

Chun-Fai Chan et al. (2005) [6]	Cohort	European and non-european countries	Individuals contacting motherisk or recruited through drug safety research unit<br><br>NA	Bupropion (NDRI)	Anytime	**Exposed:** Bupropion exposed pregnancies **Comparator group 1:** Pregnant women taking other Ads **Comparator group 2:** individuals taking non-teratogens	Telephone administration of follow-up questionnaire	Follow-up questionnaire after delivery and verified by physician	NA	Matched on age, alcohol consumption, smoking and gestational age	**Bupropion =** 20/136 **Comparison 1 (other Ads) =** 11/89 **Comparison 2 (nontertaogens) =** 6/133 **Women exposed to bupropion for depression =** 14/91	NA
Djulus et al. (2006) [7]	Cohort	European and non-european countries 2002-2005	Pregnant women contacting one of five evidence based information centres<br><br>Ages were consistent between groups due to matching	Mirtazapine (NaSSA)	Anytime	**Exposed:** pregnant women taking bupropion **Comparator group 1:** disease matched individuals taking other Ads **Comparator group 2:** individuals taking known non-teratogens	Telephone interview regarding exposure	Contacted 2-6 months after delivery and details verified by physician	312	Matched on maternal age, gestational age, tobacco use, alcohol consumption and chronic conditions	**Mirtazapine =** 20/104 **Comparison 1 (other AD) =** 18/104 **Comparison 2 (nonteratogenics) =** 11/104	NA
Einarson et al. (2009) [8]	Prospective cohort	Toronto	women contacting motherisk program<br><br>NA	Any AD	Anytime	**Exposed:** Pregnant women contacting program regarding exposure during pregnancy **Comparator group 1:** Women exposed to non-teratogenic drugs **Comparator group 2:** unexposed to Ads	Telephone interview using standardised questionnaire	Follow-up structured form	1874	Matched for maternal age, smoking, and alcohol use, gestational age	**Exposed =** 122/937 **Unexposed =** 75/937	RR = 1.38 (0.98-1.94)
Nakhai-Pour et al. (2010) [9]	Nested case-control	Quebec 1998-2003	Data from Quebec pregnancy register with individuals registered between 1998-2003, including RAMQ database<br><br>NA	Any AD	Any time between first day gestation and index date of SA	**Exposed:** At least one prescription filled between start of gestation and SA index date 10 controls sampled for every SA case	RAMQ database which collects information on prescriptions, and pregnancy register to identify unexposed	Quebec pregnancy register entered between 1998-2003	56364	Adjusted for age, urban residence, social assistance, psychiatric diagnosis, comorbid illness, medication use, gestational age	**Cases =** 284 individuals used antidepressants **Controls =** 1401 used antidepressants	**Crude OR =** 2.09 (1.83-2.38) **Adjusted OR =** 1.68 (1.38-2.06) **Adjusted SSRI alone** = 1.61 (1.28-2.04) **Adjusted TCA alone** = 1.27 (0.85-1.91)
Paulus et al. (2010) [10]	Prospective cohort: Conference abstract	Germany	National TIS who were contacted by	Paroxetine (SSRI)	First trimester	**Exposed:** Paroxetine exposure in first trimester	Patients or physicians contact TIS	NA	955	None	**Exposed** = 23/202 **Unexposed =** 81/714	NA

			physicians or patients			**Comparator group 1:** Not exposed or 'severely exposed' to paroxetine						
Chan et al. (2011) [11]	Cohort – conference abstract	US 2000-2008	Recruited from 4 US metropolitan areas	All Ads	Anytime	**Exposed:** Antidepressant use during pregnancy<br>**Comparator:** NA	Telephone interviews and medical record abstractions	Telephone interviews and medical record abstractions	4536	Maternal age, race, education, BMI, smoking, alcohol use, gravidity and nausea	NA	**Adjusted OR of exposed vs unexposed** = 1.54 (1.1-2.2)<br>**Adjusted OR of SSRI users vs unexposed** = 1.67
Einarson et al. (2011) [12]	Prospective cohort	Toronto 1992-2007	women contacting motherisk	All Ads	Anytime	**Exposed:** exposed to more than one AD during pregnancy<br>**Comparator group 1:** Exposed to single AD during pregnancy<br>**Comparator group 2:** contacting motherisk regarding non-teratogenic exposure	telephone interview using standardised questionnaire	Follow-up interview details verified from report by physician	267	Matched on maternal age, smoking and alcohol use and gestational age	**Multiple AD exposure** = 8/89<br>**Single AD exposure** = 7/89<br>**No AD exposure** = 8/89	**OR for multiple AD exposure vs single exposure** = 1.16 (0.4-3.34)<br>**OR for multiple AD exposure vs no exposure** = 1.00 (0.36-2.79)
Ban et al. (2012) [13]	Cohort	UK 1990-2009	Data collected from primary care medical records (through health improvement network - THIN)<br><br>> 50% of the women aged between 25-34	TCAs and SSRIs	First trimester	**Exposed:** depression and exposed to AD during first 90 days after conception<br>**Comparator group 1L** No history of depression and unexposed<br>**Comparator group 2:** history of depression and unexposed<br>**Comparator group 3:** prescriptions for TCAs only<br>**Comparator group 4:** prescriptions for SSRIs only<br>**Comparator group 5:** prescriptions for any other AD only<br>**Comparator group 6:** prescription for psychotropic drugs only	Extracted prescription records from THIN	Identified through THIN database	331414	Adjusted for maternal age, SES, maternal smoking status and BMI	**Reference category (group 1) =** 47258/390665<br>**History of mental illness only =** 13814/99932<br>**Unmedicated current mental illness** = 442/3647<br>**TCAs** = 443/3019<br>**SSRIs=** 1539/10132	<u>**Adjusted RRR with comparison being no current/past depression or anxiety**</u><br>**History of mental illness only:** 1.2 (1.2-1.2)<br>**Unmedicated mental illness** = 1.0 (0.9-1.2)<br>**TCAs** = 1.3 (1.1-1.5)<br>**SSRIs** = 1.5 (1.3-1.6)<br><br><u>**Adjusted RRR when compared to unmedicated antenatal depression or anxiety**</u><br>**TCAs** = 1.3 (1.1-1.5)<br>**SSRIs** = 1.4 (1.2-1.7)
Klieger-Grossman et al. (2012) [14]	Prospective cohort	Toronto, Switzerland and Florence	Data collected from women ringing TIS in the three countries	Escitalopram (SSRI)	Anytime	**Exposed:** pregnant women exposed to escitalopram<br>**Comparator group 1:** contacting service exposed to other Ads	Information taken during telephone conversation through standardised questionnaire	Follow-up questionnaire 2-3 months after delivery	637	Matched on maternal age, alcohol consumption, smoking and gestational age	**Escitalopram** = 32/213<br>**Other AD use** = 34/212<br>**Nonteratogens** = 18/212	NA

			Mean age of all women in study = 33.1			**Comparator group 2:** nonteratogenic exposures						
Kjaesgaard et al. (2013) [15]	Population based cohort	Denmark 1997-2008	Data obtained from Danish health registries<br><br>**Depression and no AD use:** mean = 29.1<br>**Use of AD (exposed):** mean = 30.9<br>**Unexposed:** mean = 30.2	All Ads	Anytime	**Exposed:** mother redeemed prescription for AD<br>**Comparator group 2:** mother hadn't redeemed prescription	Information from registry of medicinal product statistics	Identified through codes using Danish national hospital registry	1005319	Adjusted for maternal age, cohabitation, income, education, history of mental disorder or drug abuse	**Diagnosis of depression and exposed to AD** = 210/1674<br>**Diagnosis of depression and unexposed to AD**= 105/820<br>**No diagnosis of depression and AD use**= 2427/13789<br>**No diagnosis of depression and unexposed**= 110377/818426	**<u>Unadjusted RR when compared to unexposed equivalent:</u>**<br>**Diagnosis of depression exposed to AD** = 0.98 (0.78-1.23)<br>**No diagnosis of depression and AD use** = 1.31 (1.26-1.35)<br><br>**<u>Adjusted RR (when compared to unexposed equivalent):</u>**<br>**Diagnosis of depression and exposed** = 1.00 (0.8-1.24)<br>**No diagnosis of depression and AD use** = 1.17 (1.13-1.22)
Andersen et al. (2014)[16]	Cohort	Denmark 1997-2010	Identified all registered pregnancies through national hospital register	SSRIs	First 35 days of pregnancy	**Exposed:** Dispensing of prescription of SSRIs<br>**Comparator group 1:** Women exposed to SSRIs prior to pregnancy<br>**Comparator group 2:** women unexposed to SSRIs	Data collected from national prescription register containing individual level data on prescribed drugs	Identified through national hospital register using codes	1279840	Adjusted for maternal age, previous SA, income, year of outcome, educational length, antipsychotics and thyroid preparations	**Unexposed=** 139201/1256956<br>**Exposed to SSRI during first 35 days of pregnancy:** 2883/22884<br>**Discontinuation of SSRI treatment 3-12 months before conception =** 1936/14016	**<u>Unadjusted HR:</u>**<br>**Exposed during first 35 days vs unexposed:** 1.31 (1.27-1.36)<br>**Discontinued SSRI treatment 3-12 months before pregnancy vs unexposed:** 1.38 (1.32-1.44)<br><br>**<u>Adjusted HR:</u>**<br>**Exposed to SSRI during first 35 days vs unexposed:** 1.27(1.22-1.33)<br>**Discontinued use of SSRI before conception vs unexposed:** 1.24 (1.18-1.30)

Abadie et al. (2015)[17]	Nested case-control	France	French database of TERAPPEL used where healthcare professionals contact French regional pharmacovigilance centres (CPRV)<br><br>**Mean of cases** = 32.4<br>**Mean of controls** = 30.6	All Ads	First trimester	**Exposed:** First trimester drug exposure and called CPRV enquiring about drug exposure<br>**Comparator group 1:** Calling CPRV for assessing non-teratogen exposure	Healthcare professionals contacting CPRVs	Women registered to TERAPPEL database	5346	Maternal age	**Exposed to any AD**<br>**Cases of SA =** 170/838<br>**No SA** = 729/4508<br><br>**Exposed to non-selective monoamine reuptake inhibitors (NMRI)**<br>**SA** = 39/838<br>**No SA** = 88/4508	**Crude OR for all Ads** = 1.32 (1.1-1.59)<br>**Adjusted OR for all Ads** = 1.2 (0.98-1.46)<br><br>**Crude OR for NMRI=** 2.45 (1.67-3.60)<br>**Adjusted OR for NMRI** = 2.19 (1.46-2.39)
Johansen et al. (2015) [18]	Register based study cohort	Denmark 1996-2009	study population with registered pregnancies between 1996-2009 and Danish national birth cohort	SSRI	Anytime but results split into first and second trimester	**Exposed:** Redeemed prescription in time frame of month prior to LMP to 3 months after LMP or end of pregnancy (also self-report)<br>**Comparator group 1:** discontinued use of SSRIs with same LMP prescription timeframe<br>**Comparator group 2:** unexposed and no prescription	Redeemed prescriptions from Danish national prescription database	Data from Danish national patient register	1281418	Adjustment for household income, education, previous SA, parity and maternal age	**Unexposed** = 124333/1165124<br>**Discontinued use** = 670/5428<br>**Exposed** = 2359/20612	**Combined first and second trimester HR**<br><br>**Exposed vs unexposed general population** = 1.07 (1.03-1.12)<br>**SSRI use vs unmedicated depressed** = 0.97 (0.81-1.16)
Te Winkel et al. (2016) [19]	Prospective cohort – conference abstract only	Across European countries	Data from nine centres which make up european network of TIS<br><br>Mean: 31.9	Venlafaxine (SSRI)	Anytime	**Exposed:** Venlafaxine exposure<br>**Comparator group 1:** No exposure to any known teratogens	TIS data	using standardised procedures for data collection and follow-up	1462	None	**Exposed =** 85/732<br>**Unexposed =** 46/730	NA
Almeida et al. (2016) [20]	Retrospective cohort	Quebec 1998-2002	Linking four population-based registries and producing longitudinal histories of medication use	All Ads	First trimester	**Exposed:** Women who have at least one AD prescription in first trimester of pregnancy<br>**Comparator groups 1:** Unexposed but depressed in last four years<br>**Comparator group 2:** non-teratogen group	Prescription claims database	ICD-9 codes	41964	Adjustment for maternal age, welfare recipient, other teratogenic medication, no. medications any hospitalisatio	**No medication and no depression:** 1685/28343<br>**No AD but depression -** 720/10719<br>**AD use in first trimester and depression -** 165/1955	**All adjusted RRR**<br><br>**Unexposed and no depression** = 1.00<br>**Unexposed and depressed =** 1.1 (1.0-1.2)<br>**Exposed and depressed =** 1.3 (1.1-1.5)

						**Comparator group 3:** individuals with no prescriptions or depression				n or physician visits, calendar year	**Hypothyroid medication use in first trimester:** 71/947	
Evans-Hoeker et al. (2018) [21]	Cohort	US	RCT was conducted on non-IVF fertility treatment and individuals from trial were brought into cohort study	All Ads – split into SSRI and non-SSRI	Anytime	**Group 1:** Absence of major depression(MD) and no AD use **Group 2:** Absence of MD and using AD **Group 3:** Presence of MD and no AD use **Group 4:** MD & using Ads	Medication exposure assessed through RCT and PH-9 questionnaire used	Identification through RCT	1650	Adjusted for age, race, income, current smoking, depression severity and trial placed in for RCT	**PH-9<10 and unexposed =** 110/1359 **PH-9<10 and exposed =** 14/73 **PH-9>10 and unexposed =** 5/72 **PH-9>10 and exposed =** 2/16<br><br>**No AD use** = 118/1484 **Any AD use =** 16/90 **SSRI use** = 11/57 **Non-SSRI use**= 4/25 **Both use of SSRI and non-SSRI** = 1/8	**<u>Adjusted RR:</u>** **Ph-9<10 and unexposed =** reference **PH-9<10 and exposed =** 1.87 (1.18-2.99) **PH-9>10 and unexposed =** 0.85 (0.37-1.96) **PH-9>10 and exposed =** 1.4 (0.47-4.17)<br><br>**<u>specific medication types:</u>** **Any AD use** = 1.92 (1.22-3.02) **SSRI use** = 1.73 (1.0-3.0) **Non-SSRI use**= 3.45(1.99-5.98) **Both SSRI and non-SSRI** = 1.15 (0.19-7.06)
Richardson et al. (2019) [22]	Cohort	UK	Using UK collected teratogen surveillance data<br><br>Median: 31 for both groups	Venlafaxine (SNRI)	Anytime	**Exposed:** Individuals using venlafaxine at any point during pregnancy **Comparator group 1:** Unexposed to any AD medication (5:1) **Comparator group 2:** disease matched comparator of SSRI AD exposed	Surveillance data from UKTIS	Postal questionnaire sent after delivery date from healthcare professional who contacted UKTIS	2529	Adjusted for history of SA, tobacco exposure, alcohol, recreational drugs and folic acid	**Venlafaxine =** 46/281 **SSRI exposed =** 114/843 **Unexposed =** 140/1405	**Crude OR for unexposed =** 1.57 (1.06-2.30) **Crude OR for SSRI comparator =** 1.05 (0.7-1.57)<br><br>**Adjusted HR for unexposed =** 1.26 (0.829-1.91) **Adjusted HR for SSRI comparator =** 1.00 (0.655-1.53)
Wu et al. (2019) [23]	Prospective cohort	North Carolina, Tennessee and Texas	Enrolment into community-based cohort<br><br>NA	All AD – classified into SSRI and non-SSRI	First trimester	**Exposed:** reported AD use since pregnancy and before interview **Comparator group 1:** Never used AD	community-based cohort – AD use assessed during interview	Self-reported pregnancy outcomes- verified by medical records	5451	Adjustment for maternal age, BMI, caffeine consumption, smoking, alcohol, race, SA history	**Exposed to any AD =** 42/223 **Use of SSRIs =** 35/179 **Exposure to non-SSRIs =** 7/44 **Unexposed =** 617/5228	**Unadjusted HR:**<br><br>**Any AD =** 1.51 (1.10-2.06) **Only SSRIs =** 1.59 (1.13-2.23) **Only non-SSRIs = 1.21** (0.57-2.56)

												**Adjusted HR**<br>**Any AD use:** 1.34 (0.97-1.85)<br>**SSRI only:** 1.45 (1.02-2.06)<br>**Non-SSRI only:** 0.96 (0.45-2.04)
Bahat et al. (2020) [24]	Cohort – conference abstract only	Israel 2001-2015	Data utilised from Israeli TIS<br><br>NA	Duloxetine (SNRI)	First trimester	**Exposed:** Exposure to duloxetine during first trimester<br>**Comparator group 1:** women exposed to TCAs<br>**Comparator group 2:** non-teratogenic exposure	Information taken when contacted TIS	Identified through follow-up after delivery date	722	None	NA	**Adjusted HR =** 1.89 (1.04-3.43)
Kolding et al. (2021)[25]	Register based study	Denmark 2007-2014	Routinely collected data from five nationwide registries and databases<br><br>NA	All Ads	First trimester	**Exposed:** two or more redeemed prescriptions<br>**Comparator group 1:** Absence of redemptions for AD	Using redeemed prescriptions through linkage to Danish health services prescription database	Identified through Danish national patient registry	364012	None	**Exposed =** 134/3971<br>**Unexposed =** 10739/342842	NA
Ankarfeldt et al. (2021)[26]	Prospective cohort	Denmark 2004-2016	Study population used danish registry data whereby women were included if they had a recorded birth in birth register or an abortion in patient register between 2004-2016<br><br>NA	Duloxetine (SNRI)<br>SSRIs<br>Venlafaxine (SNRI)	Anytime during pregnancy	**Exposed** = redemption of AD prescription in window from 30 days prior to LMP to 140 days post LMP or end of pregnancy, whichever came first<br>**Comparator group 1:** Duloxetine non-exposed (no redemption of prescription during the window)<br>**Comparator group 2:** SSRI exposed<br>**Comparator group 3:** Venlafaxine exposed	Using the Danish national prescription register	Identified through either the birth registry for all births, or the patient register for abortions.	1020288	birth year, maternal age, psychiatric hospitalization, household income, education, comorbidities, co-medication	**Unadjusted:**<br>**Duloxetine =** 145/1212<br>**Duloxetine unexposed =** 106309/1018745<br>**SSRI =** 2900/28345<br>**Venlafaxine =** 497/4908	**Adjusted**<br><br>**Duloxetine vs unexposed** = 1.14 (0.96-1.34)<br>**Duloxetine vs SSRI =** 1.23 (1.04-1.46)<br>**Duloxetine vs venlafaxine =** 1.18 (0.98-1.42)<br><br>**PS-matched**<br><br>**duloxetine vs unexposed** = 1.08 (0.89-1.31)<br>**Duloxetine vs SSRI =** 1.25 (1.00-1.57)<br>**Duloxetine vs venlafaxine =** 1.08(0.82-1.41)

Ads=antidepressants, SSRIs = selective serotonin reuptake inhibitors, SNRIs= serotonin-norepinephrine reuptake inhibitors, TCAs= tricyclic antidepressants, NDRI = norepinephrine and dopamine reuptake inhibitors, NaSSA = noradrenergic and serotonergic specific antidepressants, RCT= randomized controlled trial, TIS=teratogen information service, NA= not available, MD = major depression

Kitchin et al. (2022)[27]	Nested case-control	Spain<br><br>2002-2015	Cohort included women of childbearing age (15-49) with at least 1 year of registration with their primary care practitioner between Jan 2002 and Dec 2015.	All ADs	Anytime during pregnancy	**nonusers:** when there were no prescriptions in any of these periods<br><br>**discontinuers:** women who had at least one prescription only during the prepregnancy period;<br><br>**continuers:** women who had at least one prescription during the prepregnancy period and also during the first trimester<br><br>**new users:** women who had at least one pre- scription only during the first trimester.	Spanish database BIFAP	Miscarriage identified via ICD codes in the BIFAP database	72,280	Number of GP visits, obesity, smoking, HTA and diabetes	**Discontinued AD use vs no use:** 2216/8276 vs 15428/62658, **Continued AD use vs no use:** 385/1205 vs 15428/62658, **Incident AD use vs no use:** 41/140 vs 15428/62658	**ORs - Discontinued AD use vs no use:** 1.05 (1.00-1.11) **Continued AD use vs no use:** 1.29 (1.13-1.46) **Incident AD use vs no use:** 1.12 (0.78-1.62)
Ostenfeld et al. (2022)[28]	Cohort	Denmark 1997-2016	Study population using Danish registry data – with the study population being all pregnancies registered in the medical birth register or national hospital register (between 1st jan 1997 to 31st Dec 2016)<br><br>NA	Mirtazapine (NaSSA)	Exposure earlier than 22 weeks of completed gestation	**Exposed:** One filled prescription of mirtazapine	Using the register for of medicinal product statistics.	Identified in the National Hospital Register of Denmark	9,500	Age at pregnancy onset, marital status, place of birth, region of residence, gross household income, education status, parity, multiple birth pregnancy, smoking during pregnancy, previous pregnancy with adverse fetal outcome, prescription drug use in past year and hospital care utilization in past year	**Mirtazepine exposed vs unexposed:** 237/1168	**HRs: Mirtazepine exposed vs unexposed:** 1.04 (0.91-1.20)

Giner-Soriano et al. (2022)[29]	Case-control	Spain	Pregnancy episodes of women aged 12-50 from SIDIAP database during 2011-2020<br><br>NA	NA	Exposure up to 120 days after pregnancy start date or at SA date	**Exposure:** dispensation of AD 30 days before to 120 days after pregnancy start date or at spontaneous abortion date, whichever occurs first	SIDIAP – contains primary care health data records	Primary healthcare records of the Catalan Health Institute	180,692	NA	NA	**ORs - antidepressants vs no antidepressants:** 1.11 (1.06-1.16)

References

1. Chambers CD, Johnson KA, Dick LM, Felix RJ, Jones KL. Birth outcomes in pregnant women taking fluoxetine. *N Engl J Med* 1996; **335**(14): 1010-5.
2. Kulin NA, Pastuszak A, Sage SR, et al. Pregnancy outcome following maternal use of the new selective serotonin reuptake inhibitors: a prospective controlled multicenter study. *Jama* 1998; **279**(8): 609-10.
3. Einarson A, Fatoye B, Sarkar M, et al. Pregnancy outcome following gestational exposure to venlafaxine: a multicenter prospective controlled study. *Am J Psychiatry* 2001; **158**(10): 1728-30.
4. Einarson A, Bonari L, Voyer-Lavigne S, et al. A multicentre prospective controlled study to determine the safety of trazodone and nefazodone use during pregnancy. *Canadian Journal of Psychiatry* 2003; **48**(2): 106-10.
5. Sivojelezova A, Shuhaiber S, Sarkissian L, Einarson A, Koren G. Citalopram use in pregnancy: Prospective comparative evaluation of pregnancy and fetal outcome. *American Journal of Obstetrics and Gynecology* 2005; **193**(6): 2004-9.
6. Chun-Fai-Chan B, Koren G, Fayez I, et al. Pregnancy outcome of women exposed to bupropion during pregnancy: a prospective comparative study. *Am J Obstet Gynecol* 2005; **192**(3): 932-6.
7. Djulus J, Koren G, Einarson TR, et al. Exposure to mirtazapine during pregnancy: A prospective, comparative study of birth outcomes. *J Clin Psychiatry* 2006; **67**(8): 1280-4.
8. Einarson A, Choi J, Einarson TR, Koren G. Rates of spontaneous and therapeutic abortions following use of antidepressants in pregnancy: results from a large prospective database. *J Obstet Gynaecol Can* 2009; **31**(5): 452-6.
9. Nakhai-Pour HR, Broy P, Berard A. Use of antidepressants during pregnancy and the risk of spontaneous abortion. *Cmaj* 2010; **182**(10): 1031-7.
10. Paulus W, Schloemp S, Stoz F. Paroxetine exposure during pregnancy: Increased risk of congenital malformations? *Archives of Gynecology and Obstetrics* 2010; **282**: S79.
11. Chan RL, Jonsson Funk ML, Savitz DA, Hartmann KE. Self-reported antidepressants use and spontaneous abortion. *American Journal of Epidemiology* 2011; **173**: S140.
12. Einarson A, Choi J, Koren G, Einarson T. Outcomes of infants exposed to multiple antidepressants during pregnancy: results of a cohort study. *J Popul Ther Clin Pharmacol* 2011; **18**(2): e390-6.
13. Ban L, Tata LJ, West J, Fiaschi L, Gibson JE. Live and non-live pregnancy outcomes among women with depression and anxiety: a population-based study. *PLoS ONE* 2012; **7**(8): e43462.
14. Klieger-Grossmann C, Weitzner B, Panchaud A, et al. Pregnancy outcomes following use of escitalopram: a prospective comparative cohort study. *J Clin Pharmacol* 2012; **52**(5): 766-70.

15. Kjaersgaard MI, Parner ET, Vestergaard M, et al. Prenatal antidepressant exposure and risk of spontaneous abortion - a population-based study. *PLoS ONE* 2013; **8**(8): e72095.

16. Andersen JT, Andersen NL, Horwitz H, Poulsen HE, Jimenez-Solem E. Exposure to selective serotonin reuptake inhibitors in early pregnancy and the risk of miscarriage. *Obstet Gynecol* 2014; **124**(4): 655-61.

17. Abadie D, Hurault-Delarue C, Damase-Michel C, Montastruc JL, Lacroix I. Medication exposure and spontaneous abortion: A case-control study using a French medical database. *Clinical and Experimental Obstetrics and Gynecology* 2015; **42**(4): 431-6.

18. Johansen RL, Mortensen LH, Andersen AM, Hansen AV, Strandberg-Larsen K. Maternal use of selective serotonin reuptake inhibitors and risk of miscarriage - assessing potential biases. *Paediatr Perinat Epidemiol* 2015; **29**(1): 72-81.

19. Te Winkel B, Beghin D, Pistelli A, et al. Venlafaxine exposure in pregnancy, a multicenter ENTIS study. *Pharmacoepidemiology and Drug Safety* 2016; **25 (Supplement 3)**: 325-6.

20. Almeida ND, Basso O, Abrahamowicz M, Gagnon R, Tamblyn R. Risk of Miscarriage in Women Receiving Antidepressants in Early Pregnancy, Correcting for Induced Abortions. *Epidemiology* 2016; **27**(4): 538-46.

21. Evans-Hoeker EA, Eisenberg E, Diamond MP, et al. Major depression, antidepressant use, and male and female fertility. *Fertility and Sterility* 2018; **109**(5): 879-87.

22. Richardson JL, Martin F, Dunstan H, et al. Pregnancy outcomes following maternal venlafaxine use: A prospective observational comparative cohort study. *Reprod Toxicol* 2019; **84**: 108-13.

23. Wu P, Velez Edwards DR, Gorrindo P, et al. Association between First Trimester Antidepressant Use and Risk of Spontaneous Abortion. *Pharmacotherapy* 2019; **39**(9): 889-98.

24. Bahat M, Shechtman S, Diav-Citrin O. Pregnancy outcome after first trimester exposure to Duloxetine. *Reprod Toxicol* 2020; **97**: 3.

25. Kolding L, Ehrenstein V, Pedersen L, et al. Antidepressant use in pregnancy and severe cardiac malformations: Danish register-based study. *Bjog* 2021; **128**(12): 1949-57.

26. Ankarfeldt MZ, Petersen J, Andersen JT, et al. Duloxetine Exposure During Pregnancy and the Risk of Spontaneous and Elective Abortion: A Danish Nationwide Observational Study. *Drugs Real World Outcomes* 2021; **8**(3): 289-99.

27. Kitchin Á, Huerta C, Llorente-García A, Martínez D, Ortega P, Cea-Soriano L. The role of prenatal exposure to antidepressants, anxiolytic, and hypnotics and its underlying illness on the risk of miscarriage using BIFAP database. Pharmacoepidemiology and Drug Safety. 2022 Aug;31(8):901-12.

28. Ostenfeld A, Petersen TS, Pedersen LH, Westergaard HB, Løkkegaard EC, Andersen JT. Mirtazapine exposure in pregnancy and fetal safety: A nationwide cohort study. Acta Psychiatrica Scandinavica. 2022 Jun;145(6):557-67.

29. Giner-Soriano M, Prat-Vallverdu O, Vazquez ML, Vilaplana-Carnerero C, Vedia C, Gomez-Lumbreras AG, Morros R. Spontaneous abortion and drug exposure. Case-control study with SIDIAP database. InPHARMACOEPIDEMIOLOGY AND DRUG SAFETY 2022 Sep 1 (Vol. 31, pp. 238-239). 111 RIVER ST, HOBOKEN 07030-5774, NJ USA: WILEY.
