# Supplementary material for: Association between antidepressant use during pregnancy and miscarriage: a systematic review and meta-analysis
Source: BMJ Open. 2024 Jan 25;14(1):e074600. doi: 10.1136/bmjopen-2023-074600 (PMC10824002; doi:10.1136/bmjopen-2023-074600)
Supplement: Supplementary data [file bmjopen-2023-074600supp006.pdf]

S4 Table. Risk of bias assessment

Study	Domain 1: Bias due to confounding	Domain 2: Bias in classification of intervention	Domain 3: Bias in selection of participants into study	Domain 4: Bias due to deviations from intended interventions	Domain 5: Bias due to missing data	Domain 6: Bias in measurement of the outcome	Domain 7: Bias in selection of the reported result	Overall RoB rating
Chambers et al (1996)	**Critical** No confounding adjustments made	**Critical**	**Critical**	**Critical**	**Critical**	**Critical**	**Critical**	**Critical**
Kulin et al (1998)	**Critical** No information on confounding adjustments	**Critical**	**Critical**	**Critical**	**Critical**	**Critical**	**Critical**	**Critical**
Einarson et al (2001)	**Serious** Justification: The paper only adjusted for maternal depression, therefore not adjusting for all three of the key confounding domains	**Serious** Justification: Exposure was determined through self-report of the individual. This was measured prospectively whereby the outcome had not occurred so would be unlikely to affect the exposure classification, however this was not verified through physician or healthcare records. Therefore, intervention status could not have been classified correctly and could be differential	**Serious:** Justification: The start of follow-up and intervention are not at the same point as women ringing the service enquiring about an exposure could have been taking it for weeks prior to call, which could induce bias as the intervention has differing effects over time, and no analytical techniques were put in place to minimise this bias	**Low** Justification: Study was not undergone in an experimental context	**Serious** Justification: Although complete case analysis was used, the number of exposed individuals and total sample was not stated. Therefore, there could have been missing data and there was no evidence to show that the missing data of outcome was not associated with the true value of the outcome	**Low** Justification: The outcome was measured through questionnaire to individual, alongside the results being verified by a physician. Although the outcome assessors could have been aware of exposure status, this is unlikely to have influenced outcome classification.	**Moderate** Justification: No statistical analysis plan was provided, however, all analysis that was stated in methods was completed and results given	**Serious**
Einarson et al (2003)	**Serious**	**Serious**	**Moderate**	**Low**	**Serious**	**Low**	**Moderate**	**Serious**

	Justification: Only indication for treatment and individual characteristics domain was adjusted for, meaning lifestyle exposures was not adjusted for resulting in serious risk of bias	Justification: Exposure was determined through self-report of the individual, however this was not verified through physician or healthcare records	Justification: Although start of intervention and follow-up were unlikely at the same time point, the study restricted to exposure during the first trimester only, therefore the differing effects of intervention across different time periods potentially inducing bias is minimised as restricted to first trimester	Justification: Study was not undergone in an experimental context	Justification: Although complete case analysis was utilised, the total sample of how many exposed individuals was not mentioned, only the number included in a complete case analysis. Therefore, there will likely be missing data, and as the outcome was determined through self-report there is no evidence that the missingness of the outcome was not related to its true value.	Justification: The outcome was determined through self-report questionnaire, however this was then confirmed through the individuals physician. Although the outcome assessors could have been aware of exposure status, this was unlikely to have influenced the reporting of the outcome	Justification: No statistical analysis plan was provided, however, all analysis that was stated in methods was completed and results given	
Sivojelezova et al (2005)	**Serious** Justification: Although presence of depression and maternal age was adjusted for, there was no factor that was adjusted for in lifestyle exposures. Therefore, if not all three of the confounding domains are	**Moderate** Justification: Self-report of exposure through a standardised intake form. In addition, letter sent to physician to confirm the information given by individual	**Serious** Justification: The start of intervention and follow-up were likely not at the same time point as women just rung up enquiring about exposure, in addition, no analysis methods were used to account for the differing effects of intervention across different time points	**Low** Justification: Study was not undergone in an experimental context	**Serious** Justification: Although complete case analysis was used, the number of exposed individuals and total sample was not stated. Therefore, there could have been missing data and there was no evidence to show that the missing data in the	**Low** Justification: The outcome was measured through a questionnaire which is not the most reliable, however, the questionnaire was also sent to healthcare professional to confirm. Therefore, the healthcare professional would likely know the exposure status of	**Moderate** Justification: No statistical analysis plan was given	**Serious**

	adjusted for it means serious rating				outcome was not associated with the the true value of the outcome.			
Chun-Fai-Chan et al (2005)	**Moderate** Justification: All three confounding domains of indication for treatment, lifestyle exposures and individual characteristics were adjusted for	**Moderate** Justification: Exposure was classified through self-report in questionnaire, however this was verified through a physician. Additionally, there was a follow-up questionnaire. As exposure was measured prospectively there was no risk of outcome influencing reporting of exposure	**Serious** Justification: The start of follow-up and intervention are not at the same point, which could induce bias as the intervention has differing effects over time, and no analytical techniques were put in place to minimise this bias	**Low** Justification: Study was not undergone in an experimental context	**Serious** Justification: Although complete case analysis was used, the number of exposed individuals and total sample was not stated. Therefore, there could have been missing data and there was no evidence to show that the missing data was not associated with the true value of the outcome. This is particularly relevant considering the outcome was individual self report.	**Low** Justification: outcome was measured through telephone conversation and then. Verified with physician. Although the outcome assessors could have been aware of exposure status	**Moderate** Justification: No statistical analysis plan was provided, however, all analysis that was stated in methods was completed and results given	**Serious**
Djulus et al (2006)	**Moderate** Justification: All three confounding factors of indication for treatment, individual characteristics	**Moderate** Justification: Women contacted the service regarding exposure and then details of exposure were taken including duration, timing	**Serious** Justification: The start of follow-up and intervention are not at the same point, which could induce bias as the intervention has differing effects over	**Low** Justification: Study was not undergone in an experimental context	**Serious** Justification: Complete case analysis was used but there was no total sample size or flow diagram indicating recruitment of	**Low** Justification: The outcome was measured through questionnaire to individual, alongside the results being verified by a physician. Although	**Moderate** Justification: No statistical analysis plan was provided, however, all analysis that was stated in methods was completed and results given	**Serious**

	and lifestyle exposures were adjusted for	and frequency - this questionnaire was then sent to practitioner for details to be confirmed	time, and no analytical techniques were put in place to minimise this bias		individuals and any exclusions. Missing data was not mentioned at all.	the outcome assessors could have been aware of exposure status, this is unlikely to have influenced outcome classification.		
Einarson et al (2009)	**Serious**<br>Justification: Although the domains of individual characteristics and lifestyle exposures were adjusted for through maternal age and smoking, indication for treatment domain was not adjusted for	**Serious**<br>Justification: Exposure was determined through self-report of the individual, however this was not verified through physician or healthcare records	**Moderate**<br>Justification: Although start of intervention and follow-up were unlikely at the same time point, the study restricted to exposure during the first trimester only, therefore the differing effects of intervention across different time periods potentially inducing bias is minimised as restricted to first trimester	**Low**<br>Justification: Study was not undergone in an experimental context	**Serious**<br>Justification: Although complete case analysis was utilised, the total sample of how many exposed individuals was not mentioned, only the number included in a complete case analysis. Therefore, there will likely be missing data, and as the outcome was determined through self-report there is no evidence that the missingness of the outcome was not related to its true value.	**Serious**<br>Justification: The outcome was reported by the mother/individual, however this was not verified with their physician or healthcare records. Therefore the exposure status known by the individual could influence their reporting of outcome classification.	**Moderate**<br>Justification: No statistical analysis plan was provided, however, all analysis that was stated in methods was completed and results given	**Serious**
Nakhai-Pour et al (2010)	**Serious**<br>Justification: It did not adjust for a factor in all three of the key confounding domains, as it did	**Moderate**<br>Justification: The exposure classification was done through GP records and therefore all	**Moderate**<br>Justification: Matching was employed, as well as controls selected independently to intervention status.	**Low**<br>Justification: Study was not undergone in an experimental context	**Low**<br>Justification: Smoking and maternal weight covariates were the only variables which had missing	**Low**<br>Justification: The outcome was measured through the Quebec pregnancy register and therefore very	**Serious**<br>Justification: No statistical analysis plan was given, additionally lots of data collected on covariates however	**Serious**

	not adjust for any factor of lifestyle exposure	information to classify intervention was recorded at start of intervention. Extent of non-differential misclassification would not be large.	The cases and controls were matched on gestational age so same risk period. Therefore, not at serious risk of bias in this domain		data. The missingness of these confounding factors is unlikely to be related to the true value of the outcome because the outcome was determined through pregnancy register rather than self-report	objective and not influenced by assessors	only some included in the adjustment model. Finally no results given for the sensitivity analysis.	
Paulus et al (2010)	**Critical** No information given on any confounding adjustments done	**Critical**	**Critical**	**Critical**	**Critical**	**Critical**	**Critical**	**Critical**
Chan et al (2011)	**Serious** Justification: Only individual characteristics and lifestyle exposure domains had a factor adjusted for in them - however indication for treatment was not adjusted for at all - therefore deemed serious as not all confounding domains satisfied.	**Serious** Justification: It was unclear the process of exposure classification, and indicated that only telephone interviews of self-report were used for exposure classification.	**Moderate** Justification: Start of intervention and follow-up were not the same, however, the analysis used was logistic time survival models so the differing effect of ads of different time periods was accounted for	**Low** Study was not undergone in an experimental context	**Serious** Justification: No information given on missing data.	**Low** Justification: Telephone interviews and medical records were used to determine the outcome, reliable method. Unclear whether outcome assessors were aware of exposure status, however unlikely to have influenced outcome determination because outcome is very objective	**Moderate** Justification: No statistical analysis plan was provided, however, all analysis that was stated in methods was completed and results given	**Serious**

Einarson et al (2011)	**Moderate** Justification: All three confounding domains were adjusted for	**Serious** Justification: Exposure was determined through self-report of the individual, however this was not verified through physician or healthcare records	**Serious** Justification: The start of follow-up and intervention are not at the same point as women ringing the service enquiring about an exposure could have been taking it for weeks prior to call, which could induce bias as the intervention has differing effects over time, and no analytical techniques were put in place to minimise this bias	**Low** Study was not undergone in an experimental context	**Serious** Justification: Although complete case analysis was used, the number of exposed individuals/total sample was not stated. Therefore, there could have been missing data on participants that would have been excluded - this missing data could include outcome data which could be related to its true value as the outcome was self-report.	**Low** Justification: The outcome was determined through self-report questionnaire; however this was then confirmed through the individuals physician. Although the outcome assessors could have been aware of exposure status	**Moderate** Justification: No statistical analysis plan was provided, however, all analysis that was stated in methods was completed and results given	**Serious**
Ban et al (2012)	**Moderate** Justification: All three confounding domains were adjusted for through some factor - including presence of depression, maternal age and smoking	**Moderate** Justification: Exposure was classified through prescription databases where all drug usage was recorded - however just small difficulty of unsure the true ingested drug usage	**Serious** Justification: Although prescription database was used, it is unlikely that the start of intervention and start of follow-up will be the same. Additionally, the authors did not use any statistical analysis including cox proportional regression to counteract	**Low** Study was not undergone in an experimental context	**Low** Justification: Missing data on covariates found, and the missingness would be unrelated to the outcome because they were taken from NHS databases before the outcome occurred. Additionally, no missing outcome data was found	**Low** Justification: Outcome was measured reliably through healthcare records, however unclear whether outcome assessors would know exposure status but unlikely this would have influenced classification	**Moderate** Justification: Although there was no statistical analysis plan there were no missing parts to the analysis.	**Serious**

Klieger-Grossman et al (2012)	**Moderate** Justification: All three confounding domains were adjusted for through some factor - including presence of depression, maternal age and smoking	**Moderate** Justification: Initial interview questioning exposure, then a follow-up interview to confirm answers given in original questioning. This was all done prospectively so no risk of outcome influencing exposure reporting	**Serious** Justification: The start of intervention and follow-up would not have been the same, therefore inducing potential bias, additionally no analytical techniques were put in place to account for this bias either through time dependent analysis or restricting the analysis to first trimester only	**Low** Study was not undergone in an experimental context	**Serious** Justification: Although complete case analysis was used, the number of exposed individuals and total sample was not stated. Therefore, there could have been missing data and there was no evidence to show that the missing data of outcome was not associated with the true value of it	**Low** Justification: Assessment of the outcome won't be influenced by the exposure	**Moderate** Justification: No statistical analysis plan was provided, however, all analysis that was stated in methods was completed and results given	**Serious**
Kjaesgaard et al (2013)	**Serious** Justification: only two of the three confounding domains were adjusted for - as the third domain of lifestyle exposures was not adjusted for	**Moderate** Justification: Prescription records were used to determine exposure, therefore likely to classify individuals correctly, however if there is misclassification this will likely be non-differential and attenuate towards the null	**Serious** Justification: Unlikely the start of follow-up and intervention were at the same time point. Additionally, no analysis was utilized to account foe the differing effect of intervention at different points during pregnancy	**Low** Study was not undergone in an experimental context	**Low** Justification: There was missing gestational age data, however this was replaced by the median of all non-missing values. Therefore covariate data is missing - as the outcome was determined through hospital registry it is unlikely that the true value of the outcome will influence the covariate	**Low** Justification: Outcome was assessed through codes on danish national hospital registry therefore very reliable method and outcome assessors unlikely aware of exposure status	**Moderate** Justification: No statistical analysis plan was provided, however, all analysis that was stated in methods was completed and results given	**Serious**

Andersen et al (2014)	**Serious**<br>Justification: No factor of lifestyle exposure was adjusted for in this analysis, only indication for treatment and individual characteristics was adjusted for	**Moderate**<br>Justification: exposure was classified through prescription databases therefore unlikely to be any issues with classification of exposure - however slight issues with true ingested drug usage.	**Moderate**<br>Justification: Prescription database was used meaning unclear whether start of intervention and start of follow-up were at the same time point. However, a cox proportional hazards regression was used which counteracts the differing effects that exposure will have at different time points during pregnancy.	**Low**<br>Study was not undergone in an experimental context	**Low**<br>Justification: Minimal missing data was found on the confounding factors used, additionally, outcome was measured through hospital records therefore unlikely that the true value of the outcome will be influencing the missingness. No missing outcome data.	**Moderate**<br>Justification: Outcome was measured in a very objective and reliable method through hospital records, unclear whether outcome assessors knew exposure status however this is unlikely to influence exposure status determination. However, the method of outcome assessment could have differed between interventions as individuals on antidepressants would be more likely to come into hospital as more contact with healthcare services compared to unexposed individuals.	**Moderate**<br>Justification: No statistical plan was given, however all results that were mentioned in method were given	**Serious**
Abadie et al (2015)	**Serious**<br>Justification: Only one of the three confounding domains had anything adjusted for - only maternal age had been accounted for -	**Moderate**<br>Justification: The CRPV was notified by healthcare professional regarding exposure, then a questionnaire was sent across and confirmation of	**Serious**<br>Justification: The authors did employ matching, however the controls were not independently selected of the intervention status. Additionally, they didn't match on	**Low**<br>Study was not undergone in an experimental context	**Low**<br>Justification: There was missing data on the confounding factors, as the outcome was measured through a database it is unlikely the missingness of	**Low**<br>Justification: The outcome was measured through database administrative records, therefore very reliable	**Moderate**<br>Justification: Although there was no statistical analysis plan there were no missing parts to the analysis.	**Serious**

	therefore not all three confounding domains satisfied and serious risk of bias	current medication taken currently, so low risk of differential misclassification.	gestational age so time at risk would have differed between cases and controls		covariates is related to true value of outcome. However, no missing data for the outcome was found.			
Johansen et al (2015)	**Serious** Justification: Although this paper did a large amount of adjustments, especially in relation to confounding by indication, which most other papers didn't, it still didn't adjust for all three confounding domains as it missed adjusting for factors on lifestyle exposure	**Moderate** Justification: Both prescription records and self-report was used to assess exposure classification for individuals, therefore very reliable method using both - it did find differences between the two methods so likely there will be misclassification however very minimal	**Moderate** Justification: It is unlikely the start of intervention and start of follow-up were at the same point, however a cox proportional hazards regression was used to account for the differing effects of exposure at different time points during pregnancy	**Low** Study was not undergone in an experimental context	**Low** Justification: A flow diagram illustrating the total sample and any excluded individuals or missing data. This highlighted there was missing data in the exposure, covariates and outcome. However, as the outcome was assessed through healthcare registers and bypassing the mother its unlikely the true value of the outcome would cause this missingness.	**Low** Justification: The outcome was assessed through healthcare registers and the outcome assessors would be unaware of exposure status therefore very little risk regarding exposure influencing outcome status, especially considering the objective nature of the outcome	**Serious** Justification: No statistical analysis plan was given, and information on confounders was taken which were not used in the adjustment	**Serious**
Te winkel et al (2016)	**Critical** No information given on any confounding adjustments done	**Critical**	**Critical**	**Critical**	**Critical**	**Critical**	**Critical**	**Critical**
Almeida et al (2016)	**Serious** Justification: Only indication for	**Moderate** Justification: exposure was	**Serious** Justification: Although	**Low** Study wasn't undergone in an	**Low** Justification: There was missing data	**Low** Justification: Outcome was	**Moderate** Justification: No statistical plan was	**Serious**

	treatment and individual characteristics domain had been adjusted for, whereas no factors were adjusted for in lifestyle exposures	classified through prescription database, therefore highly reliable. However, there may be some discrepancies as to the actual ingested drug usage.	prescription database was used, it is unlikely that the start of intervention and start of follow-up will be the same. Additionally, the authors did not use any statistical analysis including cox proportional regression to counteract the differing effects that exposure will have at different time points during pregnancy.	experimental context	on the confounding factors, as the outcome was measured through the physician rather than self-report it is unlikely the true value of the outcome would be influencing the missingness of these covariates . However, no missing data for the outcome was found.	assessed through ICD-9 codes of physician notes, therefore very objective. Unclear whether outcome assessors know exposure status, however this is unlikely to impact the outcome classification	given, however all results that were mentioned in method were given	
Evans-Hoeker et al (2018)	**Moderate** Justification: All three confounding domains were adjusted for	**Moderate** Justification: The medication exposure was assessed throughout a previous RCT, with regulators of the trial ensuring accurate medication exposure classification	**Serious** Justification: It is unlikely the start of intervention and follow-up were at the same time point, additionally no analysis was utilised to account for the differing effect of intervention at differing points during pregnancy	**Low** Study wasn't undergone in an experimental context	**Moderate** Justification: 100% of individuals were included from the trial into the study. Less than 10% of PHQ scores were missing, therefore missing covariate data, no evidence that this missing data was not driven by the true value of the outcome.	**Low** Justification: The outcome was assessed throughout the RCT through testing and so a very thorough and reliable method. Unlikely that outcome assessors knowing the exposure would influence their classification of it	**Moderate** Justification: No statistical analysis plan was provided, however, all analysis that was stated in methods was completed and results given	**Serious**
Richardson et al (2019)	**Moderate** Justification: All three confounding domains had a factor that had been adjusted for	**Moderate** Justification: Exposure classification measured prospectively and by a healthcare	**Moderate** Justification: The start of intervention and follow-up were likely not at the same time point as individual rung up	**Low** Study wasn't undergone in an experimental context	**Low** Justification: They don't have complete data as missing data was reported in only the confounding	**Low** Justification: The outcome is a very objective measure. The outcome was measured through clinician self-report,	**Moderate** Justification: Although there was no statistical analysis plan there were no missing parts to the analysis.	**Moderate**

	including presence of maternal depression, maternal age and alcohol	professional over the phone. Classification of intervention status would not be influenced by the outcome	not necessarily at start of exposure. However, analysis used was time dependent cox models therefore the differing effect of intervention across different time periods was accounted for.		variables, however the incomplete data for these covariates is unlikely to have been influenced by the true value of the outcome because this was determined through clinician rather than self report.	and although exposure would have been known to them, this is unlikely to have influenced the outcome reporting.		
Wu et al (2019)	**Serious** Justification: Only individual characteristics and lifestyle exposure domains had a factor adjusted for in them - however indication for treatment was not adjusted for at all - therefore deemed serious as not all confounding domains satisfied.	**Serious** Justification: The exposure was classified at the end of first trimester interview - so a time point after the start of follow up. Additionally in a lot of the cases the exposure was recorded after the SA had already happened. Potentially differential misclassification between exposure and outcome individuals	**Moderate** Start of intervention and follow-up did not coincide, however a cox proportional hazard survival model was utilised to counteract the differing effects that exposure will have at different time points during pregnancy	**Low** Study wasn't undergone in an experimental context	**Serious** Justification: A flow diagram and tables with the details of missing data was illustrated. Individuals were excluded if they had missing data from the interview, this results in missing outcome data that could have been a result of the true value of the outcome.	**Low** Justification: The outcome was measured through a questionnaire, this was then confirmed through medical records. Although outcome assessors could have been aware of the exposure status it is unlikely assessment of outcome would have been influenced by this.	**Moderate** Justification: No statistical analysis plan was given	**Serious**
Bahat et al (2020)	**Serious** Justification: Unclear what they adjusted for	**No information**	**Moderate** Justification: It was unclear whether the start of intervention	**Low** Study wasn't undergone in an	**No information**	**No information**	**Serious** Justification: No statistical analysis plan was given,	**Serious**

	in this analysis and was not stated, however an adjusted estimate was given so some level of confounding was accounted for		and follow-up were at the same point, however, the analysis was restricted to first trimester where there is thought to be similar effects of Ads across first trimester so accounting for this	experimental context			additionally not all of the analysis that said was going to be conducted was released as results	
Kolding et al (2021)	**Critical** No confounding adjustment done	**Critical**	**Critical**	**Critical**	**Critical**	**Critical**	**Critical**	**Critical**
Ankarfeldt et al (2021)	**Moderate** Justification: All three confounding domains of indication for treatment, lifestyle exposures and individual characteristics had one factor adjusted for	**Moderate** Justification: Exposure was classified through prescription databases therefore likely to be reliable, however may not reflect true ingested drug usage.	**Moderate** Justification: Start of intervention and follow-up were likely not at same time point. However, a cox proportional hazard survival model was utilised to counteract the differing effects that exposure will have at different time points during pregnancy	**Low** Justification: Study wasn't undergone in an experimental context	**Low:** Justification: There was a small number of missing values for two covariates, however as this was not influenced by the value of the outcome and the missing values were imputed it poses minimal risk of bias.	**Low** Justification: The outcome was measured through patient registers, therefore very objective and reliable.	**Moderate** Justification: No statistical analysis plan was given but all of the analysis planned and stated in methods was conducted and given.	**Moderate**
Kitchin et al. (2022)	**Moderate** Justification: All three confounding domains of indication for treatment, lifestyle exposures and	**Moderate** Justification: Exposure was classified through prescription databases therefore likely to be reliable, however may not	**Serious** Justification: Although prescription database was used, it is unlikely that the start of intervention and start of follow-up will be the same.	**Low** Study wasn't undergone in an experimental context	**Low:** Justification: The only missing values were the LMP values which were imputed through subtracting 74 days off recorded entry date, therefore	**Low** Justification: Outcome was determined through ICD codes in BIFAP database, therefore very objective and reliable.	**Moderate** Justification: No statistical analysis plan was given but all of the analysis planned and stated in methods was conducted and given.	**Serious**

	individual characteristics had one factor adjusted for	reflect true ingested drug usage.	Additionally, the authors did not use any statistical analysis including cox proportional regression to counteract		poses minimal risk of bias.			
Ostenfeld et al. (2022)	**Serious** Justification: Only individual characteristics and lifestyle exposure domains had a factor adjusted for in them - however indication for treatment was not adjusted for at all - therefore deemed serious as not all confounding domains satisfied	**Moderate** Justification: Exposure was classified through prescription databases therefore likely to be reliable, however may not reflect true ingested drug usage.	**Moderate** Justification: It was unclear whether the start of intervention and follow-up were at the same point, however, the analysis did take into account various timepoints within exposure periods.	**Low** Study wasn't undergone in an experimental context	**Moderate** Justification: Not all individuals in the cohort were included in the analysis due to missing/implausible gestation values; these missing values could be associated with the outcome. Additionally, there were other missing values that were imputed using the mode value.	**Low** Justification: Outcome identified in national hospital register, therefore very objective and reliable	**Moderate** Justification: No statistical analysis plan was given but all of the analysis planned and stated in methods was conducted and given.	**Serious**
Giner-Soriano et al. (2022)	**Serious** Justification: Only individual characteristics and lifestyle exposure domains had a factor adjusted for in them - however indication for treatment was not adjusted for at all - therefore	**Moderate** Justification: Exposure was classified through prescription databases therefore likely to be reliable, however may not reflect true ingested drug usage.	**Serious** Justification: Although prescription database was used, it is unlikely that the start of intervention and start of follow-up will be the same. Additionally, the authors did not use any statistical analysis including cox proportional	**Low** Study wasn't undergone in an experimental context	**No information**	**Low** Justification: Outcome identified in primary health care records of Catalan institute, therefore very objective and reliable	**Moderate** Justification: No statistical analysis plan was given but all of the analysis planned and stated in methods was conducted and given.	**Serious**

	deemed serious as not all confounding domains satisfied		regression to counteract					
