# Supplementary material for: Association between antidepressant use during pregnancy and miscarriage: a systematic review and meta-analysis
Source: BMJ Open. 2024 Jan 25;14(1):e074600. doi: 10.1136/bmjopen-2023-074600 (PMC10824002; doi:10.1136/bmjopen-2023-074600)
Supplement: Supplementary data [file bmjopen-2023-074600supp007.pdf]

S5 Table. Confounding adjustments completed

Study	Confounding domain												
	Indication for treatment		Individual characteristics								Lifestyle exposures		
	Presence of depression	Severity of depression	History of SA	Maternal age	Gestational age	Polypharmacy	SEP	Ethnicity	BMI	Comorbidities	Smoking	Alcohol	Recreational drugs
Chambers et al (1996)													
Kulin et al (1998)													
Einarson et al (2001)	■												
Einarson et al (2003)	■				■								
Sivojelezova et al (2005)	■			■	■								
Chun-Fai-Chan et al (2005)	■			■	■						■	■	
Djulus et al (2006)	■			■						■	■	■	
Einarson et al (2009)			■	■	■						■	■	
Nakhai-Pour et al (2010)	■	■	■	■	■		■			■			
Paulus et al (2010)													
Chan et al (2011)				■			■	■	■		■	■	
Einarson et al (2011)	■			■	■						■	■	
Ban et al (2012)	■			■			■		■		■		
Klieger-Grossman et al (2012)	■			■	■						■	■	
Kjaesgaard et al (2013)	■			■			■			■			
Andersen et al (2014)	■		■	■		■	■						
Abadie et al (2015)				■									
Johansen et al (2015)	■	■	■	■	■		■						
Tw Winkel et al (2016)													
Almeida et al (2016)	■			■		■	■			■			
Evans-Hoeker et al (2018)	■	■		■			■	■			■		
Richardson et al (2019)	■		■	■	■						■	■	■
Wu et al (2019)			■	■								■	

**Bahat et al (2020)**													
**Kolding et al (2021)**													
**Ankarfeldt et al (2021)**													
**Kitchin et al. (2022)**													
**Ostenfeld et al. (2022)**													
**Giner-Soriano et al. (2022)**													

Green highlighted box indicating factor adjusted for and blank box indicating factor was not adjusted for
