# Supplementary figures and images for: Association between antidepressant use during pregnancy and miscarriage: a systematic review and meta-analysis
Source: BMJ Open. 2024 Jan 25;14(1):e074600. doi: 10.1136/bmjopen-2023-074600 (PMC10824002; doi:10.1136/bmjopen-2023-074600)

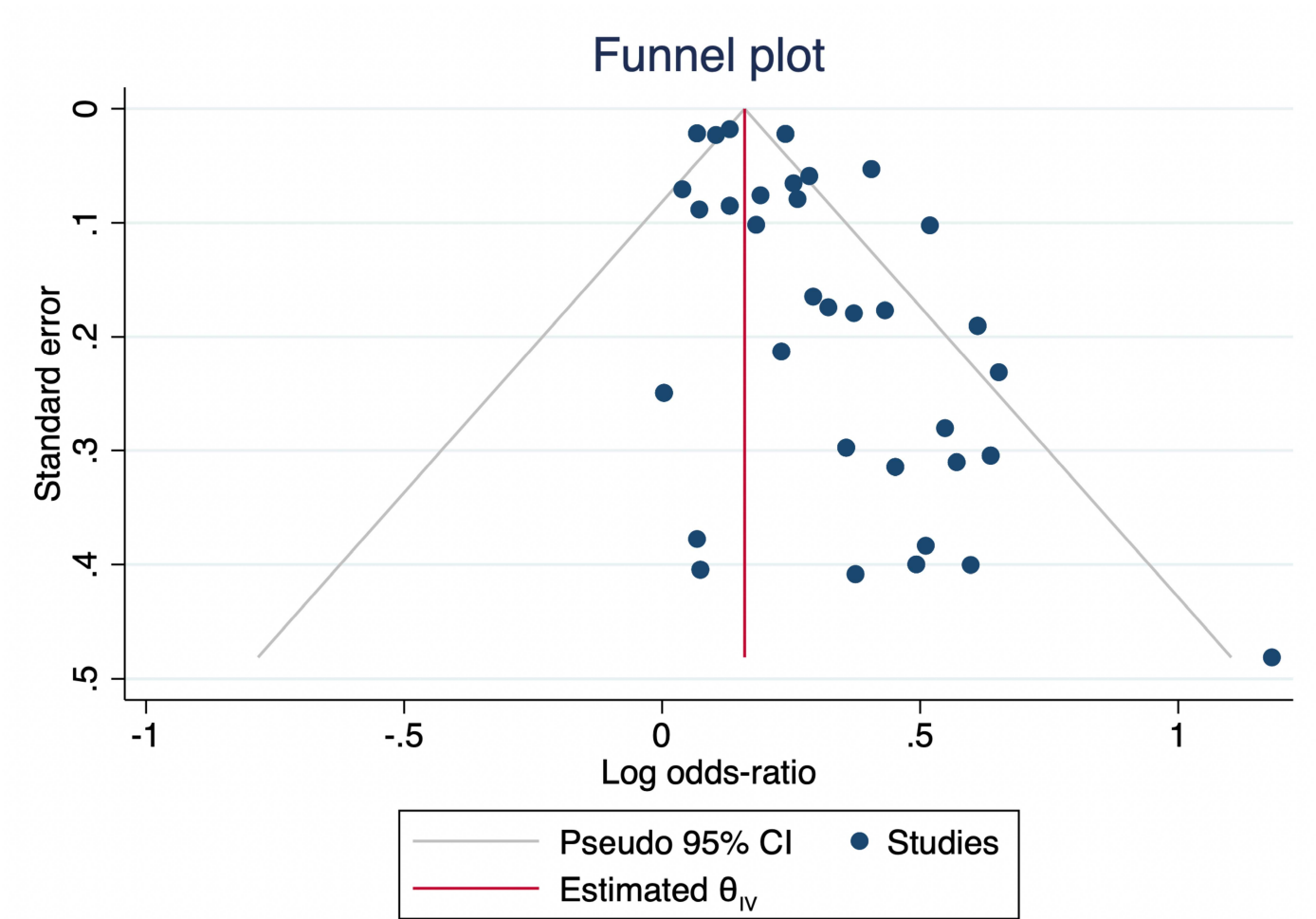

S2 Figure. Funnel plot

Supplement: Supplementary data [file bmjopen-2023-074600supp003.pdf]
