# Supplementary material for: Association between antidepressant use during pregnancy and miscarriage: a systematic review and meta-analysis
Source: BMJ Open. 2024 Jan 25;14(1):e074600. doi: 10.1136/bmjopen-2023-074600 (PMC10824002; doi:10.1136/bmjopen-2023-074600)
Supplement: Supplementary data [file bmjopen-2023-074600supp008.pdf]

**S6 Table. Meta-analysis results**

| Analysis type | | Number of studies | Number of adjusted studies used | Summary effect estimate (95% confidence interval) | Heterogeneity |
|---|---|---|---|---|---|
| **Main analysis** | | | | | |
| **Studies in the general population** | AD use vs unexposed comparator | 29 | 18 | 1.24 (1.18-1.31) | 69.2% |
| | SSRI vs unexposed comparator | 12 | 6 | 1.29 (1.15-1.44) | 81.3% |
| | SNRI vs unexposed comparator | 5 | 2 | 1.42 (1.11 – 1.81) | 46.9% |
| **Studies restricting population to women with depression** | AD use vs unmedicated depressed comparator | 6 | 4 | 1.16 (1.04 – 1.31) | 58.6% |
| | SSRI v another AD | 5 | 2 | 1.03 (0.93-1.15) | 0% |
| | SNRI vs another AD | 3 | 2 | 1.19 (1.02 – 1.39) | 0% |
| **Sensitivity analysis restricting to studies of sufficient quality** | | | | | |
| **Studies in the general population** | AD use vs unexposed comparator | 24 | 18 | 1.24 (1.17-1.31) | 72.10% |
| | SSRI vs unexposed comparator | 9 | 6 | 1.30 (1.15-1.46) | 86.0% |
| | SNRI vs unexposed comparator | 3 | 2 | 1.17 (1.01-1.36) | 0% |
| **Sensitivity analysis restricting to studies utilising prescription database as method of exposure classification** | | | | | |
| **Studies in the general population** | AD use vs unexposed comparator | 11 | 10 | 1.19 (1.13-1.26) | 83.8% |
| | SSRI use vs unexposed comparator | 4 | 4 | 1.25 (1.09-1.43) | 94.2% |
| | SNRI use vs unexposed comparator | 1 | 1 | 1.14 (0.96-1.35) | NA |
