# Supplementary material for: Association between antidepressant use during pregnancy and miscarriage: a systematic review and meta-analysis
Source: BMJ Open. 2024 Jan 25;14(1):e074600. doi: 10.1136/bmjopen-2023-074600 (PMC10824002; doi:10.1136/bmjopen-2023-074600)
Supplement: Author's manuscript [file bmjopen-2023-074600.draft_revisions.pdf]

BMJ Open

BMJ Open is committed to open peer review. As part of this commitment we make the peer review history of every article we publish publicly available.

When an article is published we post the peer reviewers' comments and the authors' responses online. We also post the versions of the paper that were used during peer review. These are the versions that the peer review comments apply to.

The versions of the paper that follow are the versions that were submitted during the peer review process. They are not the versions of record or the final published versions. They should not be cited or distributed as the published version of this manuscript.

BMJ Open is an open access journal and the full, final, typeset and author-corrected version of record of the manuscript is available on our site with no access controls, subscription charges or pay-per-view fees (http://bmjopen.bmj.com).

If you have any questions on BMJ Open's open peer review process please email info.bmjopen@bmj.com

BMJ Open

The association between antidepressant use during pregnancy and miscarriage: A systematic review and meta-analysis

Journal:	*BMJ Open*
Manuscript ID	bmjopen-2023-074600
Article Type:	Original research
Date Submitted by the Author:	23-Apr-2023
Complete List of Authors:	Smith, Sophie; University of Bristol Medical School,<br>Martin, Flo; University of Bristol Medical School<br>Rai, Dheeraj; University of Bristol, Centre for Academic Mental Health, School of Social & Community Medicine<br>Forbes, Harriet; University of Bristol,
Keywords:	Depression & mood disorders < PSYCHIATRY, EPIDEMIOLOGY, Maternal medicine < OBSTETRICS

SCHOLARONE™
Manuscripts

I, the Submitting Author has the right to grant and does grant on behalf of all authors of the Work (as defined in the below author licence), an exclusive licence and/or a non-exclusive licence for contributions from authors who are: i) UK Crown employees; ii) where BMJ has agreed a CC-BY licence shall apply, and/or iii) in accordance with the terms applicable for US Federal Government officers or employees acting as part of their official duties; on a worldwide, perpetual, irrevocable, royalty-free basis to BMJ Publishing Group Ltd ("BMJ") its licensees and where the relevant Journal is co-owned by BMJ to the co-owners of the Journal, to publish the Work in this journal and any other BMJ products and to exploit all rights, as set out in our licence.

The Submitting Author accepts and understands that any supply made under these terms is made by BMJ to the Submitting Author unless you are acting as an employee on behalf of your employer or a postgraduate student of an affiliated institution which is paying any applicable article publishing charge ("APC") for Open Access articles. Where the Submitting Author wishes to make the Work available on an Open Access basis (and intends to pay the relevant APC), the terms of reuse of such Open Access shall be governed by a Creative Commons licence – details of these licences and which Creative Commons licence will apply to this Work are set out in our licence referred to above.

Other than as permitted in any relevant BMJ Author's Self Archiving Policies, I confirm this Work has not been accepted for publication elsewhere, is not being considered for publication elsewhere and does not duplicate material already published. I confirm all authors consent to publication of this Work and authorise the granting of this licence.

For peer review only

The association between antidepressant use during pregnancy and miscarriage: A systematic review and meta-analysis

Centre for Academic Mental Health, University of Bristol, Bristol (Sophie Smith MSc)
Centre for Academic Mental Health, University of Bristol, Bristol (Florence Martin MSc)
Centre for Academic Mental Health, University of Bristol, Bristol (Dr Dheeraj Rai MRCPsychPHD)
Centre for Academic Mental Health, University of Bristol, Bristol (Dr Harriet Forbes PhD)

Correspondence to Sophie Smith, Centre for Academic Mental Health, University of Bristol, Bristol, BS8 2BN
Sophie.smith@bristol.ac.uk

Word count: 3643

For peer review only

Abstract

Background

Literature surrounding the association between antidepressant use during pregnancy and miscarriage is conflicting.

Aims

The aim of this study is to synthesise the literature and provide the most up-to-date evidence to help women and physicians make informed decisions.

Methods

We searched MEDLINE, EMBASE and PsychINFO up to 21st January 2022. Case-control, cohort and cross-sectional study designs were selected if they compared individuals exposed to any antidepressant class during pregnancy to comparator groups of either no antidepressant use or an alternate antidepressant. Effect estimates were extracted from selected studies and pooled using a random-effects meta-analysis. Risk of bias (RoB) was assessed using the ROBINS-I tool, and heterogeneity was assessed using the I^2 statistic. Subgroup analyses were utilised to explore antidepressant classes and the impact of confounding by indication. The protocol is registered in PROSPERO CRD42021262839.

Results

1800 records were identified from the search, of which 26 were included in the systematic review and meta-analysis. Antidepressant users initially appeared to have a higher risk of miscarriage compared to unexposed individuals from the general population (summary effect estimate: 1.29, 95% CI: 1.21-1.38, I^2 = 70.8%; number of studies (n)=26). However, the summary estimate decreased when comparing against unexposed individuals with maternal depression (1.16, 0.98-1.36; I^2 = 67.7%; n=5). 19 studies suffered from serious RoB, and two studies had moderate RoB.

For peer review only

Conclusion

After accounting for maternal depression there is little evidence of any association between antidepressant use during pregnancy and miscarriage. Instead, the results indicate the biasing impact of confounding by indication.

Key words: Depression; antidepressant use; miscarriage; spontaneous abortion; confounding by indication

Strengths and limitations of the study:

- Confounding by indication and depression severity was a key part of the analysis and results, elucidating the true independent effect of antidepressant use during pregnancy. As this important bias has not previously been addressed in any other review in the field, it emphasises the requirement for accounting for psychiatric illness in future reviews and studies to avoid biased estimates.
- To the best of our knowledge, our study was also the first review in this field to assess RoB using the ROBINS-I tool. This tool gave a significant advantage over the commonly used Newcastle-Ottawa scale and highlighted the need for more thorough confounding adjustment and reporting of missing data in future studies.
- This review utilised a large amount of studies, identified through an extremely thorough search strategy, thus enhancing statistical power and insight over previous reviews.
- High heterogeneity existed between papers, likely due to the included studies investigating different research questions, implementing different methodologies and populations, timing of exposure and confounding adjustment, thereby reducing the quality of the meta-analysis.
- The studies included could suffer from publication bias as few studies were distributed around the null line and most found associations that transcended the null (Supplementary Figure S1). Additionally, small studies producing inverse relationships are absent from the field. This conclusion was supported by the small p-value produced in the Egger test. If only positive results are being published this could result in exaggerated review results. However, these conclusions may not be

For peer review only

driven by publication bias, but instead either real causal associations or lack of methodological rigour throughout the studies.

Introduction

Depression affects around 264 million people worldwide and is one of the top three leading causes of years lived with disability globally.(1) This high prevalence is also seen among pregnant women, with 15-23% suffering from a depressive disorder.(2) Around 3-8% of women are prescribed antidepressants during their pregnancy in Europe.(3) This figure appears to be on an upwards trajectory since the 1990s,(4) potentially driven by both increasing prenatal depression rates,(5) and a shortage of non-pharmacological treatment options.(6)

Miscarriage, also referred to as spontaneous abortion, is defined as pregnancy loss before viability, however, the gestational threshold of viability varies between countries, from 20-28 weeks.(7) In the UK, viability is determined as up to 24 weeks.(7) Depending on the definition, miscarriage risk varies; on average it occurs in approximately 15% of pregnancies.(7) Several studies and reviews found an increased miscarriage risk in women using antidepressants during pregnancy, compared to unexposed individuals.(8-10) Antidepressants can cross the placental barrier through passive diffusion,(11) highlighting biological plausibility. Selective serotonin reuptake inhibitors (SSRIs) and serotonin-norepinephrine reuptake inhibitors (SNRIs) are the most frequently used pharmacotherapy option for depression,(12) both of which increase serotonin levels in the synaptic cleft.(3, 13) Serotonergic systems are involved in embryonic developmental processes including neural crest cell differentiation,(13) a process producing cells and tissue types.(14) This highlights the importance of serotonin in embryogenesis thus its potential influence on miscarriage. Alternatively, untreated maternal depression has been shown to be associated with miscarriage(15). This is an issue in observational studies known as confounding by indication, whereby maternal depression can independently affect the outcome of miscarriage. This could be through a number of mechanisms and mediators including lifestyle factors,(16) or stress and inflammation.(15) Including pregnant women in

For peer review only

randomised controlled trials (RCTs) is unethical, thus all evidence is observational and therefore susceptible to bias, making causal inference challenging. This is highlighted by studies whose observed associations between antidepressant use during pregnancy and miscarriage attenuated after stratifying for maternal depression.(17, 18)

Existing reviews examining this relationship investigated several perinatal outcomes and produced differing conclusions.(19-21) These reviews have some methodological limitations including incomplete search strategies,(19) do not include more recent studies from the last 10 years,(20, 21) and did not fully address confounding by indication,(19-21) a crucial element in isolating the true effect of antidepressants. This issue was addressed in the current review through utilising a depressed comparator, to allow for increased comparability between exposed and unexposed individuals.

The methodological limitations that exist in previous reviews means it remains unclear whether a causal relationship exists. Therefore, clear evidence and guidance around the association between antidepressant use during pregnancy and miscarriage is important as it will allow depressed pregnant women to make well-informed decisions and reduce their decisional conflict.(22) Poor perinatal mental health is becoming an ever-growing issue, up-to-date studies are needed now which investigate child outcomes in depressed women to help drive screening, intervention and prevention strategies.

Our aim was to conduct an updated and thorough systematic review and meta-analysis regarding the association between antidepressant use during pregnancy and miscarriage.

Methods

Search strategy and selection criteria

We searched the databases, MEDLINE, EMBASE and PsychINFO from database inception to 8th March 2021 using a comprehensive search strategy (Supplementary Table S1). In brief the following search terms were used: (antidepress* OR [generic/branded antidepressant name) AND (spontaneous abortion OR miscarriage). The search was updated on 4th August

For peer review only - http://bmjopen.bmj.com/site/about/guidelines.xhtml

2021 prior to commencement of data analysis, and a final search before finishing the review was conducted on 21st January 2022. The outcome of stillbirth was also included in our search strategy as these two outcomes are frequently reported together. However, stillbirth was not included in this review due to a higher number of relevant studies found than expected, ensuring literature was investigated at a thorough level. We placed no restrictions on language or publication status. Additionally, grey literature sources were searched. Abstracts in a language other than English were translated using a translation software, and full texts were assessed using a translator.

Studies were included if the study population included pregnant women, the exposure was antidepressants, and the outcomes included miscarriage. Any antidepressant class was eligible, and no restriction was placed on timing, dose, or duration of use. The comparator groups included pregnant women prescribed a different antidepressant to the primary specified type, or women with no antidepressant exposure. Studies investigating polypharmacy involving medication other than antidepressants were excluded, however, studies investigating more than one antidepressant class were included. Observational studies included utilised a cohort, case-control, or cross-sectional design. RCTs were included if they fit the eligibility criteria. Reviews, meta-analyses, ecological studies, case reports and case series were excluded.

The papers identified from the search strategy had their title and abstract screened independently by two reviewers in parallel (FM and HF). Outputs of the review were cross-checked, and discrepancies discussed with a third reviewer (SS). The full text of papers that passed title and abstract screening were obtained through Endnote, and three researchers completed a full-text review of a proportion of papers (SS, FM and HF). Final decisions and discrepancies were discussed within the whole team, including a consultant psychiatrist (DR).

Data analysis

A standardised template was used for data extraction. A PECOS framework was utilised and information on five domains of population, exposure, comparator, outcomes, and study characteristics was obtained. Variables of study design, country of study, study period, data

For peer review only

sampling methods, type and timing of antidepressant exposure, definition of exposure and comparator groups, identification of exposure and outcome, sample size, confounding adjustment, raw data and adjusted estimates (odds ratio (OR), risk ratio (RR), hazard ratio (HR)) including confidence intervals (CIs) were extracted. If more than one comparator group was utilised, the raw data and adjusted estimates for each comparison were extracted. Data on any secondary outcomes were not extracted. Data extraction was conducted independently by two reviewers for all papers, outputs were cross-checked (SS and FM), and discrepancies were discussed with a third reviewer (HF).

Studies were assessed using the ROBINS-I tool.(23) This tool evaluates non-randomised studies estimating intervention effects. Seven domains were assessed, including issues arising prior to and after intervention. The ROBINS-I tool is designed for cohort studies, therefore a template alteration specific to case-control studies was sought from the author of the ROBINS-I tool to allow for continuity in risk of bias (RoB) assessment across all study designs. RoB was independently assessed by two reviewers in parallel for all studies (SS and FM). The scoring and justification were compared, with any discrepancies discussed with a third reviewer (HF).

Meta-analyses were performed using STATA (version 16.0). Results were presented by comparator group used, specifically general population unexposed, depressed unexposed and depressed alternative antidepressant exposure. Additionally, subgroup analysis on SSRIs and SNRIs were conducted. Adjusted effect estimates were the preferential estimate when pooling results. HR, RR and OR were treated as equivalent due to the rare nature of miscarriages.(24) If studies gave estimates for multiple exposure groups including differing trimester estimates, these were pooled into one result using a meta-analysis. When adjusted estimates were not provided, ORs and standard errors were produced from raw data. Analyses were conducted using a random-effects model, due to the high likelihood of heterogeneity expected. Heterogeneity between studies was assessed using the I^2 statistic.(25) Additionally, a funnel plot and Egger test were conducted to assess evidence for publication bias.(26) A sensitivity analysis was conducted whereby papers with an overall critical RoB, or no information as identified through ROBINS-I tool were excluded.

For peer review only

The protocol is registered in PROSPERO CRD42021262839, available from https://www.crd.york.ac.uk/prospero/display_record.php?ID=CRD42021262839

Patient and Public Involvement

One of the authors (DR) is a clinical psychiatrist with extensive experience in the perinatal mental health space and had input into the analysis of results. Additionally, our research group has strong links with the perinatal mental health charity, Mothers For Mothers. This charity will play a part in our dissemination strategy.

Role of the funding source

The funders of the study, National Institute for Health Research and The Wellcome Trust, had no role in study design, data collection, data analysis, data interpretation, or writing of the report.

Results

Search results and characteristics of included studies

The initial and final search retrieved 2010 citations. After duplicates were removed, 1800 records had title and abstract screening, resulting in a further 1751 records being excluded. The full text of 49 articles were assessed for eligibility. Articles were excluded due to reasons including incorrect study design, insufficient data or if only stillbirth was investigated (Supplementary Table S2). This resulted in 25 studies being excluded, and 24 included, with the final searches adding a further two papers to the SR and meta-analysis;(9, 10, 17, 18, 27-48) highlighted in the PRISMA flow diagram (Figure 1).(49)

Supplementary Table S3 details characteristics of the included studies. Only observational studies were found; 24 cohort, and two case-control studies, (10, 40) and all studies were published between 1996 and 2021. The total number of subjects investigated across case-control was 61,710, compared to 5,346,953 across cohort studies. The study size varied from 247 to 1,281,418. The miscarriage definition was rarely stated, however, ranged from loss of fetus at either 20 (n=3),(10, 42, 45) 22 (n=2)(17, 18) or 24 weeks' (n=1)(44) gestation.

For peer review only

A mixture of comparator groups were utilised across studies. All studies had a comparator group of unexposed individuals without depression; some papers used additional comparator groups including depressed pregnant women exposed to an alternative antidepressant to the primary specified type, unexposed depressed pregnant women,(17, 18, 37, 42, 43) and pregnant individuals using antidepressants whereby the indication for treatment wasn't depression.(17, 32, 43) Exposure status was predominantly identified through two methods. Firstly, 16 studies utilised self-report to teratogen information services through either the individual or their physician.(9, 27-34, 36, 38, 40, 41, 43-45) Secondly, eight studies used prescription databases.(10, 17, 18, 37, 39, 42, 47, 48) Individual antidepressant types were investigated, including 11 studies examining SSRIs(18, 27-29, 31, 34, 37-39, 43, 45) and five SNRIs.(29, 41, 44, 46, 48)

Meta-analyses

Across all 26 studies using an unexposed general population comparator, antidepressant use was associated with miscarriage. Figure 2 illustrates the summary effect estimate of 1.29 (95% CI: 1.21-1.38), which included 15 adjusted estimates. However, when stratifying for maternal depression and utilising a comparator of unexposed depressed pregnant women, the summary estimate decreased to 1.16 (0.98-1.36) (Figure 3).

Additionally, studies were separated which investigated SSRIs or SNRIs and their possible varying risks. When comparing SSRI use and no antidepressant use, the summary estimate for the outcome of miscarriage was 1.32 (1.16-1.50). Alternatively, when comparing SNRI use with no antidepressant use, a summary estimate of 1.46 (1.12-1.91) was produced (Figure 2).

Subgroup analyses investigating SSRIs and SNRIs separately were performed again after restricting to a depressed cohort. When comparing SSRI use and individuals using other antidepressant types, it produced an attenuated summary estimate of 1.04 (0.93-1.16). Additionally, the summary estimate comparing SNRI against other antidepressant use on the outcome of miscarriage was 1.19 (1.02-1.39) (Figure 3).

For peer review only

When investigating all antidepressant types, the heterogeneity between studies remained high, with an I^2 of 70.8% when using a general population comparator, and 67.7% when using an unexposed depressed comparator. The analysis comparing SSRIs and SNRIs against non-users also had high heterogeneity levels, with 83.3% and 52.2%, respectively. However, the analyses comparing SSRIs and SNRIs to other antidepressants had a low heterogeneity of 0% (Supplementary Table S4).

Risk of bias

The ROBINS-I tool found much of the literature to be at serious RoB. Supplementary Table S5 highlights that only two out of 26 papers had an overall moderate RoB,(44, 48) compared to 19 deemed serious. This tool highlighted a key area of bias being confounding. The three crucial confounding domains identified for this field were indication for treatment, individual characteristics, and lifestyle exposures. The confounding variables believed to be most important included presence and severity of depression, as well as gestational age. Although a large proportion of studies stratified by depression (64%), only 16% and 36% of studies accounted for depression severity and gestational age, respectively (Supplementary Table S6). Confounding adjustments varied dramatically between studies suggesting a potential heterogeneity source. As very few confounding variables were included in these studies, it limits the reliability of the results of this systematic review.

Finally, publication bias was assessed through visual inspection of the funnel plot (Supplementary Figure S1) and the Egger test. Asymmetry of the funnel plot indicated potential for publication bias, and the Egger test produced a p-value of 0.001.

Sensitivity analysis

Papers with an overall critical RoB or inadequate information to determine their bias category were removed (n=5). This sensitivity analysis evidenced that these papers weren't influencing the overall effect estimate for the general population analyses (Supplementary Table S4).

Discussion

For peer review only

Summary of evidence

This is the largest and most up-to-date review investigating the association between antidepressant use during pregnancy and miscarriage, with around twice as many studies included than previous reviews.(19-21) After pooling best estimates from 26 studies, we found little evidence to suggest an increased risk of miscarriage among antidepressant users after stratifying by maternal depression, with CIs spanning the null. The increased risk of miscarriage among pregnant women taking antidepressants compared to unexposed pregnant women in the general population is likely driven by confounding by indication.

Interpretation of findings

The general population analysis result was comparable to other smaller meta-analyses, which found effect estimates of 1.45, 1.49 and 1.47.(8, 19, 21) The increased estimates found by these papers are likely driven by fewer included studies, with 11, 14, and six respectively, compared to 26 in our study. The elevated effect estimates when utilising a general population control could likely be driven by confounding by indication, a bias which is lessened when comparing with depressed controls. To the best of our knowledge, meta-analyses in the field investigating miscarriage have not yet utilised an unexposed depressed comparator; thus, no comparable results exist. As only five studies were used in this subgroup meta-analysis, the summary estimate lacks power and robustness, inducing potential for chance findings, highlighted by large standard errors. Additionally, this weak miscarriage risk could be reflecting depression severity, whereby unexposed depressed individuals have a lower severity of depression than exposed individuals. Studies have indicated miscarriage risk between individuals continuing and discontinuing antidepressant use upon start of pregnancy is similar.(18, 48) Therefore, future studies should incorporate the comparator of discontinued antidepressant use to allow for more comparable depression severity. Furthermore, other confounding factors that weren't adequately controlled for could be biasing this estimate, thus, adjusting for all key confounders is essential in future studies to minimise residual confounding.

Our study found that SNRIs carry a greater risk of miscarriage than SSRIs when compared to an unexposed general population comparator group. No meta-analyses have been conducted on SNRIs to our knowledge, likely due to the higher prevalence of other

For peer review only

antidepressant use during pregnancy, for example SSRIs.(12) Previous reviews utilising a fixed-effects method found odds ratios of 1.70 and 1.87 when comparing SSRIs to unexposed general population comparators.(50, 51) The lower effect estimate of 1.32 observed in our study may be partially driven by the use of a random-effects model to account for high heterogeneity, as well as the inclusion of an additional eight studies. The elevated point estimate for SNRIs compared with SSRIs may suggest that miscarriage risk differs between antidepressant classes. Alternatively, these estimates could be highlighting confounding by indication, which was further explored in a set of subgroup analyses that stratified by maternal depression. The summary effect estimates between both SSRIs and SNRIs and other antidepressant classes attenuated towards the null, thus, emphasising the potential impact of confounding by indication. The SSRI analyses had consistently lower estimates, including against other antidepressant classes, when comparing pooled estimates with those from the SNRI analyses. The difference in point estimates within the subgroup analysis could be reflecting the differing population types, as SSRIs are used as first line treatment for depression,(52) whereas SNRIs are often utilised in individuals with more severe depression or when first line options may have not worked.(53) Thus, these results provide evidence for the potential impact of confounding by depression severity, whereby the elevated pooled estimate from the SNRI analysis could be driven by non-exchangeability between individuals who use different classes of antidepressants. Confounding by disease severity was highlighted in the largest included study in the SNRI analysis which discussed that the risk difference observed between duloxetine (SNRI) and SSRI users could be depression severity and residual confounding.(48) However, given the confidence intervals overlap for the subgroup analyses of SSRIs and SNRIs, it is plausible that the true pooled estimates for each analysis are the same. Future network meta-analyses may help elucidate the intricacies of the relationship between individual antidepressant medications or classes and miscarriage.

Limitations of included studies

The included studies had several limitations. Firstly, only 8% of studies had a moderate overall RoB, due to the limited level of confounding adjustment in papers within the field. Only eight papers adjusted for any variable within all three key confounding domains (Supplementary Table S6). Therefore, the included studies may suffer from residual

For peer review only - http://bmjopen.bmj.com/site/about/guidelines.xhtml

confounding, as strong risk factors for miscarriage are likely imbalanced between exposure groups,(44) and any effect estimates may be biased away from the null.

A second limitation of the included studies is information bias due to exposure misclassification. Exposure was assessed mainly via self-report from physician or individual, or prescription databases. However, only 60% agreement has been found between prescription and self-report data,(18) with conflicting evidence as to the most accurate method to assess exposure to medication.(54, 55) Teratogen surveillance data was used in several included studies, however, it poses difficulty as women enquiring about antidepressant exposure are automatically classified as exposed, when they may discontinue use after enquiry. Prescription database error could be less impactful, as it likely suffers from non-differential misclassification and biases the estimate towards the null.(18) Although, self-report data could be more accurate in terms of ingested drug usage. Nevertheless, sensitivity analyses comparing self-report and prescription database inaccuracies indicate exposure misclassification does not impact effect estimates significantly, and only reduces precision.(18)

Thirdly, selection bias is another common methodological limitation in this field. A significant issue observed is that the start of intervention and follow-up do not coincide. This results in conditioning on women who haven't experienced a miscarriage between the start of intervention and follow-up but excluding eligible individuals who have. Additionally, exposed individuals are more likely to be in consultation with their healthcare professional when planning a pregnancy to discuss potential risks of antidepressants, compared to unexposed individuals. This results in exposed individuals having a longer observed risk period and as such, more chance of detecting and recording miscarriages, therefore biasing results away from the null. (56) These methodological errors contributing to a bias which occurs when individuals who have already experienced the outcome of interest at the point of study commencement not being included,(57) referred to as left truncation.(58)

Strengths and limitations of review

Our review has important strengths not addressed in any previous review investigating miscarriage. Confounding by indication and depression severity was a key part of the

For peer review only

analysis and results, elucidating the true independent effect of antidepressant use during pregnancy. As this important bias has not previously been addressed in any other review in the field, it emphasises the requirement for accounting for psychiatric illness in future reviews and studies to avoid biased estimates. To the best of our knowledge, our study was also the first review in this field to assess RoB using the ROBINS-I tool. This tool gave a significant advantage over the commonly used Newcastle-Ottawa scale and highlighted the need for more thorough confounding adjustment and reporting of missing data in future studies. Additionally, the issues surrounding exposure recall bias were minimised using mainly cohort studies, and the included case-control studies having prospective exposure measurement. Finally, 26 studies published up to 2021 were identified and used in the review through an extremely thorough search strategy, thus enhancing statistical power and insight over previous reviews.

The review also had limitations. The studies included could suffer from publication bias as few studies were distributed around the null line and most found associations that transcended the null (Supplementary Figure S1). Additionally, small studies producing inverse relationships are absent from the field. This conclusion was supported by the small p-value produced in the Egger test. If only positive results are being published this could result in exaggerated review results. However, these conclusions may not be driven by publication bias, but instead either real causal associations or lack of methodological rigour throughout the studies.

High heterogeneity also existed between papers, likely due to the included studies investigating different research questions, implementing different methodologies and populations, timing of exposure and confounding adjustment, thereby reducing the quality of the meta-analysis. Additionally, the lack of available data prevented a trimester specific subgroup analysis. Previous studies have identified first trimester miscarriage risk with antidepressant use to be higher compared to second trimester,(18) indicating antidepressant exposure could be time sensitive. However, this could reflect the time window for highest risk of miscarriage being between week 7 and 12, during first trimester.(59) Future reviews should aim to incorporate this subgroup analysis to investigate whether it alters conclusions made.

For peer review only

Conclusion

In conclusion, we did not find any clear evidence to suggest antidepressants increase the risk of miscarriage, though the results should be treated with caution due to the large heterogeneity present. This report highlights the effect of confounding by indication on previous literature. As RCTs cannot be utilised, future observational studies with larger sample sizes and more robust confounding adjustment, especially in relation to indication for treatment are needed. Triangulating results with differing methodologies should help strengthen causal inference. Additionally, network meta-analyses are needed to fully investigate the differing risk between antidepressant classes. Overall, the present study provides a summary of the most up-to-date findings of miscarriage risk following antidepressant use during pregnancy, to better inform clinicians and women when weighing up treatment for depression during pregnancy.

For peer review only

Declaration of interests
All authors declare no competing interests.

Funding
SS is supported by an NIHR predoctoral fellowship (NIHR301109). FM is supported by the Wellcome Trust (WT 218495/Z/19/Z). HF and DR acknowledge support from the NIH (1R01NS107607). This research was also supported by the NIHR Biomedical Research Centre at the University of Bristol and the University Hospitals Bristol and Weston NHS Foundation Trust.

Acknowledgements
The views in this research are of the authors and not of the funders. The authors thank the corresponding authors and coauthors of the studies included in this review. We thank Professor Julian Higgins who was extremely helpful with queries involving the ROBINS-I tool.

Author Contributions
All authors helped with interpretation of results. SS did the full-text screening, data extraction, risk of bias assessment, statistical analysis and wrote the report. FM did the title and abstract screening, full text screening, the second review of data extraction and risk of bias assessment, statistical analysis and had input on the report writing. HF did the title and abstract screening, full text screening, and had input on all other elements of the paper. DR suggested the research question, provided supervision and comment on the manuscript.

Data sharing statement
Please contact the first author (SS) if you would like to see any data not included in the article or supplementary material.

For peer review only

References

1. Disease GBD, Injury I, Prevalence C. Global, regional, and national incidence, prevalence, and years lived with disability for 354 diseases and injuries for 195 countries and territories, 1990-2017: a systematic analysis for the Global Burden of Disease Study 2017. Lancet. 2018;392(10159):1789-858.
2. Tak CR, Job KM, Schoen-Gentry K, Campbell SC, Carroll P, Costantine M, et al. The impact of exposure to antidepressant medications during pregnancy on neonatal outcomes: a review of retrospective database cohort studies. Eur J Clin Pharmacol. 2017;73(9):1055-69.
3. Rai D, Lee BK, Dalman C, Newschaffer C, Lewis G, Magnusson C. Antidepressants during pregnancy and autism in offspring: population based cohort study. Bmj. 2017;358:j2811.
4. Cooper WO, Willy ME, Pont SJ, Ray WA. Increasing use of antidepressants in pregnancy. Am J Obstet Gynecol. 2007;196(6):544 e1-5.
5. Pearson RM, Carnegie RE, Cree C, Rollings C, Rena-Jones L, Evans J, et al. Prevalence of Prenatal Depression Symptoms Among 2 Generations of Pregnant Mothers: The Avon Longitudinal Study of Parents and Children. JAMA Netw Open. 2018;1(3):e180725.
6. Ford E, Lee S, Shakespeare J, Ayers S. Diagnosis and management of perinatal depression and anxiety in general practice: a meta-synthesis of qualitative studies. Br J Gen Pract. 2017;67(661):e538-e46.
7. Quenby S, Gallos ID, Dhillon-Smith RK, Podesek M, Stephenson MD, Fisher J, et al. Miscarriage matters: the epidemiological, physical, psychological, and economic costs of early pregnancy loss. Lancet. 2021;397(10285):1658-67.
8. Hemels ME, Einarson A, Koren G, Lanctot KL, Einarson TR. Antidepressant use during pregnancy and the rates of spontaneous abortions: a meta-analysis. Ann Pharmacother. 2005;39(5):803-9.
9. Einarson A, Choi J, Einarson TR, Koren G. Rates of spontaneous and therapeutic abortions following use of antidepressants in pregnancy: results from a large prospective database. J Obstet Gynaecol Can. 2009;31(5):452-6.
10. Nakhai-Pour HR, Broy P, Berard A. Use of antidepressants during pregnancy and the risk of spontaneous abortion. Cmaj. 2010;182(10):1031-7.
11. Ewing G, Tatarchuk Y, Appleby D, Schwartz N, Kim D. Placental transfer of antidepressant medications: implications for postnatal adaptation syndrome. Clin Pharmacokinet. 2015;54(4):359-70.
12. Anderson HD, Pace WD, Libby AM, West DR, Valuck RJ. Rates of 5 common antidepressant side effects among new adult and adolescent cases of depression: a retrospective US claims study. Clin Ther. 2012;34(1):113-23.
13. Ornoy A, Koren G. Selective Serotonin Reuptake Inhibitors during Pregnancy: Do We Have Now More Definite Answers Related to Prenatal Exposure? Birth Defects Res Part A Clin Mol Teratol. 2017;109(12):898-908.
14. Keyte A, Hutson MR. The neural crest in cardiac congenital anomalies. Differentiation. 2012;84(1):25-40.
15. Marinescu IP, Foarfa MC, Pirlog MC, Turculeanu A. Prenatal depression and stress - risk factors for placental pathology and spontaneous abortion. Rom J Morphol Embryol. 2014;55(3 Suppl):1155-60.
16. Chan J, Natekar A, Einarson A, Koren G. Risks of untreated depression in pregnancy. Can Fam Physician. 2014;60(3):242-3.

For peer review only

17. Kjaersgaard MIS, Parner ET, Vestergaard M, Sorensen MJ, Olsen J, Christensen J, et al. Prenatal Antidepressant Exposure and Risk of Spontaneous Abortion - A Population-Based Study. PLoS ONE. 2013;8(8).
18. Johansen RL, Mortensen LH, Andersen AM, Hansen AV, Strandberg-Larsen K. Maternal use of selective serotonin reuptake inhibitors and risk of miscarriage - assessing potential biases. Paediatr Perinat Epidemiol. 2015;29(1):72-81.
19. Xing D, Wu R, Chen L, Wang T. Maternal use of antidepressants during pregnancy and risks for adverse perinatal outcomes: a meta-analysis. Journal of Psychosomatic Research. 2020;137 (no pagination).
20. Udechuku A, Nguyen T, Hill R, Szego K. Antidepressants in pregnancy: a systematic review. Aust N Z J Psychiatry. 2010;44(11):978-96.
21. Ross LE, Grigoriadis S, Mamisashvili L, Vonderporten EH, Roerecke M, Rehm J, et al. Selected pregnancy and delivery outcomes after exposure to antidepressant medication: a systematic review and meta-analysis. JAMA Psychiatry. 2013;70(4):436-43.
22. Khalifeh H, Molyneaux E, Brauer R, Vigod S, Howard LM. Patient decision aids for antidepressant use in pregnancy: a pilot randomised controlled trial in the UK. BJGP Open. 2019.
23. Sterne JA, Hernan MA, Reeves BC, Savovic J, Berkman ND, Viswanathan M, et al. ROBINS-I: a tool for assessing risk of bias in non-randomised studies of interventions. Bmj. 2016;355:i4919.
24. Cummings P. The relative merits of risk ratios and odds ratios. Arch Pediatr Adolesc Med. 2009;163(5):438-45.
25. Higgins JP, Thompson SG, Deeks JJ, Altman DG. Measuring inconsistency in meta-analyses. Bmj. 2003;327(7414):557-60.
26. Lin L, Chu H. Quantifying publication bias in meta-analysis. Biometrics. 2018;74(3):785-94.
27. Chambers CD, Johnson KA, Dick LM, Felix RJ, Jones KL. Birth outcomes in pregnant women taking fluoxetine. N Engl J Med. 1996;335(14):1010-5.
28. Kulin NA, Pastuszak A, Sage SR, Schick-Boschetto B, Spivey G, Feldkamp M, et al. Pregnancy outcome following maternal use of the new selective serotonin reuptake inhibitors: a prospective controlled multicenter study. Jama. 1998;279(8):609-10.
29. Einarson A, Fatoye B, Sarkar M, Lavigne SV, Brochu J, Chambers C, et al. Pregnancy outcome following gestational exposure to venlafaxine: a multicenter prospective controlled study. Am J Psychiatry. 2001;158(10):1728-30.
30. Einarson A, Bonari L, Voyer-Lavigne S, Addis A, Matsui D, Johnson Y, et al. A multicentre prospective controlled study to determine the safety of trazodone and nefazodone use during pregnancy. Canadian Journal of Psychiatry. 2003;48(2):106-10.
31. Sivojelezova A, Shuhaiber S, Sarkissian L, Einarson A, Koren G. Citalopram use in pregnancy: Prospective comparative evaluation of pregnancy and fetal outcome. American Journal of Obstetrics and Gynecology. 2005;193(6):2004-9.
32. Chun-Fai-Chan B, Koren G, Fayez I, Kalra S, Voyer-Lavigne S, Boshier A, et al. Pregnancy outcome of women exposed to bupropion during pregnancy: a prospective comparative study. Am J Obstet Gynecol. 2005;192(3):932-6.
33. Djulus J, Koren G, Einarson TR, Wilton L, Shakir S, Diav-Citrin O, et al. Exposure to mirtazapine during pregnancy: A prospective, comparative study of birth outcomes. J Clin Psychiatry. 2006;67(8):1280-4.

For peer review only

34. Paulus W, Schloemp S, Stoz F. Paroxetine exposure during pregnancy: Increased risk of congenital malformations? Archives of Gynecology and Obstetrics. 2010;282:S79.
35. Chan RL, Jonsson Funk ML, Savitz DA, Hartmann KE. Self-reported antidepressants use and spontaneous abortion. American Journal of Epidemiology. 2011;173:S140.
36. Einarson A, Choi J, Koren G, Einarson T. Outcomes of infants exposed to multiple antidepressants during pregnancy: results of a cohort study. J Popul Ther Clin Pharmacol. 2011;18(2):e390-6.
37. Ban L, Tata LJ, West J, Fiaschi L, Gibson JE. Live and non-live pregnancy outcomes among women with depression and anxiety: A population-based study. PLoS ONE. 2012;7(8).
38. Klieger-Grossmann C, Weitzner B, Panchaud A, Pistelli A, Einarson T, Koren G, et al. Pregnancy outcomes following use of escitalopram: a prospective comparative cohort study. J Clin Pharmacol. 2012;52(5):766-70.
39. Andersen JT, Andersen NL, Horwitz H, Poulsen HE, Jimenez-Solem E. Exposure to selective serotonin reuptake inhibitors in early pregnancy and the risk of miscarriage. Obstet Gynecol. 2014;124(4):655-61.
40. Abadie D, Hurault-Delarue C, Damase-Michel C, Montastruc JL, Lacroix I. Medication exposure and spontaneous abortion: A case-control study using a French medical database. Clinical and Experimental Obstetrics and Gynecology. 2015;42(4):431-6.
41. Te Winkel B, Beghin D, Pistelli A, De Santis M, Cassina M, Eleftheriou G, et al. Venlafaxine exposure in pregnancy, a multicenter ENTIS study. Pharmacoepidemiology and Drug Safety. 2016;25 (Supplement 3):325-6.
42. Almeida ND, Basso O, Abrahamowicz M, Gagnon R, Tamblyn R. Risk of Miscarriage in Women Receiving Antidepressants in Early Pregnancy, Correcting for Induced Abortions. Epidemiology. 2016;27(4):538-46.
43. Evans-Hoeker EA, Eisenberg E, Diamond MP, Legro RS, Alvero R, Coutifaris C, et al. Major depression, antidepressant use, and male and female fertility. Fertility and Sterility. 2018;109(5):879-87.
44. Richardson JL, Martin F, Dunstan H, Greenall A, Stephens S, Yates LM, et al. Pregnancy outcomes following maternal venlafaxine use: A prospective observational comparative cohort study. Reprod Toxicol. 2019;84:108-13.
45. Wu P, Velez Edwards DR, Gorrindo P, Sundermann AC, Torstenson ES, Jones SH, et al. Association between First Trimester Antidepressant Use and Risk of Spontaneous Abortion. Pharmacotherapy. 2019;39(9):889-98.
46. Bahat M, Shechtman S, Diav-Citrin O. Pregnancy outcome after first trimester exposure to Duloxetine. Reprod Toxicol. 2020;97:3.
47. Kolding L, Ehrenstein V, Pedersen L, Sandager P, Petersen OB, Uldbjerg N, et al. Antidepressant use in pregnancy and severe cardiac malformations: Danish register-based study. Bjog. 2021.
48. Ankarfeldt MZ, Petersen J, Andersen JT, Fernandes MFS, Li H, Motsko SP, et al. Duloxetine Exposure During Pregnancy and the Risk of Spontaneous and Elective Abortion: A Danish Nationwide Observational Study. Drugs Real World Outcomes. 2021;8(3):289-99.
49. Page MJ, McKenzie JE, Bossuyt PM, Boutron I, Hoffmann TC, Mulrow CD, et al. The PRISMA 2020 statement: an updated guideline for reporting systematic reviews. Bmj. 2021;372:n71.

For peer review only

50. Rahimi R, Nikfar S, Abdollahi M. Pregnancy outcomes following exposure to serotonin reuptake inhibitors: a meta-analysis of clinical trials. Reprod Toxicol. 2006;22(4):571-5.
51. Nikfar S, Rahimi R, Hendoiee N, Abdollahi M. Increasing the risk of spontaneous abortion and major malformations in newborns following use of serotonin reuptake inhibitors during pregnancy: A systematic review and updated meta-analysis. Daru. 2012;20(1):75.
52. Kato T, Furukawa TA, Mantani A, Kurata K, Kubouchi H, Hirota S, et al. Optimising first- and second-line treatment strategies for untreated major depressive disorder - the SUND study: a pragmatic, multi-centre, assessor-blinded randomised controlled trial. BMC Med. 2018;16(1):103.
53. Isaac MT. Treating depression with SNRIs: who will benefit most? CNS Spectr. 2008;13(7 Suppl 11):15-21.
54. West SL, Savitz DA, Koch G, Strom BL, Guess HA, Hartzema A. Recall accuracy for prescription medications: self-report compared with database information. Am J Epidemiol. 1995;142(10):1103-12.
55. Hafferty JD, Campbell AI, Navrady LB, Adams MJ, MacIntyre D, Lawrie SM, et al. Self-reported medication use validated through record linkage to national prescribing data. J Clin Epidemiol. 2018;94:132-42.
56. Schisterman EF, Cole SR, Ye A, Platt RW. Accuracy loss due to selection bias in cohort studies with left truncation. Paediatr Perinat Epidemiol. 2013;27(5):491-502.
57. Cain KC, Harlow SD, Little RJ, Nan B, Yosef M, Taffe JR, et al. Bias due to left truncation and left censoring in longitudinal studies of developmental and disease processes. Am J Epidemiol. 2011;173(9):1078-84.
58. Howards PP, Hertz-Picciotto I, Poole C. Conditions for bias from differential left truncation. Am J Epidemiol. 2007;165(4):444-52.
59. Sanchez Ortiz S, Huerta C, Llorente-Garcia A, Ortega P, Astasio P, Cea-Soriano L. A Validation Study on the Frequency and Natural History of Miscarriages Using the Spanish Primary Care Database BIFAP. Healthcare (Basel). 2021;9(5).

For peer review only

For peer review only

PRISMA 2020 flow diagram for new systematic reviews which included searches of databases and registers only

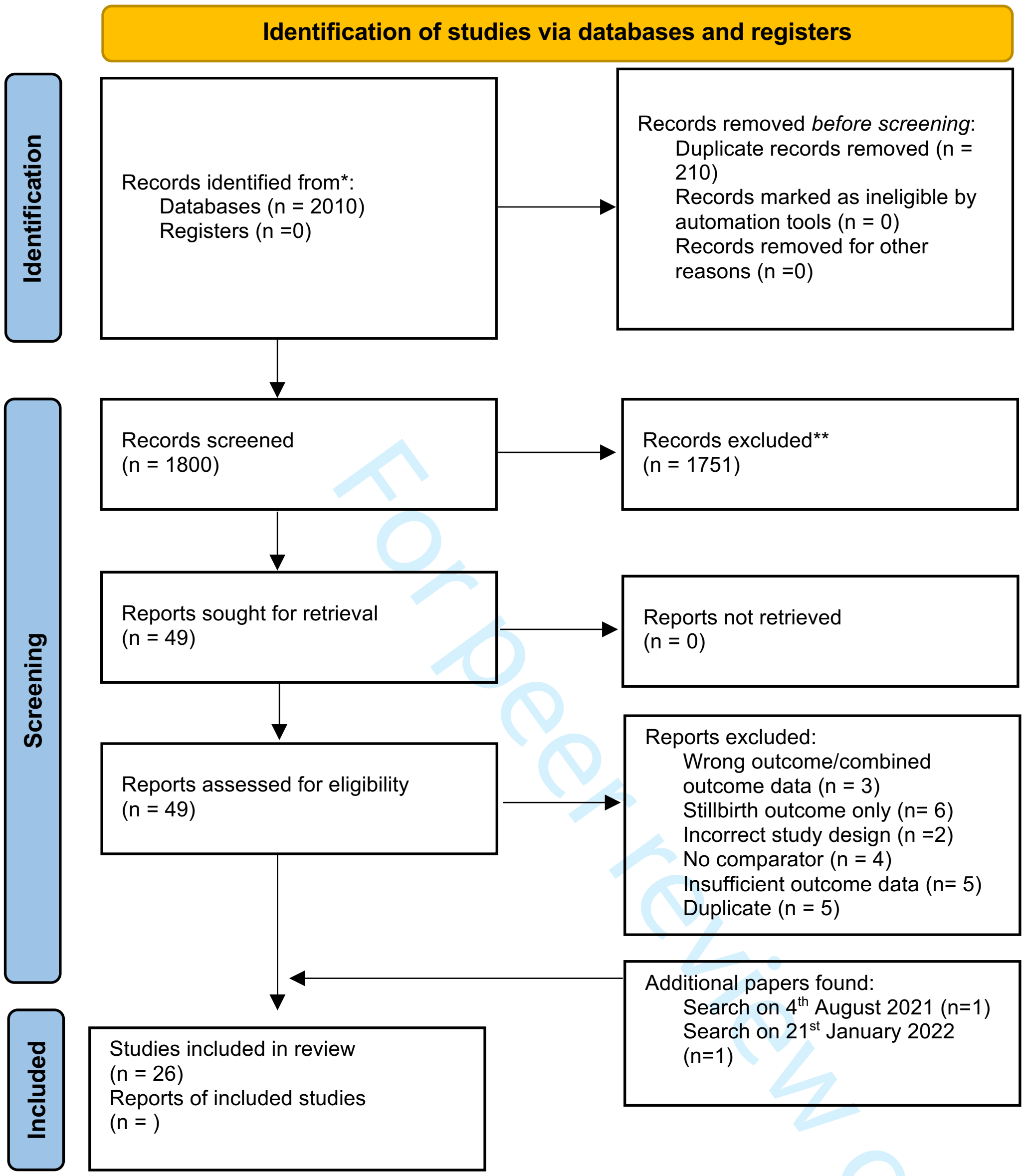

Figure 1. PRISMA flow diagram

Flow diagram illustrating the number of included and excluded studies at each stage of screening, and reasons for exclusion. This version has been adapted to include the element of additional papers found in following searches.

For peer review only

Study author (year)	Treated n/N	Controls n/N	Odds ratio (95% CI)	Weight %
**Any antidepressant use vs no use**				
Chambers *et al.* (1996)	23/169	22/254	1.66 (0.89, 3.09)	0.69
Kulin *et al.* (1998)	30/267	21/267	1.48 (0.83, 2.66)	0.77
Einarson *et al.* (2001)	18/150	11/150	1.72 (0.78, 3.79)	0.45
Einarson *et al.* (2003)	20/147	12/147	1.77 (0.83, 3.77)	0.48
Sivojelezova *et al.* (2005)	14/132	13/132	1.09 (0.49, 2.41)	0.44
Chun-Fai-Chan *et al.* (2005)	20/136	6/133	3.65 (1.42, 9.40)	0.31
Djulus *et al.* (2006)	20/104	11/104	2.01 (0.91, 4.45)	0.44
*Einarson *et al.* (2009)	122/937	75/937	1.38 (0.98, 1.94)	1.89
*Nakhai-Pour *et al.* (2010)	284/1685	4840/54679	1.68 (1.38, 2.05)	3.70
Paulus *et al.* (2010)	23/202	81/714	1.00 (0.61, 1.64)	1.05
*Chan *et al.* (2011)	NA	NA	1.54 (1.09, 2.18)	1.85
*Einarson *et al.* (2011)	8/89	8/89	1.07 (0.51, 2.24)	0.50
*Ban *et al.* (2012)	1982/13151	47258/390665	1.33 (1.18, 1.49)	5.59
Klieger-Grossman *et al.* (2012)	32/213	18/212	1.91 (1.03, 3.51)	0.71
*Kjaesgaard *et al.* (2013)	210/1674	110377/818426	1.27 (1.22, 1.33)	7.15
*Andersen *et al.* (2014)	2883/22884	139201/1256956	1.14 (1.10, 1.18)	7.26
*Abadie *et al.* (2015)	170/899	668/4447	1.20 (0.98, 1.46)	3.72
*Johansen *et al.* (2015)	2359/20612	124333/1165124	1.07 (1.03, 1.12)	7.17
Te Winkel *et al.* (2016)	85/732	46/730	1.95 (1.34, 2.84)	1.64
*Almeida *et al.* (2016)	165/1955	1685/28343	1.30 (1.11, 1.52)	4.65
*Evans-Hoeker *et al.* (2018)	16/90	118/1484	1.92 (1.22, 3.02)	1.20
*Richardson *et al.* (2019)	46/281	140/1405	1.26 (0.83, 1.91)	1.38
*Wu *et al.* (2019)	42/223	617/5228	1.34 (0.97, 1.85)	2.05
*Bahat *et al.* (2020)	NA	NA	1.89 (1.04, 3.43)	0.74
Kolding *et al.* (2021)	134/4105	10739/353581	1.08 (0.91, 1.28)	4.25
*Ankarfeldt' *et al.* (2021)	143/1201	103367/984447	1.14 (0.96, 1.35)	4.39
Subgroup (I-squared = 70.8%, p = 0.000)			1.29 (1.21, 1.38)	64.46
**SSRI use vs no use**				
Chambers *et al.* (1996)	23/169	22/254	1.66 (0.89, 3.09)	0.69
Kulin *et al.* (1998)	30/267	21/267	1.48 (0.83, 2.66)	0.77
Einarson *et al.* (2001)	16/150	11/150	1.51 (0.68, 3.37)	0.43
Sivojelezova *et al.* (2005)	14/132	13/132	1.09 (0.49, 2.41)	0.44
Paulus *et al.* (2010)	23/202	81/714	1.00 (0.61, 1.64)	1.05
*Ban *et al.* (2012)	1539/10132	47258/390665	1.50 (1.35, 1.66)	5.88
Klieger-Grossman *et al.* (2012)	32/213	18/212	1.91 (1.03, 3.51)	0.71
*Andersen *et al.* (2014)	2883/22884	139201/1256956	1.27 (1.22, 1.33)	7.15
*Johansen *et al.* (2015)	NA	NA	1.07 (1.03, 1.12)	7.17
*Evans-Hoeker *et al.* (2018)	11/57	118/1484	1.73 (1.00, 3.00)	0.86
*Wu *et al.* (2019)	35/179	617/5228	1.45 (1.02, 2.06)	1.81
Subgroup (I-squared = 83.3%, p = 0.000)			1.32 (1.16, 1.50)	26.95
**SNRI use vs no use**				
Einarson *et al.* (2001)	18/150	11/150	1.72 (0.78, 3.79)	0.45
Te Winkel *et al.* (2016)	85/732	46/730	1.95 (1.34, 2.84)	1.64
*Richardson *et al.* (2019)	46/281	140/1405	1.26 (0.83, 1.91)	1.38
Bahat *et al.* (2020)	NA	NA	1.89 (1.04, 3.43)	0.74
*Ankarfeldt' *et al.* (2021)	143/1201	103367/984447	1.14 (0.96, 1.35)	4.39
Subgroup (I-squared = 55.2%, p = 0.063)			1.46 (1.12, 1.91)	8.59

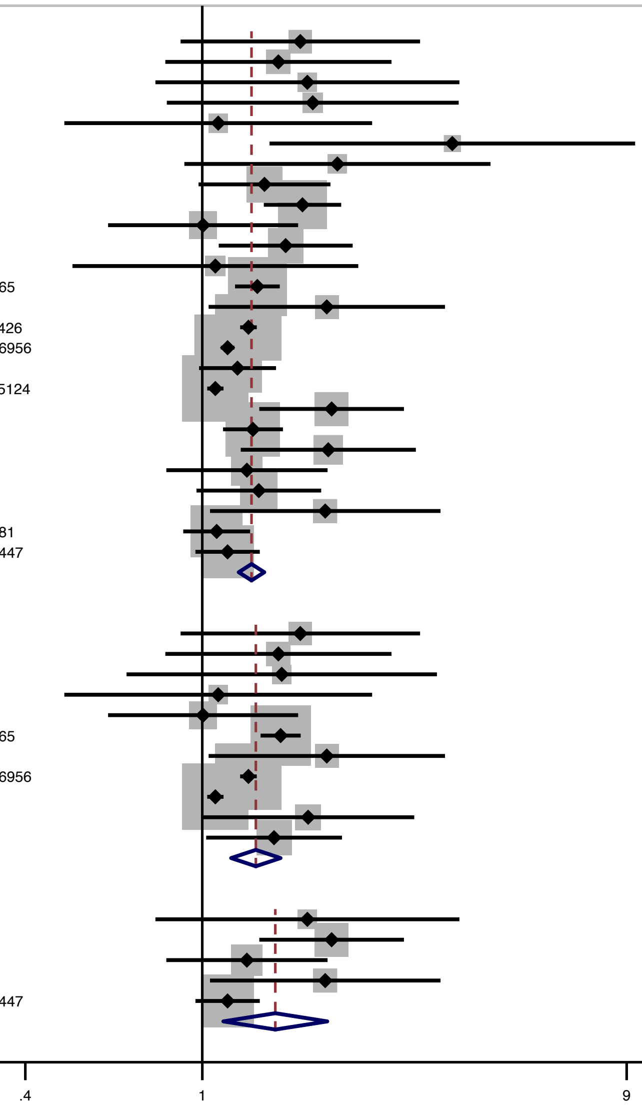

NOTE: Weights are from random-effects model

Figure 2. Subgroup meta-analyses utilising general population comparator

Three subgroup analyses conduced. First subgroup assessing any antidepressant use compared to unexposed individuals in general population. Second subgroup comparing SSRI use and unexposed general population individuals. Third subgroup comparing between SNRI use and unexposed general population individuals.

*These studies had an adjusted estimate from multivariable model which was pooled in the meta-analysis rather than raw data, therefore the treated and controls (n/N) data does not correspond to the odds ratio shown.

'The unexposed comparator in this paper was classified as 'duloxetine non-exposed' which could have also included users of other antidepressant types.

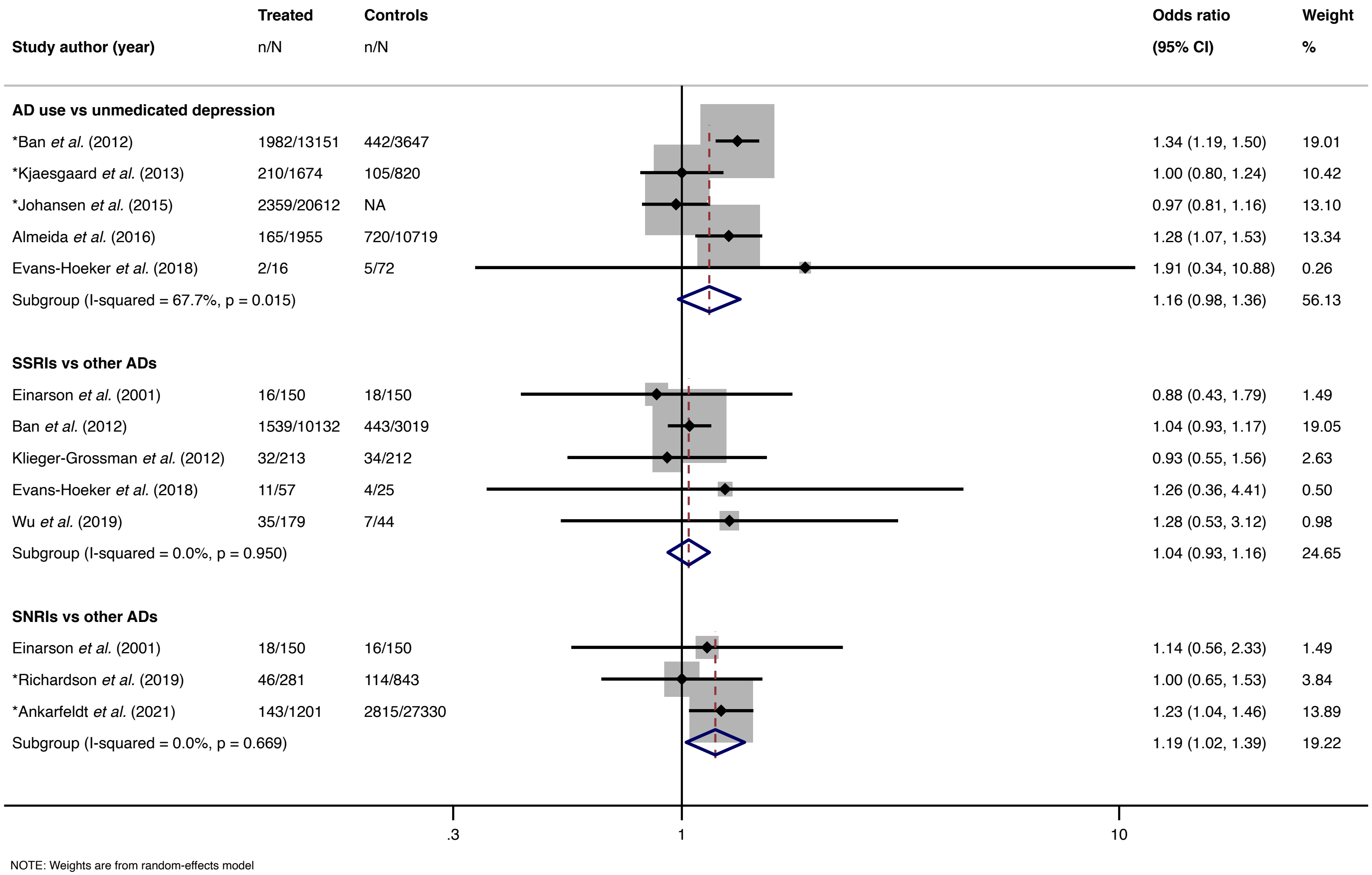

Figure 3. Three random-effects meta-analysis utilising depressed cohort comparator

First subgroup assessing any antidepressant use against unmediated depressed individuals. Second subgroup comparing SSRI use and different types of antidepressants (this includes data from papers which compared SSRIs and SNRIs, SSRIs and TCAs, SSRIs and any other antidepressants). Third subgroup analysis compared SNRI use and different types of antidepressants (this includes data from papers comparing SNRI and SSRI).

* These studies had an adjusted estimate from multivariable model which was pooled in the meta-analysis rather than raw data, therefore the treated and controls (n/N) data does not correspond to the odds ratio shown

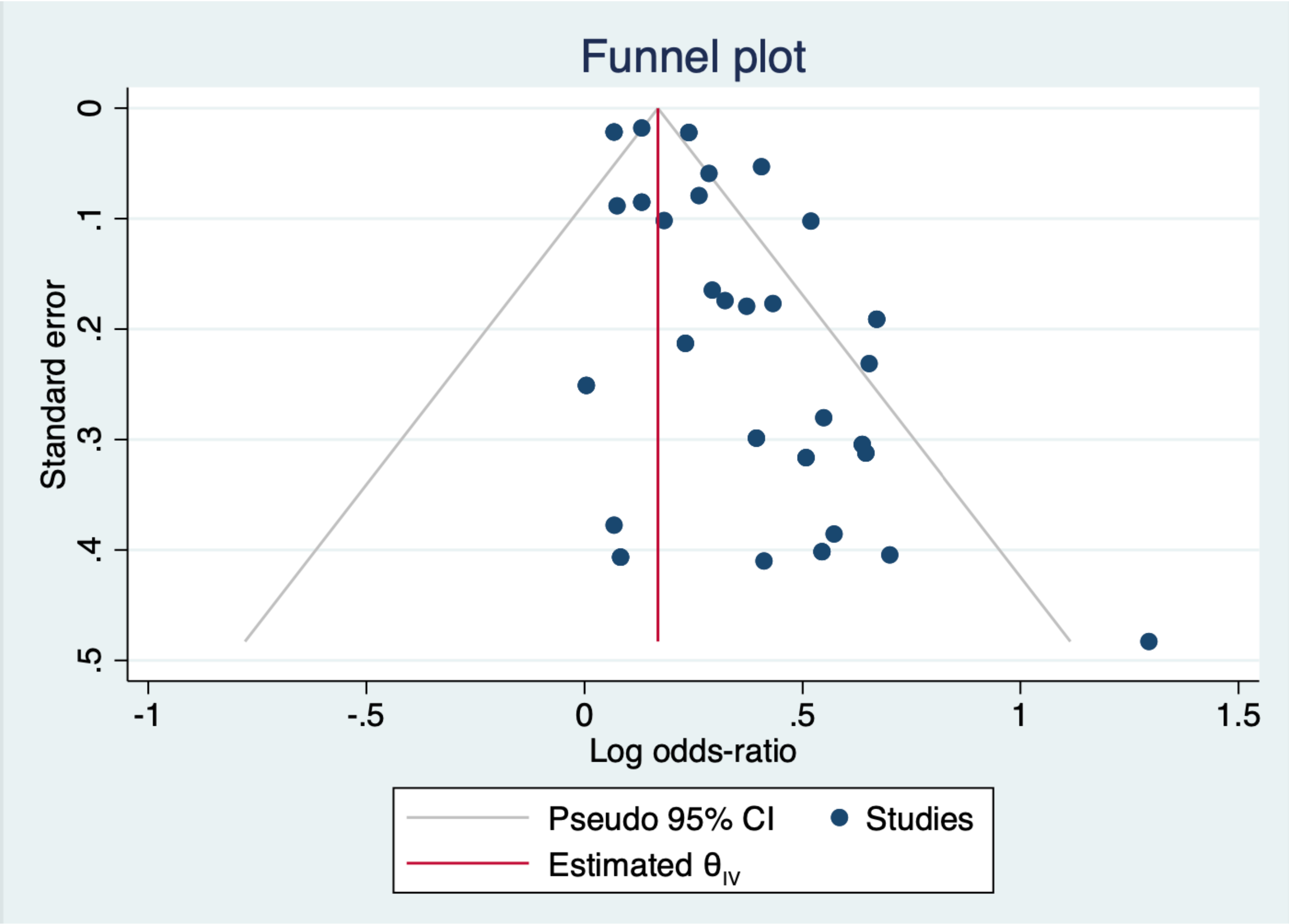

S1 Figure. Funnel plot

For peer review only

S1 Table. Search strategy

1. exp antidepressant agent/

2. Serotonin uptake inhibitor/

3. Monoamine oxidase inhibitor/

4. (anti*depress or antidepress* or tricyclic* or TCA* or heterocyclic* or serotonin uptake or SSRI* or SNRI* or monoamine oxidase inhibitor* or MAOI*).tw.

5. (desipramine or imipramine or clomipramine or opipramol or trimipramine or lofepramine or dibenzepin or amitriptyline or nortriptyline or protriptyline or doxepin or iprindole or melitracen or butriptyline or dosulepin or amoxapine or dimetacrine or amineptine or maprotiline or quinupramine or zimeldine or fluoxetine or citalopram or paroxetine or sertraline or alaproclate or fluvoxamine or etoperidone or escitalopram or isocarboxazid or nialamide or phenelzine or tranylcypromine or iproniazide or iproclozide or moclobemide or toloxatone or oxitriptan or tryptophan or mianserin or nomifensine or trazodone or nefazodone or minaprine or bifemelane or viloxazine or oxaflozane or mirtazapine or bupropion or medifoxamine or tianeptine or pivagabine or venlafaxine or milnacipran or reboxetine or gepirone or duloxetine or agomelatine or desvenlafaxine or vilazodone).tw.

6. 1 or 2 or 3 or 4 or 5

7. exp stillbirth/

8. exp perinatal mortality/

9. exp perinatal death/

10. exp fetus death/

11. (stillbirth or stillborn or perinatal death or peri-natal death or neonatal death or intrauterine death or intra-uterine death or newborn mortality).tw.

12. exp spontaneous abortion/
13. recurrent abortion/
14. (Spontaneous abortion or Miscarriage$).tw. 15. 7 or 8 or 9 or 10 or 11
16. 12 or 13 or 14
17. 6 and 15
18. 6 and 16
19. 17 or 18

S2 Table. Excluded studies

Study excluded at full-text screening	Reason
Lennestal et al. (2007)	Stillbirth outcome only
Sjaarda et al. (2020)	Combined outcome of both stillbirth and miscarriage
Stephansson et al. (2013)	Stillbirth outcome only
Stephens et al. (2011)	Wrong study design
Wen et al. (2006)	Stillbirth outcome only
Winterfeld et al. (2015)	Combined outcome of both stillbirth and miscarriage
Pastuszak et al. (1993)	No outcome data
Ramio et al. (2007)	Wrong study design
Robinson-Wolrath et al. (2016)	No outcome data
Sjaarda et al. (2019)	Duplicate
Andersen et al. (2013)	Duplicate
Burian et al. (2016)	No outcome data
Einarson et al. (2011)	No comparator
Einarson et al. (2009)	Duplicate
Jordan et al. (2016)	Wrong outcome
Einarson et al. (2011)	No comparator
Evans-Hoeker et al. (2017)	Duplicate
Klieger-Grossman et al. (2010)	Duplicate
Goldstein et al. (1997)	No comparator
Hoog et al. (2013)	No comparator
Einarson et al. (2012)	No outcome data
Innocenti et al. (2019)	No outcome data
Jimenez-Solem et al. (2014)	Stillbirth outcome only
Jimenez-Solem et al. (2013)	Stillbirth outcome only
Ericson et al. (1999)	Stillbirth outcome only

For peer review only

Table S3. Characteristics of included studies

Study	Study design	Country of study & study period	Data sampling methods	Type of AD exposure	Timing of AD exposure	Definition of exposure and comparator groups	Identification of exposure	Identification of outcome	Sample size	Confounding adjustments	Raw data (no. of SA/ total)	Adjusted estimates
Chambers et al. (1996) [1]	Prospective cohort	California 1989-1995	TIS data collection	Fluoxetine (SSRI)	Anytime	**Exposed:** Fluoxetine use **Comparator group 1:** women enquiring about non-teratogens	Telephone interviews	Telephone interview and confirmed by physician	482	Maternal age, average dose of fluoxetine	**Exposed early =** 23/169 **Control group** = 22/254	NA
Kulin et al. (1998) [2]	Prospective cohort	US&Canada	TIS data	SSRI	First trimester	**Exposed:** SSRI use during 1st trimester **Comparator group 1:** Women enquiring about non-teratogens	Telephone interview	After expected date of delivery women were interviewed	534	None	**Exposed** = 30/267 **Control =** 21/267	NA
Einarson et al. (2001) [3]	Cohort	European and non-european countries	Women contacting motherisk program around exposure advice	Venlafaxine (SNRI)	Anytime	**Exposed:** Venlafaxine use during 4th-14th week of pregnancy **Comparator group 1:** SSRI users **Comparator group 2:** women making non-teratogenic enquiries	Once contacted the service women were interviewed	Follow-up questionnaire after delivery and information verified by physician	450	None	**Venlafaxine =** 18/150 **SSRI comparator** = 16/150 **Nonteratogenic comparator =** 11/150	NA
Einarson et al. (2003)[4]	Prospective cohort	European and non-european countries	Women contacting motherisk program	Trazodone and Nefazodone (NaSSA)	First trimester	**Exposed:** Use of NaSSAs during 4th-14th week **Comparator group 1:** Women taking other ADs **Comparator group 2**: non-teratogen drug exposures	Questionnaire	Follow-up questionnaire after delivery and verified by physician	441	Matched on gestational age	**Trazodone/Nefazodone =** 20/147 **Group 1 (other Ads) =** 17/147 **Group 2 – nonteratogens** = 12/147	NA
Sivojelezova et al. (2005) [5]	Prospective cohort	Toronto 1999-2002	Women contacting motherisk program	Citalopram (SSRI)	Anytime	**Exposed:** Use of citalopram **Comparator group 1:** pregnant women taking other SSRI Ads **Comparator group 2:** non-teratogenic drug use	Telephone interview to individuals contacting the service	Follow-up questionnaire after delivery and verified by physician	396	None	**Exposed** = 14/132 **Comparison 1 =** 13/132 **Comparison 2 =** 13/132	NA
Chun-Fai Chan et al. (2005) [6]	Cohort	European and non-european countries	Individuals contacting motherisk or recruited through drug safety research unit	Bupropion (NDRI)	Anytime	**Exposed:** Bupropion exposed pregnancies **Comparator group 1:** Pregnant women taking other Ads **Comparator group 2:** individuals taking non-teratogens	Telephone administration of follow-up questionnaire	Follow-up questionnaire after delivery and verified by physician	NA	Matched on age, alcohol consumption, smoking and gestational age	**Bupropion =** 20/136 **Comparison 1 (other Ads) =** 11/89 **Comparison 2 (nontertaogens) =** 6/133 **Women exposed to bupropion for**	NA

For peer review only

											**depression =** 14/91	
Djulus et al. (2006) [7]	Cohort	European and non-european countries 2002-2005	Pregnant women contacting one of five evidence based information centres	Mirtazapine (NaSSA)	Anytime	**Exposed:** pregnant women taking bupropion **Comparator group 1:** disease matched individuals taking other Ads **Comparator group 2:** individuals taking known non-teratogens	Telephone interview regarding exposure	Contacted 2-6 months after delivery and details verified by physician	312	Matched on maternal age, gestational age, tobacco use, alcohol consumption and chronic conditions	**Mirtazapine =** 20/104 **Comparison 1 (other AD) =** 18/104 **Comparison 2 (nonteratogenics)** = 11/104	NA
Einarson et al. (2009) [8]	Prospective cohort	Toronto	women contacting motherisk program	Any AD	Anytime	**Exposed:** Pregnant women contacting program regarding exposure during pregnancy **Comparator group 1:** Women exposed to non-teratogenic drugs **Comparator group 2:** unexposed to Ads	Telephone interview using standardised questionnaire	Follow-up structured form	1874	Matched for maternal age, smoking, and alcohol use, gestational age	**Exposed =** 122/937 **Unexposed =** 75/937	RR = 1.38 (0.98-1.94)
Nakhai-Pour et al. (2010) [9]	Nested case-control	Quebec 1998-2003	Data from Quebec pregnancy register with individuals registered between 1998-2003, including RAMQ database	Any AD	Any time between first day gestation and index date of SA	**Exposed:** At least one prescription filled between start of gestation and SA index date 10 controls sampled for every SA case	RAMQ database which collects information on prescriptions, and pregnancy register to identify unexposed	Quebec pregnancy register entered between 1998-2003	56364	Adjusted for age, urban residence, social assistance, psychiatric diagnosis, comorbid illness, medication use, gestational age	**Cases =** 284 individuals used antidepressants **Controls =** 1401 used antidepressants	**Crude OR =** 2.09 (1.83-2.38) **Adjusted OR =** 1.68 (1.38-2.06) **Adjusted SSRI alone =** 1.61 (1.28-2.04) **Adjusted TCA alone =** 1.27 (0.85-1.91)
Paulus et al. (2010) [10]	Prospective cohort: Conference abstract	Germany	National TIS who were contacted by physicians or patients	Paroxetine (SSRI)	First trimester	**Exposed:** Paroxetine exposure in first trimester **Comparator group 1:** Not exposed or 'severely exposed' to paroxetine	Patients or physicians contact TIS	NA	955	None	**Exposed =** 23/202 **Unexposed =** 81/714	NA
Chan et al. (2011) [11]	Cohort – conference abstract	US 2000-2008	Recruited from 4 US metropolitan areas	All Ads	Anytime	**Exposed:** Antidepressant use during pregnancy **Comparator:** NA	Telephone interviews and medical record abstractions	Telephone interviews and medical record abstractions	4536	Maternal age, race, education, BMI, smoking, alcohol use, gravidity and nausea	NA	**Adjusted OR of exposed vs unexposed =** 1.54 (1.1-2.2) **Adjusted OR of SSRI users vs unexposed =** 1.67

For peer review only

Einarson et al. (2011) [12]	Prospective cohort	Toronto 1992-2007	women contacting motherisk	All Ads	Anytime	**Exposed:** exposed to more than one AD during pregnancy<br>**Comparator group 1:** Exposed to single AD during pregnancy<br>**Comparator group 2:** contacting motherisk regarding non-teratogenic exposure	telephone interview using standardised questionnaire	Follow-up interview details verified from report by physician	267	Matched on maternal age, smoking and alcohol use and gestational age	**Multiple AD exposure** = 8/89<br>**Single AD exposure** = 7/89<br>**No AD exposure** = 8/89	**OR for multiple AD exposure vs single exposure** = 1.16 (0.4-3.34)<br>**OR for multiple AD exposure vs no exposure** = 1.00 (0.36-2.79)
Ban et al. (2012) [13]	Cohort	UK 1990-2009	Data collected from primary care medical records (through health improvement network - THIN)	TCAs and SSRIs	First trimester	**Exposed:** depression and exposed to AD during first 90 days after conception<br>**Comparator group 1L** No history of depression and unexposed<br>**Comparator group 2:** history of depression and unexposed<br>**Comparator group 3:** prescriptions for TCAs only<br>**Comparator group 4:** prescriptions for SSRIs only<br>**Comparator group 5:** prescriptions for any other AD only<br>**Comparator group 6:** prescription for psychotropic drugs only	Extracted prescription records from THIN	Identified through THIN database	331414	Adjusted for maternal age, SES, maternal smoking status and BMI	**Reference category (group 1)** = 47258/390665<br>**History of mental illness only** = 13814/99932<br>**Unmedicated current mental illness** = 442/3647<br>**TCAs** = 443/3019<br>**SSRIs**= 1539/10132	**<u>Adjusted RRR with comparison being no current/past depression or anxiety</u>**<br>**History of mental illness only:** 1.2 (1.2-1.2)<br>**Unmedicated mental illness** = 1.0 (0.9-1.2)<br>**TCAs** = 1.3 (1.1-1.5)<br>**SSRIs** = 1.5 (1.3-1.6)<br><br>**<u>Adjusted RRR when compared to unmedicated antenatal depression or anxiety</u>**<br>**TCAs** = 1.3 (1.1-1.5)<br>**SSRIs** = 1.4 (1.2-1.7)
Klieger-Grossman et al. (2012) [14]	Prospective cohort	Toronto, Switzerland and Florence	Data collected from women ringing TIS in the three countries	Escitalopram (SSRI)	Anytime	**Exposed:** pregnant women exposed to escitalopram<br>**Comparator group 1:** contacting service exposed to other Ads<br>**Comparator group 2:** nonteratogenic exposures	Information taken during telephone conversation through standardised questionnaire	Follow-up questionnaire 2-3 months after delivery	637	Matched on maternal age, alcohol consumption, smoking and gestational age	**Escitalopram** = 32/213<br>**Other AD use** = 34/212<br>**Nonteratogens** = 18/212	NA
Kjaesgaard et al. (2013) [15]	Population based cohort	Denmark 1997-2008	Data obtained from Danish health registries	All Ads	Anytime	**Exposed:** mother redeemed prescription for AD<br>**Comparator group 2:** mother hadn't redeemed prescription	Information from registry of medicinal product statistics	Identified through codes using Danish national hospital registry	1005319	Adjusted for maternal age, cohabitation, income, education, history of mental	**Diagnosis of depression and exposed to AD** = 210/1674<br>**Diagnosis of depression and unexposed to AD**= 105/820<br>**No diagnosis of**	**<u>Unadjusted RR when compared to unexposed equivalent:</u>**<br>**Diagnosis of depression exposed to AD** = 0.98 (0.78-1.23)<br>**No diagnosis of**

For peer review only

										disorder or drug abuse	**depression and AD use=** 2427/13789<br>**No diagnosis of depression and unexposed=** 110377/818426	**depression and AD use =** 1.31 (1.26-1.35)<br><br>**Adjusted RR (when compared to unexposed equivalent):**<br>**Diagnosis of depression and exposed =** 1.00 (0.8-1.24)<br>**No diagnosis of depression and AD use =** 1.17 (1.13-1.22)
Andersen et al. (2014)[16]	Cohort	Denmark 1997-2010	Identified all registered pregnancies through national hospital register	SSRIs	First 35 days of pregnancy	**Exposed:** Dispensing of prescription of SSRIs<br>**Comparator group 1:** Women exposed to SSRIs prior to pregnancy<br>**Comparator group 2:** women unexposed to SSRIs	Data collected from national prescription register containing individual level data on prescribed drugs	Identified through national hospital register using codes	1279840	Adjusted for maternal age, previous SA, income, year of outcome, educational length, antipsychotics and thyroid preparations	**Unexposed=** 139201/1256956<br>**Exposed to SSRI during first 35 days of pregnancy:** 2883/22884<br>**Discontinuation of SSRI treatment 3-12 months before conception =** 1936/14016	**Unadjusted HR:**<br>**Exposed during first 35 days vs unexposed:** 1.31 (1.27-1.36)<br>**Discontinued SSRI treatment 3-12 months before pregnancy vs unexposed:** 1.38 (1.32-1.44)<br><br>**Adjusted HR:**<br>**Exposed to SSRI during first 35 days vs unexposed:** 1.27(1.22-1.33)<br>**Discontinued use of SSRI before conception vs unexposed:** 1.24 (1.18-1.30)
Abadie et al. (2015)[17]	Nested case-control	France	French database of TERAPPEL used where healthcare professionals contact French regional pharmacovigilance centres (CPRV)	All Ads	First trimester	**Exposed:** First trimester drug exposure and called CPRV enquiring about drug exposure<br>**Comparator group 1:** Calling CPRV for assessing non-teratogen exposure	Healthcare professionals contacting CPRVs	Women registered to TERAPPEL database	5346	Maternal age	**Exposed to any AD**<br>**Cases of SA =** 170/838<br>**No SA =** 729/4508<br><br>**Exposed to non-selective monoamine reuptake inhibitors (NMRI)**<br>**SA =** 39/838<br>**No SA =** 88/4508	**Crude OR for all Ads =** 1.32 (1.1-1.59)<br>**Adjusted OR for all Ads =** 1.2 (0.98-1.46)<br><br>**Crude OR for NMRI=** 2.45 (1.67-3.60)<br>**Adjusted OR for NMRI =** 2.19 (1.46-2.39)

For peer review only

Johansen et al. (2015) [18]	Register based study cohort	Denmark 1996-2009	study population with registered pregnancies between 1996-2009 and Danish national birth cohort	SSRI	Anytime but results split into first and second trimester	**Exposed:** Redeemed prescription in time frame of month prior to LMP to 3 months after LMP or end of pregnancy (also self-report)<br>**Comparator group 1:** discontinued use of SSRIs with same LMP prescription timeframe<br>**Comparator group 2:** unexposed and no prescription	Redeemed prescriptions from Danish national prescription database	Data from Danish national patient register	1281418	Adjustment for household income, education, previous SA, parity and maternal age	**Unexposed** = 124333/1165124<br>**Discontinued use** = 670/5428<br>**Exposed** = 2359/20612	**Combined first and second trimester HR**<br><br>**Exposed vs unexposed general population =** 1.07 (1.03-1.12)<br>**SSRI use vs unmedicated depressed =** 0.97 (0.81-1.16)
Te Winkel et al. (2016) [19]	Prospective cohort – conference abstract only	Across European countries	Data from nine centres which make up european network of TIS	Venlafaxine (SSRI)	Anytime	**Exposed:** Venlafaxine exposure<br>**Comparator group 1:** No exposure to any known teratogens	TIS data	using standardised procedures for data collection and follow-up	1462	None	**Exposed =** 85/732<br>**Unexposed =** 46/730	NA
Almeida et al. (2016) [20]	Retrospective cohort	Quebec 1998-2002	Linking four population-based registries and producing longitudinal histories of medication use	All Ads	First trimester	**Exposed:** Women who have at least one AD prescription in first trimester of pregnancy<br>**Comparator groups 1:** Unexposed but depressed in last four years<br>**Comparator group 2:** non-teratogen group<br>**Comparator group 3:** individuals with no prescriptions or depression	Prescription claims database	ICD-9 codes	41964	Adjustment for maternal age, welfare recipient, other teratogenic medication, no. medications any hospitalisation or physician visits, calendar year	**No medication and no depression:** 1685/28343<br>**No AD but depression -** 720/10719<br>**AD use in first trimester and depression -** 165/1955<br>**Hypothyroid medication use in first trimester:** 71/947	**All adjusted RRR**<br><br>**Unexposed and no depression =** 1.00<br>**Unexposed and depressed =** 1.1 (1.0-1.2)<br>**Exposed and depressed =** 1.3 (1.1-1.5)
Evans-Hoeker et al. (2018) [21]	Cohort	US	RCT was conducted on non-IVF fertility treatment and individuals from trial were brought into cohort study	All Ads – split into SSRI and non-SSRI	Anytime	**Group 1:** Absence of major depression(MD) and no AD use<br>**Group 2:** Absence of MD and using AD<br>**Group 3:** Presence of MD and no AD use<br>**Group 4:** MD & using Ads	Medication exposure assessed through RCT and PH-9 questionnaire used	Identification through RCT	1650	Adjusted for age, race, income, current smoking, depression severity and trial placed in for RCT	**PH-9<10 and unexposed =** 110/1359<br>**PH-9<10 and exposed** = 14/73<br>**PH-9>10 and unexposed** = 5/72<br>**PH-9>10 and exposed** = 2/16<br><br>**No AD use** = 118/1484<br>**Any AD use** = 16/90	**Adjusted RR:**<br>**Ph-9<10 and unexposed =** reference<br>**PH-9<10 and exposed** = 1.87 (1.18-2.99)<br>**PH-9>10 and unexposed =** 0.85 (0.37-1.96)<br>**PH-9>10 and exposed** = 1.4 (0.47-4.17)<br><br>**specific medication types:**

For peer review only

											**SSRI use** = 11/57<br>**Non-SSRI use**= 4/25<br>**Both use of SSRI and non-SSRI** = 1/8	**Any AD use** = 1.92 (1.22-3.02)<br>**SSRI use** = 1.73 (1.0-3.0)<br>**Non-SSRI use**= 3.45(1.99-5.98)<br>**Both SSRI and non-SSRI** = 1.15 (0.19-7.06)
Richardson et al. (2019) [22]	Cohort	UK	Using UK collected teratogen surveillance data	Venlafaxine (SNRI)	Anytime	**Exposed:** Individuals using venlafaxine at any point during pregnancy<br>**Comparator group 1:** Unexposed to any AD medication (5:1)<br>**Comparator group 2:** disease matched comparator of SSRI AD exposed	Surveillance data from UKTIS	Postal questionnaire sent after delivery date from healthcare professional who contacted UKTIS	2529	Adjusted for history of SA, tobacco exposure, alcohol, recreational drugs and folic acid	**Venlafaxine** = 46/281<br>**SSRI exposed** = 114/843<br>**Unexposed** = 140/1405	**Crude OR for unexposed** = 1.57 (1.06-2.30)<br>**Crude OR for SSRI comparator** = 1.05 (0.7-1.57)<br><br>**Adjusted HR for unexposed** = 1.26 (0.829-1.91)<br>**Adjusted HR for SSRI comparator** = 1.00 (0.655-1.53)
Wu et al. (2019) [23]	Prospective cohort	North Carolina, Tennessee and Texas	Enrolment into community-based cohort	All AD – classified into SSRI and non-SSRI	First trimester	**Exposed:** reported AD use since pregnancy and before interview<br>**Comparator group 1:** Never used AD	community-based cohort – AD use assessed during interview	Self-reported pregnancy outcomes-verified by medical records	5451	Adjustment for maternal age, BMI, caffeine consumption, smoking, alcohol, race, SA history	**Exposed to any AD** = 42/223<br>**Use of SSRIs** = 35/179<br>**Exposure to non-SSRIs** = 7/44<br>**Unexposed** = 617/5228	**Unadjusted HR:**<br><br>**Any AD** = 1.51 (1.10-2.06)<br>**Only SSRIs** = 1.59 (1.13-2.23)<br>**Only non-SSRIs** = **1.21** (0.57-2.56)<br><br>**Adjusted HR**<br>**Any AD use:** 1.34 (0.97-1.85)<br>**SSRI only:** 1.45 (1.02-2.06)<br>**Non-SSRI only:** 0.96 (0.45-2.04)
Bahat et al. (2020) [24]	Cohort – conference abstract only	Israel 2001-2015	Data utilised from Israeli TIS	Duloxetine (SNRI)	First trimester	**Exposed:** Exposure to duloxetine during first trimester<br>**Comparator group 1:** women exposed to TCAs<br>**Comparator group 2:** non-teratogenic exposure	Information taken when contacted TIS	Identified through follow-up after delivery date	722	None	NA	**Adjusted HR** = 1.89 (1.04-3.43)

For peer review only

Kolding et al. (2021)[25]	Register based study	Denmark 2007-2014	Routinely collected data from five nationwide registries and databases	All Ads	First trimester	**Exposed:** two or more redeemed prescriptions<br>**Comparator group 1:** Absence of redemptions for AD	Using redeemed prescriptions through linkage to Danish health services prescription database	Identified through Danish national patient registry	364012	None	**Exposed =** 134/3971<br>**Unexposed =** 10739/342842	NA
Ankarfeldt et al. (2021)[26]	Prospective cohort	Denmark 2004-2016	Study population used danish registry data whereby women were included if they had a recorded birth in birth register or an abortion in patient register between 2004-2016	Duloxetine (SNRI)<br>SSRIs<br>Venlafaxine (SNRI)	Anytime during pregnancy	**Exposed** = redemption of AD prescription in window from 30 days prior to LMP to 140 days post LMP or end of pregnancy, whichever came first<br>**Comparator group 1:** Duloxetine non-exposed (no redemption of prescription during the window)<br>**Comparator group 2:** SSRI exposed<br>**Comparator group 3:** Venlafaxine exposed	Using the Danish national prescription register	Identified through either the birth registry for all births, or the patient register for abortions.	1020288	birth year, maternal age, psychiatric hospitalization, household income, education, comorbidities, co-medication	**Unadjusted:**<br>**Duloxetine =** 145/1212<br>**Duloxetine unexposed =** 106309/1018745<br>**SSRI =** 2900/28345<br>**Venlafaxine =** 497/4908	**Adjusted**<br><br>**Duloxetine vs unexposed** = 1.14 (0.96-1.34)<br>**Duloxetine vs SSRI =** 1.23 (1.04-1.46)<br>**Duloxetine vs venlafaxine =** 1.18 (0.98-1.42)<br><br>**PS-matched**<br><br>**duloxetine vs unexposed** = 1.08 (0.89-1.31)<br>**Duloxetine vs SSRI =** 1.25 (1.00-1.57)<br>**Duloxetine vs venlafaxine =** 1.08(0.82-1.41)

1

Ads=antidepressants, SSRIs = selective serotonin reuptake inhibitors, SNRIs= serotonin-norepinephrine reuptake inhibitors, TCAs= tricyclic antidepressants, NDRI = norepinephrine and dopamine reuptake inhibitors, NaSSA = noradrenergic and serotonergic specific antidepressants, RCT= randomized controlled trial, TIS=teratogen information service, NA= not available, MD = major depression

For peer review only - http://bmjopen.bmj.com/site/about/guidelines.xhtml

References

1. Chambers CD, Johnson KA, Dick LM, Felix RJ, Jones KL. Birth outcomes in pregnant women taking fluoxetine. *N Engl J Med* 1996; **335**(14): 1010-5.
2. Kulin NA, Pastuszak A, Sage SR, et al. Pregnancy outcome following maternal use of the new selective serotonin reuptake inhibitors: a prospective controlled multicenter study. *Jama* 1998; **279**(8): 609-10.
3. Einarson A, Fatoye B, Sarkar M, et al. Pregnancy outcome following gestational exposure to venlafaxine: a multicenter prospective controlled study. *Am J Psychiatry* 2001; **158**(10): 1728-30.
4. Einarson A, Bonari L, Voyer-Lavigne S, et al. A multicentre prospective controlled study to determine the safety of trazodone and nefazodone use during pregnancy. *Canadian Journal of Psychiatry* 2003; **48**(2): 106-10.
5. Sivojelezova A, Shuhaiber S, Sarkissian L, Einarson A, Koren G. Citalopram use in pregnancy: Prospective comparative evaluation of pregnancy and fetal outcome. *American Journal of Obstetrics and Gynecology* 2005; **193**(6): 2004-9.
6. Chun-Fai-Chan B, Koren G, Fayez I, et al. Pregnancy outcome of women exposed to bupropion during pregnancy: a prospective comparative study. *Am J Obstet Gynecol* 2005; **192**(3): 932-6.
7. Djulus J, Koren G, Einarson TR, et al. Exposure to mirtazapine during pregnancy: A prospective, comparative study of birth outcomes. *J Clin Psychiatry* 2006; **67**(8): 1280-4.
8. Einarson A, Choi J, Einarson TR, Koren G. Rates of spontaneous and therapeutic abortions following use of antidepressants in pregnancy: results from a large prospective database. *J Obstet Gynaecol Can* 2009; **31**(5): 452-6.
9. Nakhai-Pour HR, Broy P, Berard A. Use of antidepressants during pregnancy and the risk of spontaneous abortion. *Cmaj* 2010; **182**(10): 1031-7.
10. Paulus W, Schloemp S, Stoz F. Paroxetine exposure during pregnancy: Increased risk of congenital malformations? *Archives of Gynecology and Obstetrics* 2010; **282**: S79.
11. Chan RL, Jonsson Funk ML, Savitz DA, Hartmann KE. Self-reported antidepressants use and spontaneous abortion. *American Journal of Epidemiology* 2011; **173**: S140.
12. Einarson A, Choi J, Koren G, Einarson T. Outcomes of infants exposed to multiple antidepressants during pregnancy: results of a cohort study. *J Popul Ther Clin Pharmacol* 2011; **18**(2): e390-6.
13. Ban L, Tata LJ, West J, Fiaschi L, Gibson JE. Live and non-live pregnancy outcomes among women with depression and anxiety: a population-based study. *PLoS ONE* 2012; **7**(8): e43462.
14. Klieger-Grossmann C, Weitzner B, Panchaud A, et al. Pregnancy outcomes following use of escitalopram: a prospective comparative cohort study. *J Clin Pharmacol* 2012; **52**(5): 766-70.

For peer review only

15. Kjaersgaard MI, Parner ET, Vestergaard M, et al. Prenatal antidepressant exposure and risk of spontaneous abortion - a population-based study. *PLoS ONE* 2013; **8**(8): e72095.
16. Andersen JT, Andersen NL, Horwitz H, Poulsen HE, Jimenez-Solem E. Exposure to selective serotonin reuptake inhibitors in early pregnancy and the risk of miscarriage. *Obstet Gynecol* 2014; **124**(4): 655-61.
17. Abadie D, Hurault-Delarue C, Damase-Michel C, Montastruc JL, Lacroix I. Medication exposure and spontaneous abortion: A case-control study using a French medical database. *Clinical and Experimental Obstetrics and Gynecology* 2015; **42**(4): 431-6.
18. Johansen RL, Mortensen LH, Andersen AM, Hansen AV, Strandberg-Larsen K. Maternal use of selective serotonin reuptake inhibitors and risk of miscarriage - assessing potential biases. *Paediatr Perinat Epidemiol* 2015; **29**(1): 72-81.
19. Te Winkel B, Beghin D, Pistelli A, et al. Venlafaxine exposure in pregnancy, a multicenter ENTIS study. *Pharmacoepidemiology and Drug Safety* 2016; **25 (Supplement 3)**: 325-6.
20. Almeida ND, Basso O, Abrahamowicz M, Gagnon R, Tamblyn R. Risk of Miscarriage in Women Receiving Antidepressants in Early Pregnancy, Correcting for Induced Abortions. *Epidemiology* 2016; **27**(4): 538-46.
21. Evans-Hoeker EA, Eisenberg E, Diamond MP, et al. Major depression, antidepressant use, and male and female fertility. *Fertility and Sterility* 2018; **109**(5): 879-87.
22. Richardson JL, Martin F, Dunstan H, et al. Pregnancy outcomes following maternal venlafaxine use: A prospective observational comparative cohort study. *Reprod Toxicol* 2019; **84**: 108-13.
23. Wu P, Velez Edwards DR, Gorrindo P, et al. Association between First Trimester Antidepressant Use and Risk of Spontaneous Abortion. *Pharmacotherapy* 2019; **39**(9): 889-98.
24. Bahat M, Shechtman S, Diav-Citrin O. Pregnancy outcome after first trimester exposure to Duloxetine. *Reprod Toxicol* 2020; **97**: 3.
25. Kolding L, Ehrenstein V, Pedersen L, et al. Antidepressant use in pregnancy and severe cardiac malformations: Danish register-based study. *Bjog* 2021; **128**(12): 1949-57.
26. Ankarfeldt MZ, Petersen J, Andersen JT, et al. Duloxetine Exposure During Pregnancy and the Risk of Spontaneous and Elective Abortion: A Danish Nationwide Observational Study. *Drugs Real World Outcomes* 2021; **8**(3): 289-99.

For peer review only

S4 Table. Meta-analysis results

Analysis type		Number of studies	Number of adjusted studies used	Summary effect estimate (95% confidence interval)	Heterogeneity
**Main analysis**					
**Studies in the general population**	AD use vs unexposed comparator	26	15	1.29 (1.21-1.38)	70.80%
	SSRI vs unexposed comparator	11	5	1.32 (1.16-1.50)	83.30%
	SNRI vs unexposed comparator	5	2	1.46 (1.12-1.91)	55.20%
**Studies restricting population to women with depression**	AD use vs unmedicated depressed comparator	5	3	1.16 (0.98-1.36)	67.70%
	SSRI v another AD	5	1	1.04 (0.93-1.16)	0%
	SNRI vs another AD	3	2	1.19 (1.02-1.39)	0%
**Sensitivity analysis restricting to studies of sufficient quality**					
**Studies in the general population**	AD use vs unexposed comparator	21	15	1.30 (1.21-1.39)	74.10%
	SSRI vs unexposed comparator	8	5	1.32 (1.15-1.52)	87.90%
	SNRI vs unexposed comparator	4	2	1.39 (1.07-1.80)	39.80%

S5 Table. Risk of bias assessment

Study	Domain 1: Bias due to confounding	Domain 2: Bias in classification of intervention	Domain 3: Bias in selection of participants into study	Domain 4: Bias due to deviations from intended interventions	Domain 5: Bias due to missing data	Domain 6: Bias in measurement of the outcome	Domain 7: Bias in selection of the reported result	Overall RoB rating
Chambers et al (1996)	**Critical** No confounding adjustments made	**Critical**	**Critical**	**Critical**	**Critical**	**Critical**	**Critical**	**Critical**
Kulin et al (1998)	**Critical** No information on confounding adjustments	**Critical**	**Critical**	**Critical**	**Critical**	**Critical**	**Critical**	**Critical**
Einarson et al (2001)	**Serious** Justification: The paper only adjusted for maternal depression, therefore not adjusting for all three of the key confounding domains	**Serious** Justification: Exposure was determined through self-report of the individual. This was measured prospectively whereby the outcome had not occurred so would be unlikely to affect the exposure classification, however this was not verified through physician or healthcare records. Therefore, intervention status could not have been classified correctly and could be differential	**Serious:** Justification: The start of follow-up and intervention are not at the same point as women ringing the service enquiring about an exposure could have been taking it for weeks prior to call, which could induce bias as the intervention has differing effects over time, and no analytical techniques were put in place to minimise this bias	**Low** Justification: Study was not undergone in an experimental context	**Serious** Justification: Although complete case analysis was used, the number of exposed individuals and total sample was not stated. Therefore, there could have been missing data and there was no evidence to show that the missing data of outcome was not associated with the true value of the outcome	**Low** Justification: The outcome was measured through questionnaire to individual, alongside the results being verified by a physician. Although the outcome assessors could have been aware of exposure status, this is unlikely to have influenced outcome classification.	**Moderate** Justification: No statistical analysis plan was provided, however, all analysis that was stated in methods was completed and results given	**Serious**
Einarson et al (2003)	**Serious**	**Serious**	**Moderate**	**Low**	**Serious**	**Low**	**Moderate**	**Serious**

	Justification: Only indication for treatment and individual characteristics domain was adjusted for, meaning lifestyle exposures was not adjusted for resulting in serious risk of bias	Justification: Exposure was determined through self-report of the individual, however this was not verified through physician or healthcare records	Justification: Although start of intervention and follow-up were unlikely at the same time point, the study restricted to exposure during the first trimester only, therefore the differing effects of intervention across different time periods potentially inducing bias is minimised as restricted to first trimester	Justification: Study was not undergone in an experimental context	Justification: Although complete case analysis was utilised, the total sample of how many exposed individuals was not mentioned, only the number included in a complete case analysis. Therefore, there will likely be missing data, and as the outcome was determined through self-report there is no evidence that the missingness of the outcome was not related to its true value.	Justification: The outcome was determined through self-report questionnaire, however this was then confirmed through the individuals physician. Although the outcome assessors could have been aware of exposure status, this was unlikely to have influenced the reporting of the outcome	Justification: No statistical analysis plan was provided, however, all analysis that was stated in methods was completed and results given	
Sivojelezova et al (2005)	**Serious** Justification: Although presence of depression and maternal age was adjusted for, there was no factor that was adjusted for in lifestyle exposures. Therefore, if not all three of the	**Moderate** Justification: Self-report of exposure through a standardised intake form. In addition, letter sent to physician to confirm the information given by individual	**Serious** Justification: The start of intervention and follow-up were likely not at the same time point as women just rung up enquiring about exposure, in addition, no analysis methods were used to account for the differing effects of	**Low** Justification: Study was not undergone in an experimental context	**Serious** Justification: Although complete case analysis was used, the number of exposed individuals and total sample was not stated. Therefore, there could have been missing data and there was no	**Low** Justification: The outcome was measured through a questionnaire which is not the most reliable, however, the questionnaire was also sent to healthcare professional to confirm. Therefore, the healthcare professional would	**Moderate** Justification: No statistical analysis plan was given	**Serious**

	confounding domains are adjusted for it means serious rating		intervention across different time points		evidence to show that the missing data in the outcome was not associated with the the true value of the outcome.	likely know the exposure status of		
Chun-Fai-Chan et al (2005)	**Moderate**<br>Justification: All three confounding domains of indication for treatment, lifestyle exposures and individual characteristics were adjusted for	**Moderate**<br>Justification: Exposure was classified through self-report in questionnaire, however this was verified through a physician. Additionally, there was a follow-up questionnaire. As exposure was measured prospectively there was no risk of outcome influencing reporting of exposure	**Serious**<br>Justification: The start of follow-up and intervention are not at the same point, which could induce bias as the intervention has differing effects over time, and no analytical techniques were put in place to minimise this bias	**Low**<br>Justification: Study was not undergone in an experimental context	**Serious**<br>Justification: Although complete case analysis was used, the number of exposed individuals and total sample was not stated. Therefore, there could have been missing data and there was no evidence to show that the missing data was not associated with the true value of the outcome. This is particularly relevant considering the outcome was individual self report.	**Low**<br>Justification: outcome was measured through telephone conversation and then. Verified with physician. Although the outcome assessors could have been aware of exposure status	**Moderate**<br>Justification: No statistical analysis plan was provided, however, all analysis that was stated in methods was completed and results given	**Serious**
Djulus et al (2006)	**Moderate**<br>Justification: All three	**Moderate**<br>Justification: Women contacted	**Serious**<br>Justification: The start of follow-up	**Low**<br>Justification: Study was not undergone	**Serious**<br>Justification: Complete case	**Low**<br>Justification: The outcome was	**Moderate**<br>Justification: No statistical analysis	**Serious**

For peer review only

	confounding factors of indication for treatment, individual characteristics and lifestyle exposures were adjusted for	the service regarding exposure and then details of exposure were taken including duration, timing and frequency - this questionnaire was then sent to practitioner for details to be confirmed	and intervention are not at the same point, which could induce bias as the intervention has differing effects over time, and no analytical techniques were put in place to minimise this bias	in an experimental context	analysis was used but there was no total sample size or flow diagram indicating recruitment of individuals and any exclusions. Missing data was not mentioned at all.	measured through questionnaire to individual, alongside the results being verified by a physician. Although the outcome assessors could have been aware of exposure status, this is unlikely to have influenced outcome classification.	plan was provided, however, all analysis that was stated in methods was completed and results given	
Einarson et al (2009)	**Serious** Justification: Although the domains of individual characteristics and lifestyle exposures were adjusted for through maternal age and smoking, indication for treatment domain was not adjusted for	**Serious** Justification: Exposure was determined through self-report of the individual, however this was not verified through physician or healthcare records	**Moderate** Justification: Although start of intervention and follow-up were unlikely at the same time point, the study restricted to exposure during the first trimester only, therefore the differing effects of intervention across different time periods potentially inducing bias is minimised as restricted to first trimester	**Low** Justification: Study was not undergone in an experimental context	**Serious** Justification: Although complete case analysis was utilised, the total sample of how many exposed individuals was not mentioned, only the number included in a complete case analysis. Therefore, there will likely be missing data, and as the outcome was determined through self-report there is no evidence that the missingness of the outcome was not related to its true value.	**Serious** Justification: The outcome was reported by the mother/individual, however this was not verified with their physician or healthcare records. Therefore the exposure status known by the individual could influence their reporting of outcome classification.	**Moderate** Justification: No statistical analysis plan was provided, however, all analysis that was stated in methods was completed and results given	**Serious**

For peer review only

Nakhai-Pour et al (2010)	**Serious** Justification: It did not adjust for a factor in all three of the key confounding domains, as it did not adjust for any factor of lifestyle exposure	**Moderate** Justification: The exposure classification was done through GP records and therefore all information to classify intervention was recorded at start of intervention. Extent of non-differential misclassification would not be large.	**Moderate** Justification: Matching was employed, as well as controls selected independently to intervention status. The cases and controls were matched on gestational age so same risk period. Therefore, not at serious risk of bias in this domain	**Low** Justification: Study was not undergone in an experimental context	**Low** Justification: Smoking and maternal weight covariates were the only variables which had missing data. The missingness of these confounding factors is unlikely to be related to the true value of the outcome because the outcome was determined through pregnancy register rather than self-report	**Low** Justification: The outcome was measured through the Quebec pregnancy register and therefore very objective and not influenced by assessors	**Serious** Justification: No statistical analysis plan was given, additionally lots of data collected on covariates however only some included in the adjustment model. Finally no results given for the sensitivity analysis.	**Serious**
Paulus et al (2010)	**Critical** No information given on any confounding adjustments done	**Critical**	**Critical**	**Critical**	**Critical**	**Critical**	**Critical**	**Critical**
Chan et al (2011)	**Serious** Justification: Only individual characteristics and lifestyle exposure domains had a factor adjusted for in them - however	**Serious** Justification: It was unclear the process of exposure classification, and indicated that only telephone interviews of self-report were used	**Moderate** Justification: Start of intervention and follow-up were not the same, however, the analysis used was logistic time survival models so the differing effect of ads of different time	**Low** Study was not undergone in an experimental context	**Serious** Justification: No information given on missing data.	**Low** Justification: Telephone interviews and medical records were used to determine the outcome, reliable method. Unclear whether outcome assessors were aware	**Moderate** Justification: No statistical analysis plan was provided, however, all analysis that was stated in methods was completed and results given	**Serious**

For peer review only

	indication for treatment was not adjusted for not adjusted for at all - therefore deemed serious as not all confounding domains satisfied.	for exposure classification.	periods was accounted for			of exposure status, however unlikely to have influenced outcome determination because outcome is very objective		
Einarson et al (2011)	**Moderate** Justification: All three confounding domains were adjusted for	**Serious** Justification: Exposure was determined through self-report of the individual, however this was not verified through physician or healthcare records	**Serious** Justification: The start of follow-up and intervention are not at the same point as women ringing the service enquiring about an exposure could have been taking it for weeks prior to call, which could induce bias as the intervention has differing effects over time, and no analytical techniques were put in place to minimise this bias	**Low** Study was not undergone in an experimental context	**Serious** Justification: Although complete case analysis was used, the number of exposed individuals/total sample was not stated. Therefore, there could have been missing data on participants that would have been excluded - this missing data could include outcome data which could be related to its true value as the outcome was self-report.	**Low** Justification: The outcome was determined through self-report questionnaire; however this was then confirmed through the individuals physician. Although the outcome assessors could have been aware of exposure status	**Moderate** Justification: No statistical analysis plan was provided, however, all analysis that was stated in methods was completed and results given	**Serious**
Ban et al (2012)	**Moderate** Justification: All three confounding domains were adjusted for	**Moderate** Justification: Exposure was classified through prescription databases where all	**Serious** Justification: Although prescription database was used, it is unlikely that the	**Low** Study was not undergone in an experimental context	**Low** Justification: Missing data on covariates found, and the missingness	**Low** Justification: Outcome was measured reliably through healthcare records, however unclear whether	**Moderate** Justification: Although there was no statistical analysis plan there were no	**Serious**

For peer review only

	through some factor - including presence of depression, maternal age and smoking	drug usage was recorded - however just small difficulty of unsure the true ingested drug usage	start of intervention and start of follow-up will be the same. Additionally, the authors did not use any statistical analysis including cox proportional regression to counteract		would be unrelated to the outcome because they were taken from NHS databases before the outcome occurred. Additionally, no missing outcome data was found	outcome assessors would know exposure status but unlikely this would have influenced classification	missing parts to the analysis.	
Klieger-Grossman et al (2012)	**Moderate** Justification: All three confounding domains were adjusted for through some factor - including presence of depression, maternal age and smoking	**Moderate** Justification: Initial interview questioning exposure, then a follow-up interview to confirm answers given in original questioning. This was all done prospectively so no risk of outcome influencing exposure reporting	**Serious** Justification: The start of intervention and follow-up would not have been the same, therefore inducing potential bias, additionally no analytical techniques were put in place to account for this bias either through time dependent analysis or restricting the analysis to first trimester only	**Low** Study was not undergone in an experimental context	**Serious** Justification: Although complete case analysis was used, the number of exposed individuals and total sample was not stated. Therefore, there could have been missing data and there was no evidence to show that the missing data of outcome was not associated with the true value of it	**Low** Justification: Assessment of the outcome won't be influenced by the exposure	**Moderate** Justification: No statistical analysis plan was provided, however, all analysis that was stated in methods was completed and results given	**Serious**
Kjaesgaard et al (2013)	**Serious** Justification: only two of the three confounding domains were adjusted for - as	**Moderate** Justification: Prescription records were used to determine exposure, therefore	**Serious** Justification: Unlikely the start of follow-up and intervention were at the same time point.	**Low** Study was not undergone in an experimental context	**Low** Justification: There was missing gestational age data, however	**Low** Justification: Outcome was assessed through codes on danish national hospital registry therefore very	**Moderate** Justification: No statistical analysis plan was provided, however, all analysis that was stated in	**Serious**

For peer review only

	the third domain of lifestyle exposures was not adjusted for	likely to classify individuals correctly, however if there is misclassification this will likely be non-differential and attenuate towards the null	Additionally, no analysis was utilized to account foe the differing effect of intervention at different points during pregnancy		this was replaced by the median of all non-missing values. Therefore covariate data is missing - as the outcome was determined through hospital registry it is unlikely that the true value of the outcome will influence the covariate	reliable method and outcome assessors unlikely aware of exposure status	methods was completed and results given	
Andersen et al (2014)	**Serious** Justification: No factor of lifestyle exposure was adjusted for in this analysis, only indication for treatment and individual characteristics was adjusted for	**Moderate** Justification: exposure was classified through prescription databases therefore unlikely to be any issues with classification of exposure - however slight issues with true ingested drug usage.	**Moderate** Justification: Prescription database was used meaning unclear whether start of intervention and start of follow-up were at the same time point. However, a cox proportional hazards regression was used which counteracts the differing effects that exposure will have at different time points during pregnancy.	**Low** Study was not undergone in an experimental context	**Low** Justification: Minimal missing data was found on the confounding factors used, additionally, outcome was measured through hospital records therefore unlikely that the true value of the outcome will be influencing the missingness. No missing outcome data.	**Moderate** Justification: Outcome was measured in a very objective and reliable method through hospital records, unclear whether outcome assessors knew exposure status however this is unlikely to influence exposure status determination. However, the method of outcome assessment could have differed between interventions as individuals on antidepressants would be more likely to come into hospital	**Moderate** Justification: No statistical plan was given, however all results that were mentioned in method were given	**Serious**

For peer review only

						as more contact with healthcare services compared to unexposed individuals.		
Abadie et al (2015)	**Serious** Justification: Only one of the three confounding domains had anything adjusted for - only maternal age had been accounted for - therefore not all three confounding domains satisfied and serious risk of bias	**Moderate** Justification: The CRPV was notified by healthcare professional regarding exposure, then a questionnaire was sent across and confirmation of current medication taken currently, so low risk of differential misclassification.	**Serious** Justification: The authors did employ matching, however the controls were not independently selected of the intervention status. Additionally, they didn't match on gestational age so time at risk would have differed between cases and controls	**Low** Study was not undergone in an experimental context	**Low** Justification: There was missing data on the confounding factors, as the outcome was measured through a database it is unlikely the missingness of covariates is related to true value of outcome. However, no missing data for the outcome was found.	**Low** Justification: The outcome was measured through database administrative records, therefore very reliable	**Moderate** Justification: Although there was no statistical analysis plan there were no missing parts to the analysis.	**Serious**
Johansen et al (2015)	**Serious** Justification: Although this paper did a large amount of adjustments, especially in relation to confounding by indication, which most other papers didn't, it still didn't adjust	**Moderate** Justification: Both prescription records and self-report was used to assess exposure classification for individuals, therefore very reliable method using both - it did find differences between the two	**Moderate** Justification: It is unlikely the start of intervention and start of follow-up were at the same point, however a cox proportional hazards regression was used to account for the differing effects of exposure at different	**Low** Study was not undergone in an experimental context	**Low** Justification: A flow diagram illustrating the total sample and any excluded individuals or missing data. This highlighted there was missing data in the exposure, covariates and outcome.	**Low** Justification: The outcome was assessed through healthcare registers and the outcome assessors would be unaware of exposure status therefore very little risk regarding exposure influencing outcome status, especially considering	**Serious** Justification: No statistical analysis plan was given, and information on confounders was taken which were not used in the adjustment	**Serious**

For peer review only - http://bmjopen.bmj.com/site/about/guidelines.xhtml

	for all three confounding domains as it missed adjusting for factors on lifestyle exposure	methods so likely there will be misclassification however very minimal	time points during pregnancy		However, as the outcome was assessed through healthcare registers and bypassing the mother its unlikely the true value of the outcome would cause this missingness.	the objective nature of the outcome		
Te winkel et al (2016)	**Critical** No information given on any confounding adjustments done	**Critical**	**Critical**	**Critical**	**Critical**	**Critical**	**Critical**	**Critical**
Almeida et al (2016)	**Serious** Justification: Only indication for treatment and individual characteristics domain had been adjusted for, whereas no factors were adjusted for in lifestyle exposures	**Moderate** Justification: exposure was classified through prescription database, therefore highly reliable. However, there may be some discrepancies as to the actual ingested drug usage.	**Serious** Justification: Although prescription database was used, it is unlikely that the start of intervention and start of follow-up will be the same. Additionally, the authors did not use any statistical analysis including cox proportional regression to counteract the differing effects that exposure will have at different time points during pregnancy.	**Low** Study wasn't undergone in an experimental context	**Low** Justification: There was missing data on the confounding factors, as the outcome was measured through the physician rather than self-report it is unlikely the true value of the outcome would be influencing the missingness of these covariates . However, no missing data for	**Low** Justification: Outcome was assessed through ICD-9 codes of physician notes, therefore very objective. Unclear whether outcome assessors know exposure status, however this is unlikely to impact the outcome classification	**Moderate** Justification: No statistical plan was given, however all results that were mentioned in method were given	**Serious**

For peer review only

					the outcome was found.			
Evans-Hoeker et al (2018)	**Moderate** Justification: All three confounding domains were adjusted for	**Moderate** Justification: The medication exposure was assessed throughout a previous RCT, with regulators of the trial ensuring accurate medication exposure classification	**Serious** Justification: It is unlikely the start of intervention and follow-up were at the same time point, additionally no analysis was utilised to account for the differing effect of intervention at differing points during pregnancy	**Low** Study wasn't undergone in an experimental context	**Moderate** Justification: 100% of individuals were included from the trial into the study. Less than 10% of PHQ scores were missing, therefore missing covariate data, no evidence that this missing data was not driven by the true value of the outcome.	**Low** Justification: The outcome was assessed throughout the RCT through testing and so a very thorough and reliable method. Unlikely that outcome assessors knowing the exposure would influence their classification of it	**Moderate** Justification: No statistical analysis plan was provided, however, all analysis that was stated in methods was completed and results given	**Serious**
Richardson et al (2019)	**Moderate** Justification: All three confounding domains had a factor that had been adjusted for including presence of maternal depression, maternal age and alcohol	**Moderate** Justification: Exposure classification measured prospectively and by a healthcare professional over the phone. Classification of intervention status would not be influenced by the outcome	**Moderate** Justification: The start of intervention and follow-up were likely not at the same time point as individual rung up not necessarily at start of exposure. However, analysis used was time dependent cox models therefore the differing effect of intervention across different time periods was accounted for.	**Low** Study wasn't undergone in an experimental context	**Low** Justification: They don't have complete data as missing data was reported in only the confounding variables, however the incomplete data for these covariates is unlikely to have been influenced by the true value of the outcome because this was determined through clinician	**Low** Justification: The outcome is a very objective measure. The outcome was measured through clinician self-report, and although exposure would have been known to them, this is unlikely to have influenced the outcome reporting.	**Moderate** Justification: Although there was no statistical analysis plan there were no missing parts to the analysis.	**Moderate**

For peer review only

					rather than self report.			
Wu et al (2019)	**Serious** Justification: Only individual characteristics and lifestyle exposure domains had a factor adjusted for in them - however indication for treatment was not adjusted for at all - therefore deemed serious as not all confounding domains satisfied.	**Serious** Justification: The exposure was classified at the end of first trimester interview - so a time point after the start of follow up. Additionally in a lot of the cases the exposure was recorded after the SA had already happened. Potentially differential misclassification between exposure and outcome individuals	**Moderate** Start of intervention and follow-up did not coincide, however a cox proportional hazard survival model was utilised to counteract the differing effects that exposure will have at different time points during pregnancy	**Low** Study wasn't undergone in an experimental context	**Serious** Justification: A flow diagram and tables with the details of missing data was illustrated. Individuals were excluded if they had missing data from the interview, this results in missing outcome data that could have been a result of the true value of the outcome.	**Low** Justification: The outcome was measured through a questionnaire, this was then confirmed through medical records. Although outcome assessors could have been aware of the exposure status it is unlikely assessment of outcome would have been influenced by this.	**Moderate** Justification: No statistical analysis plan was given	**Serious**
Bahat et al (2020)	**Serious** Justification: Unclear what they adjusted for in this analysis and was not stated, however an adjusted estimate was given so some level of confounding was accounted for	**No information**	**Moderate** Justification: It was unclear whether the start of intervention and follow-up were at the same point, however, the analysis was restricted to first trimester where there is thought to be similar effects of Ads across first trimester so accounting for this	**Low** Study wasn't undergone in an experimental context	**No information**	**No information**	**Serious** Justification: No statistical analysis plan was given, additionally not all of the analysis that said was going to be conducted was released as results	**Serious**

For peer review only

Kolding et al (2021)	**Critical** No confounding adjustment done	**Critical**	**Critical**	**Critical**	**Critical**	**Critical**	**Critical**	**Critical**
Ankarfeldt et al (2021)	**Moderate** Justification: All three confounding domains of indication for treatment, lifestyle exposures and individual characteristics had one factor adjusted for	**Moderate** Justification: Exposure was classified through prescription databases therefore likely to be reliable, however may not reflect true ingested drug usage.	**Moderate** Justification: Start of intervention and follow-up were likely not at same time point. However, a cox proportional hazard survival model was utilised to counteract the differing effects that exposure will have at different time points during pregnancy	**Low** Justification: Study wasn't undergone in an experimental context	**Low:** Justification: There was a small number of missing values for two covariates, however as this was not influenced by the value of the outcome and the missing values were imputed it poses minimal risk of bias.	**Low** Justification: The outcome was measured through patient registers, therefore very objective and reliable.	**Moderate** Justification: No statistical analysis plan was given but all of the analysis planned and stated in methods was conducted and given.	**Moderate**

For peer review only

S6 Table. Confounding adjustments completed

Study	Confounding domain												
	Indication for treatment		Individual characteristics								Lifestyle exposures		
	Presence of depression	Severity of depression	History of SA	Maternal age	Gestational age	Polypharmacy	SEP	Ethnicity	BMI	Comorbidities	Smoking	Alcohol	Recreational drugs
Chambers et al (1996)													
Kulin et al (1998)													
Einarson et al (2001)													
Einarson et al (2003)													
Sivojelezova et al (2005)													
Chun-Fai-Chan et al (2005)													
Djulus et al (2006)													
Einarson et al (2009)													
Nakhai-Pour et al (2010)													
Paulus et al (2010)													
Chan et al (2011)													
Einarson et al (2011)													
Ban et al (2012)													
Klieger-Grossman et al (2012)													
Kjaesgaard et al (2013)													
Andersen et al (2014)													
Abadie et al (2015)													
Johansen et al (2015)													
Tw Winkel et al (2016)													
Almeida et al (2016)													
Evans-Hoeker et al (2018)													
Richardson et al (2019)													
Wu et al (2019)													

For peer review only

**Bahat et al (2020)**													
**Kolding et al (2021)**													
**Ankarfeldt et al (2021)**													

Green highlighted box indicating factor adjusted for and blank box indicating factor was not adjusted for

For peer review only

MOOSE (Meta-analyses Of Observational Studies in Epidemiology) Checklist

A reporting checklist for Authors, Editors, and Reviewers of Meta-analyses of Observational Studies. You must report the page number in your manuscript where you consider each of the items listed in this checklist. If you have not included this information, either revise your manuscript accordingly before submitting or note N/A.

Reporting Criteria	Reported (Yes/No)	Reported on Page No.
**Reporting of Background**		
Problem definition	Yes	pg4
Hypothesis statement	Yes	pg5
Description of Study Outcome(s)	Yes	pg6
Type of exposure or intervention used	Yes	pg6
Type of study design used	Yes	pg6
Study population	Yes	pg6
**Reporting of Search Strategy**		
Qualifications of searchers (eg, librarians and investigators)	Yes	pg 6 &7
Search strategy, including time period included in the synthesis and keywords	Yes	pg 6
Effort to include all available studies, including contact with authors	Yes	pg 6
Databases and registries searched	Yes	appendix Table s1
Search software used, name and version, including special features used (eg, explosion)	Yes	appendix Table s1
Use of hand searching (eg, reference lists of obtained articles)	Yes	NA
List of citations located and those excluded, including justification	Yes	Table 1 & Appendix Table S2
Method for addressing articles published in languages other than English	Yes	pg 6
Method of handling abstracts and unpublished studies	Yes	pg 6
Description of any contact with authors	Yes	pg 6
**Reporting of Methods**		
Description of relevance or appropriateness of studies assembled for assessing the hypothesis to be tested	Yes	pg 7 & 8
Rationale for the selection and coding of data (eg, sound clinical principles or convenience)	Yes	pg 7 & 8
Documentation of how data were classified and coded (eg, multiple raters, blinding, and interrater reliability)	Yes	pg 7 & 8
Assessment of confounding (eg, comparability of cases and controls in studies where appropriate	Yes	pg 7, 12, 13 & 1[illegible]

For peer review only

Reporting Criteria	Reported (Yes/No)	Reported on Page No.
Assessment of study quality, including blinding of quality assessors; stratification or regression on possible predictors of study results	Yes	pg 7
Assessment of heterogeneity	Yes	pg 8
Description of statistical methods (eg, complete description of fixed or random effects models, justification of whether the chosen models account for predictors of study results, dose-response models, or cumulative meta-analysis) in sufficient detail to be replicated	Yes	pg 8
Provision of appropriate tables and graphics	Yes	Fig S1, S2, S3
**Reporting of Results**		
Table giving descriptive information for each study included	Yes	Table 1
Results of sensitivity testing (eg, subgroup analysis)	Yes	appendix Table s3
Indication of statistical uncertainty of findings	Yes	appendix Table s3
**Reporting of Discussion**		
Quantitative assessment of bias (eg, publication bias)	Yes	Appendix Figure S1
Justification for exclusion (eg, exclusion of non–English-language citations)	Yes	Appendix table s2
Assessment of quality of included studies	Yes	appendix s4
**Reporting of Conclusions**		
Consideration of alternative explanations for observed results	Yes	pg 11 & 12
Generalization of the conclusions (ie, appropriate for the data presented and within the domain of the literature review)	Yes	pg 11 & 12
Guidelines for future research	Yes	pg 15
Disclosure of funding source	Yes	pg 16

Once you have completed this checklist, please save a copy and upload it as part of your submission. DO NOT include this checklist as part of the main manuscript document. It must be uploaded as a separate file.

For peer review only

The association between antidepressant use during pregnancy and miscarriage: A systematic review and meta-analysis

Journal:	*BMJ Open*
Manuscript ID	bmjopen-2023-074600.R1
Article Type:	Original research
Date Submitted by the Author:	20-Sep-2023
Complete List of Authors:	Smith, Sophie; University of Bristol Medical School,<br>Martin, Flo; University of Bristol Medical School<br>Rai, Dheeraj; University of Bristol, Centre for Academic Mental Health, School of Social & Community Medicine<br>Forbes, Harriet; University of Bristol,
**Primary Subject Heading**:	Mental health
Secondary Subject Heading:	Epidemiology, Mental health, Obstetrics and gynaecology
Keywords:	Depression & mood disorders < PSYCHIATRY, EPIDEMIOLOGY, Maternal medicine < OBSTETRICS

I, the Submitting Author has the right to grant and does grant on behalf of all authors of the Work (as defined in the below author licence), an exclusive licence and/or a non-exclusive licence for contributions from authors who are: i) UK Crown employees; ii) where BMJ has agreed a CC-BY licence shall apply, and/or iii) in accordance with the terms applicable for US Federal Government officers or employees acting as part of their official duties; on a worldwide, perpetual, irrevocable, royalty-free basis to BMJ Publishing Group Ltd ("BMJ") its licensees and where the relevant Journal is co-owned by BMJ to the co-owners of the Journal, to publish the Work in this journal and any other BMJ products and to exploit all rights, as set out in our licence.

The Submitting Author accepts and understands that any supply made under these terms is made by BMJ to the Submitting Author unless you are acting as an employee on behalf of your employer or a postgraduate student of an affiliated institution which is paying any applicable article publishing charge ("APC") for Open Access articles. Where the Submitting Author wishes to make the Work available on an Open Access basis (and intends to pay the relevant APC), the terms of reuse of such Open Access shall be governed by a Creative Commons licence – details of these licences and which Creative Commons licence will apply to this Work are set out in our licence referred to above.

Other than as permitted in any relevant BMJ Author's Self Archiving Policies, I confirm this Work has not been accepted for publication elsewhere, is not being considered for publication elsewhere and does not duplicate material already published. I confirm all authors consent to publication of this Work and authorise the granting of this licence.

For peer review only

The association between antidepressant use during pregnancy and miscarriage: A systematic review and meta-analysis

Centre for Academic Mental Health, University of Bristol, Bristol (Sophie Smith MSc)
Centre for Academic Mental Health, University of Bristol, Bristol (Florence Martin MSc)
Centre for Academic Mental Health, University of Bristol, Bristol (Dr Dheeraj Rai MRCPsychPHD)
Centre for Academic Mental Health, University of Bristol, Bristol (Dr Harriet Forbes PhD)

Correspondence to Sophie Smith, Centre for Academic Mental Health, University of Bristol, Bristol, BS8 2BN
Sophie.smith@bristol.ac.uk

For peer review only

Word count: 3719

Abstract

Background

Literature surrounding the association between antidepressant use during pregnancy and miscarriage is conflicting. We aimed to conduct a systematic review and meta-analysis of studies among pregnant women regarding the association between exposure to antidepressants during pregnancy and the risk of miscarriage, compared to pregnant women not exposed to antidepressants.

Design

We conducted a systematic review and meta-analysis of non-randomised studies.

Data sources

We searched MEDLINE, EMBASE and PsychINFO up to 6th August 2023.

Eligibility criteria and outcomes

Case-control, cohort and cross-sectional study designs were selected if they compared individuals exposed to any antidepressant class during pregnancy to comparator groups of either no antidepressant use or an alternate antidepressant.

Data extraction and synthesis

Effect estimates were extracted from selected studies and pooled using a random-effects meta-analysis. Risk of bias (RoB) was assessed using the ROBINS-I tool, and heterogeneity assessed using the I^2 statistic. Subgroup analyses were utilised to explore antidepressant classes and the impact of confounding by indication.

Results

1800 records were identified from the search, of which 29 were included in the systematic review and meta-analysis. The total sample included 5,671,135 individuals. Antidepressant users initially appeared to have a higher risk of miscarriage compared to unexposed individuals from the general population (summary effect estimate: 1.24, 95% CI: 1.18-1.31,

For peer review only

I^2 = 69.2%; number of studies (n)=29). However, the summary estimate decreased when comparing against unexposed individuals with maternal depression (1.16, 1.04-1.31; I^2 = 58.6%; n=6), suggesting confounding by indication may be driving the association. 22 studies suffered from serious RoB, and only two of the 29 studies were deemed at moderate RoB.

Conclusions

After accounting for maternal depression there is little evidence of any association between antidepressant use during pregnancy and miscarriage. Instead, the results indicate the biasing impact of confounding by indication.

Key words: Depression; antidepressant use; miscarriage; spontaneous abortion; confounding by indication

Strengths and limitations of the study:

- A large amount of studies were identified through an extremely thorough search strategy, thus enhancing statistical power and insight over previous reviews.
- Confounding by indication and depression severity was considered in the analysis and results, elucidating the true independent effect of antidepressant use during pregnancy.
- The ROBINS-I tool was used to evaluate the strength and quality of evidence.
- The studies included could suffer from publication bias, and small studies producing inverse relationships are absent from the field.
- High heterogeneity existed between papers, likely due to the included studies investigating different research questions, implementing different methodologies and populations, timing of exposure and confounding adjustment, thereby reducing the quality of the meta-analysis.

Introduction

Depression affects around 264 million people worldwide and is one of the top three leading causes of years lived with disability globally.(1) This high prevalence is also seen among pregnant women, with 15-23% suffering from a depressive disorder.(2) Around 3-8% of

For peer review only

women are prescribed antidepressants during their pregnancy in Europe.(3) This figure appears to be on an upwards trajectory since the 1990s,(4) potentially driven by both increasing prenatal depression rates,(5) and a shortage of non-pharmacological treatment options.(6)

Miscarriage, also referred to as spontaneous abortion, is defined as pregnancy loss before viability, however, the gestational threshold of viability varies between countries, from 20-28 weeks.(7) In the UK, viability is determined as up to 24 weeks.(7) Depending on the definition, miscarriage risk varies; on average it occurs in approximately 15% of pregnancies.(7) Several studies and reviews found an increased miscarriage risk in women using antidepressants during pregnancy, compared to unexposed individuals.(8-10) Antidepressants can cross the placental barrier through passive diffusion,(11) highlighting biological plausibility. Selective serotonin reuptake inhibitors (SSRIs) and serotonin-norepinephrine reuptake inhibitors (SNRIs) are the most frequently used pharmacotherapy option for depression,(12) both of which increase serotonin levels in the synaptic cleft.(3, 13) Serotonergic systems are involved in embryonic developmental processes including neural crest cell differentiation,(13) a process producing cells and tissue types.(14) This highlights the importance of serotonin in embryogenesis thus its potential influence on miscarriage. Alternatively, untreated maternal depression has been shown to be associated with miscarriage(15). This is an issue in observational studies known as confounding by indication, whereby maternal depression can independently affect the outcome of miscarriage. This could be through a number of mechanisms and mediators including lifestyle factors,(16) or stress and inflammation.(15) Including pregnant women in randomised controlled trials (RCTs) is unethical, thus all evidence is observational and therefore susceptible to bias, making causal inference challenging. This is highlighted by studies whose observed associations between antidepressant use during pregnancy and miscarriage attenuated after stratifying for maternal depression.(17, 18)

The methodological limitations that exist in previous reviews means it remains unclear whether a causal relationship exists. Therefore, clear evidence and guidance around the association between antidepressant use during pregnancy and miscarriage is important as it will allow depressed pregnant women to make well-informed decisions and reduce their

For peer review only

decisional conflict.(19) Poor perinatal mental health is becoming an ever-growing issue, up-to-date studies are needed now which investigate child outcomes in depressed women to help drive screening, intervention and prevention strategies.

Existing reviews examining this relationship investigated several perinatal outcomes and produced differing conclusions.(20-22) These reviews have some methodological limitations including incomplete search strategies,(20) do not include more recent studies from the last 10 years,(21, 22) and did not fully address confounding by indication,(20-22) a crucial element in isolating the true effect of antidepressants. This issue was addressed in the current review through utilising a depressed comparator, to allow for increased comparability between exposed and unexposed individuals.

The main objective of this study is to conduct a systematic review and meta-analysis of studies among pregnant women regarding the association between exposure to antidepressants during pregnancy and the risk of miscarriage, compared to pregnant women not exposed to antidepressants.

Methods

Protocol registration

This study is conducted in accordance with the Preferred Reporting Items for Systematic Review and Meta Analysis (PRISMA) 2020, as well as the Cochrane handbook(23). The protocol for this meta-analysis was registered in PROSPERO CRD42021262839, available from https://www.crd.york.ac.uk/prospero/display_record.php?ID=CRD42021262839

Search strategy and information sources

We searched the databases, EMBASE (Ovid, 1980 to 2023), MEDLINE (Ovid, 1946 to 2023) and PsycInfo (Ovid, 1806 to 2023) from database inception to 8th March 2021 using a comprehensive search strategy (Supplementary Table S1). In brief the following search terms were used: (antidepress* OR [generic/branded antidepressant name) AND (spontaneous abortion OR miscarriage). The search was updated on 4th August 2021 prior

For peer review only

to commencement of data analysis, a final search before finishing the review was conducted on 21st January 2022, as well as a search to update findings before publication on 6th August 2023. Backward citation searching was conducted from papers and existing reviews, additionally, conference abstracts found in the searches were included in the review. The outcome of stillbirth was also included in our search strategy as these two outcomes are frequently reported together. However, stillbirth was not included in this review due to a higher number of relevant studies found than expected, ensuring literature was investigated at a thorough level. We placed no restrictions on language or publication status. Abstracts in a language other than English were translated using a translation software, and full texts were assessed using a translator.

Eligibility criteria

Studies were included if the study population included pregnant women, the exposure was antidepressants, and the outcomes included miscarriage. Any antidepressant class was eligible, and no restriction was placed on timing, dose, or duration of use. The comparator groups included pregnant women prescribed a different antidepressant to the primary specified type, or women with no antidepressant exposure. Studies investigating polypharmacy involving medication other than antidepressants were excluded, however, studies investigating more than one antidepressant class were included. Observational studies included utilised a cohort, case-control, or cross-sectional design. RCTs were included if they fit the eligibility criteria. Reviews, meta-analyses, ecological studies, case reports and case series were excluded.

Study selection

The papers identified from the search strategy had their title and abstract screened independently by two reviewers in parallel (FM and HF). We used Endnote to de-duplicate references. Outputs of the review were then cross-checked, and discrepancies discussed with a third reviewer (SS). The full text of papers that passed title and abstract screening were obtained through Endnote, and three researchers completed a full-text review of a proportion of papers (SS, FM and HF). Final decisions and discrepancies were discussed within the whole team, including a consultant psychiatrist (DR).

For peer review only

Data extraction

A standardised template was used for data extraction. A PECOS framework was utilised and information on five domains of population, exposure, comparator, outcomes, and study characteristics was obtained. Variables of study design, country of study, study period, data sampling methods, type and timing of antidepressant exposure, definition of exposure and comparator groups, identification of exposure and outcome, sample size, confounding adjustment, raw data and adjusted estimates (odds ratio (OR), risk ratio (RR), hazard ratio (HR)) including confidence intervals (CIs) were extracted. If more than one comparator group was utilised, the raw data and adjusted estimates for each comparison were extracted. Data on any secondary outcomes were not extracted. Data extraction was conducted independently by two reviewers for all papers, outputs were cross-checked (SS and FM), and discrepancies were discussed with a third reviewer (HF).

Risk of bias assessment

Studies were assessed using the ROBINS-I tool.(24) This tool evaluates non-randomised studies estimating intervention effects. Seven domains were assessed, including issues arising prior to and after intervention. The ROBINS-I tool is designed for cohort studies, therefore a template alteration specific to case-control studies was sought from the author of the ROBINS-I tool to allow for continuity in risk of bias (RoB) assessment across all study designs. RoB was independently assessed by two reviewers in parallel for all studies (SS and FM). The scoring and justification were compared, with any discrepancies discussed with a third reviewer (HF).

Statistical analysis

Meta-analyses were performed using STATA (version 16.0). Results were presented by comparator group used, specifically general population unexposed, depressed unexposed and depressed alternative antidepressant exposure. Additionally, subgroup analysis on SSRIs and SNRIs were conducted. Adjusted effect estimates were the preferential estimate when pooling results. HR, RR and OR were treated as equivalent and pooled together, a sensitivity analysis on this assumption was conducted (Supplementary Figure S1). If studies gave estimates for multiple exposure groups including differing trimester estimates, these were pooled into one result using a meta-analysis. When adjusted estimates were not provided,

For peer review only

ORs and standard errors were produced from raw data. Analyses were conducted using a random-effects model, due to the high likelihood of heterogeneity expected. Heterogeneity between studies was assessed using the I^2 statistic.(25) Additionally, a funnel plot and Egger test were conducted to assess evidence for publication bias.(26) A sensitivity analysis was conducted whereby papers with an overall critical RoB, or no information as identified through ROBINS-I tool were excluded.

Patient and Public Involvement

No patient or public involvement.

Results

Search results

The initial and final search retrieved 2010 citations. After duplicates were removed, 1800 records had title and abstract screening, resulting in a further 1751 records being excluded. The full text of 49 articles were assessed for eligibility. Articles were excluded due to reasons including incorrect study design, insufficient data or if only stillbirth was investigated (Supplementary Table S2). This resulted in 25 studies being excluded, and 24 included, with the final searches adding a further five papers to the SR and meta-analysis;(9, 10, 17, 18, 27-51) highlighted in the PRISMA flow diagram (Figure 1).(52)

Study characteristics

Supplementary Table S3 details characteristics of the included studies. Only observational studies were found; 25 cohort, and four case-control studies, (10, 40, 50, 51) and all studies were published between 1996 and 2022. The total number of subjects investigated across case-control was 314,682, compared to 5,356,453 across cohort studies. The study size varied from 247 to 1,281,418. The miscarriage definition was rarely stated, however, ranged from loss of fetus at either 20 (n=3),(10, 42, 45) 22 (n=2)(17, 18) or 24 weeks' (n=1)(44) gestation. A mixture of comparator groups were utilised across studies. All studies had a comparator group of unexposed individuals without depression; some papers used additional comparator groups including depressed pregnant women exposed to an alternative antidepressant to the primary specified type, unexposed depressed pregnant

women,(17, 18, 37, 42, 43, 50) and pregnant individuals using antidepressants whereby the indication for treatment wasn't depression.(17, 32, 43) Exposure status was predominantly identified through two methods. Firstly, 16 studies utilised self-report to teratogen information services through either the individual or their physician.(9, 27-34, 36, 38, 40, 41, 43-45) Secondly, fourteen studies used prescription databases.(10, 17, 18, 37, 39, 42, 47, 48) Individual antidepressant types were investigated, including 12 studies examining SSRIs(18, 27-29, 31, 34, 37-39, 43, 45, 50) and five SNRIs.(29, 41, 44, 46, 48)

Risk of bias assessment

The ROBINS-I tool found much of the literature to be at serious RoB. Supplementary Table S4 highlights that only two out of 29 papers had an overall moderate RoB,(44, 48) compared to 22 deemed serious, and the remaining deemed critical. This tool highlighted a key area of bias being confounding. The three crucial confounding domains identified for this field were indication for treatment, individual characteristics, and lifestyle exposures. The confounding variables believed to be most important included presence and severity of depression, as well as gestational age. Although a large proportion of studies stratified by depression (62%), only 14% and 31% of studies accounted for depression severity and gestational age, respectively (Supplementary Table S5). Confounding adjustments varied dramatically between studies suggesting a potential heterogeneity source. As very few confounding variables were included in these studies, it limits the reliability of the results of this systematic review.

Publication bias

Publication bias was assessed through visual inspection of the funnel plot (Supplementary Figure S2) and the Egger test. Asymmetry of the funnel plot indicated potential for publication bias, and the Egger test produced a p-value of 0.001.

Sensitivity analysis

Papers with an overall critical RoB or inadequate information to determine their bias category were removed (n=5). This sensitivity analysis evidenced that these papers weren't influencing the overall effect estimate for the general population analyses (Supplementary Table S6).

For peer review only

Meta-analysis results

Across all 29 studies using an unexposed general population comparator, antidepressant use was associated with miscarriage. Figure 2 illustrates the summary effect estimate (pooled odds ratio = 1.24 95%CI: 1.18-1.31, I^2=69.2%, p<0.001), which included 18 adjusted estimates. However, when stratifying for maternal depression and utilising a comparator of unexposed depressed pregnant women, the summary estimate decreased (pooled odds ratio= 1.16 95% CI: 1.04-1.31, I^2=58.6%, p=0.034) (Figure 3).

Additionally, studies were separated which investigated SSRIs or SNRIs and their possible varying risks. When comparing SSRI use and no antidepressant use, the pooled summary estimate for the outcome of miscarriage was 1.29 (95% CI: 1.15-1.44, I^2=81.3%, p<0.001). Alternatively, when comparing SNRI use with no antidepressant use, a higher summary estimate was observed (pooled odds ratio = 1.42 95% CI: 1.11-1.81, I^2= 49.6%, p=0.11) (Figure 2).

Subgroup analyses investigating SSRIs and SNRIs separately were performed again after restricting to a depressed cohort. When comparing SSRI use and individuals using other antidepressant types, it produced an attenuated summary estimate of 1.03 (95% CI: 0.93-1.15, I^2=0%, p=0.972). Whereas the summary estimate comparing SNRI against other antidepressant use on the outcome of miscarriage was slightly higher (pooled summary estimate = 1.19 95%CI: 1.02-1.39, I^2=0%, p=0.665) (Figure 3).

Discussion

Summary of evidence

This is the largest and most up-to-date review investigating the association between antidepressant use during pregnancy and miscarriage, with around twice as many studies included than previous reviews.(20-22) After pooling best estimates from 29 studies, we found little evidence to suggest an increased risk of miscarriage among antidepressant users after stratifying by maternal depression, with CIs almost spanning the null. The increased

For peer review only

risk of miscarriage among pregnant women taking antidepressants compared to unexposed pregnant women in the general population is likely driven by confounding by indication.

Interpretation of findings

The general population analysis result was comparable to other smaller meta-analyses, which found effect estimates of 1.45, 1.49 and 1.47.(8, 20, 22) The increased estimates found by these papers are likely driven by fewer included studies, with 11, 14, and six respectively, compared to 29 in our study. The elevated effect estimates when utilising a general population control could likely be driven by confounding by indication, a bias which is lessened when comparing with depressed controls. To the best of our knowledge, meta-analyses in the field investigating miscarriage have not yet utilised an unexposed depressed comparator; thus, no comparable results exist. As only six studies were used in this subgroup meta-analysis, the summary estimate lacks power and robustness, inducing potential for chance findings, highlighted by large standard errors. Additionally, this weak miscarriage risk could be reflecting depression severity, whereby unexposed depressed individuals have a lower severity of depression than exposed individuals. Studies have indicated miscarriage risk between individuals continuing and discontinuing antidepressant use upon start of pregnancy is similar.(18, 48) Therefore, future studies should incorporate the comparator of discontinued antidepressant use to allow for more comparable depression severity. Furthermore, other confounding factors that weren't adequately controlled for could be biasing this estimate, thus, adjusting for all key confounders is essential in future studies to minimise residual confounding.

Our study found that SNRIs carry a greater risk of miscarriage than SSRIs when compared to an unexposed general population comparator group. No meta-analyses have been conducted on SNRIs to our knowledge, likely due to the higher prevalence of other antidepressant use during pregnancy, for example SSRIs.(12) Previous reviews utilising a fixed-effects method found odds ratios of 1.70 and 1.87 when comparing SSRIs to unexposed general population comparators.(53, 54) The lower effect estimate of 1.30 observed in our study may be partially driven by the use of a random-effects model to account for high heterogeneity, as well as the inclusion of an additional eight studies. The elevated point estimate for SNRIs compared with SSRIs may suggest that miscarriage risk

For peer review only

differs between antidepressant classes. Alternatively, these estimates could be highlighting confounding by indication, which was further explored in a set of subgroup analyses that stratified by maternal depression. The summary effect estimates between both SSRIs and SNRIs and other antidepressant classes attenuated towards the null, thus, emphasising the potential impact of confounding by indication. The SSRI analyses had consistently lower estimates, including against other antidepressant classes, when comparing pooled estimates with those from the SNRI analyses. The difference in point estimates within the subgroup analysis could be reflecting the differing population types, as SSRIs are used as first line treatment for depression,(55) whereas SNRIs are often utilised in individuals with more severe depression or when first line options may have not worked.(56) Thus, these results provide evidence for the potential impact of confounding by depression severity, whereby the elevated pooled estimate from the SNRI analysis could be driven by non-exchangeability between individuals who use different classes of antidepressants. Confounding by disease severity was highlighted in the largest included study in the SNRI analysis which discussed that the risk difference observed between duloxetine (SNRI) and SSRI users could be depression severity and residual confounding.(48) However, given the confidence intervals overlap for the subgroup analyses of SSRIs and SNRIs, it is plausible that the true pooled estimates for each analysis are the same. Future network meta-analyses may help elucidate the intricacies of the relationship between individual antidepressant medications or classes and miscarriage.

Limitations of included studies

The included studies had several limitations. Firstly, only 7% of studies had a moderate overall RoB, due to the limited level of confounding adjustment in papers within the field. Only nine papers adjusted for any variable within all three key confounding domains (Supplementary Table S5). Therefore, the included studies may suffer from residual confounding, as strong risk factors for miscarriage, including cotreatments and comorbidities are likely imbalanced between exposure groups,(44) and any effect estimates may be biased away from the null. Additionally, we couldn't assess by length of depressive disorder as this was rarely stated in the studies.

For peer review only

A second limitation of the included studies is information bias due to exposure misclassification. Exposure was assessed mainly via self-report from physician or individual, or prescription databases. However, only 60% agreement has been found between prescription and self-report data,(18) with conflicting evidence as to the most accurate method to assess exposure to medication.(57, 58) Teratogen surveillance data was used in several included studies, however, it poses difficulty as women enquiring about antidepressant exposure are automatically classified as exposed, when they may discontinue use after enquiry. Prescription database error could be less impactful, as it likely suffers from non-differential misclassification and biases the estimate towards the null.(18) Although, self-report data could be more accurate in terms of ingested drug usage. Nevertheless, sensitivity analyses comparing self-report and prescription database inaccuracies indicate exposure misclassification does not impact effect estimates significantly, and only reduces precision.(18) Future studies should also look to report number of antidepressant prescriptions as this was rarely collected in previous studies, and is a crucial factor to be investigated.

Thirdly, selection bias is another common methodological limitation in this field. A significant issue observed is that the start of intervention and follow-up do not coincide. This results in conditioning on women who haven't experienced a miscarriage between the start of intervention and follow-up but excluding eligible individuals who have. Additionally, exposed individuals are more likely to be in consultation with their healthcare professional when planning a pregnancy to discuss potential risks of antidepressants, compared to unexposed individuals. This results in exposed individuals having a longer observed risk period and as such, more chance of detecting and recording miscarriages, therefore biasing results away from the null. (59) These methodological errors contributing to a bias which occurs when individuals who have already experienced the outcome of interest at the point of study commencement not being included,(60) referred to as left truncation.(61)

Strengths and limitations of review

Our review has important strengths not addressed in any previous review investigating miscarriage. Confounding by indication and depression severity was a key part of the analysis and results, elucidating the true independent effect of antidepressant use during

pregnancy. As this important bias has not previously been addressed in any other review in the field, it emphasises the requirement for accounting for psychiatric illness in future reviews and studies to avoid biased estimates. To the best of our knowledge, our study was also the first review in this field to assess RoB using the ROBINS-I tool. This tool gave a significant advantage over the commonly used Newcastle-Ottawa scale and highlighted the need for more thorough confounding adjustment and reporting of missing data in future studies. Additionally, the issues surrounding exposure recall bias were minimised using mainly cohort studies, and the included case-control studies having prospective exposure measurement. Finally, 29 studies published up to 2022 were identified and used in the review through an extremely thorough search strategy, thus enhancing statistical power and insight over previous reviews.

The review also had limitations. The studies included could suffer from publication bias as few studies were distributed around the null line and most found associations that transcended the null (Supplementary Figure S2). Additionally, small studies producing inverse relationships are absent from the field. This conclusion was supported by the small p-value produced in the Egger test. If only positive results are being published this could result in exaggerated review results. The authors didn't include grey literature due to limited resource which could have also contributed to this finding. Alternatively, these conclusions may not be driven by publication bias, but instead either real causal associations or lack of methodological rigour throughout the studies.

High heterogeneity also existed between papers, likely due to the included studies investigating different research questions, implementing different methodologies and populations, timing of exposure and confounding adjustment, thereby reducing the quality of the meta-analysis. Additionally, the lack of available data prevented a trimester specific subgroup analysis. Previous studies have identified first trimester miscarriage risk with antidepressant use to be higher compared to second trimester,(18) indicating antidepressant exposure could be time sensitive. However, this could reflect the time window for highest risk of miscarriage being between week 7 and 12, during first trimester.(62) Future reviews should aim to incorporate this subgroup analysis to investigate whether it alters conclusions made.

For peer review only

Conclusion

In conclusion, we did not find any clear evidence to suggest antidepressants increase the risk of miscarriage, though the results should be treated with caution due to the large heterogeneity present. This report highlights the effect of confounding by indication on previous literature. As RCTs cannot be utilised, future observational studies with larger sample sizes and more robust confounding adjustment, especially in relation to indication for treatment are needed. Triangulating results with differing methodologies should help strengthen causal inference. Additionally, network meta-analyses are needed to fully investigate the differing risk between antidepressant classes. Overall, the present study provides a summary of the most up-to-date findings of miscarriage risk following antidepressant use during pregnancy, to better inform clinicians and women when weighing up treatment for depression during pregnancy.

PROSPERO 2021 CRD42021262839 Available from: https://www.crd.york.ac.uk/prospero/display_record.php?ID=CRD42021262839

For peer review only

Declaration of interests
All authors declare no competing interests.

Funding
SS is supported by an NIHR predoctoral fellowship (NIHR301109). FM is supported by the Wellcome Trust (WT 218495/Z/19/Z). HF and DR acknowledge support from the NIH (1R01NS107607). This research was also supported by the NIHR Biomedical Research Centre at the University of Bristol and the University Hospitals Bristol and Weston NHS Foundation Trust.

Acknowledgements
The views in this research are of the authors and not of the funders. The authors thank the corresponding authors and coauthors of the studies included in this review. We thank Professor Julian Higgins who was extremely helpful with queries involving the ROBINS-I tool.

Author Contributions
All authors helped with interpretation of results. SS did the full-text screening, data extraction, risk of bias assessment, statistical analysis and wrote the report. FM did the title and abstract screening, full text screening, the second review of data extraction and risk of bias assessment, statistical analysis and had input on the report writing. HF did the title and abstract screening, full text screening, and had input on all other elements of the paper. DR suggested the research question, provided supervision and comment on the manuscript.

Data sharing statement
Please contact the first author (SS) if you would like to see any data not included in the article or supplementary material.

For peer review only

Figure captions

Figure 1. PRISMA flow diagram
Flow diagram illustrating the number of included and excluded studies at each stage of screening, and reasons for exclusion. This version has been adapted to include the element of additional papers found in following searches.

Figure 2. Subgroup meta-analyses utilising general population comparator

Three subgroup analyses conduced. First subgroup assessing any antidepressant use compared to unexposed individuals in general population. Second subgroup comparing SSRI use and unexposed general population individuals. Third subgroup comparing between SNRI use and unexposed general population individuals.

Figure 3. Three random-effects meta-analysis utilising depressed cohort comparator

First subgroup assessing any antidepressant use against unmediated depressed individuals. Second subgroup comparing SSRI use and different types of antidepressants (this includes data from papers which compared SSRIs and SNRIs, SSRIs and TCAs, SSRIs and any other antidepressants). Third subgroup analysis compared SNRI use and different types of antidepressants (this includes data from papers comparing SNRI and SSRI).

For peer review only

References

1. Disease GBD, Injury I, Prevalence C. Global, regional, and national incidence, prevalence, and years lived with disability for 354 diseases and injuries for 195 countries and territories, 1990-2017: a systematic analysis for the Global Burden of Disease Study 2017. Lancet. 2018;392(10159):1789-858.

2. Tak CR, Job KM, Schoen-Gentry K, Campbell SC, Carroll P, Costantine M, et al. The impact of exposure to antidepressant medications during pregnancy on neonatal outcomes: a review of retrospective database cohort studies. Eur J Clin Pharmacol. 2017;73(9):1055-69.

3. Rai D, Lee BK, Dalman C, Newschaffer C, Lewis G, Magnusson C. Antidepressants during pregnancy and autism in offspring: population based cohort study. Bmj. 2017;358:j2811.

4. Cooper WO, Willy ME, Pont SJ, Ray WA. Increasing use of antidepressants in pregnancy. Am J Obstet Gynecol. 2007;196(6):544 e1-5.

5. Pearson RM, Carnegie RE, Cree C, Rollings C, Rena-Jones L, Evans J, et al. Prevalence of Prenatal Depression Symptoms Among 2 Generations of Pregnant Mothers: The Avon Longitudinal Study of Parents and Children. JAMA Netw Open. 2018;1(3):e180725.

6. Ford E, Lee S, Shakespeare J, Ayers S. Diagnosis and management of perinatal depression and anxiety in general practice: a meta-synthesis of qualitative studies. Br J Gen Pract. 2017;67(661):e538-e46.

7. Quenby S, Gallos ID, Dhillon-Smith RK, Podesek M, Stephenson MD, Fisher J, et al. Miscarriage matters: the epidemiological, physical, psychological, and economic costs of early pregnancy loss. Lancet. 2021;397(10285):1658-67.

8. Hemels ME, Einarson A, Koren G, Lanctot KL, Einarson TR. Antidepressant use during pregnancy and the rates of spontaneous abortions: a meta-analysis. Ann Pharmacother. 2005;39(5):803-9.

9. Einarson A, Choi J, Einarson TR, Koren G. Rates of spontaneous and therapeutic abortions following use of antidepressants in pregnancy: results from a large prospective database. J Obstet Gynaecol Can. 2009;31(5):452-6.

10. Nakhai-Pour HR, Broy P, Berard A. Use of antidepressants during pregnancy and the risk of spontaneous abortion. Cmaj. 2010;182(10):1031-7.

11. Ewing G, Tatarchuk Y, Appleby D, Schwartz N, Kim D. Placental transfer of antidepressant medications: implications for postnatal adaptation syndrome. Clin Pharmacokinet. 2015;54(4):359-70.

12. Anderson HD, Pace WD, Libby AM, West DR, Valuck RJ. Rates of 5 common antidepressant side effects among new adult and adolescent cases of depression: a retrospective US claims study. Clin Ther. 2012;34(1):113-23.

13. Ornoy A, Koren G. Selective Serotonin Reuptake Inhibitors during Pregnancy: Do We Have Now More Definite Answers Related to Prenatal Exposure? Birth Defects Res Part A Clin Mol Teratol. 2017;109(12):898-908.

14. Keyte A, Hutson MR. The neural crest in cardiac congenital anomalies. Differentiation. 2012;84(1):25-40.

15. Marinescu IP, Foarfa MC, Pirlog MC, Turculeanu A. Prenatal depression and stress - risk factors for placental pathology and spontaneous abortion. Rom J Morphol Embryol. 2014;55(3 Suppl):1155-60.

16. Chan J, Natekar A, Einarson A, Koren G. Risks of untreated depression in pregnancy. Can Fam Physician. 2014;60(3):242-3.

For peer review only

17. Kjaersgaard MIS, Parner ET, Vestergaard M, Sorensen MJ, Olsen J, Christensen J, et al. Prenatal Antidepressant Exposure and Risk of Spontaneous Abortion - A Population-Based Study. PLoS ONE. 2013;8(8).
18. Johansen RL, Mortensen LH, Andersen AM, Hansen AV, Strandberg-Larsen K. Maternal use of selective serotonin reuptake inhibitors and risk of miscarriage - assessing potential biases. Paediatr Perinat Epidemiol. 2015;29(1):72-81.
19. Khalifeh H, Molyneaux E, Brauer R, Vigod S, Howard LM. Patient decision aids for antidepressant use in pregnancy: a pilot randomised controlled trial in the UK. BJGP Open. 2019.
20. Xing D, Wu R, Chen L, Wang T. Maternal use of antidepressants during pregnancy and risks for adverse perinatal outcomes: a meta-analysis. Journal of Psychosomatic Research. 2020;137 (no pagination).
21. Udechuku A, Nguyen T, Hill R, Szego K. Antidepressants in pregnancy: a systematic review. Aust N Z J Psychiatry. 2010;44(11):978-96.
22. Ross LE, Grigoriadis S, Mamisashvili L, Vonderporten EH, Roerecke M, Rehm J, et al. Selected pregnancy and delivery outcomes after exposure to antidepressant medication: a systematic review and meta-analysis. JAMA Psychiatry. 2013;70(4):436-43.
23. Higgins JPT, Cochrane Collaboration. Cochrane handbook for systematic reviews of interventions. Second edition. ed. Hoboken, NJ: Wiley-Blackwell; 2020. pages cm p.
24. Sterne JA, Hernan MA, Reeves BC, Savovic J, Berkman ND, Viswanathan M, et al. ROBINS-I: a tool for assessing risk of bias in non-randomised studies of interventions. Bmj. 2016;355:i4919.
25. Higgins JP, Thompson SG, Deeks JJ, Altman DG. Measuring inconsistency in meta-analyses. Bmj. 2003;327(7414):557-60.
26. Lin L, Chu H. Quantifying publication bias in meta-analysis. Biometrics. 2018;74(3):785-94.
27. Chambers CD, Johnson KA, Dick LM, Felix RJ, Jones KL. Birth outcomes in pregnant women taking fluoxetine. N Engl J Med. 1996;335(14):1010-5.
28. Kulin NA, Pastuszak A, Sage SR, Schick-Boschetto B, Spivey G, Feldkamp M, et al. Pregnancy outcome following maternal use of the new selective serotonin reuptake inhibitors: a prospective controlled multicenter study. Jama. 1998;279(8):609-10.
29. Einarson A, Fatoye B, Sarkar M, Lavigne SV, Brochu J, Chambers C, et al. Pregnancy outcome following gestational exposure to venlafaxine: a multicenter prospective controlled study. Am J Psychiatry. 2001;158(10):1728-30.
30. Einarson A, Bonari L, Voyer-Lavigne S, Addis A, Matsui D, Johnson Y, et al. A multicentre prospective controlled study to determine the safety of trazodone and nefazodone use during pregnancy. Canadian Journal of Psychiatry. 2003;48(2):106-10.
31. Sivojelezova A, Shuhaiber S, Sarkissian L, Einarson A, Koren G. Citalopram use in pregnancy: Prospective comparative evaluation of pregnancy and fetal outcome. American Journal of Obstetrics and Gynecology. 2005;193(6):2004-9.
32. Chun-Fai-Chan B, Koren G, Fayez I, Kalra S, Voyer-Lavigne S, Boshier A, et al. Pregnancy outcome of women exposed to bupropion during pregnancy: a prospective comparative study. Am J Obstet Gynecol. 2005;192(3):932-6.
33. Djulus J, Koren G, Einarson TR, Wilton L, Shakir S, Diav-Citrin O, et al. Exposure to mirtazapine during pregnancy: A prospective, comparative study of birth outcomes. J Clin Psychiatry. 2006;67(8):1280-4.

For peer review only

34. Paulus W, Schloemp S, Stoz F. Paroxetine exposure during pregnancy: Increased risk of congenital malformations? Archives of Gynecology and Obstetrics. 2010;282:S79.
35. Chan RL, Jonsson Funk ML, Savitz DA, Hartmann KE. Self-reported antidepressants use and spontaneous abortion. American Journal of Epidemiology. 2011;173:S140.
36. Einarson A, Choi J, Koren G, Einarson T. Outcomes of infants exposed to multiple antidepressants during pregnancy: results of a cohort study. J Popul Ther Clin Pharmacol. 2011;18(2):e390-6.
37. Ban L, Tata LJ, West J, Fiaschi L, Gibson JE. Live and non-live pregnancy outcomes among women with depression and anxiety: A population-based study. PLoS ONE. 2012;7(8).
38. Klieger-Grossmann C, Weitzner B, Panchaud A, Pistelli A, Einarson T, Koren G, et al. Pregnancy outcomes following use of escitalopram: a prospective comparative cohort study. J Clin Pharmacol. 2012;52(5):766-70.
39. Andersen JT, Andersen NL, Horwitz H, Poulsen HE, Jimenez-Solem E. Exposure to selective serotonin reuptake inhibitors in early pregnancy and the risk of miscarriage. Obstet Gynecol. 2014;124(4):655-61.
40. Abadie D, Hurault-Delarue C, Damase-Michel C, Montastruc JL, Lacroix I. Medication exposure and spontaneous abortion: A case-control study using a French medical database. Clinical and Experimental Obstetrics and Gynecology. 2015;42(4):431-6.
41. Te Winkel B, Beghin D, Pistelli A, De Santis M, Cassina M, Eleftheriou G, et al. Venlafaxine exposure in pregnancy, a multicenter ENTIS study. Pharmacoepidemiology and Drug Safety. 2016;25 (Supplement 3):325-6.
42. Almeida ND, Basso O, Abrahamowicz M, Gagnon R, Tamblyn R. Risk of Miscarriage in Women Receiving Antidepressants in Early Pregnancy, Correcting for Induced Abortions. Epidemiology. 2016;27(4):538-46.
43. Evans-Hoeker EA, Eisenberg E, Diamond MP, Legro RS, Alvero R, Coutifaris C, et al. Major depression, antidepressant use, and male and female fertility. Fertility and Sterility. 2018;109(5):879-87.
44. Richardson JL, Martin F, Dunstan H, Greenall A, Stephens S, Yates LM, et al. Pregnancy outcomes following maternal venlafaxine use: A prospective observational comparative cohort study. Reprod Toxicol. 2019;84:108-13.
45. Wu P, Velez Edwards DR, Gorrindo P, Sundermann AC, Torstenson ES, Jones SH, et al. Association between First Trimester Antidepressant Use and Risk of Spontaneous Abortion. Pharmacotherapy. 2019;39(9):889-98.
46. Bahat M, Shechtman S, Diav-Citrin O. Pregnancy outcome after first trimester exposure to Duloxetine. Reprod Toxicol. 2020;97:3.
47. Kolding L, Ehrenstein V, Pedersen L, Sandager P, Petersen OB, Uldbjerg N, et al. Antidepressant use in pregnancy and severe cardiac malformations: Danish register-based study. Bjog. 2021.
48. Ankarfeldt MZ, Petersen J, Andersen JT, Fernandes MFS, Li H, Motsko SP, et al. Duloxetine Exposure During Pregnancy and the Risk of Spontaneous and Elective Abortion: A Danish Nationwide Observational Study. Drugs Real World Outcomes. 2021;8(3):289-99.
49. Ostenfeld A, Petersen TS, Pedersen LH, Westergaard HB, Lokkegaard ECL, Andersen JT. Mirtazapine exposure in pregnancy and fetal safety: A nationwide cohort study. Acta Psychiatr Scand. 2022;145(6):557-67.
50. Kitchin A, Huerta C, Llorente-Garcia A, Martinez D, Ortega P, Cea-Soriano L. The role of prenatal exposure to antidepressants, anxiolytic, and hypnotics and its underlying illness

For peer review only

on the risk of miscarriage using BIFAP database. Pharmacoepidemiol Drug Saf. 2022;31(8):901-12.

51. Giner-Soriano MOP-VMLVCV-CCVAGG-LRM. Spontaneous abortion and drug exposure. Case-control study with SIDIAP database. Pharmaceoepidemiology and drug safety. 2022;Conference: 38th International Conference on Pharmacoepidemiology: Advancing Pharmacoepidemiology and Real-World Evidence for the Global Community. :238-9.

52. Page MJ, McKenzie JE, Bossuyt PM, Boutron I, Hoffmann TC, Mulrow CD, et al. The PRISMA 2020 statement: an updated guideline for reporting systematic reviews. Bmj. 2021;372:n71.

53. Rahimi R, Nikfar S, Abdollahi M. Pregnancy outcomes following exposure to serotonin reuptake inhibitors: a meta-analysis of clinical trials. Reprod Toxicol. 2006;22(4):571-5.

54. Nikfar S, Rahimi R, Hendoiee N, Abdollahi M. Increasing the risk of spontaneous abortion and major malformations in newborns following use of serotonin reuptake inhibitors during pregnancy: A systematic review and updated meta-analysis. Daru. 2012;20(1):75.

55. Kato T, Furukawa TA, Mantani A, Kurata K, Kubouchi H, Hirota S, et al. Optimising first- and second-line treatment strategies for untreated major depressive disorder - the SUND study: a pragmatic, multi-centre, assessor-blinded randomised controlled trial. BMC Med. 2018;16(1):103.

56. Isaac MT. Treating depression with SNRIs: who will benefit most? CNS Spectr. 2008;13(7 Suppl 11):15-21.

57. West SL, Savitz DA, Koch G, Strom BL, Guess HA, Hartzema A. Recall accuracy for prescription medications: self-report compared with database information. Am J Epidemiol. 1995;142(10):1103-12.

58. Hafferty JD, Campbell AI, Navrady LB, Adams MJ, MacIntyre D, Lawrie SM, et al. Self-reported medication use validated through record linkage to national prescribing data. J Clin Epidemiol. 2018;94:132-42.

59. Schisterman EF, Cole SR, Ye A, Platt RW. Accuracy loss due to selection bias in cohort studies with left truncation. Paediatr Perinat Epidemiol. 2013;27(5):491-502.

60. Cain KC, Harlow SD, Little RJ, Nan B, Yosef M, Taffe JR, et al. Bias due to left truncation and left censoring in longitudinal studies of developmental and disease processes. Am J Epidemiol. 2011;173(9):1078-84.

61. Howards PP, Hertz-Picciotto I, Poole C. Conditions for bias from differential left truncation. Am J Epidemiol. 2007;165(4):444-52.

62. Sanchez Ortiz S, Huerta C, Llorente-Garcia A, Ortega P, Astasio P, Cea-Soriano L. A Validation Study on the Frequency and Natural History of Miscarriages Using the Spanish Primary Care Database BIFAP. Healthcare (Basel). 2021;9(5).

For peer review only

For peer review only

PRISMA 2020 flow diagram for new systematic reviews which included searches of databases and registers only

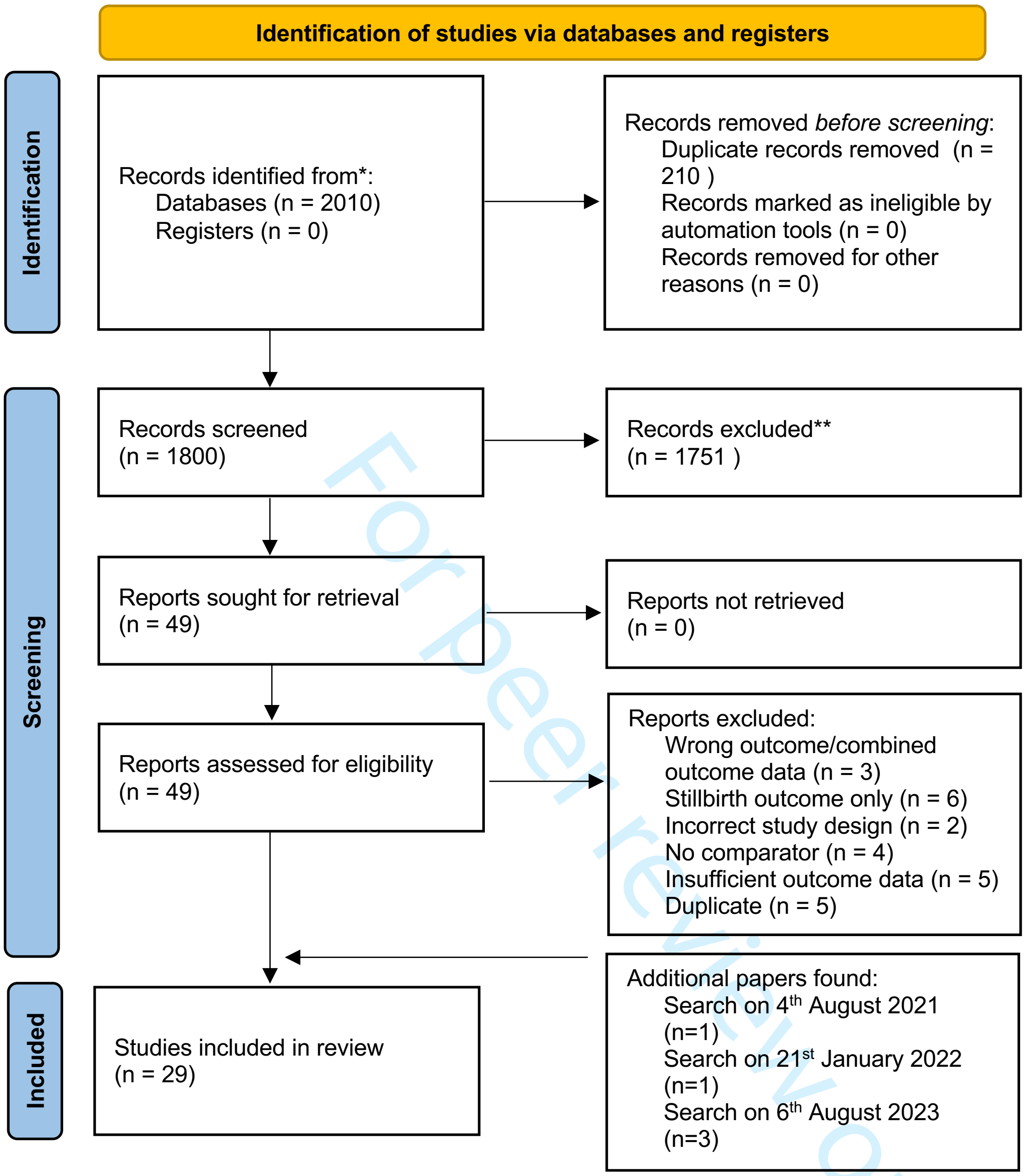

Figure 1. PRISMA flow diagram

Flow diagram illustrating the number of included and excluded studies at each stage of screening, and reasons for exclusion. This version has been adapted to include the element of additional papers found in following searches.

For peer review only

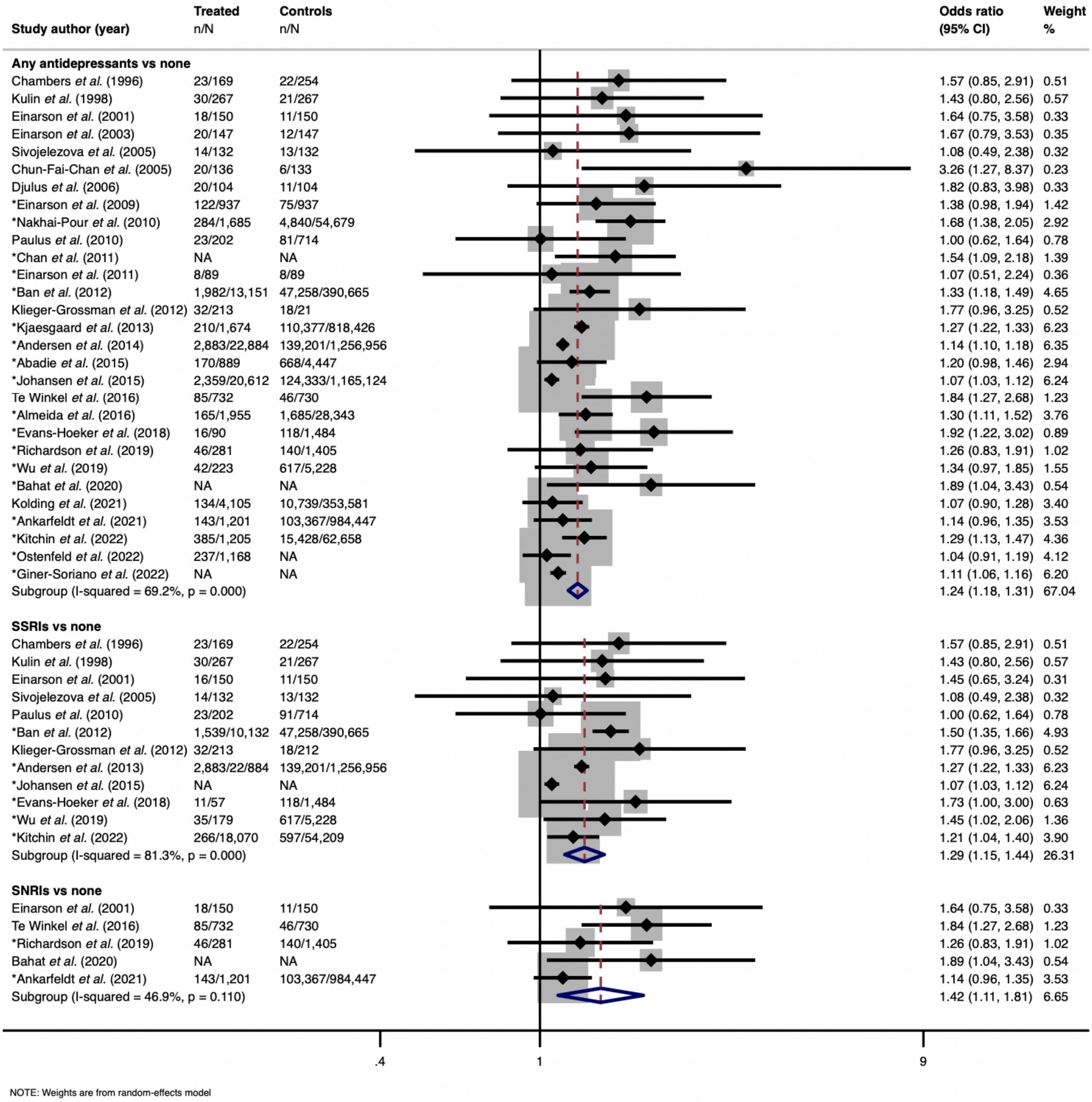

Figure 2. Subgroup meta-analyses utilising general population comparator

Three subgroup analyses conduced. First subgroup assessing any antidepressant use compared to unexposed individuals in general population. Second subgroup comparing SSRI use and unexposed general population individuals. Third subgroup comparing between SNRI use and unexposed general population individuals.

*These studies had an adjusted estimate from multivariable model which was pooled in the meta-analysis rather than raw data, therefore the treated and controls (n/N) data does not correspond to the odds ratio shown.
'The unexposed comparator in this paper was classified as 'duloxetine non-exposed' which could have also included users of other antidepressant types.

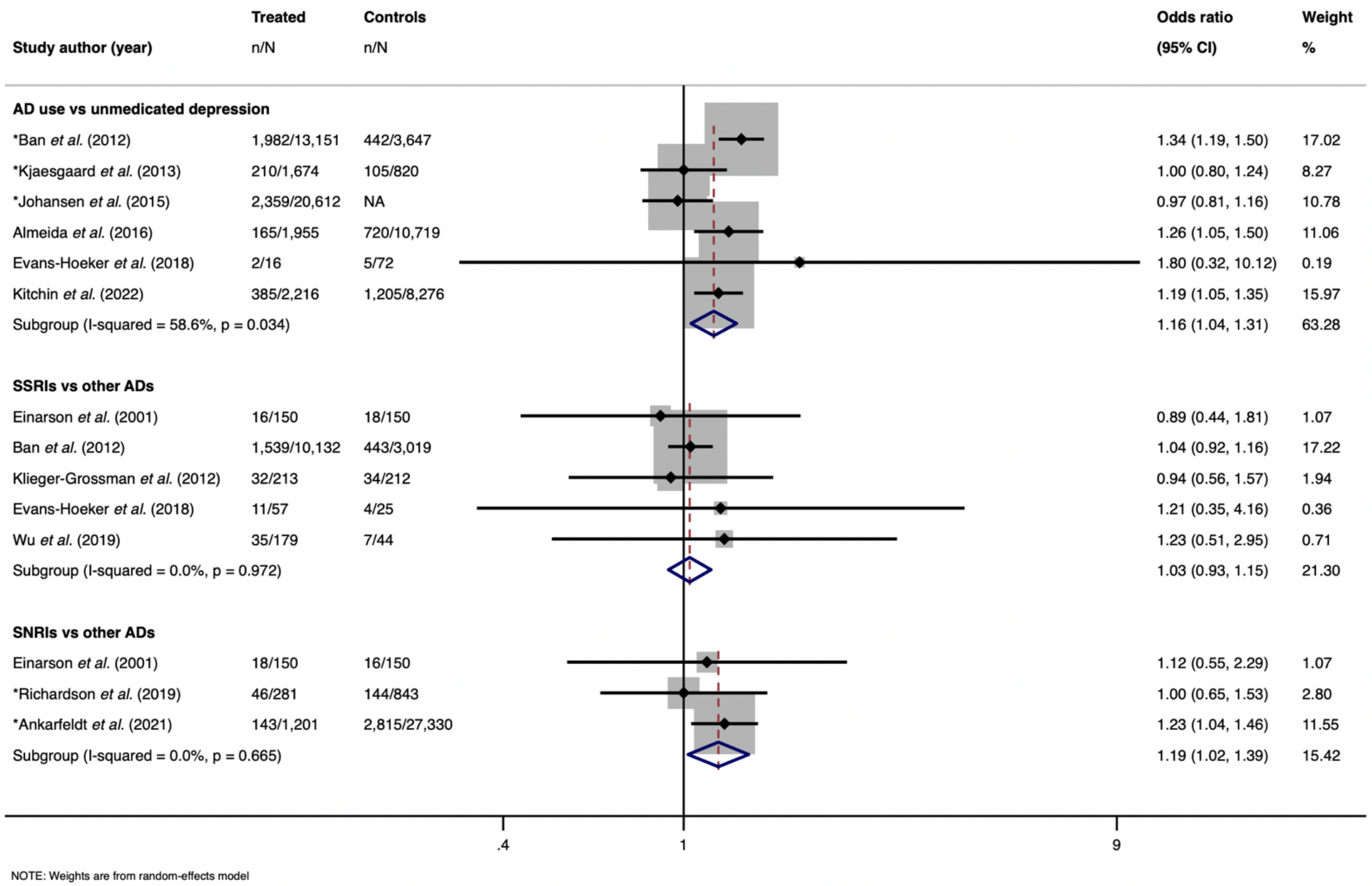

Figure 3. Three random-effects meta-analysis utilising depressed cohort comparator

First subgroup assessing any antidepressant use against unmediated depressed individuals. Second subgroup comparing SSRI use and different types of antidepressants (this includes data from papers which compared SSRIs and SNRIs, SSRIs and TCAs, SSRIs and any other antidepressants). Third subgroup analysis compared SNRI use and different types of antidepressants (this includes data from papers comparing SNRI and SSRI).

* These studies had an adjusted estimate from multivariable model which was pooled in the meta-analysis rather than raw data, therefore the treated and controls (n/N) data does not correspond to the odds ratio shown

For peer review only

EMBASE

Number	Search term	Outputs
1	exp antidepressant agent/	544895
2	Serotonin uptake inhibitor/	52823
3	Monoamine oxidase inhibitor/	15008
4	(anti*depress or antidepress* or tricyclic* or TCA* or heterocyclic* or serotonin uptake or SSRI* or SNRI* or monoamine oxidase inhibitor* or MAOI*).tw.	176810
5	(desipramine or imipramine or clomipramine or opipramol or trimipramine or lofepramine or dibenzepin or amitriptyline or nortriptyline or protriptyline or doxepin or iprindole or melitracen or butriptyline or dosulepin or amoxapine or dimetacrine or amineptine or maprotiline or quinupramine or zimeldine or fluoxetine or citalopram or paroxetine or sertraline or alaproclate or fluvoxamine or etoperidone or escitalopram or isocarboxazid or nialamide or phenelzine or tranylcypromine or iproniazide or iproclozide or moclobemide or toloxatone or oxitriptan or tryptophan or mianserin or nomifensine or trazodone or nefazodone or minaprine or bifemelane or viloxazine or oxaflozane or mirtazapine or bupropion or medifoxamine or tianeptine or pivagabine or venlafaxine or milnacipran or reboxetine or gepirone or duloxetine or agomelatine or desvenlafaxine or vilazodone).tw.	144967
6	1 or 2 or 3 or 4 or 5	673317
7	exp stillbirth/	22575
8	exp perinatal mortality/	27721
9	exp perinatal death/	4865
10	exp fetus death/	43412
11	(stillbirth or stillborn or perinatal death or peri-natal death or neonatal death or intrauterine death or intra-uterine death or newborn mortality).tw.	30264
12	exp spontaneous abortion/	49635
13	recurrent abortion/	7454
14	(Spontaneous abortion or Miscarriage$).tw.	39878
15	7 or 8 or 9 or 10 or 11	80549
16	12 or 13 or 14	62656

For peer review only

17	6 and 15	1176
18	6 and 16	1424
19	17 or 18	2340

MEDLINE

Number	Search term	Output
	exp Antidepressive Agents/	160660
	exp Serotonin Uptake Inhibitors/	46158
	exp Monoamine Oxidase Inhibitors/	22716
	(anti*depress or antidepress* or tricyclic* or TCA* or heterocyclic* or serotonin uptake or SSRI* or SNRI* or monoamine oxidase inhibitor* or MAOI*).tw.	132787
	(desipramine or imipramine or clomipramine or opipramol or trimipramine or lofepramine or dibenzepin or amitriptyline or nortriptyline or protriptyline or doxepin or iprindole or melitracen or butriptyline or dosulepin or amoxapine or dimetacrine or amineptine or maprotiline or quinupramine or zimeldine or fluoxetine or citalopram or paroxetine or sertraline or alaproclate or fluvoxamine or etoperidone or escitalopram or isocarboxazid or nialamide or phenelzine or tranylcypromine or iproniazide or iproclozide or moclobemide or toloxatone or oxitriptan or tryptophan or mianserin or nomifensine or trazodone or nefazodone or minaprine or bifemelane or viloxazine or oxaflozane or mirtazapine or bupropion or medifoxamine or tianeptine or pivagabine or venlafaxine or milnacipran or reboxetine or gepirone or duloxetine or agomelatine or desvenlafaxine or vilazodone).tw.	122965
	1 or 2 or 3 or 4 or 5	304323
	Stillbirth/	6346
	Perinatal Mortality/	2478
	exp Fetal Death/	31204
	PERINATAL DEATH/	2104
	(stillbirth or stillborn or perinatal death or peri-natal death or neonatal death or intrauterine death or intra-uterine death or newborn mortality).tw.	22241
	7 or 8 or 9 or 10 or 11	47887

For peer review only

	exp Abortion, Spontaneous/	38966
	(Spontaneous abortion or Miscarriage$).tw.	24795
	13 or 14	52292
	6 and 12	98
	6 and 15	202
	16 or 17	273

For peer review only

PSYCINFO

	Search terms	Outputs
	exp Antidepressant Drugs/	41562
	exp Serotonin Reuptake Inhibitors/	14269
	exp Monoamine Oxidase Inhibitors/	2318
	(anti*depress or antidepress* or tricyclic* or TCA* or heterocyclic* or serotonin uptake or SSRI* or SNRI* or monoamine oxidase inhibitor* or MAOI*).tw.	50486
	(desipramine or imipramine or clomipramine or opipramol or trimipramine or lofepramine or dibenzepin or amitriptyline or nortriptyline or protriptyline or doxepin or iprindole or melitracen or butriptyline or dosulepin or amoxapine or dimetacrine or amineptine or maprotiline or quinupramine or zimeldine or fluoxetine or citalopram or paroxetine or sertraline or alaproclate or fluvoxamine or etoperidone or escitalopram or isocarboxazid or nialamide or phenelzine or tranylcypromine or iproniazide or iproclozide or moclobemide or toloxatone or oxitriptan or tryptophan or mianserin or nomifensine or trazodone or nefazodone or minaprine or bifemelane or viloxazine or oxaflozane or mirtazapine or bupropion or medifoxamine or tianeptine or pivagabine or venlafaxine or milnacipran or reboxetine or gepirone or duloxetine or agomelatine or desvenlafaxine or vilazodone).tw.	35997
	(stillbirth or stillborn or perinatal death or peri-natal death or neonatal death or intrauterine death or intra-uterine death or newborn mortality).tw.	1195
	exp Spontaneous Abortion/	980
	(Spontaneous abortion or Miscarriage$).tw.	1706
	1 or 2 or 3 or 4 or 5	72741

	7 or 8	1981
	9 and 6	15
	9 and 10	46
	11 or 12	56

For peer review only

S2 Table. Excluded studies

Study excluded at full-text screening	Reason
Lennestal et al. (2007)	Stillbirth outcome only
Sjaarda et al. (2020)	Combined outcome of both stillbirth and miscarriage
Stephansson et al. (2013)	Stillbirth outcome only
Stephens et al. (2011)	Wrong study design
Wen et al. (2006)	Stillbirth outcome only
Winterfeld et al. (2015)	Combined outcome of both stillbirth and miscarriage
Pastuszak et al. (1993)	No outcome data
Ramio et al. (2007)	Wrong study design
Robinson-Wolrath et al. (2016)	No outcome data
Sjaarda et al. (2019)	Duplicate
Andersen et al. (2013)	Duplicate
Burian et al. (2016)	No outcome data
Einarson et al. (2011)	No comparator
Einarson et al. (2009)	Duplicate
Jordan et al. (2016)	Wrong outcome
Einarson et al. (2011)	No comparator
Evans-Hoeker et al. (2017)	Duplicate
Klieger-Grossman et al. (2010)	Duplicate
Goldstein et al. (1997)	No comparator
Hoog et al. (2013)	No comparator
Einarson et al. (2012)	No outcome data
Innocenti et al. (2019)	No outcome data
Jimenez-Solem et al. (2014)	Stillbirth outcome only
Jimenez-Solem et al. (2013)	Stillbirth outcome only
Ericson et al. (1999)	Stillbirth outcome only

For peer review only

Table S3. Characteristics of included studies

Study	Study design	Country of study & study period	Data sampling methods & mean age of individuals	Type of AD exposure	Timing of AD exposure	Definition of exposure and comparator groups	Identification of exposure	Identification of outcome	Sample size	Confounding adjustments	Raw data (no. of SA/ total)	Adjusted estimates
Chambers et al. (1996) [1]	Prospective cohort	California 1989-1995	TIS data collection<br><br>**Exposed early:** 32+-5<br>**Exposed late:** 32+-6<br>**Control:** 30+-5	Fluoxetine (SSRI)	Anytime	**Exposed:** Fluoxetine use<br>**Comparator group 1:** women enquiring about non-teratogens	Telephone interviews	Telephone interview and confirmed by physician	482	Maternal age, average dose of fluoxetine	**Exposed early** = 23/169<br>**Control group** = 22/254	NA
Kulin et al. (1998) [2]	Prospective cohort	US&Canada	TIS data<br><br>**Exposed :** mean = 31.3<br>**Unexposed:** mean = 30.8	SSRI	First trimester	**Exposed:** SSRI use during 1st trimester<br>**Comparator group 1:** Women enquiring about non-teratogens	Telephone interview	After expected date of delivery women were interviewed	534	None	**Exposed** = 30/267<br>**Control** = 21/267	NA
Einarson et al. (2001) [3]	Cohort	European and non-european countries	Women contacting motherisk program around exposure advice<br><br>NA	Venlafaxine (SNRI)	Anytime	**Exposed:** Venlafaxine use during 4th-14th week of pregnancy<br>**Comparator group 1:** SSRI users<br>**Comparator group 2:** women making non-teratogenic enquiries	Once contacted the service women were interviewed	Follow-up questionnaire after delivery and information verified by physician	450	None	**Venlafaxine** = 18/150<br>**SSRI comparator** = 16/150<br>**Nonteratogenic comparator** = 11/150	NA
Einarson et al. (2003) [4]	Prospective cohort	European and non-european countries	Women contacting motherisk program<br><br>NA	Trazodone and Nefazodone (NaSSA)	First trimester	**Exposed:** Use of NaSSAs during 4th-14th week<br>**Comparator group 1:** Women taking other ADs<br>**Comparator group 2**: non-teratogen drug exposures	Questionnaire	Follow-up questionnaire after delivery and verified by physician	441	Matched on gestational age	**Trazodone/Nefazodone** = 20/147<br>**Group 1 (other Ads)** = 17/147<br>**Group 2 – nonteratogens** = 12/147	NA
Sivojelezova et al. (2005) [5]	Prospective cohort	Toronto 1999-2002	Women contacting motherisk program<br><br>Range of 18-42 and mean of 31.9	Citalopram (SSRI)	Anytime	**Exposed:** Use of citalopram<br>**Comparator group 1:** pregnant women taking other SSRI Ads<br>**Comparator group 2:** non-teratogenic drug use	Telephone interview to individuals contacting the service	Follow-up questionnaire after delivery and verified by physician	396	None	**Exposed** = 14/132<br>**Comparison 1** = 13/132<br>**Comparison 2** = 13/132	NA

For peer review only

Chun-Fai Chan et al. (2005) [6]	Cohort	European and non-european countries	Individuals contacting motherisk or recruited through drug safety research unit<br><br>NA	Bupropion (NDRI)	Anytime	**Exposed:** Bupropion exposed pregnancies<br>**Comparator group 1:** Pregnant women taking other Ads<br>**Comparator group 2:** individuals taking non-teratogens	Telephone administration of follow-up questionnaire	Follow-up questionnaire after delivery and verified by physician	NA	Matched on age, alcohol consumption, smoking and gestational age	**Bupropion =** 20/136<br>**Comparison 1 (other Ads) =** 11/89<br>**Comparison 2 (nontertaogens) =** 6/133<br>**Women exposed to bupropion for depression =** 14/91	NA
Djulus et al. (2006) [7]	Cohort	European and non-european countries 2002-2005	Pregnant women contacting one of five evidence based information centres<br><br>Ages were consistent between groups due to matching	Mirtazapine (NaSSA)	Anytime	**Exposed:** pregnant women taking bupropion<br>**Comparator group 1:** disease matched individuals taking other Ads<br>**Comparator group 2:** individuals taking known non-teratogens	Telephone interview regarding exposure	Contacted 2-6 months after delivery and details verified by physician	312	Matched on maternal age, gestational age, tobacco use, alcohol consumption and chronic conditions	**Mirtazapine =** 20/104<br>**Comparison 1 (other AD) =** 18/104<br>**Comparison 2 (nonteratogenics)** = 11/104	NA
Einarson et al. (2009) [8]	Prospective cohort	Toronto	women contacting motherisk program<br><br>NA	Any AD	Anytime	**Exposed:** Pregnant women contacting program regarding exposure during pregnancy<br>**Comparator group 1:** Women exposed to non-teratogenic drugs<br>**Comparator group 2:** unexposed to Ads	Telephone interview using standardised questionnaire	Follow-up structured form	1874	Matched for maternal age, smoking, and alcohol use, gestational age	**Exposed =** 122/937<br>**Unexposed =** 75/937	RR = 1.38 (0.98-1.94)
Nakhai-Pour et al. (2010) [9]	Nested case-control	Quebec 1998-2003	Data from Quebec pregnancy register with individuals registered between 1998-2003, including RAMQ database<br><br>NA	Any AD	Any time between first day gestation and index date of SA	**Exposed:** At least one prescription filled between start of gestation and SA index date<br>10 controls sampled for every SA case	RAMQ database which collects information on prescriptions, and pregnancy register to identify unexposed	Quebec pregnancy register entered between 1998-2003	56364	Adjusted for age, urban residence, social assistance, psychiatric diagnosis, comorbid illness, medication use, gestational age	**Cases** = 284 individuals used antidepressants<br>**Controls =** 1401 used antidepressants	**Crude OR =** 2.09 (1.83-2.38)<br>**Adjusted OR =** 1.68 (1.38-2.06)<br>**Adjusted SSRI alone** = 1.61 (1.28-2.04)<br>**Adjusted TCA alone** = 1.27 (0.85-1.91)
Paulus et al. (2010) [10]	Prospective cohort: Conference abstract	Germany	National TIS who were contacted by	Paroxetine (SSRI)	First trimester	**Exposed:** Paroxetine exposure in first trimester	Patients or physicians contact TIS	NA	955	None	**Exposed** = 23/202<br>**Unexposed =** 81/714	NA

For peer review only

			physicians or patients			**Comparator group 1:** Not exposed or 'severely exposed' to paroxetine						
Chan et al. (2011) [11]	Cohort – conference abstract	US<br>2000-2008	Recruited from 4 US metropolitan areas	All Ads	Anytime	**Exposed:** Antidepressant use during pregnancy<br>**Comparator:** NA	Telephone interviews and medical record abstractions	Telephone interviews and medical record abstractions	4536	Maternal age, race, education, BMI, smoking, alcohol use, gravidity and nausea	NA	**Adjusted OR of exposed vs unexposed** = 1.54 (1.1-2.2)<br>**Adjusted OR of SSRI users vs unexposed** = 1.67
Einarson et al. (2011) [12]	Prospective cohort	Toronto<br>1992-2007	women contacting motherisk	All Ads	Anytime	**Exposed:** exposed to more than one AD during pregnancy<br>**Comparator group 1:** Exposed to single AD during pregnancy<br>**Comparator group 2:** contacting motherisk regarding non-teratogenic exposure	telephone interview using standardised questionnaire	Follow-up interview details verified from report by physician	267	Matched on maternal age, smoking and alcohol use and gestational age	**Multiple AD exposure** = 8/89<br>**Single AD exposure** = 7/89<br>**No AD exposure** = 8/89	**OR for multiple AD exposure vs single exposure** = 1.16 (0.4-3.34)<br>**OR for multiple AD exposure vs no exposure** = 1.00 (0.36-2.79)
Ban et al. (2012) [13]	Cohort	UK<br>1990-2009	Data collected from primary care medical records (through health improvement network - THIN)<br><br>> 50% of the women aged between 25-34	TCAs and SSRIs	First trimester	**Exposed:** depression and exposed to AD during first 90 days after conception<br>**Comparator group 1L** No history of depression and unexposed<br>**Comparator group 2:** history of depression and unexposed<br>**Comparator group 3:** prescriptions for TCAs only<br>**Comparator group 4:** prescriptions for SSRIs only<br>**Comparator group 5:** prescriptions for any other AD only<br>**Comparator group 6:** prescription for psychotropic drugs only	Extracted prescription records from THIN	Identified through THIN database	331414	Adjusted for maternal age, SES, maternal smoking status and BMI	**Reference category (group 1)** = 47258/390665<br>**History of mental illness only** = 13814/99932<br>**Unmedicated current mental illness** = 442/3647<br>**TCAs** = 443/3019<br>**SSRIs**= 1539/10132	**Adjusted RRR with comparison being no current/past depression or anxiety**<br>**History of mental illness only:** 1.2 (1.2-1.2)<br>**Unmedicated mental illness** = 1.0 (0.9-1.2)<br>**TCAs** = 1.3 (1.1-1.5)<br>**SSRIs** = 1.5 (1.3-1.6)<br><br>**Adjusted RRR when compared to unmedicated antenatal depression or anxiety**<br>**TCAs** = 1.3 (1.1-1.5)<br>**SSRIs** = 1.4 (1.2-1.7)
Klieger-Grossman et al. (2012) [14]	Prospective cohort	Toronto, Switzerland and Florence	Data collected from women ringing TIS in the three countries	Escitalopram (SSRI)	Anytime	**Exposed:** pregnant women exposed to escitalopram<br>**Comparator group 1:** contacting service exposed to other Ads	Information taken during telephone conversation through standardised questionnaire	Follow-up questionnaire 2-3 months after delivery	637	Matched on maternal age, alcohol consumption, smoking and gestational age	**Escitalopram** = 32/213<br>**Other AD use** = 34/212<br>**Nonteratogens** = 18/212	NA

For peer review only

For peer review only - http://bmjopen.bmj.com/site/about/guidelines.xhtml

			Mean age of all women in study = 33.1			**Comparator group 2:** nonteratogenic exposures						
Kjaesgaard et al. (2013) [15]	Population based cohort	Denmark 1997-2008	Data obtained from Danish health registries<br><br>**Depression and no AD use:** mean = 29.1<br>**Use of AD (exposed):** mean = 30.9<br>**Unexposed:** mean = 30.2	All Ads	Anytime	**Exposed:** mother redeemed prescription for AD<br>**Comparator group 2:** mother hadn't redeemed prescription	Information from registry of medicinal product statistics	Identified through codes using Danish national hospital registry	1005319	Adjusted for maternal age, cohabitation, income, education, history of mental disorder or drug abuse	**Diagnosis of depression and exposed to AD =** 210/1674<br>**Diagnosis of depression and unexposed to AD=** 105/820<br>**No diagnosis of depression and AD use=** 2427/13789<br>**No diagnosis of depression and unexposed=** 110377/818426	**<u>Unadjusted RR when compared to unexposed equivalent:</u>**<br>**Diagnosis of depression exposed to AD =** 0.98 (0.78-1.23)<br>**No diagnosis of depression and AD use =** 1.31 (1.26-1.35)<br><br>**<u>Adjusted RR (when compared to unexposed equivalent):</u>**<br>**Diagnosis of depression and exposed =** 1.00 (0.8-1.24)<br>**No diagnosis of depression and AD use =** 1.17 (1.13-1.22)
Andersen et al. (2014)[16]	Cohort	Denmark 1997-2010	Identified all registered pregnancies through national hospital register	SSRIs	First 35 days of pregnancy	**Exposed:** Dispensing of prescription of SSRIs<br>**Comparator group 1:** Women exposed to SSRIs prior to pregnancy<br>**Comparator group 2:** women unexposed to SSRIs	Data collected from national prescription register containing individual level data on prescribed drugs	Identified through national hospital register using codes	1279840	Adjusted for maternal age, previous SA, income, year of outcome, educational length, antipsychotics and thyroid preparations	**Unexposed=** 139201/1256956<br>**Exposed to SSRI during first 35 days of pregnancy:** 2883/22884<br>**Discontinuation of SSRI treatment 3-12 months before conception =** 1936/14016	**<u>Unadjusted HR:</u>**<br>**Exposed during first 35 days vs unexposed:** 1.31 (1.27-1.36)<br>**Discontinued SSRI treatment 3-12 months before pregnancy vs unexposed:** 1.38 (1.32-1.44)<br><br>**<u>Adjusted HR:</u>**<br>**Exposed to SSRI during first 35 days vs unexposed:** 1.27(1.22-1.33)<br>**Discontinued use of SSRI before conception vs unexposed:** 1.24 (1.18-1.30)

For peer review only

Abadie et al. (2015)[17]	Nested case-control	France	French database of TERAPPEL used where healthcare professionals contact French regional pharmacovigilance centres (CPRV)<br><br>**Mean of cases** = 32.4<br>**Mean of controls** = 30.6	All Ads	First trimester	**Exposed:** First trimester drug exposure and called CPRV enquiring about drug exposure<br>**Comparator group 1:** Calling CPRV for assessing non-teratogen exposure	Healthcare professionals contacting CPRVs	Women registered to TERAPPEL database	5346	Maternal age	**Exposed to any AD**<br>**Cases of SA** = 170/838<br>**No SA** = 729/4508<br><br>**Exposed to non-selective monoamine reuptake inhibitors (NMRI)**<br>**SA** = 39/838<br>**No SA** = 88/4508	**Crude OR for all Ads** = 1.32 (1.1-1.59)<br>**Adjusted OR for all Ads** = 1.2 (0.98-1.46)<br><br>**Crude OR for NMRI**= 2.45 (1.67-3.60)<br>**Adjusted OR for NMRI** = 2.19 (1.46-2.39)
Johansen et al. (2015) [18]	Register based study cohort	Denmark 1996-2009	study population with registered pregnancies between 1996-2009 and Danish national birth cohort	SSRI	Anytime but results split into first and second trimester	**Exposed:** Redeemed prescription in time frame of month prior to LMP to 3 months after LMP or end of pregnancy (also self-report)<br>**Comparator group 1:** discontinued use of SSRIs with same LMP prescription timeframe<br>**Comparator group 2:** unexposed and no prescription	Redeemed prescriptions from Danish national prescription database	Data from Danish national patient register	1281418	Adjustment for household income, education, previous SA, parity and maternal age	**Unexposed** = 124333/1165124<br>**Discontinued use** = 670/5428<br>**Exposed** = 2359/20612	**Combined first and second trimester HR**<br><br>**Exposed vs unexposed general population** = 1.07 (1.03-1.12)<br>**SSRI use vs unmedicated depressed** = 0.97 (0.81-1.16)
Te Winkel et al. (2016) [19]	Prospective cohort – conference abstract only	Across European countries	Data from nine centres which make up european network of TIS<br><br>Mean: 31.9	Venlafaxine (SSRI)	Anytime	**Exposed:** Venlafaxine exposure<br>**Comparator group 1:** No exposure to any known teratogens	TIS data	using standardised procedures for data collection and follow-up	1462	None	**Exposed** = 85/732<br>**Unexposed** = 46/730	NA
Almeida et al. (2016) [20]	Retrospective cohort	Quebec 1998-2002	Linking four population-based registries and producing longitudinal histories of medication use	All Ads	First trimester	**Exposed:** Women who have at least one AD prescription in first trimester of pregnancy<br>**Comparator groups 1:** Unexposed but depressed in last four years<br>**Comparator group 2:** non-teratogen group	Prescription claims database	ICD-9 codes	41964	Adjustment for maternal age, welfare recipient, other teratogenic medication, no. medications any hospitalisatio	**No medication and no depression:** 1685/28343<br>**No AD but depression** - 720/10719<br>**AD use in first trimester and depression** - 165/1955	**All adjusted RRR**<br><br>**Unexposed and no depression** = 1.00<br>**Unexposed and depressed** = 1.1 (1.0-1.2)<br>**Exposed and depressed** = 1.3 (1.1-1.5)

For peer review only

						**Comparator group 3:** individuals with no prescriptions or depression				n or physician visits, calendar year	**Hypothyroid medication use in first trimester:** 71/947	
Evans-Hoeker et al. (2018) [21]	Cohort	US	RCT was conducted on non-IVF fertility treatment and individuals from trial were brought into cohort study	All Ads – split into SSRI and non-SSRI	Anytime	**Group 1:** Absence of major depression(MD) and no AD use<br>**Group 2:** Absence of MD and using AD<br>**Group 3:** Presence of MD and no AD use<br>**Group 4:** MD & using Ads	Medication exposure assessed through RCT and PH-9 questionnaire used	Identification through RCT	1650	Adjusted for age, race, income, current smoking, depression severity and trial placed in for RCT	**PH-9<10 and unexposed =** 110/1359<br>**PH-9<10 and exposed =** 14/73<br>**PH-9>10 and unexposed =** 5/72<br>**PH-9>10 and exposed =** 2/16<br><br>**No AD use =** 118/1484<br>**Any AD use =** 16/90<br>**SSRI use =** 11/57<br>**Non-SSRI use=** 4/25<br>**Both use of SSRI and non-SSRI =** 1/8	**Adjusted RR:**<br>**Ph-9<10 and unexposed =** reference<br>**PH-9<10 and exposed =** 1.87 (1.18-2.99)<br>**PH-9>10 and unexposed =** 0.85 (0.37-1.96)<br>**PH-9>10 and exposed =** 1.4 (0.47-4.17)<br><br>**specific medication types:**<br>**Any AD use =** 1.92 (1.22-3.02)<br>**SSRI use =** 1.73 (1.0-3.0)<br>**Non-SSRI use=** 3.45(1.99-5.98)<br>**Both SSRI and non-SSRI =** 1.15 (0.19-7.06)
Richardson et al. (2019) [22]	Cohort	UK	Using UK collected teratogen surveillance data<br><br>Median: 31 for both groups	Venlafaxine (SNRI)	Anytime	**Exposed:** Individuals using venlafaxine at any point during pregnancy<br>**Comparator group 1:** Unexposed to any AD medication (5:1)<br>**Comparator group 2:** disease matched comparator of SSRI AD exposed	Surveillance data from UKTIS	Postal questionnaire sent after delivery date from healthcare professional who contacted UKTIS	2529	Adjusted for history of SA, tobacco exposure, alcohol, recreational drugs and folic acid	**Venlafaxine =** 46/281<br>**SSRI exposed =** 114/843<br>**Unexposed =** 140/1405	**Crude OR for unexposed =** 1.57 (1.06-2.30)<br>**Crude OR for SSRI comparator =** 1.05 (0.7-1.57)<br><br>**Adjusted HR for unexposed =** 1.26 (0.829-1.91)<br>**Adjusted HR for SSRI comparator =** 1.00 (0.655-1.53)
Wu et al. (2019) [23]	Prospective cohort	North Carolina, Tennessee and Texas	Enrolment into community-based cohort<br><br>NA	All AD – classified into SSRI and non-SSRI	First trimester	**Exposed:** reported AD use since pregnancy and before interview<br>**Comparator group 1:** Never used AD	community-based cohort – AD use assessed during interview	Self-reported pregnancy outcomes-verified by medical records	5451	Adjustment for maternal age, BMI, caffeine consumption, smoking, alcohol, race, SA history	**Exposed to any AD =** 42/223<br>**Use of SSRIs =** 35/179<br>**Exposure to non-SSRIs =** 7/44<br>**Unexposed =** 617/5228	**Unadjusted HR:**<br><br>**Any AD =** 1.51 (1.10-2.06)<br>**Only SSRIs =** 1.59 (1.13-2.23)<br>**Only non-SSRIs = 1.21** (0.57-2.56)

For peer review only

												**Adjusted HR**<br>**Any AD use:** 1.34 (0.97-1.85)<br>**SSRI only:** 1.45 (1.02-2.06)<br>**Non-SSRI only:** 0.96 (0.45-2.04)
Bahat et al. (2020) [24]	Cohort – conference abstract only	Israel 2001-2015	Data utilised from Israeli TIS<br><br>NA	Duloxetine (SNRI)	First trimester	**Exposed:** Exposure to duloxetine during first trimester<br>**Comparator group 1:** women exposed to TCAs<br>**Comparator group 2:** non-teratogenic exposure	Information taken when contacted TIS	Identified through follow-up after delivery date	722	None	NA	**Adjusted HR =** 1.89 (1.04-3.43)
Kolding et al. (2021)[25]	Register based study	Denmark 2007-2014	Routinely collected data from five nationwide registries and databases<br><br>NA	All Ads	First trimester	**Exposed:** two or more redeemed prescriptions<br>**Comparator group 1:** Absence of redemptions for AD	Using redeemed prescriptions through linkage to Danish health services prescription database	Identified through Danish national patient registry	364012	None	**Exposed =** 134/3971<br>**Unexposed =** 10739/342842	NA
Ankarfeldt et al. (2021)[26]	Prospective cohort	Denmark 2004-2016	Study population used danish registry data whereby women were included if they had a recorded birth in birth register or an abortion in patient register between 2004-2016<br><br>NA	Duloxetine (SNRI)<br>SSRIs<br>Venlafaxine (SNRI)	Anytime during pregnancy	**Exposed** = redemption of AD prescription in window from 30 days prior to LMP to 140 days post LMP or end of pregnancy, whichever came first<br>**Comparator group 1:** Duloxetine non-exposed (no redemption of prescription during the window)<br>**Comparator group 2:** SSRI exposed<br>**Comparator group 3:** Venlafaxine exposed	Using the Danish national prescription register	Identified through either the birth registry for all births, or the patient register for abortions.	1020288	birth year, maternal age, psychiatric hospitalization, household income, education, comorbidities, co-medication	**Unadjusted:**<br>**Duloxetine =** 145/1212<br>**Duloxetine unexposed =** 106309/1018745<br>**SSRI =** 2900/28345<br>**Venlafaxine =** 497/4908	**Adjusted**<br><br>**Duloxetine vs unexposed** = 1.14 (0.96-1.34)<br>**Duloxetine vs SSRI =** 1.23 (1.04-1.46)<br>**Duloxetine vs venlafaxine =** 1.18 (0.98-1.42)<br><br>**PS-matched**<br><br>**duloxetine vs unexposed** = 1.08 (0.89-1.31)<br>**Duloxetine vs SSRI =** 1.25 (1.00-1.57)<br>**Duloxetine vs venlafaxine** = 1.08(0.82-1.41)

Ads=antidepressants, SSRIs = selective serotonin reuptake inhibitors, SNRIs= serotonin-norepinephrine reuptake inhibitors, TCAs= tricyclic antidepressants, NDRI = norepinephrine and dopamine reuptake inhibitors, NaSSA = noradrenergic and serotonergic specific antidepressants, RCT= randomized controlled trial, TIS=teratogen information service, NA= not available, MD = major depression

For peer review only

For peer review only - http://bmjopen.bmj.com/site/about/guidelines.xhtml

Kitchin et al. (2022)[27]	Nested case-control	Spain<br><br>2002-2015	Cohort included women of childbearing age (15-49) with at least 1 year of registration with their primary care practitioner between Jan 2002 and Dec 2015.	All ADs	Anytime during pregnancy	**nonusers:** when there were no prescriptions in any of these periods<br><br>**discontinuers:** women who had at least one prescription only during the prepregnancy period;<br><br>**continuers:** women who had at least one prescription during the prepregnancy period and also during the first trimester<br><br>**new users:** women who had at least one pre- scription only during the first trimester.	Spanish database BIFAP	Miscarriage identified via ICD codes in the BIFAP database	72,280	Number of GP visits, obesity, smoking, HTA and diabetes	**Discontinued AD use vs no use:** 2216/8276 vs 15428/62658, **Continued AD use vs no use:** 385/1205 vs 15428/62658, **Incident AD use vs no use:** 41/140 vs 15428/62658	**ORs - Discontinued AD use vs no use:** 1.05 (1.00-1.11) **Continued AD use vs no use:** 1.29 (1.13-1.46) **Incident AD use vs no use:** 1.12 (0.78-1.62)
Ostenfeld et al. (2022)[28]	Cohort	Denmark 1997-2016	Study population using Danish registry data – with the study population being all pregnancies registered in the medical birth register or national hospital register (between 1st jan 1997 to 31st Dec 2016)<br><br>NA	Mirtazapine (NaSSA)	Exposure earlier than 22 weeks of completed gestation	**Exposed:** One filled prescription of mirtazapine	Using the register for of medicinal product statistics.	Identified in the National Hospital Register of Denmark	9,500	Age at pregnancy onset, marital status, place of birth, region of residence, gross household income, education status, parity, multiple birth pregnancy, smoking during pregnancy, previous pregnancy with adverse fetal outcome, prescription drug use in past year and hospital care utilization in past year	**Mirtazepine exposed vs unexposed:** 237/1168	**HRs: Mirtazepine exposed vs unexposed:** 1.04 (0.91-1.20)

For peer review only

Giner-Soriano et al. (2022)[29]	Case-control	Spain	Pregnancy episodes of women aged 12-50 from SIDIAP database during 2011-2020<br><br>NA	NA	Exposure up to 120 days after pregnancy start date or at SA date	**Exposure:** dispensation of AD 30 days before to 120 days after pregnancy start date or at spontaneous abortion date, whichever occurs first	SIDIAP – contains primary care health data records	Primary healthcare records of the Catalan Health Institute	180,692	NA	NA	**ORs - antidepressants vs no antidepressants:** 1.11 (1.06-1.16)

For peer review only

For peer review only - http://bmjopen.bmj.com/site/about/guidelines.xhtml

References

1. Chambers CD, Johnson KA, Dick LM, Felix RJ, Jones KL. Birth outcomes in pregnant women taking fluoxetine. *N Engl J Med* 1996; **335**(14): 1010-5.

2. Kulin NA, Pastuszak A, Sage SR, et al. Pregnancy outcome following maternal use of the new selective serotonin reuptake inhibitors: a prospective controlled multicenter study. *Jama* 1998; **279**(8): 609-10.

3. Einarson A, Fatoye B, Sarkar M, et al. Pregnancy outcome following gestational exposure to venlafaxine: a multicenter prospective controlled study. *Am J Psychiatry* 2001; **158**(10): 1728-30.

4. Einarson A, Bonari L, Voyer-Lavigne S, et al. A multicentre prospective controlled study to determine the safety of trazodone and nefazodone use during pregnancy. *Canadian Journal of Psychiatry* 2003; **48**(2): 106-10.

5. Sivojelezova A, Shuhaiber S, Sarkissian L, Einarson A, Koren G. Citalopram use in pregnancy: Prospective comparative evaluation of pregnancy and fetal outcome. *American Journal of Obstetrics and Gynecology* 2005; **193**(6): 2004-9.

6. Chun-Fai-Chan B, Koren G, Fayez I, et al. Pregnancy outcome of women exposed to bupropion during pregnancy: a prospective comparative study. *Am J Obstet Gynecol* 2005; **192**(3): 932-6.

7. Djulus J, Koren G, Einarson TR, et al. Exposure to mirtazapine during pregnancy: A prospective, comparative study of birth outcomes. *J Clin Psychiatry* 2006; **67**(8): 1280-4.

8. Einarson A, Choi J, Einarson TR, Koren G. Rates of spontaneous and therapeutic abortions following use of antidepressants in pregnancy: results from a large prospective database. *J Obstet Gynaecol Can* 2009; **31**(5): 452-6.

9. Nakhai-Pour HR, Broy P, Berard A. Use of antidepressants during pregnancy and the risk of spontaneous abortion. *Cmaj* 2010; **182**(10): 1031-7.

10. Paulus W, Schloemp S, Stoz F. Paroxetine exposure during pregnancy: Increased risk of congenital malformations? *Archives of Gynecology and Obstetrics* 2010; **282**: S79.

11. Chan RL, Jonsson Funk ML, Savitz DA, Hartmann KE. Self-reported antidepressants use and spontaneous abortion. *American Journal of Epidemiology* 2011; **173**: S140.

12. Einarson A, Choi J, Koren G, Einarson T. Outcomes of infants exposed to multiple antidepressants during pregnancy: results of a cohort study. *J Popul Ther Clin Pharmacol* 2011; **18**(2): e390-6.

13. Ban L, Tata LJ, West J, Fiaschi L, Gibson JE. Live and non-live pregnancy outcomes among women with depression and anxiety: a population-based study. *PLoS ONE* 2012; **7**(8): e43462.

14. Klieger-Grossmann C, Weitzner B, Panchaud A, et al. Pregnancy outcomes following use of escitalopram: a prospective comparative cohort study. *J Clin Pharmacol* 2012; **52**(5): 766-70.

For peer review only

15. Kjaersgaard MI, Parner ET, Vestergaard M, et al. Prenatal antidepressant exposure and risk of spontaneous abortion - a population-based study. *PLoS ONE* 2013; **8**(8): e72095.
16. Andersen JT, Andersen NL, Horwitz H, Poulsen HE, Jimenez-Solem E. Exposure to selective serotonin reuptake inhibitors in early pregnancy and the risk of miscarriage. *Obstet Gynecol* 2014; **124**(4): 655-61.
17. Abadie D, Hurault-Delarue C, Damase-Michel C, Montastruc JL, Lacroix I. Medication exposure and spontaneous abortion: A case-control study using a French medical database. *Clinical and Experimental Obstetrics and Gynecology* 2015; **42**(4): 431-6.
18. Johansen RL, Mortensen LH, Andersen AM, Hansen AV, Strandberg-Larsen K. Maternal use of selective serotonin reuptake inhibitors and risk of miscarriage - assessing potential biases. *Paediatr Perinat Epidemiol* 2015; **29**(1): 72-81.
19. Te Winkel B, Beghin D, Pistelli A, et al. Venlafaxine exposure in pregnancy, a multicenter ENTIS study. *Pharmacoepidemiology and Drug Safety* 2016; **25 (Supplement 3)**: 325-6.
20. Almeida ND, Basso O, Abrahamowicz M, Gagnon R, Tamblyn R. Risk of Miscarriage in Women Receiving Antidepressants in Early Pregnancy, Correcting for Induced Abortions. *Epidemiology* 2016; **27**(4): 538-46.
21. Evans-Hoeker EA, Eisenberg E, Diamond MP, et al. Major depression, antidepressant use, and male and female fertility. *Fertility and Sterility* 2018; **109**(5): 879-87.
22. Richardson JL, Martin F, Dunstan H, et al. Pregnancy outcomes following maternal venlafaxine use: A prospective observational comparative cohort study. *Reprod Toxicol* 2019; **84**: 108-13.
23. Wu P, Velez Edwards DR, Gorrindo P, et al. Association between First Trimester Antidepressant Use and Risk of Spontaneous Abortion. *Pharmacotherapy* 2019; **39**(9): 889-98.
24. Bahat M, Shechtman S, Diav-Citrin O. Pregnancy outcome after first trimester exposure to Duloxetine. *Reprod Toxicol* 2020; **97**: 3.
25. Kolding L, Ehrenstein V, Pedersen L, et al. Antidepressant use in pregnancy and severe cardiac malformations: Danish register-based study. *Bjog* 2021; **128**(12): 1949-57.
26. Ankarfeldt MZ, Petersen J, Andersen JT, et al. Duloxetine Exposure During Pregnancy and the Risk of Spontaneous and Elective Abortion: A Danish Nationwide Observational Study. *Drugs Real World Outcomes* 2021; **8**(3): 289-99.
27. Kitchin Á, Huerta C, Llorente-García A, Martínez D, Ortega P, Cea-Soriano L. The role of prenatal exposure to antidepressants, anxiolytic, and hypnotics and its underlying illness on the risk of miscarriage using BIFAP database. Pharmacoepidemiology and Drug Safety. 2022 Aug;31(8):901-12.
28. Ostenfeld A, Petersen TS, Pedersen LH, Westergaard HB, Løkkegaard EC, Andersen JT. Mirtazapine exposure in pregnancy and fetal safety: A nationwide cohort study. Acta Psychiatrica Scandinavica. 2022 Jun;145(6):557-67.

For peer review only

29. Giner-Soriano M, Prat-Vallverdu O, Vazquez ML, Vilaplana-Carnerero C, Vedia C, Gomez-Lumbreras AG, Morros R. Spontaneous abortion and drug exposure. Case-control study with SIDIAP database. InPHARMACOEPIDEMIOLOGY AND DRUG SAFETY 2022 Sep 1 (Vol. 31, pp. 238-239). 111 RIVER ST, HOBOKEN 07030-5774, NJ USA: WILEY.

For peer review only

S4 Table. Risk of bias assessment

Study	Domain 1: Bias due to confounding	Domain 2: Bias in classification of intervention	Domain 3: Bias in selection of participants into study	Domain 4: Bias due to deviations from intended interventions	Domain 5: Bias due to missing data	Domain 6: Bias in measurement of the outcome	Domain 7: Bias in selection of the reported result	Overall RoB rating
Chambers et al (1996)	**Critical** No confounding adjustments made	**Critical**	**Critical**	**Critical**	**Critical**	**Critical**	**Critical**	**Critical**
Kulin et al (1998)	**Critical** No information on confounding adjustments	**Critical**	**Critical**	**Critical**	**Critical**	**Critical**	**Critical**	**Critical**
Einarson et al (2001)	**Serious** Justification: The paper only adjusted for maternal depression, therefore not adjusting for all three of the key confounding domains	**Serious** Justification: Exposure was determined through self-report of the individual. This was measured prospectively whereby the outcome had not occurred so would be unlikely to affect the exposure classification, however this was not verified through physician or healthcare records. Therefore, intervention status could not have been classified correctly and could be differential	**Serious:** Justification: The start of follow-up and intervention are not at the same point as women ringing the service enquiring about an exposure could have been taking it for weeks prior to call, which could induce bias as the intervention has differing effects over time, and no analytical techniques were put in place to minimise this bias	**Low** Justification: Study was not undergone in an experimental context	**Serious** Justification: Although complete case analysis was used, the number of exposed individuals and total sample was not stated. Therefore, there could have been missing data and there was no evidence to show that the missing data of outcome was not associated with the true value of the outcome	**Low** Justification: The outcome was measured through questionnaire to individual, alongside the results being verified by a physician. Although the outcome assessors could have been aware of exposure status, this is unlikely to have influenced outcome classification.	**Moderate** Justification: No statistical analysis plan was provided, however, all analysis that was stated in methods was completed and results given	**Serious**
Einarson et al (2003)	**Serious**	**Serious**	**Moderate**	**Low**	**Serious**	**Low**	**Moderate**	**Serious**

For peer review only

	Justification: Only indication for treatment and individual characteristics domain was adjusted for, meaning lifestyle exposures was not adjusted for resulting in serious risk of bias	Justification: Exposure was determined through self-report of the individual, however this was not verified through physician or healthcare records	Justification: Although start of intervention and follow-up were unlikely at the same time point, the study restricted to exposure during the first trimester only, therefore the differing effects of intervention across different time periods potentially inducing bias is minimised as restricted to first trimester	Justification: Study was not undergone in an experimental context	Justification: Although complete case analysis was utilised, the total sample of how many exposed individuals was not mentioned, only the number included in a complete case analysis. Therefore, there will likely be missing data, and as the outcome was determined through self-report there is no evidence that the missingness of the outcome was not related to its true value.	Justification: The outcome was determined through self-report questionnaire, however this was then confirmed through the individuals physician. Although the outcome assessors could have been aware of exposure status, this was unlikely to have influenced the reporting of the outcome	Justification: No statistical analysis plan was provided, however, all analysis that was stated in methods was completed and results given	
Sivojelezova et al (2005)	**Serious**<br>Justification: Although presence of depression and maternal age was adjusted for, there was no factor that was adjusted for in lifestyle exposures. Therefore, if not all three of the confounding domains are	**Moderate**<br>Justification: Self-report of exposure through a standardised intake form. In addition, letter sent to physician to confirm the information given by individual	**Serious**<br>Justification: The start of intervention and follow-up were likely not at the same time point as women just rung up enquiring about exposure, in addition, no analysis methods were used to account for the differing effects of intervention across different time points	**Low**<br>Justification: Study was not undergone in an experimental context	**Serious**<br>Justification: Although complete case analysis was used, the number of exposed individuals and total sample was not stated. Therefore, there could have been missing data and there was no evidence to show that the missing data in the	**Low**<br>Justification: The outcome was measured through a questionnaire which is not the most reliable, however, the questionnaire was also sent to healthcare professional to confirm. Therefore, the healthcare professional would likely know the exposure status of	**Moderate**<br>Justification: No statistical analysis plan was given	**Serious**

	adjusted for it means serious rating				outcome was not associated with the the true value of the outcome.			
Chun-Fai-Chan et al (2005)	**Moderate** Justification: All three confounding domains of indication for treatment, lifestyle exposures and individual characteristics were adjusted for	**Moderate** Justification: Exposure was classified through self-report in questionnaire, however this was verified through a physician. Additionally, there was a follow-up questionnaire. As exposure was measured prospectively there was no risk of outcome influencing reporting of exposure	**Serious** Justification: The start of follow-up and intervention are not at the same point, which could induce bias as the intervention has differing effects over time, and no analytical techniques were put in place to minimise this bias	**Low** Justification: Study was not undergone in an experimental context	**Serious** Justification: Although complete case analysis was used, the number of exposed individuals and total sample was not stated. Therefore, there could have been missing data and there was no evidence to show that the missing data was not associated with the true value of the outcome. This is particularly relevant considering the outcome was individual self report.	**Low** Justification: outcome was measured through telephone conversation and then. Verified with physician. Although the outcome assessors could have been aware of exposure status	**Moderate** Justification: No statistical analysis plan was provided, however, all analysis that was stated in methods was completed and results given	**Serious**
Djulus et al (2006)	**Moderate** Justification: All three confounding factors of indication for treatment, individual characteristics	**Moderate** Justification: Women contacted the service regarding exposure and then details of exposure were taken including duration, timing	**Serious** Justification: The start of follow-up and intervention are not at the same point, which could induce bias as the intervention has differing effects over	**Low** Justification: Study was not undergone in an experimental context	**Serious** Justification: Complete case analysis was used but there was no total sample size or flow diagram indicating recruitment of	**Low** Justification: The outcome was measured through questionnaire to individual, alongside the results being verified by a physician. Although	**Moderate** Justification: No statistical analysis plan was provided, however, all analysis that was stated in methods was completed and results given	**Serious**

For peer review only

	and lifestyle exposures were adjusted for	and frequency - this questionnaire was then sent to practitioner for details to be confirmed	time, and no analytical techniques were put in place to minimise this bias		individuals and any exclusions. Missing data was not mentioned at all.	the outcome assessors could have been aware of exposure status, this is unlikely to have influenced outcome classification.		
Einarson et al (2009)	**Serious** Justification: Although the domains of individual characteristics and lifestyle exposures were adjusted for through maternal age and smoking, indication for treatment domain was not adjusted for	**Serious** Justification: Exposure was determined through self-report of the individual, however this was not verified through physician or healthcare records	**Moderate** Justification: Although start of intervention and follow-up were unlikely at the same time point, the study restricted to exposure during the first trimester only, therefore the differing effects of intervention across different time periods potentially inducing bias is minimised as restricted to first trimester	**Low** Justification: Study was not undergone in an experimental context	**Serious** Justification: Although complete case analysis was utilised, the total sample of how many exposed individuals was not mentioned, only the number included in a complete case analysis. Therefore, there will likely be missing data, and as the outcome was determined through self-report there is no evidence that the missingness of the outcome was not related to its true value.	**Serious** Justification: The outcome was reported by the mother/individual, however this was not verified with their physician or healthcare records. Therefore the exposure status known by the individual could influence their reporting of outcome classification.	**Moderate** Justification: No statistical analysis plan was provided, however, all analysis that was stated in methods was completed and results given	**Serious**
Nakhai-Pour et al (2010)	**Serious** Justification: It did not adjust for a factor in all three of the key confounding domains, as it did	**Moderate** Justification: The exposure classification was done through GP records and therefore all	**Moderate** Justification: Matching was employed, as well as controls selected independently to intervention status.	**Low** Justification: Study was not undergone in an experimental context	**Low** Justification: Smoking and maternal weight covariates were the only variables which had missing	**Low** Justification: The outcome was measured through the Quebec pregnancy register and therefore very	**Serious** Justification: No statistical analysis plan was given, additionally lots of data collected on covariates however	**Serious**

For peer review only

	not adjust for any factor of lifestyle exposure	information to classify intervention was recorded at start of intervention. Extent of non-differential misclassification would not be large.	The cases and controls were matched on gestational age so same risk period. Therefore, not at serious risk of bias in this domain		data. The missingness of these confounding factors is unlikely to be related to the true value of the outcome because the outcome was determined through pregnancy register rather than self-report	objective and not influenced by assessors	only some included in the adjustment model. Finally no results given for the sensitivity analysis.	
Paulus et al (2010)	**Critical** No information given on any confounding adjustments done	**Critical**	**Critical**	**Critical**	**Critical**	**Critical**	**Critical**	**Critical**
Chan et al (2011)	**Serious** Justification: Only individual characteristics and lifestyle exposure domains had a factor adjusted for in them - however indication for treatment was not adjusted for at all - therefore deemed serious as not all confounding domains satisfied.	**Serious** Justification: It was unclear the process of exposure classification, and indicated that only telephone interviews of self-report were used for exposure classification.	**Moderate** Justification: Start of intervention and follow-up were not the same, however, the analysis used was logistic time survival models so the differing effect of ads of different time periods was accounted for	**Low** Study was not undergone in an experimental context	**Serious** Justification: No information given on missing data.	**Low** Justification: Telephone interviews and medical records were used to determine the outcome, reliable method. Unclear whether outcome assessors were aware of exposure status, however unlikely to have influenced outcome determination because outcome is very objective	**Moderate** Justification: No statistical analysis plan was provided, however, all analysis that was stated in methods was completed and results given	**Serious**

Einarson et al (2011)	**Moderate**<br>Justification: All three confounding domains were adjusted for	**Serious**<br>Justification: Exposure was determined through self-report of the individual, however this was not verified through physician or healthcare records	**Serious**<br>Justification: The start of follow-up and intervention are not at the same point as women ringing the service enquiring about an exposure could have been taking it for weeks prior to call, which could induce bias as the intervention has differing effects over time, and no analytical techniques were put in place to minimise this bias	**Low**<br>Study was not undergone in an experimental context	**Serious**<br>Justification: Although complete case analysis was used, the number of exposed individuals/total sample was not stated. Therefore, there could have been missing data on participants that would have been excluded - this missing data could include outcome data which could be related to its true value as the outcome was self-report.	**Low**<br>Justification: The outcome was determined through self-report questionnaire; however this was then confirmed through the individuals physician. Although the outcome assessors could have been aware of exposure status	**Moderate**<br>Justification: No statistical analysis plan was provided, however, all analysis that was stated in methods was completed and results given	**Serious**
Ban et al (2012)	**Moderate**<br>Justification: All three confounding domains were adjusted for through some factor - including presence of depression, maternal age and smoking	**Moderate**<br>Justification: Exposure was classified through prescription databases where all drug usage was recorded - however just small difficulty of unsure the true ingested drug usage	**Serious**<br>Justification: Although prescription database was used, it is unlikely that the start of intervention and start of follow-up will be the same. Additionally, the authors did not use any statistical analysis including cox proportional regression to counteract	**Low**<br>Study was not undergone in an experimental context	**Low**<br>Justification: Missing data on covariates found, and the missingness would be unrelated to the outcome because they were taken from NHS databases before the outcome occurred. Additionally, no missing outcome data was found	**Low**<br>Justification: Outcome was measured reliably through healthcare records, however unclear whether outcome assessors would know exposure status but unlikely this would have influenced classification	**Moderate**<br>Justification: Although there was no statistical analysis plan there were no missing parts to the analysis.	**Serious**

For peer review only - http://bmjopen.bmj.com/site/about/guidelines.xhtml

Klieger-Grossman et al (2012)	**Moderate** Justification: All three confounding domains were adjusted for through some factor - including presence of depression, maternal age and smoking	**Moderate** Justification: Initial interview questioning exposure, then a follow-up interview to confirm answers given in original questioning. This was all done prospectively so no risk of outcome influencing exposure reporting	**Serious** Justification: The start of intervention and follow-up would not have been the same, therefore inducing potential bias, additionally no analytical techniques were put in place to account for this bias either through time dependent analysis or restricting the analysis to first trimester only	**Low** Study was not undergone in an experimental context	**Serious** Justification: Although complete case analysis was used, the number of exposed individuals and total sample was not stated. Therefore, there could have been missing data and there was no evidence to show that the missing data of outcome was not associated with the true value of it	**Low** Justification: Assessment of the outcome won't be influenced by the exposure	**Moderate** Justification: No statistical analysis plan was provided, however, all analysis that was stated in methods was completed and results given	**Serious**
Kjaesgaard et al (2013)	**Serious** Justification: only two of the three confounding domains were adjusted for - as the third domain of lifestyle exposures was not adjusted for	**Moderate** Justification: Prescription records were used to determine exposure, therefore likely to classify individuals correctly, however if there is misclassification this will likely be non-differential and attenuate towards the null	**Serious** Justification: Unlikely the start of follow-up and intervention were at the same time point. Additionally, no analysis was utilized to account foe the differing effect of intervention at different points during pregnancy	**Low** Study was not undergone in an experimental context	**Low** Justification: There was missing gestational age data, however this was replaced by the median of all non-missing values. Therefore covariate data is missing - as the outcome was determined through hospital registry it is unlikely that the true value of the outcome will influence the covariate	**Low** Justification: Outcome was assessed through codes on danish national hospital registry therefore very reliable method and outcome assessors unlikely aware of exposure status	**Moderate** Justification: No statistical analysis plan was provided, however, all analysis that was stated in methods was completed and results given	**Serious**

For peer review only

Andersen et al (2014)	**Serious** Justification: No factor of lifestyle exposure was adjusted for in this analysis, only indication for treatment and individual characteristics was adjusted for	**Moderate** Justification: exposure was classified through prescription databases therefore unlikely to be any issues with classification of exposure - however slight issues with true ingested drug usage.	**Moderate** Justification: Prescription database was used meaning unclear whether start of intervention and start of follow-up were at the same time point. However, a cox proportional hazards regression was used which counteracts the differing effects that exposure will have at different time points during pregnancy.	**Low** Study was not undergone in an experimental context	**Low** Justification: Minimal missing data was found on the confounding factors used, additionally, outcome was measured through hospital records therefore unlikely that the true value of the outcome will be influencing the missingness. No missing outcome data.	**Moderate** Justification: Outcome was measured in a very objective and reliable method through hospital records, unclear whether outcome assessors knew exposure status however this is unlikely to influence exposure status determination. However, the method of outcome assessment could have differed between interventions as individuals on antidepressants would be more likely to come into hospital as more contact with healthcare services compared to unexposed individuals.	**Moderate** Justification: No statistical plan was given, however all results that were mentioned in method were given	**Serious**
Abadie et al (2015)	**Serious** Justification: Only one of the three confounding domains had anything adjusted for - only maternal age had been accounted for -	**Moderate** Justification: The CRPV was notified by healthcare professional regarding exposure, then a questionnaire was sent across and confirmation of	**Serious** Justification: The authors did employ matching, however the controls were not independently selected of the intervention status. Additionally, they didn't match on	**Low** Study was not undergone in an experimental context	**Low** Justification: There was missing data on the confounding factors, as the outcome was measured through a database it is unlikely the missingness of	**Low** Justification: The outcome was measured through database administrative records, therefore very reliable	**Moderate** Justification: Although there was no statistical analysis plan there were no missing parts to the analysis.	**Serious**

For peer review only

	therefore not all three confounding domains satisfied and serious risk of bias	current medication taken currently, so low risk of differential misclassification.	gestational age so time at risk would have differed between cases and controls		covariates is related to true value of outcome. However, no missing data for the outcome was found.			
Johansen et al (2015)	**Serious** Justification: Although this paper did a large amount of adjustments, especially in relation to confounding by indication, which most other papers didn't, it still didn't adjust for all three confounding domains as it missed adjusting for factors on lifestyle exposure	**Moderate** Justification: Both prescription records and self-report was used to assess exposure classification for individuals, therefore very reliable method using both - it did find differences between the two methods so likely there will be misclassification however very minimal	**Moderate** Justification: It is unlikely the start of intervention and start of follow-up were at the same point, however a cox proportional hazards regression was used to account for the differing effects of exposure at different time points during pregnancy	**Low** Study was not undergone in an experimental context	**Low** Justification: A flow diagram illustrating the total sample and any excluded individuals or missing data. This highlighted there was missing data in the exposure, covariates and outcome. However, as the outcome was assessed through healthcare registers and bypassing the mother its unlikely the true value of the outcome would cause this missingness.	**Low** Justification: The outcome was assessed through healthcare registers and the outcome assessors would be unaware of exposure status therefore very little risk regarding exposure influencing outcome status, especially considering the objective nature of the outcome	**Serious** Justification: No statistical analysis plan was given, and information on confounders was taken which were not used in the adjustment	**Serious**
Te winkel et al (2016)	**Critical** No information given on any confounding adjustments done	**Critical**	**Critical**	**Critical**	**Critical**	**Critical**	**Critical**	**Critical**
Almeida et al (2016)	**Serious** Justification: Only indication for	**Moderate** Justification: exposure was	**Serious** Justification: Although	**Low** Study wasn't undergone in an	**Low** Justification: There was missing data	**Low** Justification: Outcome was	**Moderate** Justification: No statistical plan was	**Serious**

	treatment and individual characteristics domain had been adjusted for, whereas no factors were adjusted for in lifestyle exposures	classified through prescription database, therefore highly reliable. However, there may be some discrepancies as to the actual ingested drug usage.	prescription database was used, it is unlikely that the start of intervention and start of follow-up will be the same. Additionally, the authors did not use any statistical analysis including cox proportional regression to counteract the differing effects that exposure will have at different time points during pregnancy.	experimental context	on the confounding factors, as the outcome was measured through the physician rather than self-report it is unlikely the true value of the outcome would be influencing the missingness of these covariates . However, no missing data for the outcome was found.	assessed through ICD-9 codes of physician notes, therefore very objective. Unclear whether outcome assessors know exposure status, however this is unlikely to impact the outcome classification	given, however all results that were mentioned in method were given	
Evans-Hoeker et al (2018)	**Moderate** Justification: All three confounding domains were adjusted for	**Moderate** Justification: The medication exposure was assessed throughout a previous RCT, with regulators of the trial ensuring accurate medication exposure classification	**Serious** Justification: It is unlikely the start of intervention and follow-up were at the same time point, additionally no analysis was utilised to account for the differing effect of intervention at differing points during pregnancy	**Low** Study wasn't undergone in an experimental context	**Moderate** Justification: 100% of individuals were included from the trial into the study. Less than 10% of PHQ scores were missing, therefore missing covariate data, no evidence that this missing data was not driven by the true value of the outcome.	**Low** Justification: The outcome was assessed throughout the RCT through testing and so a very thorough and reliable method. Unlikely that outcome assessors knowing the exposure would influence their classification of it	**Moderate** Justification: No statistical analysis plan was provided, however, all analysis that was stated in methods was completed and results given	**Serious**
Richardson et al (2019)	**Moderate** Justification: All three confounding domains had a factor that had been adjusted for	**Moderate** Justification: Exposure classification measured prospectively and by a healthcare	**Moderate** Justification: The start of intervention and follow-up were likely not at the same time point as individual rung up	**Low** Study wasn't undergone in an experimental context	**Low** Justification: They don't have complete data as missing data was reported in only the confounding	**Low** Justification: The outcome is a very objective measure. The outcome was measured through clinician self-report,	**Moderate** Justification: Although there was no statistical analysis plan there were no missing parts to the analysis.	**Moderate**

For peer review only

	including presence of maternal depression, maternal age and alcohol	professional over the phone. Classification of intervention status would not be influenced by the outcome	not necessarily at start of exposure. However, analysis used was time dependent cox models therefore the differing effect of intervention across different time periods was accounted for.		variables, however the incomplete data for these covariates is unlikely to have been influenced by the true value of the outcome because this was determined through clinician rather than self report.	and although exposure would have been known to them, this is unlikely to have influenced the outcome reporting.		
Wu et al (2019)	**Serious** Justification: Only individual characteristics and lifestyle exposure domains had a factor adjusted for in them - however indication for treatment was not adjusted for at all - therefore deemed serious as not all confounding domains satisfied.	**Serious** Justification: The exposure was classified at the end of first trimester interview - so a time point after the start of follow up. Additionally in a lot of the cases the exposure was recorded after the SA had already happened. Potentially differential misclassification between exposure and outcome individuals	**Moderate** Start of intervention and follow-up did not coincide, however a cox proportional hazard survival model was utilised to counteract the differing effects that exposure will have at different time points during pregnancy	**Low** Study wasn't undergone in an experimental context	**Serious** Justification: A flow diagram and tables with the details of missing data was illustrated. Individuals were excluded if they had missing data from the interview, this results in missing outcome data that could have been a result of the true value of the outcome.	**Low** Justification: The outcome was measured through a questionnaire, this was then confirmed through medical records. Although outcome assessors could have been aware of the exposure status it is unlikely assessment of outcome would have been influenced by this.	**Moderate** Justification: No statistical analysis plan was given	**Serious**
Bahat et al (2020)	**Serious** Justification: Unclear what they adjusted for	**No information**	**Moderate** Justification: It was unclear whether the start of intervention	**Low** Study wasn't undergone in an	**No information**	**No information**	**Serious** Justification: No statistical analysis plan was given,	**Serious**

For peer review only

	in this analysis and was not stated, however an adjusted estimate was given so some level of confounding was accounted for		and follow-up were at the same point, however, the analysis was restricted to first trimester where there is thought to be similar effects of Ads across first trimester so accounting for this	experimental context			additionally not all of the analysis that said was going to be conducted was released as results	
Kolding et al (2021)	**Critical** No confounding adjustment done	**Critical**	**Critical**	**Critical**	**Critical**	**Critical**	**Critical**	**Critical**
Ankarfeldt et al (2021)	**Moderate** Justification: All three confounding domains of indication for treatment, lifestyle exposures and individual characteristics had one factor adjusted for	**Moderate** Justification: Exposure was classified through prescription databases therefore likely to be reliable, however may not reflect true ingested drug usage.	**Moderate** Justification: Start of intervention and follow-up were likely not at same time point. However, a cox proportional hazard survival model was utilised to counteract the differing effects that exposure will have at different time points during pregnancy	**Low** Justification: Study wasn't undergone in an experimental context	**Low:** Justification: There was a small number of missing values for two covariates, however as this was not influenced by the value of the outcome and the missing values were imputed it poses minimal risk of bias.	**Low** Justification: The outcome was measured through patient registers, therefore very objective and reliable.	**Moderate** Justification: No statistical analysis plan was given but all of the analysis planned and stated in methods was conducted and given.	**Moderate**
Kitchin et al. (2022)	**Moderate** Justification: All three confounding domains of indication for treatment, lifestyle exposures and	**Moderate** Justification: Exposure was classified through prescription databases therefore likely to be reliable, however may not	**Serious** Justification: Although prescription database was used, it is unlikely that the start of intervention and start of follow-up will be the same.	**Low** Study wasn't undergone in an experimental context	**Low:** Justification: The only missing values were the LMP values which were imputed through subtracting 74 days off recorded entry date, therefore	**Low** Justification: Outcome was determined through ICD codes in BIFAP database, therefore very objective and reliable.	**Moderate** Justification: No statistical analysis plan was given but all of the analysis planned and stated in methods was conducted and given.	**Serious**

For peer review only

	individual characteristics had one factor adjusted for	reflect true ingested drug usage.	Additionally, the authors did not use any statistical analysis including cox proportional regression to counteract		poses minimal risk of bias.			
Ostenfeld et al. (2022)	**Serious** Justification: Only individual characteristics and lifestyle exposure domains had a factor adjusted for in them - however indication for treatment was not adjusted for at all - therefore deemed serious as not all confounding domains satisfied	**Moderate** Justification: Exposure was classified through prescription databases therefore likely to be reliable, however may not reflect true ingested drug usage.	**Moderate** Justification: It was unclear whether the start of intervention and follow-up were at the same point, however, the analysis did take into account various timepoints within exposure periods.	**Low** Study wasn't undergone in an experimental context	**Moderate** Justification: Not all individuals in the cohort were included in the analysis due to missing/implausible gestation values; these missing values could be associated with the outcome. Additionally, there were other missing values that were imputed using the mode value.	**Low** Justification: Outcome identified in national hospital register, therefore very objective and reliable	**Moderate** Justification: No statistical analysis plan was given but all of the analysis planned and stated in methods was conducted and given.	**Serious**
Giner-Soriano et al. (2022)	**Serious** Justification: Only individual characteristics and lifestyle exposure domains had a factor adjusted for in them - however indication for treatment was not adjusted for at all - therefore	**Moderate** Justification: Exposure was classified through prescription databases therefore likely to be reliable, however may not reflect true ingested drug usage.	**Serious** Justification: Although prescription database was used, it is unlikely that the start of intervention and start of follow-up will be the same. Additionally, the authors did not use any statistical analysis including cox proportional	**Low** Study wasn't undergone in an experimental context	**No information**	**Low** Justification: Outcome identified in primary health care records of Catalan institute, therefore very objective and reliable	**Moderate** Justification: No statistical analysis plan was given but all of the analysis planned and stated in methods was conducted and given.	**Serious**

For peer review only

	deemed serious as not all confounding domains satisfied		regression to counteract					

For peer review only

S5 Table. Confounding adjustments completed

Study	Confounding domain												
	Indication for treatment		Individual characteristics								Lifestyle exposures		
	Presence of depression	Severity of depression	History of SA	Maternal age	Gestational age	Polypharmacy	SEP	Ethnicity	BMI	Comorbidities	Smoking	Alcohol	Recreational drugs
Chambers et al (1996)													
Kulin et al (1998)													
Einarson et al (2001)	■												
Einarson et al (2003)	■				■								
Sivojelezova et al (2005)	■			■	■								
Chun-Fai-Chan et al (2005)	■			■	■						■	■	
Djulus et al (2006)	■			■						■	■	■	
Einarson et al (2009)			■	■	■						■	■	
Nakhai-Pour et al (2010)	■	■	■	■	■		■			■			
Paulus et al (2010)													
Chan et al (2011)				■			■	■	■		■	■	
Einarson et al (2011)	■			■	■						■	■	
Ban et al (2012)	■			■			■		■		■		
Klieger-Grossman et al (2012)	■			■	■						■	■	
Kjaesgaard et al (2013)	■			■			■			■			
Andersen et al (2014)	■		■	■		■	■						
Abadie et al (2015)				■									
Johansen et al (2015)	■	■	■	■	■		■						
Tw Winkel et al (2016)													
Almeida et al (2016)	■			■		■	■			■			
Evans-Hoeker et al (2018)	■	■		■			■	■			■		
Richardson et al (2019)	■		■	■	■						■	■	■
Wu et al (2019)			■	■								■	

For peer review only

**Bahat et al (2020)**													
**Kolding et al (2021)**													
**Ankarfeldt et al (2021)**													
**Kitchin et al. (2022)**													
**Ostenfeld et al. (2022)**													
**Giner-Soriano et al. (2022)**													

Green highlighted box indicating factor adjusted for and blank box indicating factor was not adjusted for

For peer review only

S6 Table. Meta-analysis results

Analysis type		Number of studies	Number of adjusted studies used	Summary effect estimate (95% confidence interval)	Heterogeneity
**Main analysis**					
**Studies in the general population**	AD use vs unexposed comparator	29	18	1.24 (1.18-1.31)	69.2%
	SSRI vs unexposed comparator	12	6	1.29 (1.15-1.44)	81.3%
	SNRI vs unexposed comparator	5	2	1.42 (1.11 – 1.81)	46.9%
**Studies restricting population to women with depression**	AD use vs unmedicated depressed comparator	6	4	1.16 (1.04 – 1.31)	58.6%
	SSRI v another AD	5	2	1.03 (0.93-1.15)	0%
	SNRI vs another AD	3	2	1.19 (1.02 – 1.39)	0%
**Sensitivity analysis restricting to studies of sufficient quality**					
**Studies in the general population**	AD use vs unexposed comparator	24	18	1.24 (1.17-1.31)	72.10%
	SSRI vs unexposed comparator	9	6	1.30 (1.15-1.46)	86.0%
	SNRI vs unexposed comparator	3	2	1.17 (1.01-1.36)	0%

For peer review only - http://bmjopen.bmj.com/site/about/guidelines.xhtml

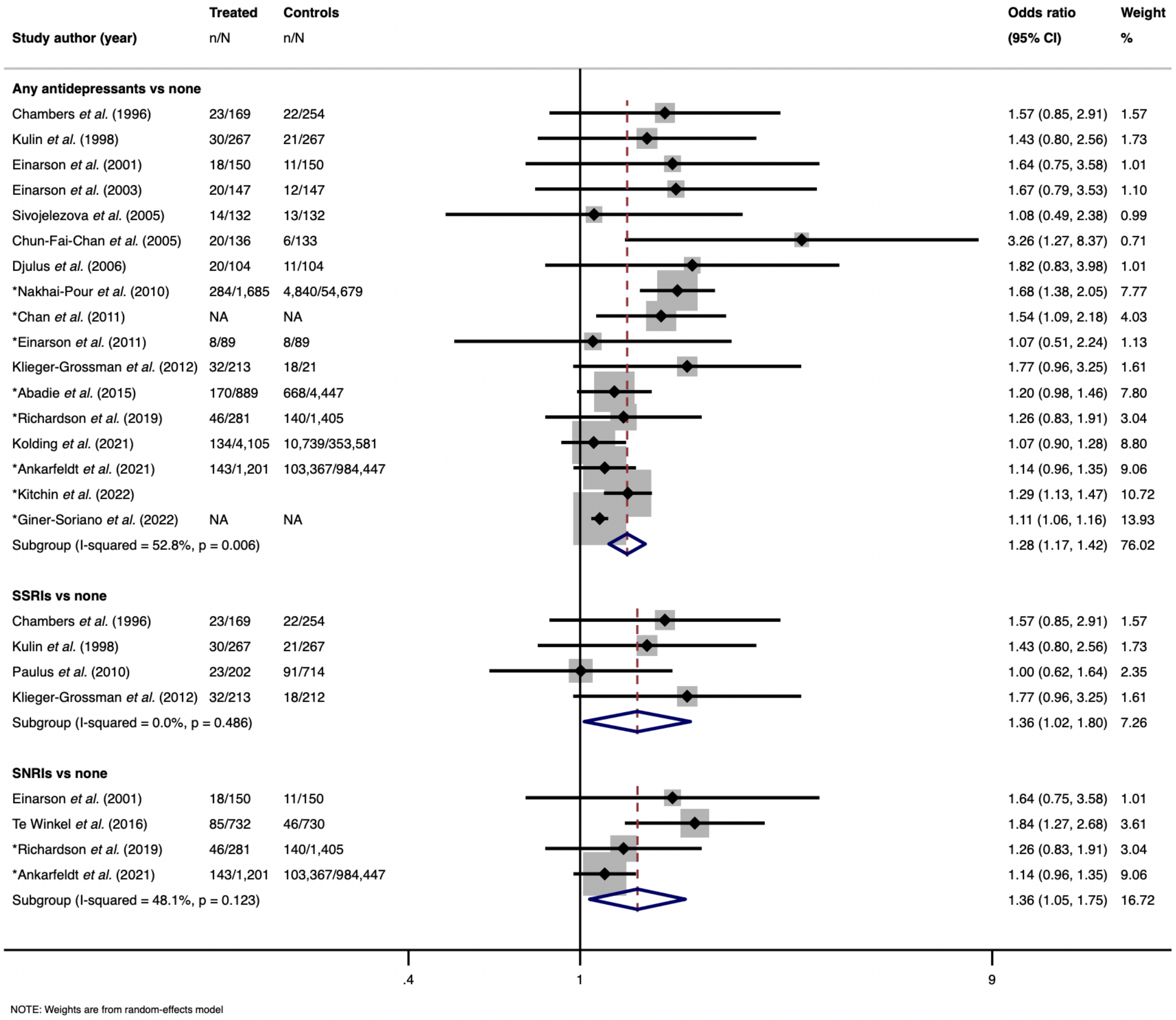

S1 Figure. Sensitivity analysis utilising only papers reporting odds ratios.

General population sensitivity analysis including only papers which included odds ratio, and excluding papers reporting hazards and risk ratio

*These studies had an adjusted estimate from multivariable model which was pooled in the meta-analysis rather than raw data, therefore the treated and controls (n/N) data does not correspond to the odds ratio shown.

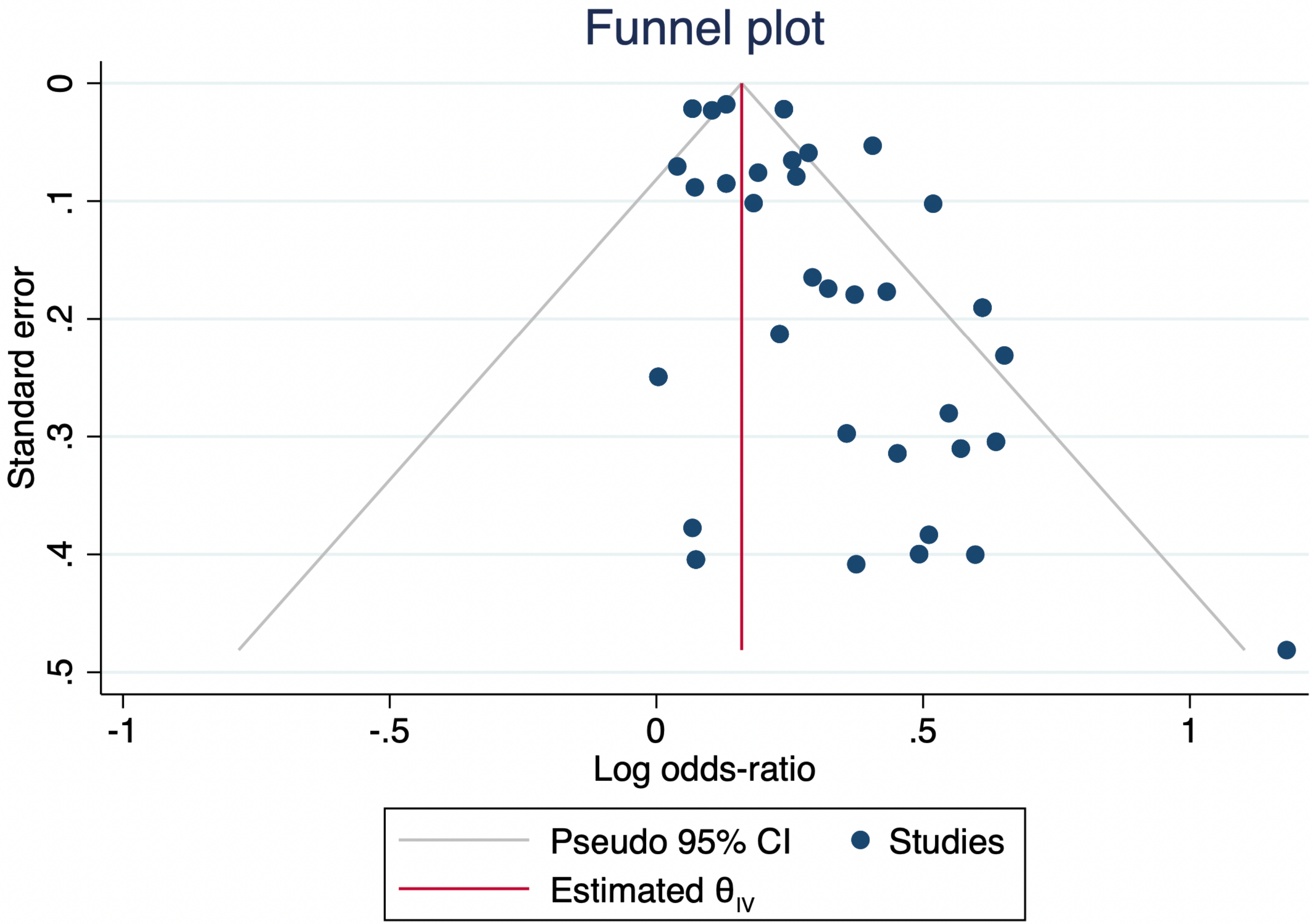

S2 Figure. Funnel plot

For peer review only

PRISMA 2020 Checklist

Section and Topic	Item #	Checklist item	Location where item is reported
**TITLE**			
Title	1	Identify the report as a systematic review.	Pg1
**ABSTRACT**			
Abstract	2	See the PRISMA 2020 for Abstracts checklist.	Pg2
**INTRODUCTION**			
Rationale	3	Describe the rationale for the review in the context of existing knowledge.	Pg5
Objectives	4	Provide an explicit statement of the objective(s) or question(s) the review addresses.	Pg5
**METHODS**			
Eligibility criteria	5	Specify the inclusion and exclusion criteria for the review and how studies were grouped for the syntheses.	Pg6
Information sources	6	Specify all databases, registers, websites, organisations, reference lists and other sources searched or consulted to identify studies. Specify the date when each source was last searched or consulted.	Pg6
Search strategy	7	Present the full search strategies for all databases, registers and websites, including any filters and limits used.	Appendix S1
Selection process	8	Specify the methods used to decide whether a study met the inclusion criteria of the review, including how many reviewers screened each record and each report retrieved, whether they worked independently, and if applicable, details of automation tools used in the process.	Pg6 & 7
Data collection process	9	Specify the methods used to collect data from reports, including how many reviewers collected data from each report, whether they worked independently, any processes for obtaining or confirming data from study investigators, and if applicable, details of automation tools used in the process.	Pg7
Data items	10a	List and define all outcomes for which data were sought. Specify whether all results that were compatible with each outcome domain in each study were sought (e.g. for all measures, time points, analyses), and if not, the methods used to decide which results to collect.	Pg7
	10b	List and define all other variables for which data were sought (e.g. participant and intervention characteristics, funding sources). Describe any assumptions made about any missing or unclear information.	Pg7
Study risk of bias assessment	11	Specify the methods used to assess risk of bias in the included studies, including details of the tool(s) used, how many reviewers assessed each study and whether they worked independently, and if applicable, details of automation tools used in the process.	Pg7
Effect measures	12	Specify for each outcome the effect measure(s) (e.g. risk ratio, mean difference) used in the synthesis or presentation of results.	Pg7
Synthesis methods	13a	Describe the processes used to decide which studies were eligible for each synthesis (e.g. tabulating the study intervention characteristics and comparing against the planned groups for each synthesis (item #5)).	Pg7
	13b	Describe any methods required to prepare the data for presentation or synthesis, such as handling of missing summary statistics, or data conversions.	Pg7
	13c	Describe any methods used to tabulate or visually display results of individual studies and syntheses.	Pg7
	13d	Describe any methods used to synthesize results and provide a rationale for the choice(s). If meta-analysis was performed, describe the model(s), method(s) to identify the presence and extent of statistical heterogeneity, and software package(s) used.	Pg7 & pg8
	13e	Describe any methods used to explore possible causes of heterogeneity among study results (e.g. subgroup analysis, meta-regression).	Pg 7 & 8
	13f	Describe any sensitivity analyses conducted to assess robustness of the synthesized results.	Pg 8
Reporting bias assessment	14	Describe any methods used to assess risk of bias due to missing results in a synthesis (arising from reporting biases).	Pg 7

For peer review only - http://bmjopen.bmj.com/site/about/guidelines.xhtml

PRISMA 2020 Checklist

Section and Topic	Item #	Checklist item	Location where item is reported
Certainty assessment	15	Describe any methods used to assess certainty (or confidence) in the body of evidence for an outcome.	Pg pg 8
**RESULTS**			
Study selection	16a	Describe the results of the search and selection process, from the number of records identified in the search to the number of studies included in the review, ideally using a flow diagram.	Figure s1
	16b	Cite studies that might appear to meet the inclusion criteria, but which were excluded, and explain why they were excluded.	Table s1
Study characteristics	17	Cite each included study and present its characteristics.	Appendix s2
Risk of bias in studies	18	Present assessments of risk of bias for each included study.	Appendix s4
Results of individual studies	19	For all outcomes, present, for each study: (a) summary statistics for each group (where appropriate) and (b) an effect estimate and its precision (e.g. confidence/credible interval), ideally using structured tables or plots.	Figure s1, s2, s3 and appendix s3
Results of syntheses	20a	For each synthesis, briefly summarise the characteristics and risk of bias among contributing studies.	Appendix s4<br>Pg10
	20b	Present results of all statistical syntheses conducted. If meta-analysis was done, present for each the summary estimate and its precision (e.g. confidence/credible interval) and measures of statistical heterogeneity. If comparing groups, describe the direction of the effect.	Appendix s3<br>Pg9
	20c	Present results of all investigations of possible causes of heterogeneity among study results.	Appendix s3<br>Pg 14
	20d	Present results of all sensitivity analyses conducted to assess the robustness of the synthesized results.	Appendix s3
Reporting biases	21	Present assessments of risk of bias due to missing results (arising from reporting biases) for each synthesis assessed.	NA
Certainty of evidence	22	Present assessments of certainty (or confidence) in the body of evidence for each outcome assessed.	Appendix s3<br>Pg 9
**DISCUSSION**			
Discussion	23a	Provide a general interpretation of the results in the context of other evidence.	Pg 11 & 12
	23b	Discuss any limitations of the evidence included in the review.	Pg 12 & 13
	23c	Discuss any limitations of the review processes used.	Pg 14
	23d	Discuss implications of the results for practice, policy, and future research.	Pg 15
**OTHER INFORMATION**			
Registration and protocol	24a	Provide registration information for the review, including register name and registration number, or state that the review was not registered.	Pg 8
	24b	Indicate where the review protocol can be accessed, or state that a protocol was not prepared.	Pg 8

For peer review only

PRISMA 2020 Checklist

Section and Topic	Item #	Checklist item	Location where item is reported
	24c	Describe and explain any amendments to information provided at registration or in the protocol.	NA
Support	25	Describe sources of financial or non-financial support for the review, and the role of the funders or sponsors in the review.	Pg 4
Competing interests	26	Declare any competing interests of review authors.	Pg 16
Availability of data, code and other materials	27	Report which of the following are publicly available and where they can be found: template data collection forms; data extracted from included studies; data used for all analyses; analytic code; any other materials used in the review.	Throughout

From: Page MJ, McKenzie JE, Bossuyt PM, Boutron I, Hoffmann TC, Mulrow CD, et al. The PRISMA 2020 statement: an updated guideline for reporting systematic reviews. BMJ 2021;372:n71. doi: 10.1136/bmj.n71

For more information, visit: http://www.prisma-statement.org/

For peer review only

BMJ Open

The association between antidepressant use during pregnancy and miscarriage: A systematic review and meta-analysis

Journal:	*BMJ Open*
Manuscript ID	bmjopen-2023-074600.R2
Article Type:	Original research
Date Submitted by the Author:	01-Nov-2023
Complete List of Authors:	Smith, Sophie; University of Bristol Medical School,<br>Martin, Flo; University of Bristol Medical School<br>Rai, Dheeraj; University of Bristol, Centre for Academic Mental Health, School of Social & Community Medicine<br>Forbes, Harriet; University of Bristol,
**Primary Subject Heading**:	Mental health
Secondary Subject Heading:	Epidemiology, Mental health, Obstetrics and gynaecology
Keywords:	Depression & mood disorders < PSYCHIATRY, EPIDEMIOLOGY, Maternal medicine < OBSTETRICS

SCHOLARONE™ Manuscripts

BMJ

I, the Submitting Author has the right to grant and does grant on behalf of all authors of the Work (as defined in the below author licence), an exclusive licence and/or a non-exclusive licence for contributions from authors who are: i) UK Crown employees; ii) where BMJ has agreed a CC-BY licence shall apply, and/or iii) in accordance with the terms applicable for US Federal Government officers or employees acting as part of their official duties; on a worldwide, perpetual, irrevocable, royalty-free basis to BMJ Publishing Group Ltd ("BMJ") its licensees and where the relevant Journal is co-owned by BMJ to the co-owners of the Journal, to publish the Work in this journal and any other BMJ products and to exploit all rights, as set out in our licence.

The Submitting Author accepts and understands that any supply made under these terms is made by BMJ to the Submitting Author unless you are acting as an employee on behalf of your employer or a postgraduate student of an affiliated institution which is paying any applicable article publishing charge ("APC") for Open Access articles. Where the Submitting Author wishes to make the Work available on an Open Access basis (and intends to pay the relevant APC), the terms of reuse of such Open Access shall be governed by a Creative Commons licence – details of these licences and which Creative Commons licence will apply to this Work are set out in our licence referred to above.

Other than as permitted in any relevant BMJ Author's Self Archiving Policies, I confirm this Work has not been accepted for publication elsewhere, is not being considered for publication elsewhere and does not duplicate material already published. I confirm all authors consent to publication of this Work and authorise the granting of this licence.

For peer review only

The association between antidepressant use during pregnancy and miscarriage: A systematic review and meta-analysis

Centre for Academic Mental Health, University of Bristol, Bristol (Sophie Smith MSc)
Centre for Academic Mental Health, University of Bristol, Bristol (Florence Martin MSc)
Centre for Academic Mental Health, University of Bristol, Bristol (Dr Dheeraj Rai MRCPsychPHD)
Centre for Academic Mental Health, University of Bristol, Bristol (Dr Harriet Forbes PhD)

Correspondence to Sophie Smith, Centre for Academic Mental Health, University of Bristol, Bristol, BS8 2BN
Sophie.smith@bristol.ac.uk

Word count: 3865

For peer review only

Abstract

Background

Literature surrounding the association between antidepressant use during pregnancy and miscarriage is conflicting. We aimed to conduct a systematic review and meta-analysis of studies among pregnant women regarding the association between exposure to antidepressants during pregnancy and the risk of miscarriage, compared to pregnant women not exposed to antidepressants.

Design

We conducted a systematic review and meta-analysis of non-randomised studies.

Data sources

We searched MEDLINE, EMBASE and PsychINFO up to 6th August 2023.

Eligibility criteria and outcomes

Case-control, cohort and cross-sectional study designs were selected if they compared individuals exposed to any antidepressant class during pregnancy to comparator groups of either no antidepressant use or an alternate antidepressant.

Data extraction and synthesis

Effect estimates were extracted from selected studies and pooled using a random-effects meta-analysis. Risk of bias (RoB) was assessed using the ROBINS-I tool, and heterogeneity assessed using the I^2 statistic. Subgroup analyses were utilised to explore antidepressant classes and the impact of confounding by indication.

Results

1800 records were identified from the search, of which 29 were included in the systematic review and meta-analysis. The total sample included 5,671,135 individuals. Antidepressant users initially appeared to have a higher risk of miscarriage compared to unexposed individuals from the general population (summary effect estimate: 1.24, 95% CI: 1.18-1.31,

For peer review only

I^2 = 69.2%; number of studies (n)=29). However, the summary estimate decreased when comparing against unexposed individuals with maternal depression (1.16, 1.04-1.31; I^2 = 58.6%; n=6), suggesting confounding by indication may be driving the association. 22 studies suffered from serious RoB, and only two of the 29 studies were deemed at moderate RoB.

Conclusions

After accounting for maternal depression there is little evidence of any association between antidepressant use during pregnancy and miscarriage. Instead, the results indicate the biasing impact of confounding by indication.

Key words: Depression; antidepressant use; miscarriage; spontaneous abortion; confounding by indication

Strengths and limitations of the study:

- A large amount of studies were identified through an extremely thorough search strategy, thus enhancing statistical power and insight over previous reviews.
- Confounding by indication and depression severity was considered in the analysis and results, elucidating the true independent effect of antidepressant use during pregnancy.
- The ROBINS-I tool was used to evaluate the strength and quality of evidence.
- The studies included could suffer from publication bias, and small studies producing inverse relationships are absent from the field.
- High heterogeneity existed between papers, likely due to the included studies investigating different research questions, implementing different methodologies and populations, timing of exposure and confounding adjustment, thereby reducing the quality of the meta-analysis.

Introduction

Depression affects around 264 million people worldwide and is one of the top three leading causes of years lived with disability globally.(1) This high prevalence is also seen among pregnant women, with 15-23% suffering from a depressive disorder.(2) Around 3-8% of

For peer review only

women are prescribed antidepressants during their pregnancy in Europe.(3) This figure appears to be on an upwards trajectory since the 1990s,(4) potentially driven by both increasing prenatal depression rates,(5) and a shortage of non-pharmacological treatment options.(6)

Miscarriage, also referred to as spontaneous abortion, is defined as pregnancy loss before viability, however, the gestational threshold of viability varies between countries, from 20-28 weeks.(7) In the UK, viability is determined as up to 24 weeks.(7) Depending on the definition, miscarriage risk varies; on average it occurs in approximately 15% of pregnancies.(7) Several studies and reviews found an increased miscarriage risk in women using antidepressants during pregnancy, compared to unexposed individuals.(8-10) Antidepressants can cross the placental barrier through passive diffusion,(11) highlighting biological plausibility. Selective serotonin reuptake inhibitors (SSRIs) and serotonin-norepinephrine reuptake inhibitors (SNRIs) are the most frequently used pharmacotherapy option for depression,(12) both of which increase serotonin levels in the synaptic cleft.(3, 13) Serotonergic systems are involved in embryonic developmental processes including neural crest cell differentiation,(13) a process producing cells and tissue types.(14) This highlights the importance of serotonin in embryogenesis thus its potential influence on miscarriage. Alternatively, untreated maternal depression has been shown to be associated with miscarriage(15). This is an issue in observational studies known as confounding by indication, whereby maternal depression can independently affect the outcome of miscarriage. This could be through a number of mechanisms and mediators including lifestyle factors,(16) or stress and inflammation.(15) Including pregnant women in randomised controlled trials (RCTs) is unethical, thus all evidence is observational and therefore susceptible to bias, making causal inference challenging. This is highlighted by studies whose observed associations between antidepressant use during pregnancy and miscarriage attenuated after stratifying for maternal depression.(17, 18)

The methodological limitations that exist in previous reviews means it remains unclear whether a causal relationship exists. Therefore, clear evidence and guidance around the association between antidepressant use during pregnancy and miscarriage is important as it will allow depressed pregnant women to make well-informed decisions and reduce their

For peer review only

decisional conflict.(19) Poor perinatal mental health is becoming an ever-growing issue, up-to-date studies are needed now which investigate child outcomes in depressed women to help drive screening, intervention and prevention strategies.

Existing reviews examining this relationship investigated several perinatal outcomes and produced differing conclusions.(20-22) These reviews have some methodological limitations including incomplete search strategies,(20) do not include more recent studies from the last 10 years,(21, 22) and did not fully address confounding by indication,(20-22) a crucial element in isolating the true effect of antidepressants. This issue was addressed in the current review through utilising a depressed comparator, to allow for increased comparability between exposed and unexposed individuals.

The main objective of this study is to conduct a systematic review and meta-analysis of studies among pregnant women regarding the association between exposure to antidepressants during pregnancy and the risk of miscarriage, compared to pregnant women not exposed to antidepressants.

Methods

Protocol registration

This study is conducted in accordance with the Preferred Reporting Items for Systematic Review and Meta Analysis (PRISMA) 2020, as well as the Cochrane handbook(23). The protocol for this meta-analysis was registered in PROSPERO CRD42021262839, available from
https://www.crd.york.ac.uk/prospero/display_record.php?ID=CRD42021262839

Search strategy and information sources

We searched the databases, EMBASE (Ovid, 1980 to 2023), MEDLINE (Ovid, 1946 to 2023) and PsycInfo (Ovid, 1806 to 2023) from database inception to 8th March 2021 using a comprehensive search strategy (Supplementary Table S1). In brief the following search terms were used: (antidepress* OR [generic/branded antidepressant name) AND (spontaneous abortion OR miscarriage). The search was updated on 4th August 2021 prior

to commencement of data analysis, a final search before finishing the review was conducted on 21st January 2022, as well as a search to update findings before publication on 6th August 2023. Backward citation searching was conducted from papers and existing reviews, additionally, conference abstracts found in the searches were included in the review. The outcome of stillbirth was also included in our search strategy as these two outcomes are frequently reported together. However, stillbirth was not included in this review due to a higher number of relevant studies found than expected, ensuring literature was investigated at a thorough level. We placed no restrictions on language or publication status. Abstracts in a language other than English were translated using a translation software, and full texts were assessed using a translator.

Eligibility criteria

Studies were included if the study population included pregnant women, the exposure was antidepressants, and the outcomes included miscarriage. Any antidepressant class was eligible, and no restriction was placed on timing, dose, or duration of use. The comparator groups included pregnant women prescribed a different antidepressant to the primary specified type, or women with no antidepressant exposure. Studies investigating polypharmacy involving medication other than antidepressants were excluded, however, studies investigating more than one antidepressant class were included. Observational studies included utilised a cohort, case-control, or cross-sectional design. RCTs were included if they fit the eligibility criteria. Reviews, meta-analyses, ecological studies, case reports and case series were excluded.

Study selection

The papers identified from the search strategy had their title and abstract screened independently by two reviewers in parallel (FM and HF). We used Endnote to de-duplicate references. Outputs of the review were then cross-checked, and discrepancies discussed with a third reviewer (SS). The full text of papers that passed title and abstract screening were obtained through Endnote, and three researchers completed a full-text review of a proportion of papers (SS, FM and HF). Final decisions and discrepancies were discussed within the whole team, including a consultant psychiatrist (DR).

For peer review only

Data extraction

A standardised template was used for data extraction. A PECOS framework was utilised and information on five domains of population, exposure, comparator, outcomes, and study characteristics was obtained. Variables of study design, country of study, study period, data sampling methods, type and timing of antidepressant exposure, definition of exposure and comparator groups, identification of exposure and outcome, sample size, confounding adjustment, raw data and adjusted estimates (odds ratio (OR), risk ratio (RR), hazard ratio (HR)) including confidence intervals (CIs) were extracted. If more than one comparator group was utilised, the raw data and adjusted estimates for each comparison were extracted. Data on any secondary outcomes were not extracted. Data extraction was conducted independently by two reviewers for all papers, outputs were cross-checked (SS and FM), and discrepancies were discussed with a third reviewer (HF).

Risk of bias assessment

Studies were assessed using the ROBINS-I tool.(24) This tool evaluates non-randomised studies estimating intervention effects. Seven domains were assessed, including issues arising prior to and after intervention. The ROBINS-I tool is designed for cohort studies, therefore a template alteration specific to case-control studies was sought from the author of the ROBINS-I tool to allow for continuity in risk of bias (RoB) assessment across all study designs. RoB was independently assessed by two reviewers in parallel for all studies (SS and FM). The scoring and justification were compared, with any discrepancies discussed with a third reviewer (HF).

Statistical analysis

Meta-analyses were performed using STATA (version 16.0). Results were presented by comparator group used, specifically general population unexposed, depressed unexposed and depressed alternative antidepressant exposure. Additionally, subgroup analysis on SSRIs and SNRIs were conducted. Adjusted effect estimates were the preferential estimate when pooling results. HR, RR and OR were treated as equivalent and pooled together, a sensitivity analysis on this assumption was conducted (Supplementary Figure S1). If studies gave estimates for multiple exposure groups including differing trimester estimates, these were pooled into one result using a meta-analysis. When adjusted estimates were not provided,

For peer review only

ORs and standard errors were produced from raw data. Analyses were conducted using a random-effects model, due to the high likelihood of heterogeneity expected. Heterogeneity between studies was assessed using the I^2 statistic.(25) Additionally, a funnel plot and Egger test were conducted to assess evidence for publication bias.(26) A sensitivity analysis was conducted whereby papers with an overall critical RoB, or no information as identified through ROBINS-I tool were excluded. Additionally, another sensitivity analysis was done whereby only papers which used prescription databases to classify exposure were included.

Patient and Public Involvement

No patient or public involvement.

Results

Search results

The initial and final search retrieved 2010 citations. After duplicates were removed, 1800 records had title and abstract screening, resulting in a further 1751 records being excluded. The full text of 49 articles were assessed for eligibility. Articles were excluded due to reasons including incorrect study design, insufficient data or if only stillbirth was investigated (Supplementary Table S2). This resulted in 25 studies being excluded, and 24 included, with the final searches adding a further five papers to the SR and meta-analysis;(9, 10, 17, 18, 27-51) highlighted in the PRISMA flow diagram (Figure 1).(52)

Study characteristics

Supplementary Table S3 details characteristics of the included studies. Only observational studies were found; 25 cohort, and four case-control studies, (10, 40, 50, 51) and all studies were published between 1996 and 2022. The total number of subjects investigated across case-control was 314,682, compared to 5,356,453 across cohort studies. The study size varied from 247 to 1,281,418. The miscarriage definition was rarely stated, however, ranged from loss of fetus at either 20 (n=3),(10, 42, 45) 22 (n=2)(17, 18) or 24 weeks' (n=1)(44) gestation. A mixture of comparator groups were utilised across studies. All studies had a comparator group of unexposed individuals without depression; some papers used additional comparator groups including depressed pregnant women exposed to an

For peer review only

alternative antidepressant to the primary specified type(29, 37, 38, 43, 44, 48), unexposed depressed pregnant women,(17, 18, 37, 42, 43, 50) and pregnant individuals using antidepressants whereby the indication for treatment wasn't depression.(17, 32, 43) Exposure status was predominantly identified through two methods. Firstly, 16 studies utilised self-report to teratogen information services through either the individual or their physician.(9, 27-34, 36, 38, 40, 41, 43-45) Secondly, fourteen studies used prescription databases.(10, 17, 18, 37, 39, 42, 47, 48) Individual antidepressant types were investigated, including 12 studies examining SSRIs(18, 27-29, 31, 34, 37-39, 43, 45, 50) and five SNRIs.(29, 41, 44, 46, 48)

Risk of bias assessment

The ROBINS-I tool found much of the literature to be at serious RoB. Supplementary Table S4 highlights that only two out of 29 papers had an overall moderate RoB,(44, 48) compared to 22 deemed serious, and the remaining deemed critical. This tool highlighted a key area of bias being confounding. The three crucial confounding domains identified for this field were indication for treatment, individual characteristics, and lifestyle exposures. The confounding variables believed to be most important included presence and severity of depression, as well as gestational age. Although a large proportion of studies stratified by depression (62%), only 14% and 31% of studies accounted for depression severity and gestational age, respectively (Supplementary Table S5). Confounding adjustments varied dramatically between studies suggesting a potential heterogeneity source. As very few confounding variables were included in these studies, it limits the reliability of the results of this systematic review.

Publication bias

Publication bias was assessed through visual inspection of the funnel plot (Supplementary Figure S2) and the Egger test. Asymmetry of the funnel plot indicated potential for publication bias, and the Egger test produced a p-value of 0.001.

Sensitivity analysis

Papers with an overall critical RoB or inadequate information to determine their bias category were removed (n=5). The papers removed were deemed critical as they had no

For peer review only

confounding adjustments. Results remained very similar, with a change in the pooled estimate of 0 and 0.01 in all antidepressants and SSRI analysis respectively in the general population after removing the critical papers. Additionally, in the SNRI general population analysis there was a change in the pooled estimate of 0.25 after removing the papers with no adjustments (Supplementary Table S6).

Additionally, we performed a meta-analysis utilising papers whereby exposure data was collected only through prescription databases. These results highlighted an attenuation towards the null compared to the primary meta-analysis utilising all exposure classification methods (supplementary table S6).

Meta-analysis results

Across all 29 studies using an unexposed general population comparator, antidepressant use was associated with miscarriage. Figure 2 illustrates the summary effect estimate (pooled odds ratio = 1.24 95%CI: 1.18-1.31, I^2=69.2%, p<0.001), which included 18 adjusted estimates. However, when stratifying for maternal depression and utilising a comparator of unexposed depressed pregnant women, the summary estimate decreased (pooled odds ratio= 1.16 95% CI: 1.04-1.31, I^2=58.6%, p=0.034) (Figure 3).

Additionally, studies were separated which investigated SSRIs or SNRIs and their possible varying risks. When comparing SSRI use and no antidepressant use, the pooled summary estimate for the outcome of miscarriage was 1.29 (95% CI: 1.15-1.44, I^2=81.3%, p<0.001). Alternatively, when comparing SNRI use with no antidepressant use, a higher summary estimate was observed (pooled odds ratio = 1.42 95% CI: 1.11-1.81, I^2= 49.6%, p=0.11) (Figure 2).

Subgroup analyses investigating SSRIs and SNRIs separately were performed again after restricting to a depressed cohort. When comparing SSRI use and individuals using other antidepressant types, it produced an attenuated summary estimate of 1.03 (95% CI: 0.93-1.15, I^2=0%, p=0.972). Whereas the summary estimate comparing SNRI against other antidepressant use on the outcome of miscarriage was slightly higher (pooled summary estimate = 1.19 95%CI: 1.02-1.39, I^2=0%, p=0.665) (Figure 3).

For peer review only

Discussion

Summary of evidence

This is the largest and most up-to-date review investigating the association between antidepressant use during pregnancy and miscarriage, with around twice as many studies included than previous reviews.(20-22) After pooling best estimates from 29 studies, we found little evidence to suggest an increased risk of miscarriage among antidepressant users after stratifying by maternal depression, with CIs almost spanning the null. The increased risk of miscarriage among pregnant women taking antidepressants compared to unexposed pregnant women in the general population is likely driven by confounding by indication.

Interpretation of findings

The general population analysis result was comparable to other smaller meta-analyses, which found effect estimates of 1.45, 1.49 and 1.47.(8, 20, 22) The increased estimates found by these papers are likely driven by fewer included studies, with 11, 14, and six respectively, compared to 29 in our study. The elevated effect estimates when utilising a general population control could likely be driven by confounding by indication, a bias which is lessened when comparing with depressed controls. To the best of our knowledge, meta-analyses in the field investigating miscarriage have not yet utilised an unexposed depressed comparator; thus, no comparable results exist. As only six studies were used in this subgroup meta-analysis, the summary estimate lacks power and robustness, inducing potential for chance findings, highlighted by large standard errors. Additionally, this weak miscarriage risk could be reflecting depression severity, whereby unexposed depressed individuals have a lower severity of depression than exposed individuals. Studies have indicated miscarriage risk between individuals continuing and discontinuing antidepressant use upon start of pregnancy is similar.(18, 48) Therefore, future studies should incorporate the comparator of discontinued antidepressant use to allow for more comparable depression severity. Furthermore, other confounding factors that weren't adequately controlled for could be biasing this estimate, thus, adjusting for all key confounders is essential in future studies to minimise residual confounding.

For peer review only

Our study found that SNRIs carry a greater risk of miscarriage than SSRIs when compared to an unexposed general population comparator group. No meta-analyses have been conducted on SNRIs to our knowledge, likely due to the higher prevalence of other antidepressant use during pregnancy, for example SSRIs.(12) Previous reviews utilising a fixed-effects method found odds ratios of 1.70 and 1.87 when comparing SSRIs to unexposed general population comparators.(53, 54) The lower effect estimate of 1.30 observed in our study may be partially driven by the use of a random-effects model to account for high heterogeneity, as well as the inclusion of an additional eight studies. The elevated point estimate for SNRIs compared with SSRIs may suggest that miscarriage risk differs between antidepressant classes. Alternatively, these estimates could be highlighting confounding by indication, which was further explored in a set of subgroup analyses that stratified by maternal depression. The summary effect estimates between both SSRIs and SNRIs and other antidepressant classes attenuated towards the null, thus, emphasising the potential impact of confounding by indication. The SSRI analyses had consistently lower estimates, including against other antidepressant classes, when comparing pooled estimates with those from the SNRI analyses. The difference in point estimates within the subgroup analysis could be reflecting the differing population types, as SSRIs are used as first line treatment for depression,(55) whereas SNRIs are often utilised in individuals with more severe depression or when first line options may have not worked.(56) Thus, these results provide evidence for the potential impact of confounding by depression severity, whereby the elevated pooled estimate from the SNRI analysis could be driven by non-exchangeability between individuals who use different classes of antidepressants. Confounding by disease severity was highlighted in the largest included study in the SNRI analysis which discussed that the risk difference observed between duloxetine (SNRI) and SSRI users could be depression severity and residual confounding.(48) However, given the confidence intervals overlap for the subgroup analyses of SSRIs and SNRIs, it is plausible that the true pooled estimates for each analysis are the same. Future network meta-analyses may help elucidate the intricacies of the relationship between individual antidepressant medications or classes and miscarriage.

Limitations of included studies

For peer review only - http://bmjopen.bmj.com/site/about/guidelines.xhtml

The included studies had several limitations. Firstly, only 7% of studies had a moderate overall RoB, due to the limited level of confounding adjustment in papers within the field. Only nine papers adjusted for any variable within all three key confounding domains (Supplementary Table S5). Therefore, the included studies may suffer from residual confounding, as strong risk factors for miscarriage, including cotreatments and comorbidities are likely imbalanced between exposure groups,(44) and any effect estimates may be biased away from the null. Additionally, we couldn't assess by length or severity of depressive disorder as this was rarely stated in the studies.

A second limitation of the included studies is information bias due to exposure misclassification. Exposure was assessed mainly via self-report from physician or individual, or prescription databases. However, only 60% agreement has been found between prescription and self-report data,(18) with conflicting evidence as to the most accurate method to assess exposure to medication.(57, 58) Teratogen surveillance data was used in several included studies, however, it poses difficulty as women enquiring about antidepressant exposure are automatically classified as exposed, when they may discontinue use after enquiry. Prescription database error could be less impactful, as it likely suffers from non-differential misclassification and biases the estimate towards the null.(18) This is shown in our sensitivity analysis that was conducted utilising only prescription database papers whereby results attenuated towards the null (Supplementary table S6), and could potentially be reflecting a more accurate measure of risk. Alternatively, self-report data could be more accurate in terms of ingested drug usage. Nevertheless, sensitivity analyses comparing self-report and prescription database inaccuracies indicate exposure misclassification does not impact effect estimates significantly, and only reduces precision.(18) Future studies should also look to report number of antidepressant prescriptions as this was rarely collected in previous studies, and is a crucial factor to be investigated.

Thirdly, selection bias is another common methodological limitation in this field. A significant issue observed is that the start of intervention and follow-up do not coincide. This results in conditioning on women who haven't experienced a miscarriage between the start of intervention and follow-up but excluding eligible individuals who have. Additionally,

For peer review only

exposed individuals are more likely to be in consultation with their healthcare professional when planning a pregnancy to discuss potential risks of antidepressants, compared to unexposed individuals. This results in exposed individuals having a longer observed risk period and as such, more chance of detecting and recording miscarriages, therefore biasing results away from the null. (59) These methodological errors contributing to a bias which occurs when individuals who have already experienced the outcome of interest at the point of study commencement not being included,(60) referred to as left truncation.(61)

Strengths and limitations of review

Our review has important strengths not addressed in any previous review investigating miscarriage. Confounding by indication and depression severity was a key part of the analysis and results, elucidating the true independent effect of antidepressant use during pregnancy. As this important bias has not previously been addressed in any other review in the field, it emphasises the requirement for accounting for psychiatric illness in future reviews and studies to avoid biased estimates. To the best of our knowledge, our study was also the first review in this field to assess RoB using the ROBINS-I tool. This tool gave a significant advantage over the commonly used Newcastle-Ottawa scale and highlighted the need for more thorough confounding adjustment and reporting of missing data in future studies. Additionally, the issues surrounding exposure recall bias were minimised using mainly cohort studies, and the included case-control studies having prospective exposure measurement. Finally, 29 studies published up to 2022 were identified and used in the review through an extremely thorough search strategy, thus enhancing statistical power and insight over previous reviews.

The review also had limitations. The studies included could suffer from publication bias as few studies were distributed around the null line and most found associations that transcended the null (Supplementary Figure S2). Additionally, small studies producing inverse relationships are absent from the field. This conclusion was supported by the small p-value produced in the Egger test. If only positive results are being published this could result in exaggerated review results. The authors didn’t include grey literature due to limited resource which could have also contributed to this finding. Alternatively, these

For peer review only

conclusions may not be driven by publication bias, but instead either real causal associations or lack of methodological rigour throughout the studies.

High heterogeneity also existed between papers, likely due to the included studies investigating different research questions, implementing different methodologies and populations, timing of exposure and confounding adjustment, thereby reducing the quality of the meta-analysis. Additionally, the lack of available data prevented a trimester specific subgroup analysis. Previous studies have identified first trimester miscarriage risk with antidepressant use to be higher compared to second trimester,(18) indicating antidepressant exposure could be time sensitive. However, this could reflect the time window for highest risk of miscarriage being between week 7 and 12, during first trimester.(62) Future reviews should aim to incorporate this subgroup analysis to investigate whether it alters conclusions made.

Conclusion

In conclusion, we did not find any clear evidence to suggest antidepressants increase the risk of miscarriage, though the results should be treated with caution due to the large heterogeneity present. This report highlights the effect of confounding by indication on previous literature. As RCTs cannot be utilised, future observational studies with larger sample sizes and more robust confounding adjustment, especially in relation to indication for treatment are needed. Triangulating results with differing methodologies should help strengthen causal inference. Additionally, network meta-analyses are needed to fully investigate the differing risk between antidepressant classes. Overall, the present study provides a summary of the most up-to-date findings of miscarriage risk following antidepressant use during pregnancy, to better inform clinicians and women when weighing up treatment for depression during pregnancy.

PROSPERO 2021 CRD42021262839 Available from: https://www.crd.york.ac.uk/prospero/display_record.php?ID=CRD42021262839

For peer review only

Declaration of interests
All authors declare no competing interests.

Funding
SS is supported by an NIHR predoctoral fellowship (NIHR301109). FM is supported by the Wellcome Trust (WT 218495/Z/19/Z). HF and DR acknowledge support from the NIH (1R01NS107607). This research was also supported by the NIHR Biomedical Research Centre at the University of Bristol and the University Hospitals Bristol and Weston NHS Foundation Trust.

Acknowledgements
The views in this research are of the authors and not of the funders. The authors thank the corresponding authors and coauthors of the studies included in this review. We thank Professor Julian Higgins who was extremely helpful with queries involving the ROBINS-I tool.

Author Contributions
All authors helped with interpretation of results. SS did the full-text screening, data extraction, risk of bias assessment, statistical analysis and wrote the report. FM did the title and abstract screening, full text screening, the second review of data extraction and risk of bias assessment, statistical analysis and had input on the report writing. HF did the title and abstract screening, full text screening, and had input on all other elements of the paper. DR suggested the research question, provided supervision and comment on the manuscript.

Data sharing statement
Please contact the first author (SS) if you would like to see any data not included in the article or supplementary material.

Figure captions

For peer review only

Figure 1. PRISMA flow diagram
Flow diagram illustrating the number of included and excluded studies at each stage of screening, and reasons for exclusion. This version has been adapted to include the element of additional papers found in following searches.

Figure 2. Subgroup meta-analyses utilising general population comparator

Three subgroup analyses conduced. First subgroup assessing any antidepressant use compared to unexposed individuals in general population. Second subgroup comparing SSRI use and unexposed general population individuals. Third subgroup comparing between SNRI use and unexposed general population individuals.

Figure 3. Three random-effects meta-analysis utilising depressed cohort comparator

First subgroup assessing any antidepressant use against unmediated depressed individuals. Second subgroup comparing SSRI use and different types of antidepressants (this includes data from papers which compared SSRIs and SNRIs, SSRIs and TCAs, SSRIs and any other antidepressants). Third subgroup analysis compared SNRI use and different types of antidepressants (this includes data from papers comparing SNRI and SSRI).

For peer review only

Uncategorized References

1. Disease GBD, Injury I, Prevalence C. Global, regional, and national incidence, prevalence, and years lived with disability for 354 diseases and injuries for 195 countries and territories, 1990-2017: a systematic analysis for the Global Burden of Disease Study 2017. Lancet. 2018;392(10159):1789-858.
2. Tak CR, Job KM, Schoen-Gentry K, Campbell SC, Carroll P, Costantine M, et al. The impact of exposure to antidepressant medications during pregnancy on neonatal outcomes: a review of retrospective database cohort studies. Eur J Clin Pharmacol. 2017;73(9):1055-69.
3. Rai D, Lee BK, Dalman C, Newschaffer C, Lewis G, Magnusson C. Antidepressants during pregnancy and autism in offspring: population based cohort study. Bmj. 2017;358:j2811.
4. Cooper WO, Willy ME, Pont SJ, Ray WA. Increasing use of antidepressants in pregnancy. Am J Obstet Gynecol. 2007;196(6):544 e1-5.
5. Pearson RM, Carnegie RE, Cree C, Rollings C, Rena-Jones L, Evans J, et al. Prevalence of Prenatal Depression Symptoms Among 2 Generations of Pregnant Mothers: The Avon Longitudinal Study of Parents and Children. JAMA Netw Open. 2018;1(3):e180725.
6. Ford E, Lee S, Shakespeare J, Ayers S. Diagnosis and management of perinatal depression and anxiety in general practice: a meta-synthesis of qualitative studies. Br J Gen Pract. 2017;67(661):e538-e46.
7. Quenby S, Gallos ID, Dhillon-Smith RK, Podesek M, Stephenson MD, Fisher J, et al. Miscarriage matters: the epidemiological, physical, psychological, and economic costs of early pregnancy loss. Lancet. 2021;397(10285):1658-67.
8. Hemels ME, Einarson A, Koren G, Lanctot KL, Einarson TR. Antidepressant use during pregnancy and the rates of spontaneous abortions: a meta-analysis. Ann Pharmacother. 2005;39(5):803-9.
9. Einarson A, Choi J, Einarson TR, Koren G. Rates of spontaneous and therapeutic abortions following use of antidepressants in pregnancy: results from a large prospective database. J Obstet Gynaecol Can. 2009;31(5):452-6.
10. Nakhai-Pour HR, Broy P, Berard A. Use of antidepressants during pregnancy and the risk of spontaneous abortion. Cmaj. 2010;182(10):1031-7.
11. Ewing G, Tatarchuk Y, Appleby D, Schwartz N, Kim D. Placental transfer of antidepressant medications: implications for postnatal adaptation syndrome. Clin Pharmacokinet. 2015;54(4):359-70.
12. Anderson HD, Pace WD, Libby AM, West DR, Valuck RJ. Rates of 5 common antidepressant side effects among new adult and adolescent cases of depression: a retrospective US claims study. Clin Ther. 2012;34(1):113-23.
13. Ornoy A, Koren G. Selective Serotonin Reuptake Inhibitors during Pregnancy: Do We Have Now More Definite Answers Related to Prenatal Exposure? Birth Defects Res Part A Clin Mol Teratol. 2017;109(12):898-908.
14. Keyte A, Hutson MR. The neural crest in cardiac congenital anomalies. Differentiation. 2012;84(1):25-40.
15. Marinescu IP, Foarfa MC, Pirlog MC, Turculeanu A. Prenatal depression and stress - risk factors for placental pathology and spontaneous abortion. Rom J Morphol Embryol. 2014;55(3 Suppl):1155-60.
16. Chan J, Natekar A, Einarson A, Koren G. Risks of untreated depression in pregnancy. Can Fam Physician. 2014;60(3):242-3.

For peer review only

17. Kjaersgaard MIS, Parner ET, Vestergaard M, Sorensen MJ, Olsen J, Christensen J, et al. Prenatal Antidepressant Exposure and Risk of Spontaneous Abortion - A Population-Based Study. PLoS ONE. 2013;8(8).
18. Johansen RL, Mortensen LH, Andersen AM, Hansen AV, Strandberg-Larsen K. Maternal use of selective serotonin reuptake inhibitors and risk of miscarriage - assessing potential biases. Paediatr Perinat Epidemiol. 2015;29(1):72-81.
19. Khalifeh H, Molyneaux E, Brauer R, Vigod S, Howard LM. Patient decision aids for antidepressant use in pregnancy: a pilot randomised controlled trial in the UK. BJGP Open. 2019.
20. Xing D, Wu R, Chen L, Wang T. Maternal use of antidepressants during pregnancy and risks for adverse perinatal outcomes: a meta-analysis. Journal of Psychosomatic Research. 2020;137 (no pagination).
21. Udechuku A, Nguyen T, Hill R, Szego K. Antidepressants in pregnancy: a systematic review. Aust N Z J Psychiatry. 2010;44(11):978-96.
22. Ross LE, Grigoriadis S, Mamisashvili L, Vonderporten EH, Roerecke M, Rehm J, et al. Selected pregnancy and delivery outcomes after exposure to antidepressant medication: a systematic review and meta-analysis. JAMA Psychiatry. 2013;70(4):436-43.
23. Higgins JPT, Cochrane Collaboration. Cochrane handbook for systematic reviews of interventions. Second edition. ed. Hoboken, NJ: Wiley-Blackwell; 2020. pages cm p.
24. Sterne JA, Hernan MA, Reeves BC, Savovic J, Berkman ND, Viswanathan M, et al. ROBINS-I: a tool for assessing risk of bias in non-randomised studies of interventions. Bmj. 2016;355:i4919.
25. Higgins JP, Thompson SG, Deeks JJ, Altman DG. Measuring inconsistency in meta-analyses. Bmj. 2003;327(7414):557-60.
26. Lin L, Chu H. Quantifying publication bias in meta-analysis. Biometrics. 2018;74(3):785-94.
27. Chambers CD, Johnson KA, Dick LM, Felix RJ, Jones KL. Birth outcomes in pregnant women taking fluoxetine. N Engl J Med. 1996;335(14):1010-5.
28. Kulin NA, Pastuszak A, Sage SR, Schick-Boschetto B, Spivey G, Feldkamp M, et al. Pregnancy outcome following maternal use of the new selective serotonin reuptake inhibitors: a prospective controlled multicenter study. Jama. 1998;279(8):609-10.
29. Einarson A, Fatoye B, Sarkar M, Lavigne SV, Brochu J, Chambers C, et al. Pregnancy outcome following gestational exposure to venlafaxine: a multicenter prospective controlled study. Am J Psychiatry. 2001;158(10):1728-30.
30. Einarson A, Bonari L, Voyer-Lavigne S, Addis A, Matsui D, Johnson Y, et al. A multicentre prospective controlled study to determine the safety of trazodone and nefazodone use during pregnancy. Canadian Journal of Psychiatry. 2003;48(2):106-10.
31. Sivojelezova A, Shuhaiber S, Sarkissian L, Einarson A, Koren G. Citalopram use in pregnancy: Prospective comparative evaluation of pregnancy and fetal outcome. American Journal of Obstetrics and Gynecology. 2005;193(6):2004-9.
32. Chun-Fai-Chan B, Koren G, Fayez I, Kalra S, Voyer-Lavigne S, Boshier A, et al. Pregnancy outcome of women exposed to bupropion during pregnancy: a prospective comparative study. Am J Obstet Gynecol. 2005;192(3):932-6.
33. Djulus J, Koren G, Einarson TR, Wilton L, Shakir S, Diav-Citrin O, et al. Exposure to mirtazapine during pregnancy: A prospective, comparative study of birth outcomes. J Clin Psychiatry. 2006;67(8):1280-4.

For peer review only

34. Paulus W, Schloemp S, Stoz F. Paroxetine exposure during pregnancy: Increased risk of congenital malformations? Archives of Gynecology and Obstetrics. 2010;282:S79.
35. Chan RL, Jonsson Funk ML, Savitz DA, Hartmann KE. Self-reported antidepressants use and spontaneous abortion. American Journal of Epidemiology. 2011;173:S140.
36. Einarson A, Choi J, Koren G, Einarson T. Outcomes of infants exposed to multiple antidepressants during pregnancy: results of a cohort study. J Popul Ther Clin Pharmacol. 2011;18(2):e390-6.
37. Ban L, Tata LJ, West J, Fiaschi L, Gibson JE. Live and non-live pregnancy outcomes among women with depression and anxiety: A population-based study. PLoS ONE. 2012;7(8).
38. Klieger-Grossmann C, Weitzner B, Panchaud A, Pistelli A, Einarson T, Koren G, et al. Pregnancy outcomes following use of escitalopram: a prospective comparative cohort study. J Clin Pharmacol. 2012;52(5):766-70.
39. Andersen JT, Andersen NL, Horwitz H, Poulsen HE, Jimenez-Solem E. Exposure to selective serotonin reuptake inhibitors in early pregnancy and the risk of miscarriage. Obstet Gynecol. 2014;124(4):655-61.
40. Abadie D, Hurault-Delarue C, Damase-Michel C, Montastruc JL, Lacroix I. Medication exposure and spontaneous abortion: A case-control study using a French medical database. Clinical and Experimental Obstetrics and Gynecology. 2015;42(4):431-6.
41. Te Winkel B, Beghin D, Pistelli A, De Santis M, Cassina M, Eleftheriou G, et al. Venlafaxine exposure in pregnancy, a multicenter ENTIS study. Pharmacoepidemiology and Drug Safety. 2016;25 (Supplement 3):325-6.
42. Almeida ND, Basso O, Abrahamowicz M, Gagnon R, Tamblyn R. Risk of Miscarriage in Women Receiving Antidepressants in Early Pregnancy, Correcting for Induced Abortions. Epidemiology. 2016;27(4):538-46.
43. Evans-Hoeker EA, Eisenberg E, Diamond MP, Legro RS, Alvero R, Coutifaris C, et al. Major depression, antidepressant use, and male and female fertility. Fertility and Sterility. 2018;109(5):879-87.
44. Richardson JL, Martin F, Dunstan H, Greenall A, Stephens S, Yates LM, et al. Pregnancy outcomes following maternal venlafaxine use: A prospective observational comparative cohort study. Reprod Toxicol. 2019;84:108-13.
45. Wu P, Velez Edwards DR, Gorrindo P, Sundermann AC, Torstenson ES, Jones SH, et al. Association between First Trimester Antidepressant Use and Risk of Spontaneous Abortion. Pharmacotherapy. 2019;39(9):889-98.
46. Bahat M, Shechtman S, Diav-Citrin O. Pregnancy outcome after first trimester exposure to Duloxetine. Reprod Toxicol. 2020;97:3.
47. Kolding L, Ehrenstein V, Pedersen L, Sandager P, Petersen OB, Uldbjerg N, et al. Antidepressant use in pregnancy and severe cardiac malformations: Danish register-based study. Bjog. 2021.
48. Ankarfeldt MZ, Petersen J, Andersen JT, Fernandes MFS, Li H, Motsko SP, et al. Duloxetine Exposure During Pregnancy and the Risk of Spontaneous and Elective Abortion: A Danish Nationwide Observational Study. Drugs Real World Outcomes. 2021;8(3):289-99.
49. Ostenfeld A, Petersen TS, Pedersen LH, Westergaard HB, Lokkegaard ECL, Andersen JT. Mirtazapine exposure in pregnancy and fetal safety: A nationwide cohort study. Acta Psychiatr Scand. 2022;145(6):557-67.
50. Kitchin A, Huerta C, Llorente-Garcia A, Martinez D, Ortega P, Cea-Soriano L. The role of prenatal exposure to antidepressants, anxiolytic, and hypnotics and its underlying illness

For peer review only

on the risk of miscarriage using BIFAP database. Pharmacoepidemiol Drug Saf. 2022;31(8):901-12.
51. Giner-Soriano MOP-VMLVCV-CCVAGG-LRM. Spontaneous abortion and drug exposure. Case-control study with SIDIAP database. Pharmaceoepidemiology and drug safety. 2022;Conference: 38th International Conference on Pharmacoepidemiology: Advancing Pharmacoepidemiology and Real-World Evidence for the Global Community. :238-9.
52. Page MJ, McKenzie JE, Bossuyt PM, Boutron I, Hoffmann TC, Mulrow CD, et al. The PRISMA 2020 statement: an updated guideline for reporting systematic reviews. Bmj. 2021;372:n71.
53. Rahimi R, Nikfar S, Abdollahi M. Pregnancy outcomes following exposure to serotonin reuptake inhibitors: a meta-analysis of clinical trials. Reprod Toxicol. 2006;22(4):571-5.
54. Nikfar S, Rahimi R, Hendoiee N, Abdollahi M. Increasing the risk of spontaneous abortion and major malformations in newborns following use of serotonin reuptake inhibitors during pregnancy: A systematic review and updated meta-analysis. Daru. 2012;20(1):75.
55. Kato T, Furukawa TA, Mantani A, Kurata K, Kubouchi H, Hirota S, et al. Optimising first- and second-line treatment strategies for untreated major depressive disorder - the SUND study: a pragmatic, multi-centre, assessor-blinded randomised controlled trial. BMC Med. 2018;16(1):103.
56. Isaac MT. Treating depression with SNRIs: who will benefit most? CNS Spectr. 2008;13(7 Suppl 11):15-21.
57. West SL, Savitz DA, Koch G, Strom BL, Guess HA, Hartzema A. Recall accuracy for prescription medications: self-report compared with database information. Am J Epidemiol. 1995;142(10):1103-12.
58. Hafferty JD, Campbell AI, Navrady LB, Adams MJ, MacIntyre D, Lawrie SM, et al. Self-reported medication use validated through record linkage to national prescribing data. J Clin Epidemiol. 2018;94:132-42.
59. Schisterman EF, Cole SR, Ye A, Platt RW. Accuracy loss due to selection bias in cohort studies with left truncation. Paediatr Perinat Epidemiol. 2013;27(5):491-502.
60. Cain KC, Harlow SD, Little RJ, Nan B, Yosef M, Taffe JR, et al. Bias due to left truncation and left censoring in longitudinal studies of developmental and disease processes. Am J Epidemiol. 2011;173(9):1078-84.
61. Howards PP, Hertz-Picciotto I, Poole C. Conditions for bias from differential left truncation. Am J Epidemiol. 2007;165(4):444-52.
62. Sanchez Ortiz S, Huerta C, Llorente-Garcia A, Ortega P, Astasio P, Cea-Soriano L. A Validation Study on the Frequency and Natural History of Miscarriages Using the Spanish Primary Care Database BIFAP. Healthcare (Basel). 2021;9(5).

For peer review only

For peer review only

PRISMA 2020 flow diagram for new systematic reviews which included searches of databases and registers only

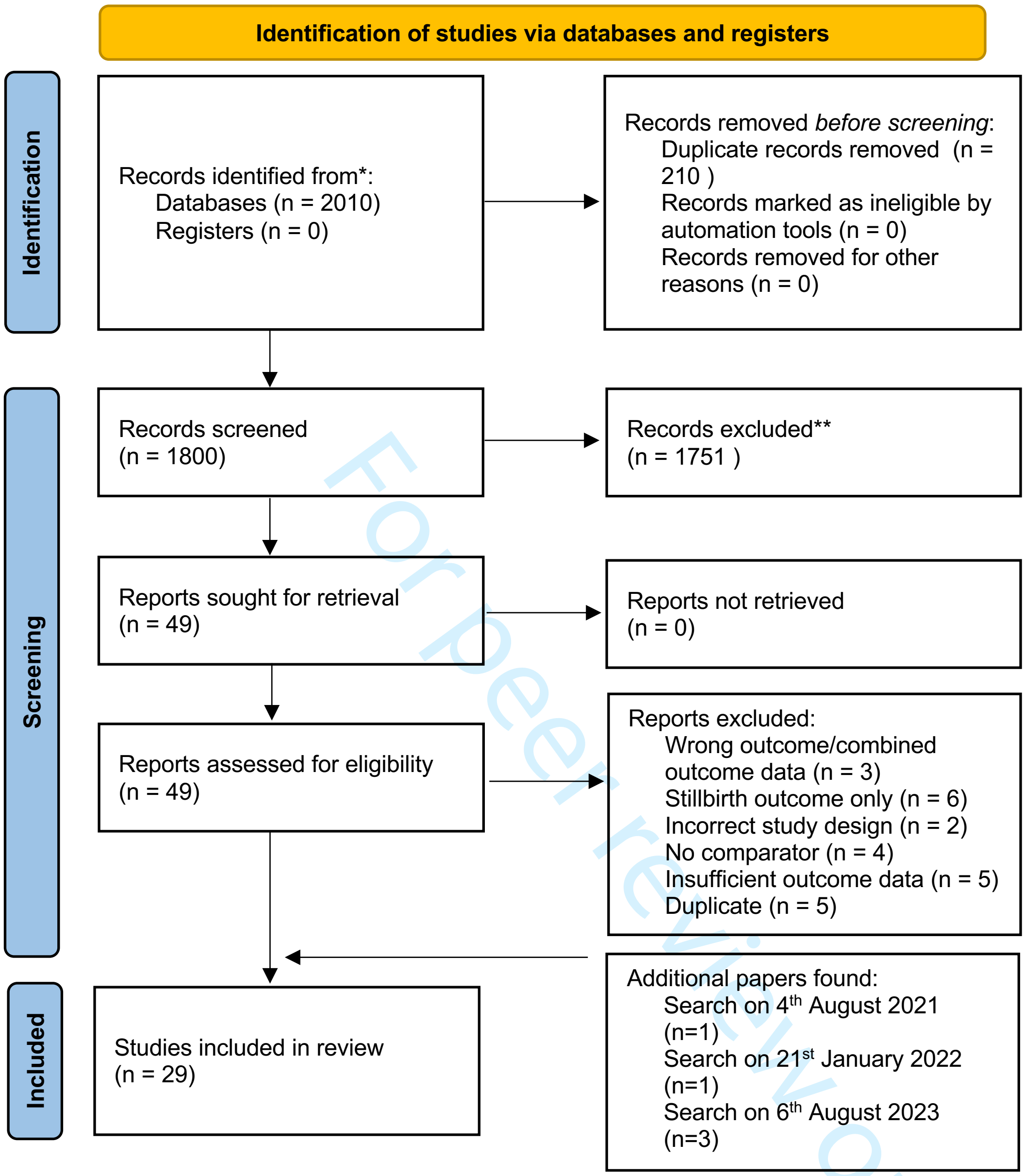

Figure 1. PRISMA flow diagram

Flow diagram illustrating the number of included and excluded studies at each stage of screening, and reasons for exclusion. This version has been adapted to include the element of additional papers found in following searches.

For peer review only

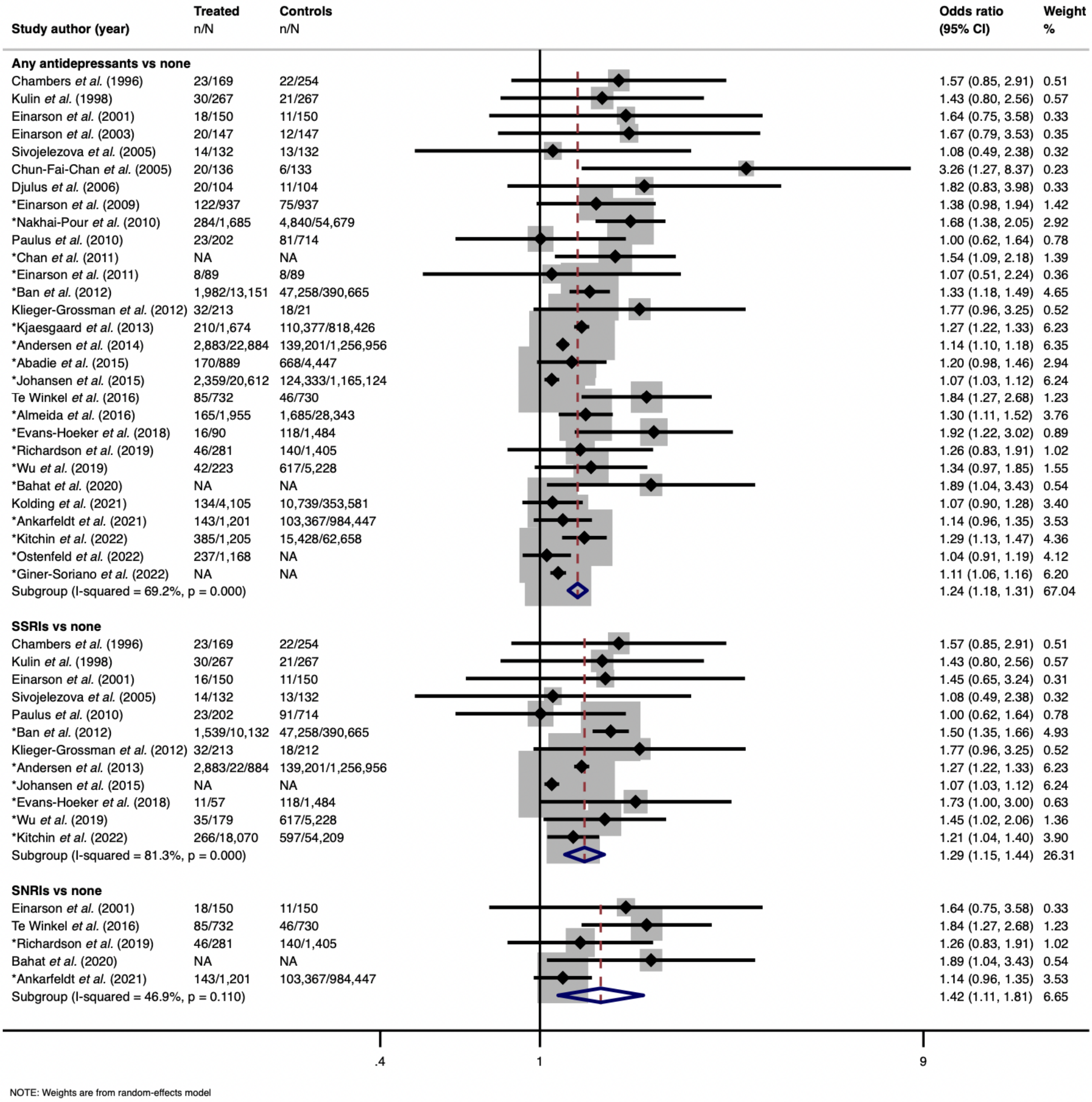

Figure 2. Subgroup meta-analyses utilising general population comparator

Three subgroup analyses conduced. First subgroup assessing any antidepressant use compared to unexposed individuals in general population. Second subgroup comparing SSRI use and unexposed general population individuals. Third subgroup comparing between SNRI use and unexposed general population individuals.

*These studies had an adjusted estimate from multivariable model which was pooled in the meta-analysis rather than raw data, therefore the treated and controls (n/N) data does not correspond to the odds ratio shown.
'The unexposed comparator in this paper was classified as 'duloxetine non-exposed' which could have also included users of other antidepressant types.

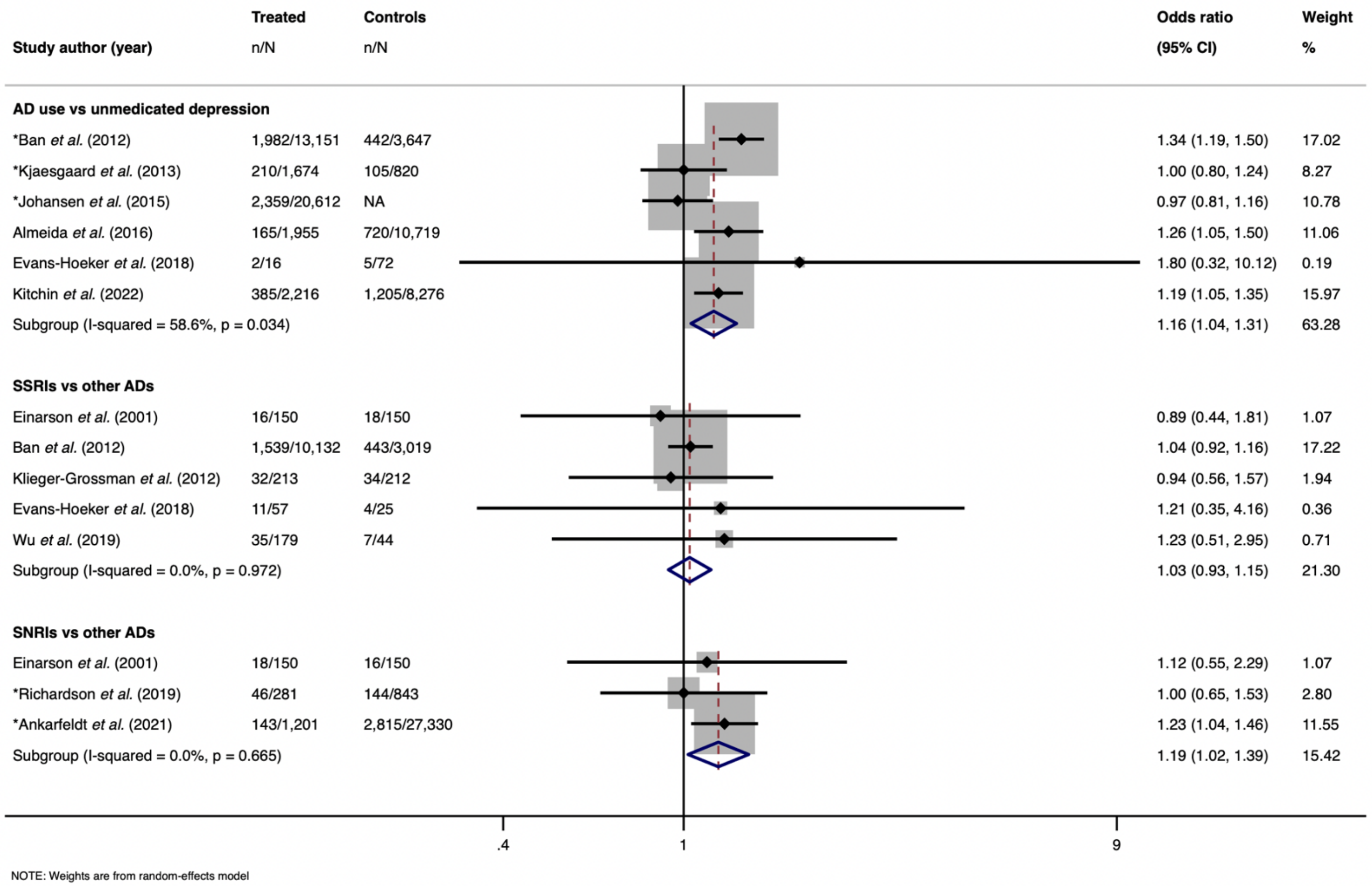

Figure 3. Three random-effects meta-analysis utilising depressed cohort comparator

First subgroup assessing any antidepressant use against unmediated depressed individuals. Second subgroup comparing SSRI use and different types of antidepressants (this includes data from papers which compared SSRIs and SNRIs, SSRIs and TCAs, SSRIs and any other antidepressants). Third subgroup analysis compared SNRI use and different types of antidepressants (this includes data from papers comparing SNRI and SSRI).

* These studies had an adjusted estimate from multivariable model which was pooled in the meta-analysis rather than raw data, therefore the treated and controls (n/N) data does not correspond to the odds ratio shown

For peer review only

EMBASE

Number	Search term	Outputs
1	exp antidepressant agent/	544895
2	Serotonin uptake inhibitor/	52823
3	Monoamine oxidase inhibitor/	15008
4	(anti*depress or antidepress* or tricyclic* or TCA* or heterocyclic* or serotonin uptake or SSRI* or SNRI* or monoamine oxidase inhibitor* or MAOI*).tw.	176810
5	(desipramine or imipramine or clomipramine or opipramol or trimipramine or lofepramine or dibenzepin or amitriptyline or nortriptyline or protriptyline or doxepin or iprindole or melitracen or butriptyline or dosulepin or amoxapine or dimetacrine or amineptine or maprotiline or quinupramine or zimeldine or fluoxetine or citalopram or paroxetine or sertraline or alaproclate or fluvoxamine or etoperidone or escitalopram or isocarboxazid or nialamide or phenelzine or tranylcypromine or iproniazide or iproclozide or moclobemide or toloxatone or oxitriptan or tryptophan or mianserin or nomifensine or trazodone or nefazodone or minaprine or bifemelane or viloxazine or oxaflozane or mirtazapine or bupropion or medifoxamine or tianeptine or pivagabine or venlafaxine or milnacipran or reboxetine or gepirone or duloxetine or agomelatine or desvenlafaxine or vilazodone).tw.	144967
6	1 or 2 or 3 or 4 or 5	673317
7	exp stillbirth/	22575
8	exp perinatal mortality/	27721
9	exp perinatal death/	4865
10	exp fetus death/	43412
11	(stillbirth or stillborn or perinatal death or peri-natal death or neonatal death or intrauterine death or intra-uterine death or newborn mortality).tw.	30264
12	exp spontaneous abortion/	49635
13	recurrent abortion/	7454
14	(Spontaneous abortion or Miscarriage$).tw.	39878
15	7 or 8 or 9 or 10 or 11	80549
16	12 or 13 or 14	62656

For peer review only

17	6 and 15	1176
18	6 and 16	1424
19	17 or 18	2340

MEDLINE

Number	Search term	Output
	exp Antidepressive Agents/	160660
	exp Serotonin Uptake Inhibitors/	46158
	exp Monoamine Oxidase Inhibitors/	22716
	(anti*depress or antidepress* or tricyclic* or TCA* or heterocyclic* or serotonin uptake or SSRI* or SNRI* or monoamine oxidase inhibitor* or MAOI*).tw.	132787
	(desipramine or imipramine or clomipramine or opipramol or trimipramine or lofepramine or dibenzepin or amitriptyline or nortriptyline or protriptyline or doxepin or iprindole or melitracen or butriptyline or dosulepin or amoxapine or dimetacrine or amineptine or maprotiline or quinupramine or zimeldine or fluoxetine or citalopram or paroxetine or sertraline or alaproclate or fluvoxamine or etoperidone or escitalopram or isocarboxazid or nialamide or phenelzine or tranylcypromine or iproniazide or iproclozide or moclobemide or toloxatone or oxitriptan or tryptophan or mianserin or nomifensine or trazodone or nefazodone or minaprine or bifemelane or viloxazine or oxaflozane or mirtazapine or bupropion or medifoxamine or tianeptine or pivagabine or venlafaxine or milnacipran or reboxetine or gepirone or duloxetine or agomelatine or desvenlafaxine or vilazodone).tw.	122965
	1 or 2 or 3 or 4 or 5	304323
	Stillbirth/	6346
	Perinatal Mortality/	2478
	exp Fetal Death/	31204
	PERINATAL DEATH/	2104
	(stillbirth or stillborn or perinatal death or peri-natal death or neonatal death or intrauterine death or intra-uterine death or newborn mortality).tw.	22241
	7 or 8 or 9 or 10 or 11	47887

For peer review only

	exp Abortion, Spontaneous/	38966
	(Spontaneous abortion or Miscarriage$).tw.	24795
	13 or 14	52292
	6 and 12	98
	6 and 15	202
	16 or 17	273

For peer review only

PSYCINFO

	Search terms	Outputs
	exp Antidepressant Drugs/	41562
	exp Serotonin Reuptake Inhibitors/	14269
	exp Monoamine Oxidase Inhibitors/	2318
	(anti*depress or antidepress* or tricyclic* or TCA* or heterocyclic* or serotonin uptake or SSRI* or SNRI* or monoamine oxidase inhibitor* or MAOI*).tw.	50486
	(desipramine or imipramine or clomipramine or opipramol or trimipramine or lofepramine or dibenzepin or amitriptyline or nortriptyline or protriptyline or doxepin or iprindole or melitracen or butriptyline or dosulepin or amoxapine or dimetacrine or amineptine or maprotiline or quinupramine or zimeldine or fluoxetine or citalopram or paroxetine or sertraline or alaproclate or fluvoxamine or etoperidone or escitalopram or isocarboxazid or nialamide or phenelzine or tranylcypromine or iproniazide or iproclozide or moclobemide or toloxatone or oxitriptan or tryptophan or mianserin or nomifensine or trazodone or nefazodone or minaprine or bifemelane or viloxazine or oxaflozane or mirtazapine or bupropion or medifoxamine or tianeptine or pivagabine or venlafaxine or milnacipran or reboxetine or gepirone or duloxetine or agomelatine or desvenlafaxine or vilazodone).tw.	35997
	(stillbirth or stillborn or perinatal death or peri-natal death or neonatal death or intrauterine death or intra-uterine death or newborn mortality).tw.	1195
	exp Spontaneous Abortion/	980
	(Spontaneous abortion or Miscarriage$).tw.	1706
	1 or 2 or 3 or 4 or 5	72741

	7 or 8	1981
	9 and 6	15
	9 and 10	46
	11 or 12	56

For peer review only

S2 Table. Excluded studies

Study excluded at full-text screening	Reason
Lennestal et al. (2007)	Stillbirth outcome only
Sjaarda et al. (2020)	Combined outcome of both stillbirth and miscarriage
Stephansson et al. (2013)	Stillbirth outcome only
Stephens et al. (2011)	Wrong study design
Wen et al. (2006)	Stillbirth outcome only
Winterfeld et al. (2015)	Combined outcome of both stillbirth and miscarriage
Pastuszak et al. (1993)	No outcome data
Ramio et al. (2007)	Wrong study design
Robinson-Wolrath et al. (2016)	No outcome data
Sjaarda et al. (2019)	Duplicate
Andersen et al. (2013)	Duplicate
Burian et al. (2016)	No outcome data
Einarson et al. (2011)	No comparator
Einarson et al. (2009)	Duplicate
Jordan et al. (2016)	Wrong outcome
Einarson et al. (2011)	No comparator
Evans-Hoeker et al. (2017)	Duplicate
Klieger-Grossman et al. (2010)	Duplicate
Goldstein et al. (1997)	No comparator
Hoog et al. (2013)	No comparator
Einarson et al. (2012)	No outcome data
Innocenti et al. (2019)	No outcome data
Jimenez-Solem et al. (2014)	Stillbirth outcome only
Jimenez-Solem et al. (2013)	Stillbirth outcome only
Ericson et al. (1999)	Stillbirth outcome only

For peer review only

Table S3. Characteristics of included studies

Study	Study design	Country of study & study period	Data sampling methods & mean age of individuals	Type of AD exposure	Timing of AD exposure	Definition of exposure and comparator groups	Identification of exposure	Identification of outcome	Sample size	Confounding adjustments	Raw data (no. of SA/ total)	Adjusted estimates
Chambers et al. (1996) [1]	Prospective cohort	California 1989-1995	TIS data collection<br><br>**Exposed early:** 32+-5<br>**Exposed late:** 32+-6<br>**Control:** 30+-5	Fluoxetine (SSRI)	Anytime	**Exposed:** Fluoxetine use<br>**Comparator group 1:** women enquiring about non-teratogens	Telephone interviews	Telephone interview and confirmed by physician	482	Maternal age, average dose of fluoxetine	**Exposed early** = 23/169<br>**Control group** = 22/254	NA
Kulin et al. (1998) [2]	Prospective cohort	US&Canada	TIS data<br><br>**Exposed :** mean = 31.3<br>**Unexposed:** mean = 30.8	SSRI	First trimester	**Exposed:** SSRI use during 1st trimester<br>**Comparator group 1:** Women enquiring about non-teratogens	Telephone interview	After expected date of delivery women were interviewed	534	None	**Exposed** = 30/267<br>**Control** = 21/267	NA
Einarson et al. (2001) [3]	Cohort	European and non-european countries	Women contacting motherisk program around exposure advice<br><br>NA	Venlafaxine (SNRI)	Anytime	**Exposed:** Venlafaxine use during 4th-14th week of pregnancy<br>**Comparator group 1:** SSRI users<br>**Comparator group 2:** women making non-teratogenic enquiries	Once contacted the service women were interviewed	Follow-up questionnaire after delivery and information verified by physician	450	None	**Venlafaxine** = 18/150<br>**SSRI comparator** = 16/150<br>**Nonteratogenic comparator** = 11/150	NA
Einarson et al. (2003)[4]	Prospective cohort	European and non-european countries	Women contacting motherisk program<br><br>NA	Trazodone and Nefazodone (NaSSA)	First trimester	**Exposed:** Use of NaSSAs during 4th-14th week<br>**Comparator group 1:** Women taking other ADs<br>**Comparator group 2**: non-teratogen drug exposures	Questionnaire	Follow-up questionnaire after delivery and verified by physician	441	Matched on gestational age	**Trazodone/Nefazodone** = 20/147<br>**Group 1 (other Ads)** = 17/147<br>**Group 2 – nonteratogens** = 12/147	NA
Sivojelezova et al. (2005) [5]	Prospective cohort	Toronto 1999-2002	Women contacting motherisk program<br><br>Range of 18-42 and mean of 31.9	Citalopram (SSRI)	Anytime	**Exposed:** Use of citalopram<br>**Comparator group 1:** pregnant women taking other SSRI Ads<br>**Comparator group 2:** non-teratogenic drug use	Telephone interview to individuals contacting the service	Follow-up questionnaire after delivery and verified by physician	396	None	**Exposed** = 14/132<br>**Comparison 1** = 13/132<br>**Comparison 2** = 13/132	NA

For peer review only

Chun-Fai Chan et al. (2005) [6]	Cohort	European and non-european countries	Individuals contacting motherisk or recruited through drug safety research unit<br><br>NA	Bupropion (NDRI)	Anytime	**Exposed:** Bupropion exposed pregnancies<br>**Comparator group 1:** Pregnant women taking other Ads<br>**Comparator group 2:** individuals taking non-teratogens	Telephone administration of follow-up questionnaire	Follow-up questionnaire after delivery and verified by physician	NA	Matched on age, alcohol consumption, smoking and gestational age	**Bupropion =** 20/136<br>**Comparison 1 (other Ads) =** 11/89<br>**Comparison 2 (nontertaogens) =** 6/133<br>**Women exposed to bupropion for depression =** 14/91	NA
Djulus et al. (2006) [7]	Cohort	European and non-european countries 2002-2005	Pregnant women contacting one of five evidence based information centres<br><br>Ages were consistent between groups due to matching	Mirtazapine (NaSSA)	Anytime	**Exposed:** pregnant women taking bupropion<br>**Comparator group 1:** disease matched individuals taking other Ads<br>**Comparator group 2:** individuals taking known non-teratogens	Telephone interview regarding exposure	Contacted 2-6 months after delivery and details verified by physician	312	Matched on maternal age, gestational age, tobacco use, alcohol consumption and chronic conditions	**Mirtazapine =** 20/104<br>**Comparison 1 (other AD) =** 18/104<br>**Comparison 2 (nonteratogenics)** = 11/104	NA
Einarson et al. (2009) [8]	Prospective cohort	Toronto	women contacting motherisk program<br><br>NA	Any AD	Anytime	**Exposed:** Pregnant women contacting program regarding exposure during pregnancy<br>**Comparator group 1:** Women exposed to non-teratogenic drugs<br>**Comparator group 2:** unexposed to Ads	Telephone interview using standardised questionnaire	Follow-up structured form	1874	Matched for maternal age, smoking, and alcohol use, gestational age	**Exposed =** 122/937<br>**Unexposed =** 75/937	RR = 1.38 (0.98-1.94)
Nakhai-Pour et al. (2010) [9]	Nested case-control	Quebec 1998-2003	Data from Quebec pregnancy register with individuals registered between 1998-2003, including RAMQ database<br><br>NA	Any AD	Any time between first day gestation and index date of SA	**Exposed:** At least one prescription filled between start of gestation and SA index date<br>10 controls sampled for every SA case	RAMQ database which collects information on prescriptions, and pregnancy register to identify unexposed	Quebec pregnancy register entered between 1998-2003	56364	Adjusted for age, urban residence, social assistance, psychiatric diagnosis, comorbid illness, medication use, gestational age	**Cases** = 284 individuals used antidepressants<br>**Controls =** 1401 used antidepressants	**Crude OR =** 2.09 (1.83-2.38)<br>**Adjusted OR =** 1.68 (1.38-2.06)<br>**Adjusted SSRI alone** = 1.61 (1.28-2.04)<br>**Adjusted TCA alone** = 1.27 (0.85-1.91)
Paulus et al. (2010) [10]	Prospective cohort: Conference abstract	Germany	National TIS who were contacted by	Paroxetine (SSRI)	First trimester	**Exposed:** Paroxetine exposure in first trimester	Patients or physicians contact TIS	NA	955	None	**Exposed** = 23/202<br>**Unexposed =** 81/714	NA

			physicians or patients			**Comparator group 1:** Not exposed or 'severely exposed' to paroxetine						
Chan et al. (2011) [11]	Cohort – conference abstract	US 2000-2008	Recruited from 4 US metropolitan areas	All Ads	Anytime	**Exposed:** Antidepressant use during pregnancy **Comparator:** NA	Telephone interviews and medical record abstractions	Telephone interviews and medical record abstractions	4536	Maternal age, race, education, BMI, smoking, alcohol use, gravidity and nausea	NA	**Adjusted OR of exposed vs unexposed** = 1.54 (1.1-2.2) **Adjusted OR of SSRI users vs unexposed** = 1.67
Einarson et al. (2011) [12]	Prospective cohort	Toronto 1992-2007	women contacting motherisk	All Ads	Anytime	**Exposed:** exposed to more than one AD during pregnancy **Comparator group 1:** Exposed to single AD during pregnancy **Comparator group 2:** contacting motherisk regarding non-teratogenic exposure	telephone interview using standardised questionnaire	Follow-up interview details verified from report by physician	267	Matched on maternal age, smoking and alcohol use and gestational age	**Multiple AD exposure** = 8/89 **Single AD exposure** = 7/89 **No AD exposure** = 8/89	**OR for multiple AD exposure vs single exposure** = 1.16 (0.4-3.34) **OR for multiple AD exposure vs no exposure** = 1.00 (0.36-2.79)
Ban et al. (2012) [13]	Cohort	UK 1990-2009	Data collected from primary care medical records (through health improvement network - THIN) <br>> 50% of the women aged between 25-34	TCAs and SSRIs	First trimester	**Exposed:** depression and exposed to AD during first 90 days after conception **Comparator group 1L** No history of depression and unexposed **Comparator group 2:** history of depression and unexposed **Comparator group 3:** prescriptions for TCAs only **Comparator group 4:** prescriptions for SSRIs only **Comparator group 5:** prescriptions for any other AD only **Comparator group 6:** prescription for psychotropic drugs only	Extracted prescription records from THIN	Identified through THIN database	331414	Adjusted for maternal age, SES, maternal smoking status and BMI	**Reference category (group 1)** = 47258/390665 **History of mental illness only** = 13814/99932 **Unmedicated current mental illness** = 442/3647 **TCAs** = 443/3019 **SSRIs**= 1539/10132	**Adjusted RRR with comparison being no current/past depression or anxiety** **History of mental illness only:** 1.2 (1.2-1.2) **Unmedicated mental illness** = 1.0 (0.9-1.2) **TCAs** = 1.3 (1.1-1.5) **SSRIs** = 1.5 (1.3-1.6) <br>**Adjusted RRR when compared to unmedicated antenatal depression or anxiety** **TCAs** = 1.3 (1.1-1.5) **SSRIs** = 1.4 (1.2-1.7)
Klieger-Grossman et al. (2012) [14]	Prospective cohort	Toronto, Switzerland and Florence	Data collected from women ringing TIS in the three countries	Escitalopram (SSRI)	Anytime	**Exposed:** pregnant women exposed to escitalopram **Comparator group 1:** contacting service exposed to other Ads	Information taken during telephone conversation through standardised questionnaire	Follow-up questionnaire 2-3 months after delivery	637	Matched on maternal age, alcohol consumption, smoking and gestational age	**Escitalopram** = 32/213 **Other AD use** = 34/212 **Nonteratogens** = 18/212	NA

For peer review only

			Mean age of all women in study = 33.1			**Comparator group 2:** nonteratogenic exposures						
Kjaesgaard et al. (2013) [15]	Population based cohort	Denmark 1997-2008	Data obtained from Danish health registries<br><br>**Depression and no AD use:** mean = 29.1<br>**Use of AD (exposed):** mean = 30.9<br>**Unexposed:** mean = 30.2	All Ads	Anytime	**Exposed:** mother redeemed prescription for AD<br>**Comparator group 2:** mother hadn't redeemed prescription	Information from registry of medicinal product statistics	Identified through codes using Danish national hospital registry	1005319	Adjusted for maternal age, cohabitation, income, education, history of mental disorder or drug abuse	**Diagnosis of depression and exposed to AD** = 210/1674<br>**Diagnosis of depression and unexposed to AD**= 105/820<br>**No diagnosis of depression and AD use**= 2427/13789<br>**No diagnosis of depression and unexposed**= 110377/818426	**Unadjusted RR when compared to unexposed equivalent:**<br>**Diagnosis of depression exposed to AD** = 0.98 (0.78-1.23)<br>**No diagnosis of depression and AD use** = 1.31 (1.26-1.35)<br><br>**Adjusted RR (when compared to unexposed equivalent):**<br>**Diagnosis of depression and exposed** = 1.00 (0.8-1.24)<br>**No diagnosis of depression and AD use** = 1.17 (1.13-1.22)
Andersen et al. (2014)[16]	Cohort	Denmark 1997-2010	Identified all registered pregnancies through national hospital register	SSRIs	First 35 days of pregnancy	**Exposed:** Dispensing of prescription of SSRIs<br>**Comparator group 1:** Women exposed to SSRIs prior to pregnancy<br>**Comparator group 2:** women unexposed to SSRIs	Data collected from national prescription register containing individual level data on prescribed drugs	Identified through national hospital register using codes	1279840	Adjusted for maternal age, previous SA, income, year of outcome, educational length, antipsychotic s and thyroid preparations	**Unexposed**= 139201/1256956<br>**Exposed to SSRI during first 35 days of pregnancy:** 2883/22884<br>**Discontinuation of SSRI treatment 3-12 months before conception** = 1936/14016	**Unadjusted HR:**<br>**Exposed during first 35 days vs unexposed:** 1.31 (1.27-1.36)<br>**Discontinued SSRI treatment 3-12 months before pregnancy vs unexposed:** 1.38 (1.32-1.44)<br><br>**Adjusted HR:**<br>**Exposed to SSRI during first 35 days vs unexposed:** 1.27(1.22-1.33)<br>**Discontinued use of SSRI before conception vs unexposed:** 1.24 (1.18-1.30)

For peer review only

Abadie et al. (2015)[17]	Nested case-control	France	French database of TERAPPEL used where healthcare professionals contact French regional pharmacovigilance centres (CPRV)<br><br>**Mean of cases** = 32.4<br>**Mean of controls** = 30.6	All Ads	First trimester	**Exposed:** First trimester drug exposure and called CPRV enquiring about drug exposure<br>**Comparator group 1:** Calling CPRV for assessing non-teratogen exposure	Healthcare professionals contacting CPRVs	Women registered to TERAPPEL database	5346	Maternal age	**Exposed to any AD**<br>**Cases of SA** = 170/838<br>**No SA** = 729/4508<br><br>**Exposed to non-selective monoamine reuptake inhibitors (NMRI)**<br>**SA** = 39/838<br>**No SA** = 88/4508	**Crude OR for all Ads** = 1.32 (1.1-1.59)<br>**Adjusted OR for all Ads** = 1.2 (0.98-1.46)<br><br>**Crude OR for NMRI**= 2.45 (1.67-3.60)<br>**Adjusted OR for NMRI** = 2.19 (1.46-2.39)
Johansen et al. (2015) [18]	Register based study cohort	Denmark 1996-2009	study population with registered pregnancies between 1996-2009 and Danish national birth cohort	SSRI	Anytime but results split into first and second trimester	**Exposed:** Redeemed prescription in time frame of month prior to LMP to 3 months after LMP or end of pregnancy (also self-report)<br>**Comparator group 1:** discontinued use of SSRIs with same LMP prescription timeframe<br>**Comparator group 2:** unexposed and no prescription	Redeemed prescriptions from Danish national prescription database	Data from Danish national patient register	1281418	Adjustment for household income, education, previous SA, parity and maternal age	**Unexposed** = 124333/1165124<br>**Discontinued use** = 670/5428<br>**Exposed** = 2359/20612	**Combined first and second trimester HR**<br><br>**Exposed vs unexposed general population** = 1.07 (1.03-1.12)<br>**SSRI use vs unmedicated depressed** = 0.97 (0.81-1.16)
Te Winkel et al. (2016) [19]	Prospective cohort – conference abstract only	Across European countries	Data from nine centres which make up european network of TIS<br><br>Mean: 31.9	Venlafaxine (SSRI)	Anytime	**Exposed:** Venlafaxine exposure<br>**Comparator group 1:** No exposure to any known teratogens	TIS data	using standardised procedures for data collection and follow-up	1462	None	**Exposed** = 85/732<br>**Unexposed** = 46/730	NA
Almeida et al. (2016) [20]	Retrospective cohort	Quebec 1998-2002	Linking four population-based registries and producing longitudinal histories of medication use	All Ads	First trimester	**Exposed:** Women who have at least one AD prescription in first trimester of pregnancy<br>**Comparator groups 1:** Unexposed but depressed in last four years<br>**Comparator group 2:** non-teratogen group	Prescription claims database	ICD-9 codes	41964	Adjustment for maternal age, welfare recipient, other teratogenic medication, no. medications any hospitalisatio	**No medication and no depression:** 1685/28343<br>**No AD but depression** - 720/10719<br>**AD use in first trimester and depression** - 165/1955	**All adjusted RRR**<br><br>**Unexposed and no depression** = 1.00<br>**Unexposed and depressed** = 1.1 (1.0-1.2)<br>**Exposed and depressed** = 1.3 (1.1-1.5)

For peer review only

						**Comparator group 3:** individuals with no prescriptions or depression				n or physician visits, calendar year	**Hypothyroid medication use in first trimester:** 71/947	
Evans-Hoeker et al. (2018) [21]	Cohort	US	RCT was conducted on non-IVF fertility treatment and individuals from trial were brought into cohort study	All Ads – split into SSRI and non-SSRI	Anytime	**Group 1:** Absence of major depression(MD) and no AD use<br>**Group 2:** Absence of MD and using AD<br>**Group 3:** Presence of MD and no AD use<br>**Group 4:** MD & using Ads	Medication exposure assessed through RCT and PH-9 questionnaire used	Identification through RCT	1650	Adjusted for age, race, income, current smoking, depression severity and trial placed in for RCT	**PH-9<10 and unexposed =** 110/1359<br>**PH-9<10 and exposed =** 14/73<br>**PH-9>10 and unexposed =** 5/72<br>**PH-9>10 and exposed =** 2/16<br><br>**No AD use =** 118/1484<br>**Any AD use =** 16/90<br>**SSRI use =** 11/57<br>**Non-SSRI use=** 4/25<br>**Both use of SSRI and non-SSRI =** 1/8	<u>**Adjusted RR:**</u><br>**Ph-9<10 and unexposed =** reference<br>**PH-9<10 and exposed =** 1.87 (1.18-2.99)<br>**PH-9>10 and unexposed =** 0.85 (0.37-1.96)<br>**PH-9>10 and exposed =** 1.4 (0.47-4.17)<br><br><u>**specific medication types:**</u><br>**Any AD use =** 1.92 (1.22-3.02)<br>**SSRI use =** 1.73 (1.0-3.0)<br>**Non-SSRI use=** 3.45(1.99-5.98)<br>**Both SSRI and non-SSRI =** 1.15 (0.19-7.06)
Richardson et al. (2019) [22]	Cohort	UK	Using UK collected teratogen surveillance data<br><br>Median: 31 for both groups	Venlafaxine (SNRI)	Anytime	**Exposed:** Individuals using venlafaxine at any point during pregnancy<br>**Comparator group 1:** Unexposed to any AD medication (5:1)<br>**Comparator group 2:** disease matched comparator of SSRI AD exposed	Surveillance data from UKTIS	Postal questionnaire sent after delivery date from healthcare professional who contacted UKTIS	2529	Adjusted for history of SA, tobacco exposure, alcohol, recreational drugs and folic acid	**Venlafaxine =** 46/281<br>**SSRI exposed =** 114/843<br>**Unexposed =** 140/1405	**Crude OR for unexposed =** 1.57 (1.06-2.30)<br>**Crude OR for SSRI comparator =** 1.05 (0.7-1.57)<br><br>**Adjusted HR for unexposed =** 1.26 (0.829-1.91)<br>**Adjusted HR for SSRI comparator =** 1.00 (0.655-1.53)
Wu et al. (2019) [23]	Prospective cohort	North Carolina, Tennessee and Texas	Enrolment into community-based cohort<br><br>NA	All AD – classified into SSRI and non-SSRI	First trimester	**Exposed:** reported AD use since pregnancy and before interview<br>**Comparator group 1:** Never used AD	community-based cohort – AD use assessed during interview	Self-reported pregnancy outcomes-verified by medical records	5451	Adjustment for maternal age, BMI, caffeine consumption, smoking, alcohol, race, SA history	**Exposed to any AD =** 42/223<br>**Use of SSRIs =** 35/179<br>**Exposure to non-SSRIs =** 7/44<br>**Unexposed =** 617/5228	**Unadjusted HR:**<br><br>**Any AD =** 1.51 (1.10-2.06)<br>**Only SSRIs =** 1.59 (1.13-2.23)<br>**Only non-SSRIs = 1.21** (0.57-2.56)

For peer review only

												**Adjusted HR**<br>**Any AD use:** 1.34 (0.97-1.85)<br>**SSRI only:** 1.45 (1.02-2.06)<br>**Non-SSRI only:** 0.96 (0.45-2.04)
Bahat et al. (2020) [24]	Cohort – conference abstract only	Israel 2001-2015	Data utilised from Israeli TIS<br><br>NA	Duloxetine (SNRI)	First trimester	**Exposed:** Exposure to duloxetine during first trimester<br>**Comparator group 1:** women exposed to TCAs<br>**Comparator group 2:** non-teratogenic exposure	Information taken when contacted TIS	Identified through follow-up after delivery date	722	None	NA	**Adjusted HR** = 1.89 (1.04-3.43)
Kolding et al. (2021)[25]	Register based study	Denmark 2007-2014	Routinely collected data from five nationwide registries and databases<br><br>NA	All Ads	First trimester	**Exposed:** two or more redeemed prescriptions<br>**Comparator group 1:** Absence of redemptions for AD	Using redeemed prescriptions through linkage to Danish health services prescription database	Identified through Danish national patient registry	364012	None	**Exposed** = 134/3971<br>**Unexposed** = 10739/342842	NA
Ankarfeldt et al. (2021)[26]	Prospective cohort	Denmark 2004-2016	Study population used danish registry data whereby women were included if they had a recorded birth in birth register or an abortion in patient register between 2004-2016<br><br>NA	Duloxetine (SNRI)<br>SSRIs<br>Venlafaxine (SNRI)	Anytime during pregnancy	**Exposed** = redemption of AD prescription in window from 30 days prior to LMP to 140 days post LMP or end of pregnancy, whichever came first<br>**Comparator group 1:** Duloxetine non-exposed (no redemption of prescription during the window)<br>**Comparator group 2:** SSRI exposed<br>**Comparator group 3:** Venlafaxine exposed	Using the Danish national prescription register	Identified through either the birth registry for all births, or the patient register for abortions.	1020288	birth year, maternal age, psychiatric hospitalization, household income, education, comorbidities, co-medication	**Unadjusted:**<br>**Duloxetine** = 145/1212<br>**Duloxetine unexposed** = 106309/1018745<br>**SSRI** = 2900/28345<br>**Venlafaxine** = 497/4908	**Adjusted**<br><br>**Duloxetine vs unexposed** = 1.14 (0.96-1.34)<br>**Duloxetine vs SSRI** = 1.23 (1.04-1.46)<br>**Duloxetine vs venlafaxine** = 1.18 (0.98-1.42)<br><br>**PS-matched**<br><br>**duloxetine vs unexposed** = 1.08 (0.89-1.31)<br>**Duloxetine vs SSRI** = 1.25 (1.00-1.57)<br>**Duloxetine vs venlafaxine** = 1.08(0.82-1.41)

Ads=antidepressants, SSRIs = selective serotonin reuptake inhibitors, SNRIs= serotonin-norepinephrine reuptake inhibitors, TCAs= tricyclic antidepressants, NDRI = norepinephrine and dopamine reuptake inhibitors, NaSSA = noradrenergic and serotonergic specific antidepressants, RCT= randomized controlled trial, TIS=teratogen information service, NA= not available, MD = major depression

For peer review only

Kitchin et al. (2022)[27]	Nested case-control	Spain<br><br>2002-2015	Cohort included women of childbearing age (15-49) with at least 1 year of registration with their primary care practitioner between Jan 2002 and Dec 2015.	All ADs	Anytime during pregnancy	**nonusers:** when there were no prescriptions in any of these periods<br><br>**discontinuers:** women who had at least one prescription only during the prepregnancy period;<br><br>**continuers:** women who had at least one prescription during the prepregnancy period and also during the first trimester<br><br>**new users:** women who had at least one pre- scription only during the first trimester.	Spanish database BIFAP	Miscarriage identified via ICD codes in the BIFAP database	72,280	Number of GP visits, obesity, smoking, HTA and diabetes	**Discontinued AD use vs no use:** 2216/8276 vs 15428/62658, **Continued AD use vs no use:** 385/1205 vs 15428/62658, **Incident AD use vs no use:** 41/140 vs 15428/62658	**ORs - Discontinued AD use vs no use:** 1.05 (1.00-1.11) **Continued AD use vs no use:** 1.29 (1.13-1.46) **Incident AD use vs no use:** 1.12 (0.78-1.62)
Ostenfeld et al. (2022)[28]	Cohort	Denmark 1997-2016	Study population using Danish registry data – with the study population being all pregnancies registered in the medical birth register or national hospital register (between 1st jan 1997 to 31st Dec 2016)<br><br>NA	Mirtazapine (NaSSA)	Exposure earlier than 22 weeks of completed gestation	**Exposed:** One filled prescription of mirtazapine	Using the register for of medicinal product statistics.	Identified in the National Hospital Register of Denmark	9,500	Age at pregnancy onset, marital status, place of birth, region of residence, gross household income, education status, parity, multiple birth pregnancy, smoking during pregnancy, previous pregnancy with adverse fetal outcome, prescription drug use in past year and hospital care utilization in past year	**Mirtazepine exposed vs unexposed:** 237/1168	**HRs: Mirtazepine exposed vs unexposed:** 1.04 (0.91-1.20)

For peer review only

Giner-Soriano et al. (2022)[29]	Case-control	Spain	Pregnancy episodes of women aged 12-50 from SIDIAP database during 2011-2020<br><br>NA	NA	Exposure up to 120 days after pregnancy start date or at SA date	**Exposure:** dispensation of AD 30 days before to 120 days after pregnancy start date or at spontaneous abortion date, whichever occurs first	SIDIAP – contains primary care health data records	Primary healthcare records of the Catalan Health Institute	180,692	NA	NA	**ORs - antidepressants vs no antidepressants:** 1.11 (1.06-1.16)

1

For peer review only

References

1. Chambers CD, Johnson KA, Dick LM, Felix RJ, Jones KL. Birth outcomes in pregnant women taking fluoxetine. *N Engl J Med* 1996; **335**(14): 1010-5.

2. Kulin NA, Pastuszak A, Sage SR, et al. Pregnancy outcome following maternal use of the new selective serotonin reuptake inhibitors: a prospective controlled multicenter study. *Jama* 1998; **279**(8): 609-10.

3. Einarson A, Fatoye B, Sarkar M, et al. Pregnancy outcome following gestational exposure to venlafaxine: a multicenter prospective controlled study. *Am J Psychiatry* 2001; **158**(10): 1728-30.

4. Einarson A, Bonari L, Voyer-Lavigne S, et al. A multicentre prospective controlled study to determine the safety of trazodone and nefazodone use during pregnancy. *Canadian Journal of Psychiatry* 2003; **48**(2): 106-10.

5. Sivojelezova A, Shuhaiber S, Sarkissian L, Einarson A, Koren G. Citalopram use in pregnancy: Prospective comparative evaluation of pregnancy and fetal outcome. *American Journal of Obstetrics and Gynecology* 2005; **193**(6): 2004-9.

6. Chun-Fai-Chan B, Koren G, Fayez I, et al. Pregnancy outcome of women exposed to bupropion during pregnancy: a prospective comparative study. *Am J Obstet Gynecol* 2005; **192**(3): 932-6.

7. Djulus J, Koren G, Einarson TR, et al. Exposure to mirtazapine during pregnancy: A prospective, comparative study of birth outcomes. *J Clin Psychiatry* 2006; **67**(8): 1280-4.

8. Einarson A, Choi J, Einarson TR, Koren G. Rates of spontaneous and therapeutic abortions following use of antidepressants in pregnancy: results from a large prospective database. *J Obstet Gynaecol Can* 2009; **31**(5): 452-6.

9. Nakhai-Pour HR, Broy P, Berard A. Use of antidepressants during pregnancy and the risk of spontaneous abortion. *Cmaj* 2010; **182**(10): 1031-7.

10. Paulus W, Schloemp S, Stoz F. Paroxetine exposure during pregnancy: Increased risk of congenital malformations? *Archives of Gynecology and Obstetrics* 2010; **282**: S79.

11. Chan RL, Jonsson Funk ML, Savitz DA, Hartmann KE. Self-reported antidepressants use and spontaneous abortion. *American Journal of Epidemiology* 2011; **173**: S140.

12. Einarson A, Choi J, Koren G, Einarson T. Outcomes of infants exposed to multiple antidepressants during pregnancy: results of a cohort study. *J Popul Ther Clin Pharmacol* 2011; **18**(2): e390-6.

13. Ban L, Tata LJ, West J, Fiaschi L, Gibson JE. Live and non-live pregnancy outcomes among women with depression and anxiety: a population-based study. *PLoS ONE* 2012; **7**(8): e43462.

14. Klieger-Grossmann C, Weitzner B, Panchaud A, et al. Pregnancy outcomes following use of escitalopram: a prospective comparative cohort study. *J Clin Pharmacol* 2012; **52**(5): 766-70.

For peer review only

15. Kjaersgaard MI, Parner ET, Vestergaard M, et al. Prenatal antidepressant exposure and risk of spontaneous abortion - a population-based study. *PLoS ONE* 2013; **8**(8): e72095.
16. Andersen JT, Andersen NL, Horwitz H, Poulsen HE, Jimenez-Solem E. Exposure to selective serotonin reuptake inhibitors in early pregnancy and the risk of miscarriage. *Obstet Gynecol* 2014; **124**(4): 655-61.
17. Abadie D, Hurault-Delarue C, Damase-Michel C, Montastruc JL, Lacroix I. Medication exposure and spontaneous abortion: A case-control study using a French medical database. *Clinical and Experimental Obstetrics and Gynecology* 2015; **42**(4): 431-6.
18. Johansen RL, Mortensen LH, Andersen AM, Hansen AV, Strandberg-Larsen K. Maternal use of selective serotonin reuptake inhibitors and risk of miscarriage - assessing potential biases. *Paediatr Perinat Epidemiol* 2015; **29**(1): 72-81.
19. Te Winkel B, Beghin D, Pistelli A, et al. Venlafaxine exposure in pregnancy, a multicenter ENTIS study. *Pharmacoepidemiology and Drug Safety* 2016; **25 (Supplement 3)**: 325-6.
20. Almeida ND, Basso O, Abrahamowicz M, Gagnon R, Tamblyn R. Risk of Miscarriage in Women Receiving Antidepressants in Early Pregnancy, Correcting for Induced Abortions. *Epidemiology* 2016; **27**(4): 538-46.
21. Evans-Hoeker EA, Eisenberg E, Diamond MP, et al. Major depression, antidepressant use, and male and female fertility. *Fertility and Sterility* 2018; **109**(5): 879-87.
22. Richardson JL, Martin F, Dunstan H, et al. Pregnancy outcomes following maternal venlafaxine use: A prospective observational comparative cohort study. *Reprod Toxicol* 2019; **84**: 108-13.
23. Wu P, Velez Edwards DR, Gorrindo P, et al. Association between First Trimester Antidepressant Use and Risk of Spontaneous Abortion. *Pharmacotherapy* 2019; **39**(9): 889-98.
24. Bahat M, Shechtman S, Diav-Citrin O. Pregnancy outcome after first trimester exposure to Duloxetine. *Reprod Toxicol* 2020; **97**: 3.
25. Kolding L, Ehrenstein V, Pedersen L, et al. Antidepressant use in pregnancy and severe cardiac malformations: Danish register-based study. *Bjog* 2021; **128**(12): 1949-57.
26. Ankarfeldt MZ, Petersen J, Andersen JT, et al. Duloxetine Exposure During Pregnancy and the Risk of Spontaneous and Elective Abortion: A Danish Nationwide Observational Study. *Drugs Real World Outcomes* 2021; **8**(3): 289-99.
27. Kitchin Á, Huerta C, Llorente-García A, Martínez D, Ortega P, Cea-Soriano L. The role of prenatal exposure to antidepressants, anxiolytic, and hypnotics and its underlying illness on the risk of miscarriage using BIFAP database. Pharmacoepidemiology and Drug Safety. 2022 Aug;31(8):901-12.
28. Ostenfeld A, Petersen TS, Pedersen LH, Westergaard HB, Løkkegaard EC, Andersen JT. Mirtazapine exposure in pregnancy and fetal safety: A nationwide cohort study. Acta Psychiatrica Scandinavica. 2022 Jun;145(6):557-67.

For peer review only

29. Giner-Soriano M, Prat-Vallverdu O, Vazquez ML, Vilaplana-Carnerero C, Vedia C, Gomez-Lumbreras AG, Morros R. Spontaneous abortion and drug exposure. Case-control study with SIDIAP database. InPHARMACOEPIDEMIOLOGY AND DRUG SAFETY 2022 Sep 1 (Vol. 31, pp. 238-239). 111 RIVER ST, HOBOKEN 07030-5774, NJ USA: WILEY.

For peer review only

S4 Table. Risk of bias assessment

Study	Domain 1: Bias due to confounding	Domain 2: Bias in classification of intervention	Domain 3: Bias in selection of participants into study	Domain 4: Bias due to deviations from intended interventions	Domain 5: Bias due to missing data	Domain 6: Bias in measurement of the outcome	Domain 7: Bias in selection of the reported result	Overall RoB rating
Chambers et al (1996)	**Critical**<br>No confounding adjustments made	**Critical**	**Critical**	**Critical**	**Critical**	**Critical**	**Critical**	**Critical**
Kulin et al (1998)	**Critical**<br>No information on confounding adjustments	**Critical**	**Critical**	**Critical**	**Critical**	**Critical**	**Critical**	**Critical**
Einarson et al (2001)	**Serious**<br>Justification: The paper only adjusted for maternal depression, therefore not adjusting for all three of the key confounding domains	**Serious**<br>Justification: Exposure was determined through self-report of the individual. This was measured prospectively whereby the outcome had not occurred so would be unlikely to affect the exposure classification, however this was not verified through physician or healthcare records. Therefore, intervention status could not have been classified correctly and could be differential	**Serious:**<br>Justification: The start of follow-up and intervention are not at the same point as women ringing the service enquiring about an exposure could have been taking it for weeks prior to call, which could induce bias as the intervention has differing effects over time, and no analytical techniques were put in place to minimise this bias	**Low**<br>Justification: Study was not undergone in an experimental context	**Serious**<br>Justification: Although complete case analysis was used, the number of exposed individuals and total sample was not stated. Therefore, there could have been missing data and there was no evidence to show that the missing data of outcome was not associated with the true value of the outcome	**Low**<br>Justification: The outcome was measured through questionnaire to individual, alongside the results being verified by a physician. Although the outcome assessors could have been aware of exposure status, this is unlikely to have influenced outcome classification.	**Moderate**<br>Justification: No statistical analysis plan was provided, however, all analysis that was stated in methods was completed and results given	**Serious**
Einarson et al (2003)	**Serious**	**Serious**	**Moderate**	**Low**	**Serious**	**Low**	**Moderate**	**Serious**

	Justification: Only indication for treatment and individual characteristics domain was adjusted for, meaning lifestyle exposures was not adjusted for resulting in serious risk of bias	Justification: Exposure was determined through self-report of the individual, however this was not verified through physician or healthcare records	Justification: Although start of intervention and follow-up were unlikely at the same time point, the study restricted to exposure during the first trimester only, therefore the differing effects of intervention across different time periods potentially inducing bias is minimised as restricted to first trimester	Justification: Study was not undergone in an experimental context	Justification: Although complete case analysis was utilised, the total sample of how many exposed individuals was not mentioned, only the number included in a complete case analysis. Therefore, there will likely be missing data, and as the outcome was determined through self-report there is no evidence that the missingness of the outcome was not related to its true value.	Justification: The outcome was determined through self-report questionnaire, however this was then confirmed through the individuals physician. Although the outcome assessors could have been aware of exposure status, this was unlikely to have influenced the reporting of the outcome	Justification: No statistical analysis plan was provided, however, all analysis that was stated in methods was completed and results given	
Sivojelezova et al (2005)	**Serious** Justification: Although presence of depression and maternal age was adjusted for, there was no factor that was adjusted for in lifestyle exposures. Therefore, if not all three of the confounding domains are	**Moderate** Justification: Self-report of exposure through a standardised intake form. In addition, letter sent to physician to confirm the information given by individual	**Serious** Justification: The start of intervention and follow-up were likely not at the same time point as women just rung up enquiring about exposure, in addition, no analysis methods were used to account for the differing effects of intervention across different time points	**Low** Justification: Study was not undergone in an experimental context	**Serious** Justification: Although complete case analysis was used, the number of exposed individuals and total sample was not stated. Therefore, there could have been missing data and there was no evidence to show that the missing data in the	**Low** Justification: The outcome was measured through a questionnaire which is not the most reliable, however, the questionnaire was also sent to healthcare professional to confirm. Therefore, the healthcare professional would likely know the exposure status of	**Moderate** Justification: No statistical analysis plan was given	**Serious**

	adjusted for it means serious rating				outcome was not associated with the the true value of the outcome.			
Chun-Fai-Chan et al (2005)	**Moderate** Justification: All three confounding domains of indication for treatment, lifestyle exposures and individual characteristics were adjusted for	**Moderate** Justification: Exposure was classified through self-report in questionnaire, however this was verified through a physician. Additionally, there was a follow-up questionnaire. As exposure was measured prospectively there was no risk of outcome influencing reporting of exposure	**Serious** Justification: The start of follow-up and intervention are not at the same point, which could induce bias as the intervention has differing effects over time, and no analytical techniques were put in place to minimise this bias	**Low** Justification: Study was not undergone in an experimental context	**Serious** Justification: Although complete case analysis was used, the number of exposed individuals and total sample was not stated. Therefore, there could have been missing data and there was no evidence to show that the missing data was not associated with the true value of the outcome. This is particularly relevant considering the outcome was individual self report.	**Low** Justification: outcome was measured through telephone conversation and then. Verified with physician. Although the outcome assessors could have been aware of exposure status	**Moderate** Justification: No statistical analysis plan was provided, however, all analysis that was stated in methods was completed and results given	**Serious**
Djulus et al (2006)	**Moderate** Justification: All three confounding factors of indication for treatment, individual characteristics	**Moderate** Justification: Women contacted the service regarding exposure and then details of exposure were taken including duration, timing	**Serious** Justification: The start of follow-up and intervention are not at the same point, which could induce bias as the intervention has differing effects over	**Low** Justification: Study was not undergone in an experimental context	**Serious** Justification: Complete case analysis was used but there was no total sample size or flow diagram indicating recruitment of	**Low** Justification: The outcome was measured through questionnaire to individual, alongside the results being verified by a physician. Although	**Moderate** Justification: No statistical analysis plan was provided, however, all analysis that was stated in methods was completed and results given	**Serious**

For peer review only

	and lifestyle exposures were adjusted for	and frequency - this questionnaire was then sent to practitioner for details to be confirmed	time, and no analytical techniques were put in place to minimise this bias		individuals and any exclusions. Missing data was not mentioned at all.	the outcome assessors could have been aware of exposure status, this is unlikely to have influenced outcome classification.		
Einarson et al (2009)	**Serious** Justification: Although the domains of individual characteristics and lifestyle exposures were adjusted for through maternal age and smoking, indication for treatment domain was not adjusted for	**Serious** Justification: Exposure was determined through self-report of the individual, however this was not verified through physician or healthcare records	**Moderate** Justification: Although start of intervention and follow-up were unlikely at the same time point, the study restricted to exposure during the first trimester only, therefore the differing effects of intervention across different time periods potentially inducing bias is minimised as restricted to first trimester	**Low** Justification: Study was not undergone in an experimental context	**Serious** Justification: Although complete case analysis was utilised, the total sample of how many exposed individuals was not mentioned, only the number included in a complete case analysis. Therefore, there will likely be missing data, and as the outcome was determined through self-report there is no evidence that the missingness of the outcome was not related to its true value.	**Serious** Justification: The outcome was reported by the mother/individual, however this was not verified with their physician or healthcare records. Therefore the exposure status known by the individual could influence their reporting of outcome classification.	**Moderate** Justification: No statistical analysis plan was provided, however, all analysis that was stated in methods was completed and results given	**Serious**
Nakhai-Pour et al (2010)	**Serious** Justification: It did not adjust for a factor in all three of the key confounding domains, as it did	**Moderate** Justification: The exposure classification was done through GP records and therefore all	**Moderate** Justification: Matching was employed, as well as controls selected independently to intervention status.	**Low** Justification: Study was not undergone in an experimental context	**Low** Justification: Smoking and maternal weight covariates were the only variables which had missing	**Low** Justification: The outcome was measured through the Quebec pregnancy register and therefore very	**Serious** Justification: No statistical analysis plan was given, additionally lots of data collected on covariates however	**Serious**

For peer review only

	not adjust for any factor of lifestyle exposure	information to classify intervention was recorded at start of intervention. Extent of non-differential misclassification would not be large.	The cases and controls were matched on gestational age so same risk period. Therefore, not at serious risk of bias in this domain		data. The missingness of these confounding factors is unlikely to be related to the true value of the outcome because the outcome was determined through pregnancy register rather than self-report	objective and not influenced by assessors	only some included in the adjustment model. Finally no results given for the sensitivity analysis.	
Paulus et al (2010)	**Critical** No information given on any confounding adjustments done	**Critical**	**Critical**	**Critical**	**Critical**	**Critical**	**Critical**	**Critical**
Chan et al (2011)	**Serious** Justification: Only individual characteristics and lifestyle exposure domains had a factor adjusted for in them - however indication for treatment was not adjusted for at all - therefore deemed serious as not all confounding domains satisfied.	**Serious** Justification: It was unclear the process of exposure classification, and indicated that only telephone interviews of self-report were used for exposure classification.	**Moderate** Justification: Start of intervention and follow-up were not the same, however, the analysis used was logistic time survival models so the differing effect of ads of different time periods was accounted for	**Low** Study was not undergone in an experimental context	**Serious** Justification: No information given on missing data.	**Low** Justification: Telephone interviews and medical records were used to determine the outcome, reliable method. Unclear whether outcome assessors were aware of exposure status, however unlikely to have influenced outcome determination because outcome is very objective	**Moderate** Justification: No statistical analysis plan was provided, however, all analysis that was stated in methods was completed and results given	**Serious**

For peer review only

Einarson et al (2011)	**Moderate**<br>Justification: All three confounding domains were adjusted for	**Serious**<br>Justification: Exposure was determined through self-report of the individual, however this was not verified through physician or healthcare records	**Serious**<br>Justification: The start of follow-up and intervention are not at the same point as women ringing the service enquiring about an exposure could have been taking it for weeks prior to call, which could induce bias as the intervention has differing effects over time, and no analytical techniques were put in place to minimise this bias	**Low**<br>Study was not undergone in an experimental context	**Serious**<br>Justification: Although complete case analysis was used, the number of exposed individuals/total sample was not stated. Therefore, there could have been missing data on participants that would have been excluded - this missing data could include outcome data which could be related to its true value as the outcome was self-report.	**Low**<br>Justification: The outcome was determined through self-report questionnaire; however this was then confirmed through the individuals physician. Although the outcome assessors could have been aware of exposure status	**Moderate**<br>Justification: No statistical analysis plan was provided, however, all analysis that was stated in methods was completed and results given	**Serious**
Ban et al (2012)	**Moderate**<br>Justification: All three confounding domains were adjusted for through some factor - including presence of depression, maternal age and smoking	**Moderate**<br>Justification: Exposure was classified through prescription databases where all drug usage was recorded - however just small difficulty of unsure the true ingested drug usage	**Serious**<br>Justification: Although prescription database was used, it is unlikely that the start of intervention and start of follow-up will be the same. Additionally, the authors did not use any statistical analysis including cox proportional regression to counteract	**Low**<br>Study was not undergone in an experimental context	**Low**<br>Justification: Missing data on covariates found, and the missingness would be unrelated to the outcome because they were taken from NHS databases before the outcome occurred. Additionally, no missing outcome data was found	**Low**<br>Justification: Outcome was measured reliably through healthcare records, however unclear whether outcome assessors would know exposure status but unlikely this would have influenced classification	**Moderate**<br>Justification: Although there was no statistical analysis plan there were no missing parts to the analysis.	**Serious**

For peer review only

Klieger-Grossman et al (2012)	**Moderate** Justification: All three confounding domains were adjusted for through some factor - including presence of depression, maternal age and smoking	**Moderate** Justification: Initial interview questioning exposure, then a follow-up interview to confirm answers given in original questioning. This was all done prospectively so no risk of outcome influencing exposure reporting	**Serious** Justification: The start of intervention and follow-up would not have been the same, therefore inducing potential bias, additionally no analytical techniques were put in place to account for this bias either through time dependent analysis or restricting the analysis to first trimester only	**Low** Study was not undergone in an experimental context	**Serious** Justification: Although complete case analysis was used, the number of exposed individuals and total sample was not stated. Therefore, there could have been missing data and there was no evidence to show that the missing data of outcome was not associated with the true value of it	**Low** Justification: Assessment of the outcome won't be influenced by the exposure	**Moderate** Justification: No statistical analysis plan was provided, however, all analysis that was stated in methods was completed and results given	**Serious**
Kjaesgaard et al (2013)	**Serious** Justification: only two of the three confounding domains were adjusted for - as the third domain of lifestyle exposures was not adjusted for	**Moderate** Justification: Prescription records were used to determine exposure, therefore likely to classify individuals correctly, however if there is misclassification this will likely be non-differential and attenuate towards the null	**Serious** Justification: Unlikely the start of follow-up and intervention were at the same time point. Additionally, no analysis was utilized to account foe the differing effect of intervention at different points during pregnancy	**Low** Study was not undergone in an experimental context	**Low** Justification: There was missing gestational age data, however this was replaced by the median of all non-missing values. Therefore covariate data is missing - as the outcome was determined through hospital registry it is unlikely that the true value of the outcome will influence the covariate	**Low** Justification: Outcome was assessed through codes on danish national hospital registry therefore very reliable method and outcome assessors unlikely aware of exposure status	**Moderate** Justification: No statistical analysis plan was provided, however, all analysis that was stated in methods was completed and results given	**Serious**

Andersen et al (2014)	**Serious**<br>Justification: No factor of lifestyle exposure was adjusted for in this analysis, only indication for treatment and individual characteristics was adjusted for	**Moderate**<br>Justification: exposure was classified through prescription databases therefore unlikely to be any issues with classification of exposure - however slight issues with true ingested drug usage.	**Moderate**<br>Justification: Prescription database was used meaning unclear whether start of intervention and start of follow-up were at the same time point. However, a cox proportional hazards regression was used which counteracts the differing effects that exposure will have at different time points during pregnancy.	**Low**<br>Study was not undergone in an experimental context	**Low**<br>Justification: Minimal missing data was found on the confounding factors used, additionally, outcome was measured through hospital records therefore unlikely that the true value of the outcome will be influencing the missingness. No missing outcome data.	**Moderate**<br>Justification: Outcome was measured in a very objective and reliable method through hospital records, unclear whether outcome assessors knew exposure status however this is unlikely to influence exposure status determination. However, the method of outcome assessment could have differed between interventions as individuals on antidepressants would be more likely to come into hospital as more contact with healthcare services compared to unexposed individuals.	**Moderate**<br>Justification: No statistical plan was given, however all results that were mentioned in method were given	**Serious**
Abadie et al (2015)	**Serious**<br>Justification: Only one of the three confounding domains had anything adjusted for - only maternal age had been accounted for -	**Moderate**<br>Justification: The CRPV was notified by healthcare professional regarding exposure, then a questionnaire was sent across and confirmation of	**Serious**<br>Justification: The authors did employ matching, however the controls were not independently selected of the intervention status. Additionally, they didn't match on	**Low**<br>Study was not undergone in an experimental context	**Low**<br>Justification: There was missing data on the confounding factors, as the outcome was measured through a database it is unlikely the missingness of	**Low**<br>Justification: The outcome was measured through database administrative records, therefore very reliable	**Moderate**<br>Justification: Although there was no statistical analysis plan there were no missing parts to the analysis.	**Serious**

For peer review only

	therefore not all three confounding domains satisfied and serious risk of bias	current medication taken currently, so low risk of differential misclassification.	gestational age so time at risk would have differed between cases and controls		covariates is related to true value of outcome. However, no missing data for the outcome was found.			
Johansen et al (2015)	**Serious** Justification: Although this paper did a large amount of adjustments, especially in relation to confounding by indication, which most other papers didn't, it still didn't adjust for all three confounding domains as it missed adjusting for factors on lifestyle exposure	**Moderate** Justification: Both prescription records and self-report was used to assess exposure classification for individuals, therefore very reliable method using both - it did find differences between the two methods so likely there will be misclassification however very minimal	**Moderate** Justification: It is unlikely the start of intervention and start of follow-up were at the same point, however a cox proportional hazards regression was used to account for the differing effects of exposure at different time points during pregnancy	**Low** Study was not undergone in an experimental context	**Low** Justification: A flow diagram illustrating the total sample and any excluded individuals or missing data. This highlighted there was missing data in the exposure, covariates and outcome. However, as the outcome was assessed through healthcare registers and bypassing the mother its unlikely the true value of the outcome would cause this missingness.	**Low** Justification: The outcome was assessed through healthcare registers and the outcome assessors would be unaware of exposure status therefore very little risk regarding exposure influencing outcome status, especially considering the objective nature of the outcome	**Serious** Justification: No statistical analysis plan was given, and information on confounders was taken which were not used in the adjustment	**Serious**
Te winkel et al (2016)	**Critical** No information given on any confounding adjustments done	**Critical**	**Critical**	**Critical**	**Critical**	**Critical**	**Critical**	**Critical**
Almeida et al (2016)	**Serious** Justification: Only indication for	**Moderate** Justification: exposure was	**Serious** Justification: Although	**Low** Study wasn't undergone in an	**Low** Justification: There was missing data	**Low** Justification: Outcome was	**Moderate** Justification: No statistical plan was	**Serious**

	treatment and individual characteristics domain had been adjusted for, whereas no factors were adjusted for in lifestyle exposures	classified through prescription database, therefore highly reliable. However, there may be some discrepancies as to the actual ingested drug usage.	prescription database was used, it is unlikely that the start of intervention and start of follow-up will be the same. Additionally, the authors did not use any statistical analysis including cox proportional regression to counteract the differing effects that exposure will have at different time points during pregnancy.	experimental context	on the confounding factors, as the outcome was measured through the physician rather than self-report it is unlikely the true value of the outcome would be influencing the missingness of these covariates . However, no missing data for the outcome was found.	assessed through ICD-9 codes of physician notes, therefore very objective. Unclear whether outcome assessors know exposure status, however this is unlikely to impact the outcome classification	given, however all results that were mentioned in method were given	
Evans-Hoeker et al (2018)	**Moderate** Justification: All three confounding domains were adjusted for	**Moderate** Justification: The medication exposure was assessed throughout a previous RCT, with regulators of the trial ensuring accurate medication exposure classification	**Serious** Justification: It is unlikely the start of intervention and follow-up were at the same time point, additionally no analysis was utilised to account for the differing effect of intervention at differing points during pregnancy	**Low** Study wasn't undergone in an experimental context	**Moderate** Justification: 100% of individuals were included from the trial into the study. Less than 10% of PHQ scores were missing, therefore missing covariate data, no evidence that this missing data was not driven by the true value of the outcome.	**Low** Justification: The outcome was assessed throughout the RCT through testing and so a very thorough and reliable method. Unlikely that outcome assessors knowing the exposure would influence their classification of it	**Moderate** Justification: No statistical analysis plan was provided, however, all analysis that was stated in methods was completed and results given	**Serious**
Richardson et al (2019)	**Moderate** Justification: All three confounding domains had a factor that had been adjusted for	**Moderate** Justification: Exposure classification measured prospectively and by a healthcare	**Moderate** Justification: The start of intervention and follow-up were likely not at the same time point as individual rung up	**Low** Study wasn't undergone in an experimental context	**Low** Justification: They don't have complete data as missing data was reported in only the confounding	**Low** Justification: The outcome is a very objective measure. The outcome was measured through clinician self-report,	**Moderate** Justification: Although there was no statistical analysis plan there were no missing parts to the analysis.	**Moderate**

For peer review only

	including presence of maternal depression, maternal age and alcohol	professional over the phone. Classification of intervention status would not be influenced by the outcome	not necessarily at start of exposure. However, analysis used was time dependent cox models therefore the differing effect of intervention across different time periods was accounted for.		variables, however the incomplete data for these covariates is unlikely to have been influenced by the true value of the outcome because this was determined through clinician rather than self report.	and although exposure would have been known to them, this is unlikely to have influenced the outcome reporting.		
Wu et al (2019)	**Serious** Justification: Only individual characteristics and lifestyle exposure domains had a factor adjusted for in them - however indication for treatment was not adjusted for at all - therefore deemed serious as not all confounding domains satisfied.	**Serious** Justification: The exposure was classified at the end of first trimester interview - so a time point after the start of follow up. Additionally in a lot of the cases the exposure was recorded after the SA had already happened. Potentially differential misclassification between exposure and outcome individuals	**Moderate** Start of intervention and follow-up did not coincide, however a cox proportional hazard survival model was utilised to counteract the differing effects that exposure will have at different time points during pregnancy	**Low** Study wasn't undergone in an experimental context	**Serious** Justification: A flow diagram and tables with the details of missing data was illustrated. Individuals were excluded if they had missing data from the interview, this results in missing outcome data that could have been a result of the true value of the outcome.	**Low** Justification: The outcome was measured through a questionnaire, this was then confirmed through medical records. Although outcome assessors could have been aware of the exposure status it is unlikely assessment of outcome would have been influenced by this.	**Moderate** Justification: No statistical analysis plan was given	**Serious**
Bahat et al (2020)	**Serious** Justification: Unclear what they adjusted for	**No information**	**Moderate** Justification: It was unclear whether the start of intervention	**Low** Study wasn't undergone in an	**No information**	**No information**	**Serious** Justification: No statistical analysis plan was given,	**Serious**

For peer review only

	in this analysis and was not stated, however an adjusted estimate was given so some level of confounding was accounted for		and follow-up were at the same point, however, the analysis was restricted to first trimester where there is thought to be similar effects of Ads across first trimester so accounting for this	experimental context			additionally not all of the analysis that said was going to be conducted was released as results	
Kolding et al (2021)	**Critical** No confounding adjustment done	**Critical**	**Critical**	**Critical**	**Critical**	**Critical**	**Critical**	**Critical**
Ankarfeldt et al (2021)	**Moderate** Justification: All three confounding domains of indication for treatment, lifestyle exposures and individual characteristics had one factor adjusted for	**Moderate** Justification: Exposure was classified through prescription databases therefore likely to be reliable, however may not reflect true ingested drug usage.	**Moderate** Justification: Start of intervention and follow-up were likely not at same time point. However, a cox proportional hazard survival model was utilised to counteract the differing effects that exposure will have at different time points during pregnancy	**Low** Justification: Study wasn't undergone in an experimental context	**Low:** Justification: There was a small number of missing values for two covariates, however as this was not influenced by the value of the outcome and the missing values were imputed it poses minimal risk of bias.	**Low** Justification: The outcome was measured through patient registers, therefore very objective and reliable.	**Moderate** Justification: No statistical analysis plan was given but all of the analysis planned and stated in methods was conducted and given.	**Moderate**
Kitchin et al. (2022)	**Moderate** Justification: All three confounding domains of indication for treatment, lifestyle exposures and	**Moderate** Justification: Exposure was classified through prescription databases therefore likely to be reliable, however may not	**Serious** Justification: Although prescription database was used, it is unlikely that the start of intervention and start of follow-up will be the same.	**Low** Study wasn't undergone in an experimental context	**Low:** Justification: The only missing values were the LMP values which were imputed through subtracting 74 days off recorded entry date, therefore	**Low** Justification: Outcome was determined through ICD codes in BIFAP database, therefore very objective and reliable.	**Moderate** Justification: No statistical analysis plan was given but all of the analysis planned and stated in methods was conducted and given.	**Serious**

For peer review only

	individual characteristics had one factor adjusted for	reflect true ingested drug usage.	Additionally, the authors did not use any statistical analysis including cox proportional regression to counteract		poses minimal risk of bias.			
Ostenfeld et al. (2022)	**Serious** Justification: Only individual characteristics and lifestyle exposure domains had a factor adjusted for in them - however indication for treatment was not adjusted for at all - therefore deemed serious as not all confounding domains satisfied	**Moderate** Justification: Exposure was classified through prescription databases therefore likely to be reliable, however may not reflect true ingested drug usage.	**Moderate** Justification: It was unclear whether the start of intervention and follow-up were at the same point, however, the analysis did take into account various timepoints within exposure periods.	**Low** Study wasn't undergone in an experimental context	**Moderate** Justification: Not all individuals in the cohort were included in the analysis due to missing/implausible gestation values; these missing values could be associated with the outcome. Additionally, there were other missing values that were imputed using the mode value.	**Low** Justification: Outcome identified in national hospital register, therefore very objective and reliable	**Moderate** Justification: No statistical analysis plan was given but all of the analysis planned and stated in methods was conducted and given.	**Serious**
Giner-Soriano et al. (2022)	**Serious** Justification: Only individual characteristics and lifestyle exposure domains had a factor adjusted for in them - however indication for treatment was not adjusted for at all - therefore	**Moderate** Justification: Exposure was classified through prescription databases therefore likely to be reliable, however may not reflect true ingested drug usage.	**Serious** Justification: Although prescription database was used, it is unlikely that the start of intervention and start of follow-up will be the same. Additionally, the authors did not use any statistical analysis including cox proportional	**Low** Study wasn't undergone in an experimental context	**No information**	**Low** Justification: Outcome identified in primary health care records of Catalan institute, therefore very objective and reliable	**Moderate** Justification: No statistical analysis plan was given but all of the analysis planned and stated in methods was conducted and given.	**Serious**

	deemed serious as not all confounding domains satisfied		regression to counteract					

For peer review only

S5 Table. Confounding adjustments completed

Study	Confounding domain												
	Indication for treatment		Individual characteristics								Lifestyle exposures		
	Presence of depression	Severity of depression	History of SA	Maternal age	Gestational age	Polyphar macy	SEP	Ethnicity	BMI	Comorbi dities	Smoking	Alcohol	Recreational drugs
Chambers et al (1996)													
Kulin et al (1998)													
Einarson et al (2001)	■												
Einarson et al (2003)	■				■								
Sivojelezova et al (2005)	■			■	■								
Chun-Fai-Chan et al (2005)	■			■	■						■	■	
Djulus et al (2006)	■			■						■	■	■	
Einarson et al (2009)			■	■	■						■	■	
Nakhai-Pour et al (2010)	■	■	■	■	■		■			■			
Paulus et al (2010)													
Chan et al (2011)				■			■	■	■		■	■	
Einarson et al (2011)	■			■	■						■	■	
Ban et al (2012)	■			■			■		■		■		
Klieger-Grossman et al (2012)	■			■	■						■	■	
Kjaesgaard et al (2013)	■			■			■			■			
Andersen et al (2014)	■		■	■		■	■						
Abadie et al (2015)				■									
Johansen et al (2015)	■	■	■	■	■		■						
Tw Winkel et al (2016)													
Almeida et al (2016)	■			■		■	■			■			
Evans-Hoeker et al (2018)	■	■		■			■	■			■		
Richardson et al (2019)	■		■	■	■						■	■	■
Wu et al (2019)			■	■								■	

For peer review only

**Bahat et al (2020)**													
**Kolding et al (2021)**													
**Ankarfeldt et al (2021)**													
**Kitchin et al. (2022)**													
**Ostenfeld et al. (2022)**													
**Giner-Soriano et al. (2022)**													

Green highlighted box indicating factor adjusted for and blank box indicating factor was not adjusted for

For peer review only

S6 Table. Meta-analysis results

Analysis type		Number of studies	Number of adjusted studies used	Summary effect estimate (95% confidence interval)	Heterogeneity
**Main analysis**					
**Studies in the general population**	AD use vs unexposed comparator	29	18	1.24 (1.18-1.31)	69.2%
	SSRI vs unexposed comparator	12	6	1.29 (1.15-1.44)	81.3%
	SNRI vs unexposed comparator	5	2	1.42 (1.11 – 1.81)	46.9%
**Studies restricting population to women with depression**	AD use vs unmedicated depressed comparator	6	4	1.16 (1.04 – 1.31)	58.6%
	SSRI v another AD	5	2	1.03 (0.93-1.15)	0%
	SNRI vs another AD	3	2	1.19 (1.02 – 1.39)	0%
**Sensitivity analysis restricting to studies of sufficient quality**					
**Studies in the general population**	AD use vs unexposed comparator	24	18	1.24 (1.17-1.31)	72.10%
	SSRI vs unexposed comparator	9	6	1.30 (1.15-1.46)	86.0%
	SNRI vs unexposed comparator	3	2	1.17 (1.01-1.36)	0%
**Sensitivity analysis restricting to studies utilising prescription database as method of exposure classification**					
**Studies in the general population**	AD use vs unexposed comparator	11	10	1.19 (1.13-1.26)	83.8%
	SSRI use vs unexposed comparator	4	4	1.25 (1.09-1.43)	94.2%
	SNRI use vs unexposed comparator	1	1	1.14 (0.96-1.35)	NA

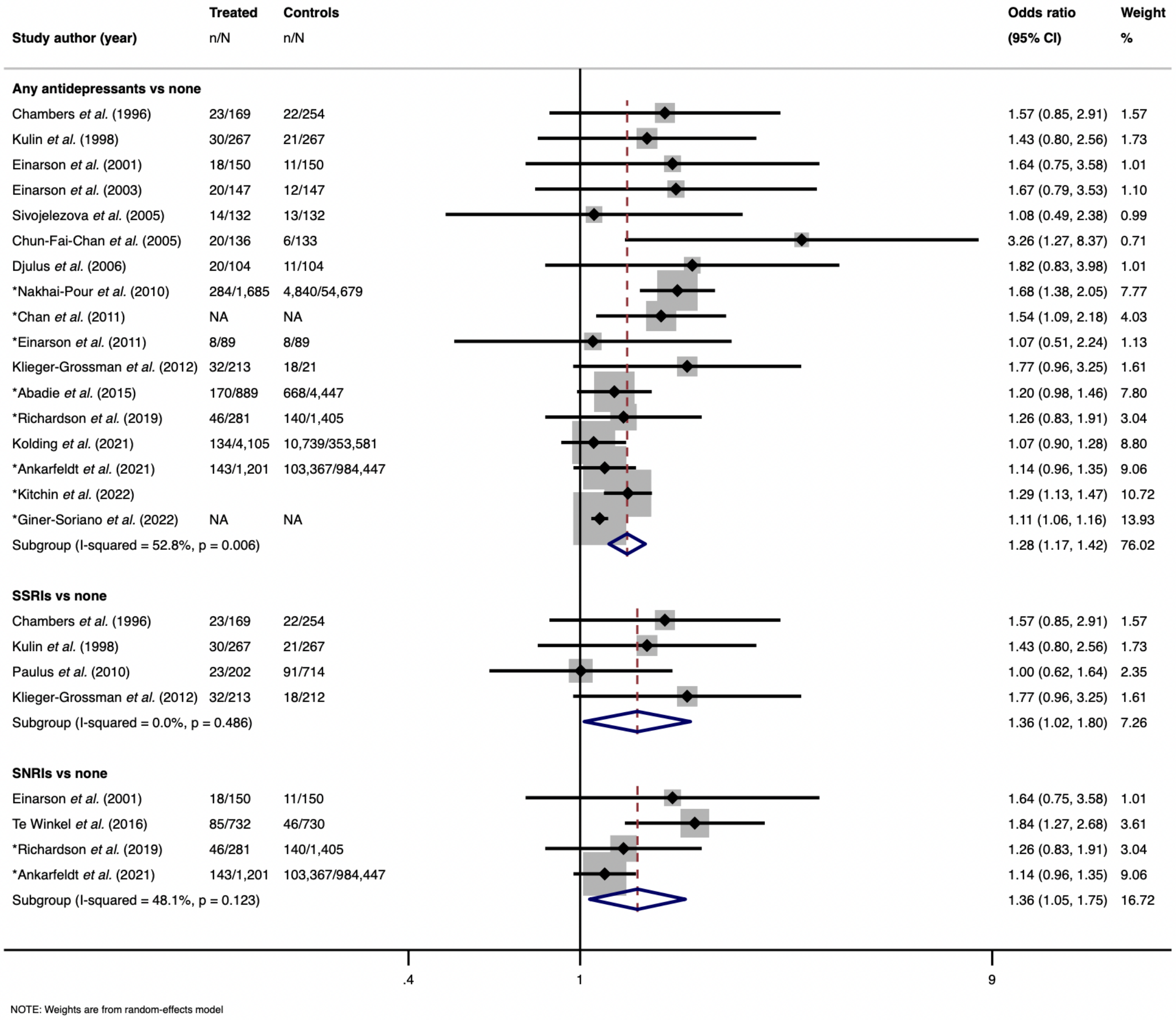

S1 Figure. Sensitivity analysis utilising only papers reporting odds ratios.

General population sensitivity analysis including only papers which included odds ratio, and excluding papers reporting hazards and risk ratio

*These studies had an adjusted estimate from multivariable model which was pooled in the meta-analysis rather than raw data, therefore the treated and controls (n/N) data does not correspond to the odds ratio shown.

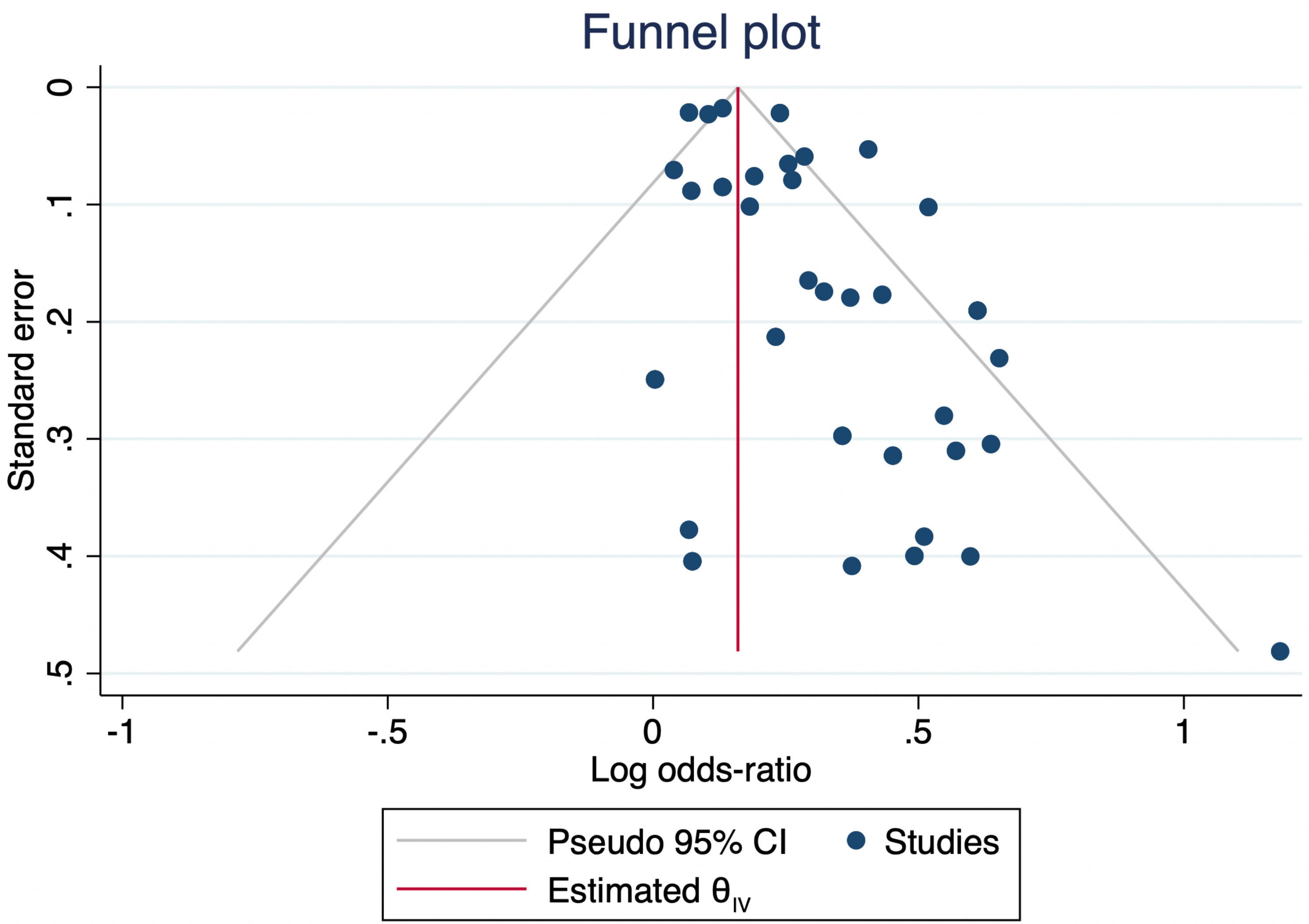

S2 Figure. Funnel plot

For peer review only

PRISMA 2020 Checklist

Section and Topic	Item #	Checklist item	Location where item is reported
**TITLE**			
Title	1	Identify the report as a systematic review.	Pg1
**ABSTRACT**			
Abstract	2	See the PRISMA 2020 for Abstracts checklist.	Pg2
**INTRODUCTION**			
Rationale	3	Describe the rationale for the review in the context of existing knowledge.	Pg5
Objectives	4	Provide an explicit statement of the objective(s) or question(s) the review addresses.	Pg5
**METHODS**			
Eligibility criteria	5	Specify the inclusion and exclusion criteria for the review and how studies were grouped for the syntheses.	Pg6
Information sources	6	Specify all databases, registers, websites, organisations, reference lists and other sources searched or consulted to identify studies. Specify the date when each source was last searched or consulted.	Pg6
Search strategy	7	Present the full search strategies for all databases, registers and websites, including any filters and limits used.	Appendix S1
Selection process	8	Specify the methods used to decide whether a study met the inclusion criteria of the review, including how many reviewers screened each record and each report retrieved, whether they worked independently, and if applicable, details of automation tools used in the process.	Pg6 & 7
Data collection process	9	Specify the methods used to collect data from reports, including how many reviewers collected data from each report, whether they worked independently, any processes for obtaining or confirming data from study investigators, and if applicable, details of automation tools used in the process.	Pg7
Data items	10a	List and define all outcomes for which data were sought. Specify whether all results that were compatible with each outcome domain in each study were sought (e.g. for all measures, time points, analyses), and if not, the methods used to decide which results to collect.	Pg7
	10b	List and define all other variables for which data were sought (e.g. participant and intervention characteristics, funding sources). Describe any assumptions made about any missing or unclear information.	Pg7
Study risk of bias assessment	11	Specify the methods used to assess risk of bias in the included studies, including details of the tool(s) used, how many reviewers assessed each study and whether they worked independently, and if applicable, details of automation tools used in the process.	Pg7
Effect measures	12	Specify for each outcome the effect measure(s) (e.g. risk ratio, mean difference) used in the synthesis or presentation of results.	Pg7
Synthesis methods	13a	Describe the processes used to decide which studies were eligible for each synthesis (e.g. tabulating the study intervention characteristics and comparing against the planned groups for each synthesis (item #5)).	Pg7
	13b	Describe any methods required to prepare the data for presentation or synthesis, such as handling of missing summary statistics, or data conversions.	Pg7
	13c	Describe any methods used to tabulate or visually display results of individual studies and syntheses.	Pg7
	13d	Describe any methods used to synthesize results and provide a rationale for the choice(s). If meta-analysis was performed, describe the model(s), method(s) to identify the presence and extent of statistical heterogeneity, and software package(s) used.	Pg7 & pg8
	13e	Describe any methods used to explore possible causes of heterogeneity among study results (e.g. subgroup analysis, meta-regression).	Pg 7 & 8
	13f	Describe any sensitivity analyses conducted to assess robustness of the synthesized results.	Pg 8
Reporting bias assessment	14	Describe any methods used to assess risk of bias due to missing results in a synthesis (arising from reporting biases).	Pg 7

For peer review only - http://bmjopen.bmj.com/site/about/guidelines.xhtml

PRISMA 2020 Checklist

Section and Topic	Item #	Checklist item	Location where item is reported
Certainty assessment	15	Describe any methods used to assess certainty (or confidence) in the body of evidence for an outcome.	Pg pg 8
**RESULTS**			
Study selection	16a	Describe the results of the search and selection process, from the number of records identified in the search to the number of studies included in the review, ideally using a flow diagram.	Figure s1
	16b	Cite studies that might appear to meet the inclusion criteria, but which were excluded, and explain why they were excluded.	Table s1
Study characteristics	17	Cite each included study and present its characteristics.	Appendix s2
Risk of bias in studies	18	Present assessments of risk of bias for each included study.	Appendix s4
Results of individual studies	19	For all outcomes, present, for each study: (a) summary statistics for each group (where appropriate) and (b) an effect estimate and its precision (e.g. confidence/credible interval), ideally using structured tables or plots.	Figure s1, s2, s3 and appendix s3
Results of syntheses	20a	For each synthesis, briefly summarise the characteristics and risk of bias among contributing studies.	Appendix s4<br>Pg10
	20b	Present results of all statistical syntheses conducted. If meta-analysis was done, present for each the summary estimate and its precision (e.g. confidence/credible interval) and measures of statistical heterogeneity. If comparing groups, describe the direction of the effect.	Appendix s3<br>Pg9
	20c	Present results of all investigations of possible causes of heterogeneity among study results.	Appendix s3<br>Pg 14
	20d	Present results of all sensitivity analyses conducted to assess the robustness of the synthesized results.	Appendix s3
Reporting biases	21	Present assessments of risk of bias due to missing results (arising from reporting biases) for each synthesis assessed.	NA
Certainty of evidence	22	Present assessments of certainty (or confidence) in the body of evidence for each outcome assessed.	Appendix s3<br>Pg 9
**DISCUSSION**			
Discussion	23a	Provide a general interpretation of the results in the context of other evidence.	Pg 11 & 12
	23b	Discuss any limitations of the evidence included in the review.	Pg 12 & 13
	23c	Discuss any limitations of the review processes used.	Pg 14
	23d	Discuss implications of the results for practice, policy, and future research.	Pg 15
**OTHER INFORMATION**			
Registration and protocol	24a	Provide registration information for the review, including register name and registration number, or state that the review was not registered.	Pg 8
	24b	Indicate where the review protocol can be accessed, or state that a protocol was not prepared.	Pg 8

For peer review only - http://bmjopen.bmj.com/site/about/guidelines.xhtml

PRISMA 2020 Checklist

Section and Topic	Item #	Checklist item	Location where item is reported
	24c	Describe and explain any amendments to information provided at registration or in the protocol.	NA
Support	25	Describe sources of financial or non-financial support for the review, and the role of the funders or sponsors in the review.	Pg 4
Competing interests	26	Declare any competing interests of review authors.	Pg 16
Availability of data, code and other materials	27	Report which of the following are publicly available and where they can be found: template data collection forms; data extracted from included studies; data used for all analyses; analytic code; any other materials used in the review.	Throughout

From: Page MJ, McKenzie JE, Bossuyt PM, Boutron I, Hoffmann TC, Mulrow CD, et al. The PRISMA 2020 statement: an updated guideline for reporting systematic reviews. BMJ 2021;372:n71. doi: 10.1136/bmj.n71

For more information, visit: http://www.prisma-statement.org/

For peer review only
